# Aziridine Group Transfer via Transient *N*-Aziridinyl Radicals

**DOI:** 10.1021/jacs.4c14169

**Published:** 2024-11-04

**Authors:** Promita Biswas, Asim Maity, Matthew T. Figgins, David C. Powers

**Affiliations:** Department of Chemistry, Texas A&M University, College Station, Texas 77843, United States

## Abstract

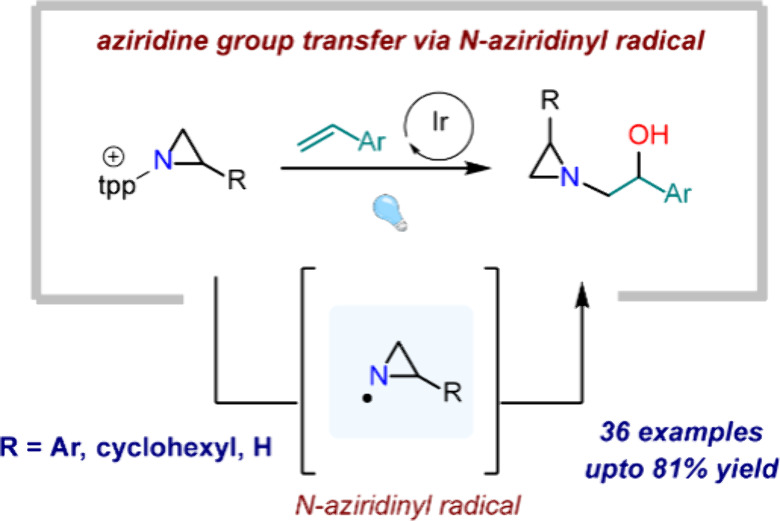

Aziridines are the smallest nitrogen-containing heterocycles. Strain-enhanced electrophilicity renders aziridines useful synthetic intermediates and gives rise to biological activity. Classical aziridine syntheses—based on either [2 + 1] cycloadditions or intramolecular substitution chemistry—assemble aziridines from acyclic precursors. Here, we introduce *N-*aziridinyl radicals as a reactive intermediate that enables the transfer of intact aziridine fragments in organic synthesis. Transient *N-*aziridinyl radicals are generated by the reductive activation of *N*-pyridinium aziridines and are directly characterized by spin-trapped EPR spectroscopy. In the presence of O_2_, *N*-aziridinyl radicals are added to styrenyl olefins to afford 1,2-hydroxyaziridination products. These results establish aziridinyl radicals as new reactive intermediates in synthetic chemistry and demonstrate aziridine group transfer as a viable synthetic disconnection.

Aziridines are important synthetic building blocks and represent electrophilic pharmacophores in a variety of organic small molecule therapeutics and natural products (Figure 1a).^1^ Aziridines are typically constructed via [2 + 1] cycloadditions between either olefins with nitrene equivalents or imines with carbene equivalents, or via intramolecular nucleophilic substitution chemistry within prefunctionalized substrates (Figure 1b).^2^*N*-Alkylation and metal-catalyzed C–N cross-coupling reactions provide opportunities to functionalize the exocyclic N–H valence of preformed aziridines;^3^ however, ring-opening chemistry to deliver 1,2-aminofunctionalization products is often observed during these transformations.^4^ Methods to transfer intact aziridines to relatively unfunctionalized substrates, such as C–H bonds or olefins, are currently unavailable.

**Figure 1:**
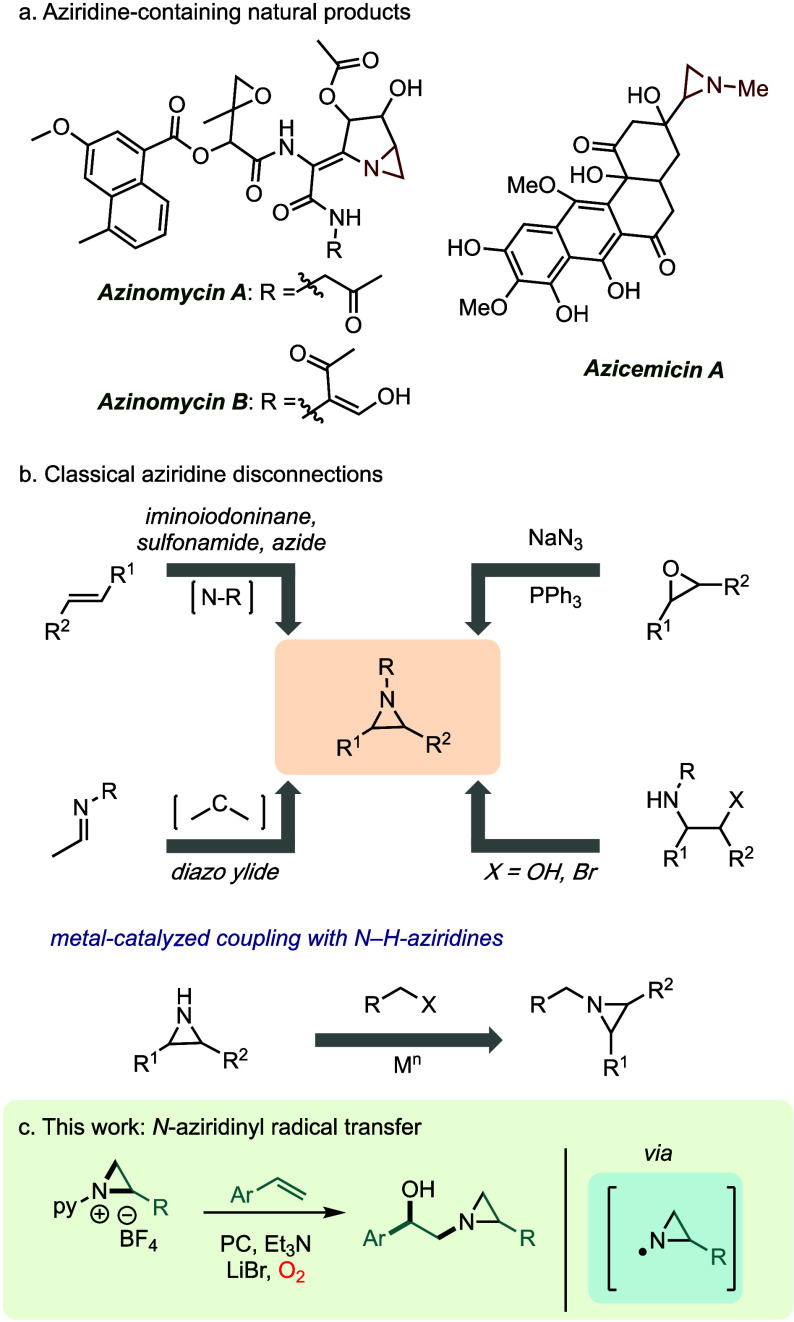
(a) Selected aziridine-containing natural products. (b) Classical synthetic disconnections for aziridines and *N*-functionalization chemistry. (c) Here, we report the synthesis and reactivity of *N*-aziridinyl radicals, which engage in intermolecular olefin addition chemistry. PC = photocatalyst.

Aziridine transfer to olefins via 1,2-aziridine functionalization would complement extant nitrene transfer reactions and provide new disconnections in aziridination chemistry. Based on the burgeoning literature of *N*-centered radical addition to olefins,^5,6^ we envisioned that access to *N*-aziridinyl radicals would enable aziridine transfer chemistry. Given the strength of aziridine N–H bonds (∼92 kcal/mol)^7^ in comparison to the C–N bonds of the strained three-membered ring (∼54 kcal/mol),^8^ we viewed direct generation of *N*-aziridinyl radicals from N–H precursors as unlikely. Inspired by strategies to access *N*-centered radicals by single-electron transfer (SET) between R_2_N–X (LG = −Cl, −Br, −O_2_CR) reagents and either transition metal catalysts^9^ or photocatalysts,^10^ we speculated that an *N-*substituted aziridine featuring a (photo)cleavable *N-*substituent could provide selective entry to aziridine radical chemistry.

We previously developed *N-*pyridinium aziridines as electrophiles in C–N cross coupling chemistry.^11^ We envisioned facile access to *N*-aziridinyl radicals from these precursors via reductive activation of the N–N bond.^12,13^ Here, we demonstrate that the reductive photoactivation of *N*-pyridinium aziridines generates *N*-aziridinyl radicals. DFT studies indicate the *N*-aziridinyl radical is planar with the unpaired spin in a p-orbital, which we hypothesize results in electrophilic reactivity.^14^ In the synthetic context, the transient *N*-aziridinyl radicals can be trapped with olefinic substrates in the presence of O_2_ to afford 1,2-hydroxyaziridination products (Figure 1c). Together, these results establish *N-*aziridinyl radicals as a new reactive intermediate for synthetic chemistry and demonstrate aziridine group-transfer as a viable synthetic disconnection.

We initiated the development of aziridine transfer chemistry with *N*-pyridinium aziridine **2a** (tpp = triphenylpyridinium), which displays a reductive electrochemical feature at −0.85 V vs Fc^+^/Fc.^15^ We hypothesized that reductive quenching of an appropriate photocatalyst would reveal an *N*-aziridinyl radical. Accordingly, photolysis of a MeCN solution of **2a** in the presence of Ir(ppy)_3_ (Ir(III)*/Ir(IV) = −1.73 V vs Fc^+^/Fc),^16^ triethyl amine, and radical acceptor **1** afforded **3**, the product of aziridine transfer (eq 1).
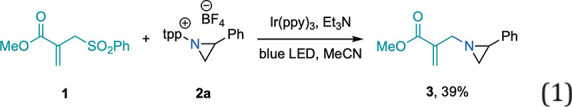
1

With the conditions in hand to promote *N-*aziridinyl radical transfer, we sought to engage this novel fragment in olefin addition chemistry. To this end, photolysis (blue LED) of **2a**, styrene, Ir(ppy)_3_ (1.0 mol %), and Et_3_N in an O_2_-saturated H_2_O:MeCN solution afforded compound **5a**, the product of olefin 1,2-hydroxyaziridination, in 35% yield (1:1 *dr*, Table 1, entry 1). Addition of LiBr increased the yield of hydroxyazirdine **5a** to 42% (50 mol % LiBr, entry 2) and 69% (1.0 equiv LiBr, entry 3). LiOTf and LiBF_4_ also have a positive impact on the efficiency of hydroxyaziridination; addition of [TBA]Br has no impact on reaction efficiency. These observations suggest Li^+^ serves as a Lewis acid while Br^–^ does not impact the developed reaction. No aziridine transfer products were obtained in the absence of Et_3_N, a photocatalyst, or light (entries 4–6). Changes to the relative stoichiometries of olefin **4a** and aziridine radical precursor **2a** did not improve the efficiency of olefin hydroxyaziridination (entries 7 and 8). Using unsubstituted *N*-pyridinium aziridine **2a′** afforded only a 29% yield of **5a**, which is consistent with the more negative reduction potential of unsubstituted pyridinium aziridines as compared to 2,4,6-triphenylpyridinium aziridines.^15^ In the absence of water, the reaction afforded 62% **5a** but was less clean which complicated purification, and thus the optimized conditions utilized a 1:1 H_2_O:MeCN mixture (see Tables S1–S5 for optimization details). Compound **5a** can be envisioned as the product of epoxide opening with an aziridine nucleophile, which, to our knowledge, is unknown.

**Table 1:**
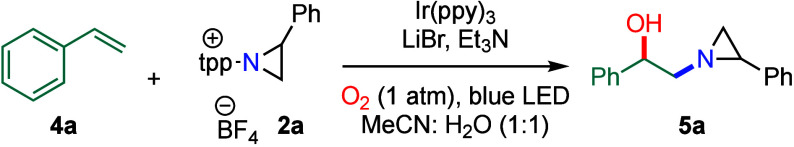
Olefin 1,2-Hydroxyaziridination^a^

entry	variation from standard conditions	yield^b^ (%)
1	no LiBr	35
2	LiBr (50 mol %)	42
3	none	69 (65)
4	no PC	0
5	no Et_3_N	0
6	no light	0
7	**4a** (1.0 equiv), **2a** (1.5 equiv)	41
8	**4a** (3.0 equiv) of styrene	45
9	py-aziridine was used instead of tpp-aziridine	29
10	MeCN	62

a Optimized conditions: **4a** (0.15 mmol), **2a** (0.10 mmol), Ir(ppy)_3_ (1.0 mol %), Et_3_N (0.2 mmol), LiBr (0.10 mmol) in MeCN:H_2_O (1:1, 2.0 mL) under blue LED irradiation.

b NMR yields (isolated yield).

With conditions for *N*-aziridinyl radical transfer, we used radical precursor **2a** to canvass the reactivity of this fragment against various olefinic partners (**4a**-**4o**) (Figure 2). Substrates with electron-donating groups such as 4-Me– (**4b**) and 4-OMe– (**4c**) substituents afforded **5b** and **5c** in 58 and 49% yield, respectively. *Para*-fluorinated (**4d**) and -chlorinated (**4e**) substrates engage in efficient hydroxyaziridination, and *ortho*-brominated **4f** affords the corresponding hydroxyaziridine in 56% yield, which evidence the compatibility of the protocol with large ortho substituents. Electron-deficient substrates such as **4g** (−CO_2_Et), **4h** (−CN), and **4i** (−CF_3_) are hydroxyaziridinated to **5g**, **5h**, and **5i** in 56–64% yield; 3-nitrostyrene furnished the corresponding aziridine-addition product **5j** in 81% isolated yield. Heterocycle-containing substrates are also compatible with the reaction conditions, with 4-vinylpyridine delivering product **5k** in 40% yield. 1,1-Disubstituted styrenes were competent substrates: α-Methyl- and phenyl-substituted styrene (**4l** and **4m**) gave the hydroxylated products (**5l** and **5m**) with moderate yields (41% and 44%, respectively); 1,2-disubstituted derivatives were not productive coupling partners. Styrenes derived from pharmaceuticals such as indomethacin and ibuprofen also provided the desired products (**5n** and **5o**) in 48% and 54% yield, respectively. Consistent with the high N–H BDE and proclivity of aminyl radicals to engage in H atom abstraction reactions, N–H aziridines were often observed as byproducts of the developed aziridine transfer chemistry.^17^ Finally, attempts to translate this chemistry to aliphatic olefins or enol ether derivatives were unsuccessful (Figure S1).

**Figure 2:**
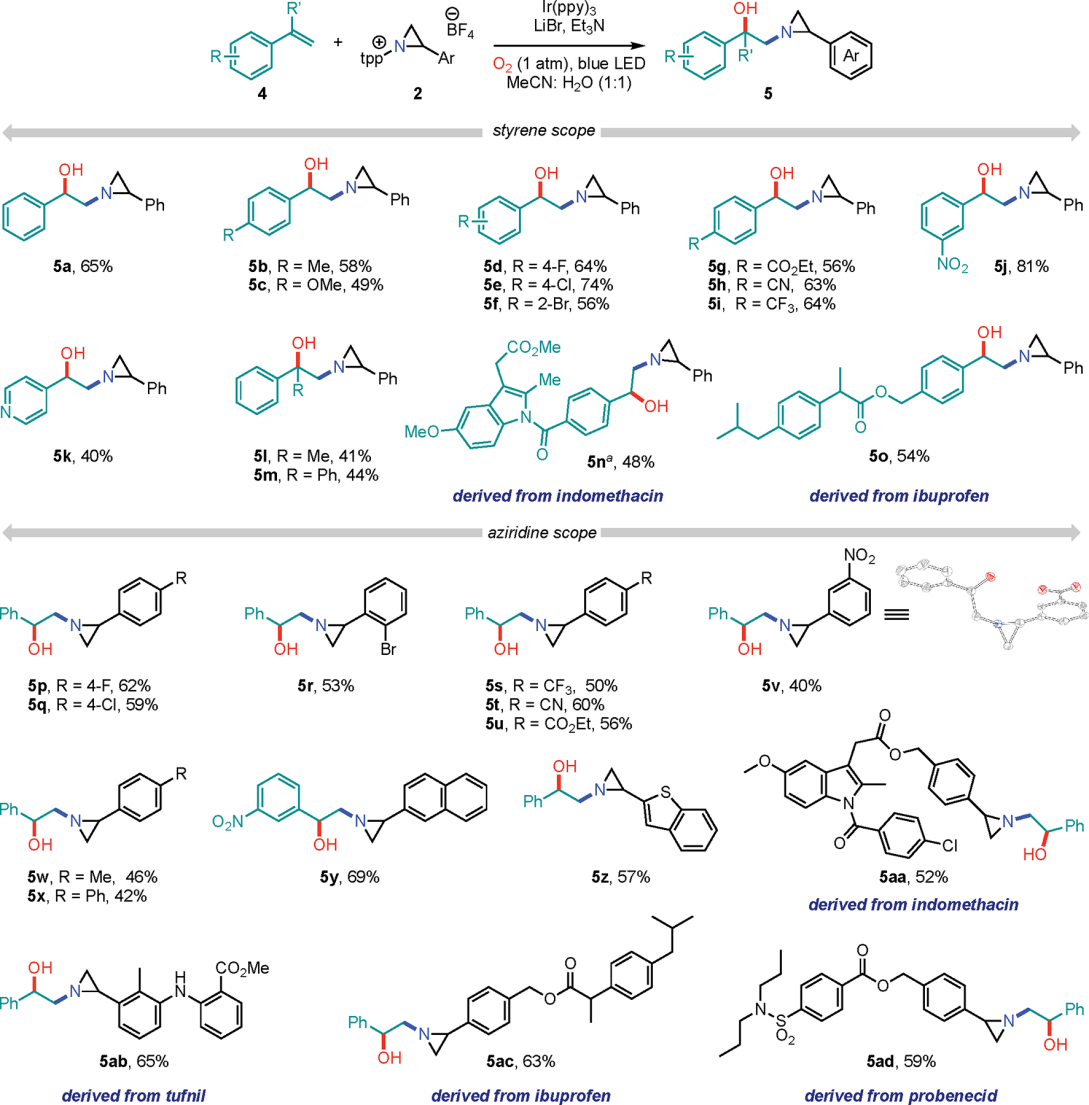
Styrene scope. Conditions: **2a** (0.1 mmol), **4a**–**4o** (0.15 mmol), Et_3_N (0.2 mmol), Ir(ppy)_3_ (1.0 mol %), LiBr (0.1 mmol), blue LED, 23 °C in MeCN:H_2_O (1:1, 2.0 mL). Aziridine scope. Conditions: **2p**–**2ad** (0.1 mmol), **4a** (0.15 mmol), Et_3_N (0.2 mmol), Ir(ppy)_3_ (1.0 mol %), LiBr (0.1 mmol), blue LED, 23 °C in MeCN:H_2_O (1:1, 2.0 mL). ^*a*^0.2 mL of CH_2_Cl_2_ was added as the olefin **4n** is sparingly soluble in MeCN. Isolated yields.

Diverse *N-*aziridinyl radical precursors (**2p**-**2ad**) are also accommodated in the hydroxyaziridination protocol. Electron-neutral (**5p**-**5r**) and electron-deficient (**5s**-**5v**) radical precursors delivered addition products in moderate to good yields. *N*-Pyridinium aziridines with electron-donating substituents, such as **2w** and **2x**, were less efficient, delivering hydoxyaziridines **5w** and **5x** in 46% and 42% yield, respectively. The less efficient coupling of electron-rich precursors is consistent with a more challenging one-electron reduction of these substrates. Photoactivation of naphthylstyrene-derived *N-*pyridinium aziridine salt **2y** in the presence of 3-nitrostyrene delivered product **5y** in 69% isolated yield. Aziridine **2z**, derived from 2-vinyl benzothiophene, is also compatible with the aziridine-transfer protocol, delivering **5z** with a 57% yield. Moreover, *N-*pyridinium aziridines derived from pharmaceutical scaffolds such as indomethacin (**2aa**), tufnil (**2ab**), ibuprofen (**2ac**), and probenecid (**2ad**) all engage in efficient aziridine transfer chemistry (52–65% yields).

Photoactivation of *N-*pyridinium aziridines derived from aliphatic olefins is less efficient than those derived from styrenes (Figure 3). Photoactivation of cyclohexene-derived *N*-pyridinium aziridine **6** in the presence of styrene (**4a**) afforded hydroxyaziridinated product **7a** in 42% yield. A small family of substituted styrene derivatives were treated with **6** under blue-light irradiation and all afforded the corresponding aziridine-transfer products (**7b-7d**, 33–48% isolated yield). Complex styrene derivatives, such as indomethacin derived **4p**, could be engaged similarly, albeit in a low yield: Product **7e** was isolated in an 18% yield. Finally, access to ethylene-derived *N*-pyridinium aziridine **8** provided the opportunity to evaluate the transfer of the simplest, completely unsubstituted *N*-aziridinyl radical (Figure 3b). 1,2-Hydroxyaziridination of styrene with an unsubstituted aziridine radical affords 2-hydroxy-2-phenyl-1-aziridinoethane (**9**, HPAE), which is currently being used as an experimental anticancer agent against neuroblastoma,^18^ in 22% yield.

**Figure 3:**
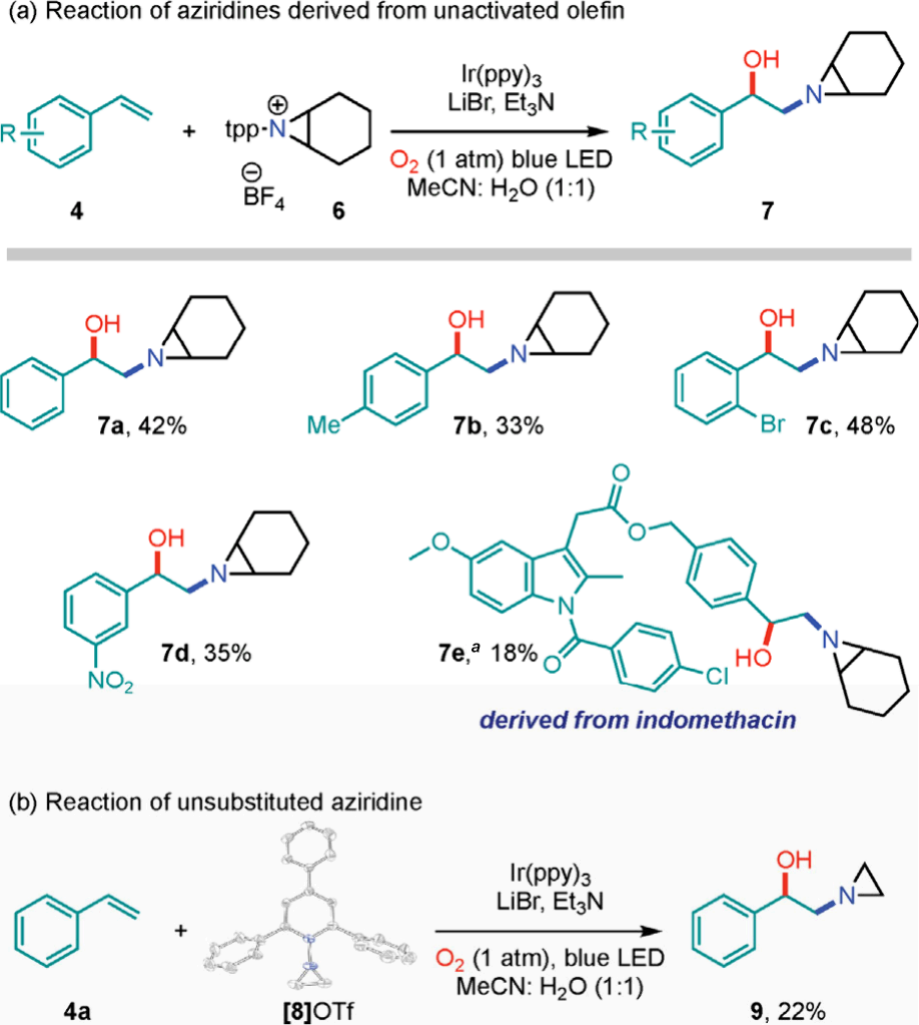
(a) Conditions: **6** (0.1 mmol), **4** (0.15 mmol), Et_3_N (0.2 mmol), Ir(ppy)_3_ (1.0 mol %), LiBr (0.1 mmol), blue LED, 23 °C in MeCN:H_2_O (1:1, 2.0 mL). ^*a*^0.2 mL of CH_2_Cl_2_ was added as the olefin **4p** was sparingly soluble in MeCN. Isolated yields. (b) Unsubstituted aziridine was engaged into a photocatalytic reaction to get hydroxyazirinated product **9** involving the simplest aziridinyl radical.

The development of hydroxyaziridination chemistry was predicated on the reductive activation of *N*-pyridinium aziridines to afford transient *N*-aziridinyl radicals. DFT optimization of the parent *N*-aziridinyl radical indicates a planar geometry with the unpaired spin in a p-orbital (Figure 4a).^14^ Under O_2_, hydroxyaziridination of **4q** afforded hydroxyaziridination product **10**; under N_2_, hydroxyaziridination of **4q** afforded cyclopropyl ring opened product **11** (Figure 4b).^19^ Further, hydroxyaziridination of **4a** in the presence of H_2_^18^O afforded **5a** without significant ^18^O incorporation, indicating that O_2_ is the source of the hydroxyl group in this reaction.^20^ Finally, the addition of TEMPO completely inhibited the hydroxyaziridination of **2a**. Together, these observations are consistent with the intermediacy of *bona fide N*-aziridinyl radical intermediates.

**Figure 4:**
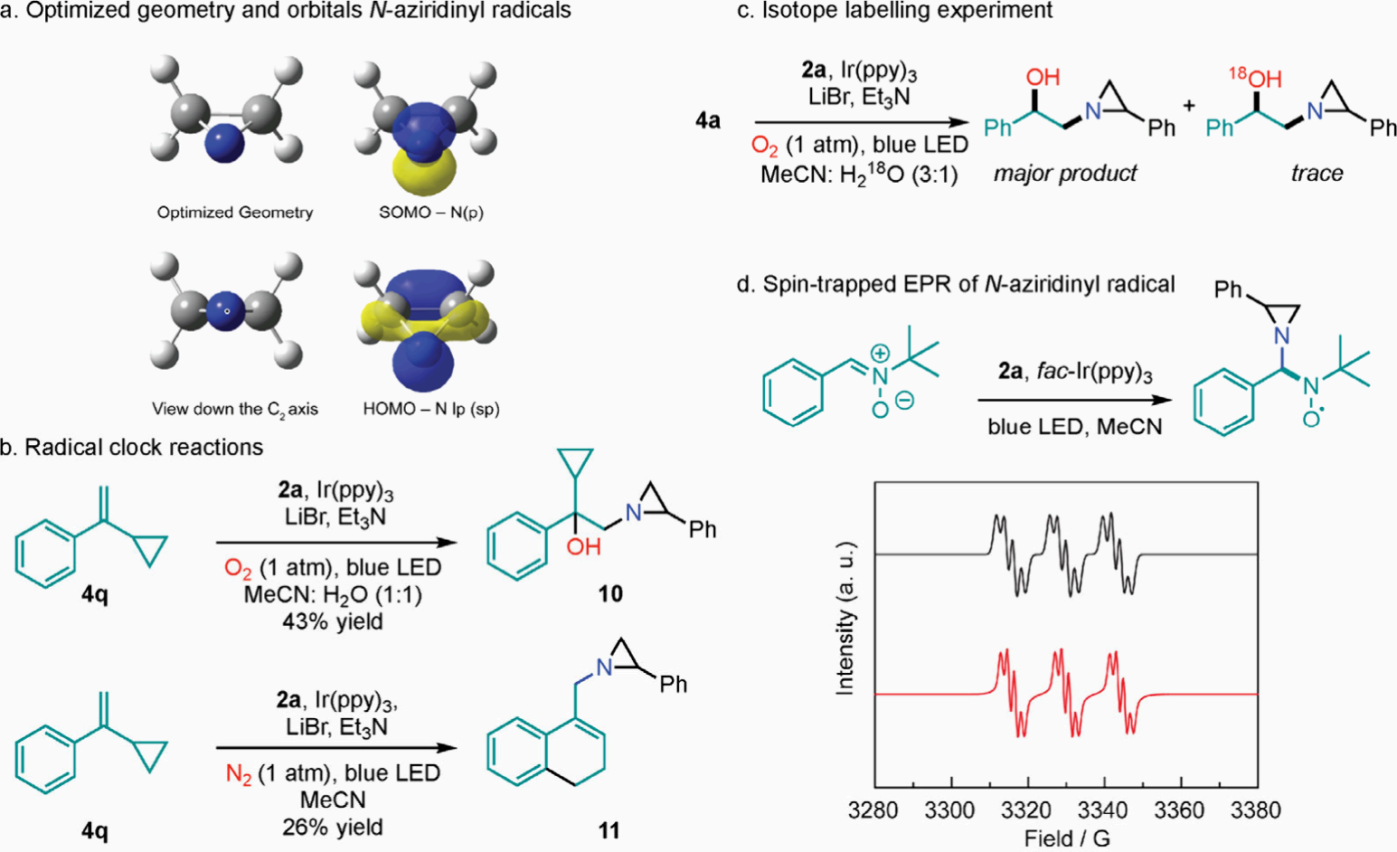
(a) Optimized geometry and SOMO of the ethylene-derived *N*-aziridinyl radical. (b) Radical clock reaction was carried out between **2a** and **4q**. (c) Isotope labeling experiment with H_2_^18^O. (d) EPR data of PBN-trapped *N-*aziridinyl radical.

To directly evaluate the intermediacy of *N*-aziridinyl radicals, we carried out the photolysis of a MeCN solution of **2a**, Ir(ppy)_3_, and Et_3_N in the presence of *N*-*tert*-butyl-α-phenylnitrone (PBN). PBN is an attractive spin trap, because unlike BHT or TEMPO, radical intermediates form kinetically persistent covalent adducts with PBN that can be characterized by a combination of electron paramagnetic resonance (EPR) spectroscopy and mass spectrometry. The EPR spectrum following photolysis of a mixture of **2a** and PBN under the conditions described above (i.e., Ir(ppy)_3_ (1.0 mol %), Et_3_N, blue LED) displayed a triplet of quartets attributed to PBN-trapped aziridnyl radical with a_N(PBN)_ = 14.0 G, a_H_ = 1.8 G, and a_N(aziridinyl)_ = 2.1 G (Figure 4d). The apparent triplet of the quartet is due to unresolved hyperfine coupling from a_H_ and a_N(aziridinyl)_. Formation of PBN-trapped aziridinyl radical was further confirmed by mass spectrometry of the EPR sample: Calculated for [M + H]^+^ = 295.1805, observed [M + H]^+^ = 295.1797.

Stern–Volmer quenching studies as a function of both [**2a**] and [Et_3_N] indicate that **2a** is the primary quencher for this reaction (Supporting Information Section D.5). As the hydroxyaziridination reaction is carried out under O_2_, one might envision that reductive quenching could also be accomplished by O_2_. To evaluate this possibility, we collected cyclic voltammetry (CVs) of **2a**. Under N_2_, compound **2a** displays an irreversible reductive event at −0.85 V vs Fc^+^/Fc; under an aerobic atmosphere, in addition to the reductive wave at −0.85 V, an O_2_ reduction wave is observed at −1.2 V vs Fc^+^/Fc. From these data, **2a** appears to be a more competent electron transfer partner than O_2_, although given the high concentration of O_2_ during olefin functionalization, the O_2_-mediated reduction of **2a** may contribute to the overall observed catalytic rates (for more details see the Supporting Information).

The available data are consistent with the mechanism illustrated in Figure 5. Single-electron transfer from the Ir(III) catalyst to **2a** can generate aziridinyl radical **I**. Examination of the Frontier orbitals of reaction between styrene and the *N-*aziridinyl radical derived from **2a** indicates energetic matching of the styrene HOMO (i.e., C=C π-bond) with the partially occupied *N-*orbital, which is consistent with electrophilic addition (Figure S2). Further, the radical polarity (ω) of *N*-aziridinyl radicals was calculated using the workflow recently introduced by Nagib et al.,^21^ to be 1.58 eV, which is consistent with a weakly electrophilic radical.^22^ Reaction of **I** with styrene generates benzylic radical **II**,^23^ which is trapped with O_2_ to afford hydroxyaziridine **5a**.

**Figure 5:**
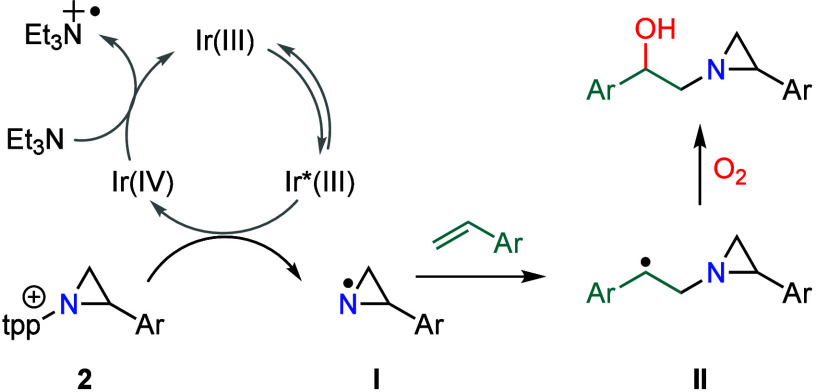
Potential photocatalytic mechanism for the *N*-aziridinyl radical generation and transfer.

In summary, *N*-aziridinyl radicals are novel reactive intermediates that enable transfer of intact aziridines to olefinic substrates. *N*-Aziridinyl radicals are generated under mild photochemical conditions, which enables the strained fragment to engage in intermolecular addition chemistry without an appreciable ring opening. Olefin 1,2-hydroxyaziridination was demonstrated with a variety of aziridine precursors, including the simplest unsubstituted fragment. Radical trapping experiments supported the formation and intermediacy of freely diffusing *N*-aziridinyl radical intermediates. Together, these results provide new disconnections for aziridines in functional organic molecules and demonstrate the accessibility of strained aminyl radical intermediates in synthesis.

## Supporting Information Available

Experimental methods, optimization data, spectral data and experimental spectra, diffraction data. (PDF)
