# Supplementary material for: Aziridine Group Transfer via Transient N-Aziridinyl Radicals
Source: J Am Chem Soc. 2024 Nov 4;146(45):30796–801. doi: 10.1021/jacs.4c14169 (PMC11565639; doi:10.1021/jacs.4c14169)
Supplement: Supplementary file 1 — ja4c14169_si_001.pdf [file ja4c14169_si_001.pdf]

Supporting Information

Aziridine Group Transfer via Transient *N*-Aziridinyl Radicals

Promita Biswas, Asim Maity, Matthew T. Figgins, and David C. Powers*

[a]*Department of Chemistry, Texas A&M University, College Station, TX 77843, USA*

Email: powers@chem.tamu.edu

Table of Contents

A. General Considerations **S3**

A.1 Materials

A.2 Characterization Details

A.3. X-Ray Diffraction Details

B. Synthesis and Characterization **S4**

B.1 Synthesis of *N*-pyridinium Aziridine **2aa**

B.2 Synthesis of *N*-pyridinium Aziridine **8**

C. Photocatalytic Olefin Hydroaziridination **S6**

C.1 General Procedure for Olefin Hydroxyaziridination

C.2 Optimization Studies for Photocatalyzed Olefin Hydroxyaziridination

D. Mechanistic Investigations **S30**

D.1. Examination of Radical Acceptors

D.2. Spin-Trapping Experiments

D.3. Radical Inhibition Experiments

D.4. Radical Clock Experiments

D.5. Stern-Volmer Quenching Experiments

D.6. Cyclic Voltammetry of *N*-Pyridinium Aziridine **2a**

D.7. Isotope Labelling Experiments

D.8. Experiments to Check the Potential Role of Oxygenated Intermediates

E. Optimized Coordinates **S40**

F. X-Ray Diffraction Data **S43**

G. NMR Spectra for New Compounds **S46**

H. References **S171**

A. General Considerations

A.1 Materials All chemicals and solvents were obtained as ACS reagent grade and used as received. Styrene was acquired from BeanTown Chemical (BTC). 4-Fluorostyrene (**4d**) and 2-bromostyrene (**4f**) were purchased from Matrix Scientific. 4-Trifluoromethylstyrene (**4g**), ibuprofen, 4-(dipropylsulfamoyl)benzoic acid (Probenecid,), and triethyl amine were acquired from Oakwood. Hexanes, ethyl acetate, dichloromethane, tetrabutylammonium iodide, 4-vinylpyridine (**4k**) and 1,1-diphenylethane (**4m**) were obtained from Sigma Aldrich. 2-((3-Chloro-2-methylphenyl)amino)benzoic acid (Tufnil), 2-(1-(4-chlorobenzoyl)-5-methoxy-2-methyl-1H-indol-3-yl)acetic acid (Indometacin) and tris(2-phenylpyridine)iridium were acquired from Ambeed. 4-Vinylbenzoic acid, and lithium bromide were acquired from TCI. 3-Nitrostyrene (**4j**) and α-methyl styrene (**4l**) were acquired from Acros. Anhydrous sodium sulfate and anhydrous potassium carbonate were obtained from VWR. Acetonitrile and methanol were obtained from Fischer Scientific. Dry dichloromethane (purchased from Fisher scientific, HPLC grade) was obtained from a drying column and stored over activated 4 Å molecular sieves.[1] NMR solvents were purchased from Cambridge Isotope Laboratories and were used as received. All reactions were carried out under ambient atmosphere unless otherwise noted. Pyridinium aziridines **2a**, **2p-2z**, **2ab-2ad**, and **6** were prepared according to literature methods.[2]

A.2 Characterization Details ^{1}H and ^{13}C NMR spectral acquisitions were recorded on an Avance Neo 500 or a Acsend™ 400 NMR and were referenced against residual proteo solvent signals: $CDCl_3$ (7.26 ppm, ^{1}H; 77.16 ppm, ^{13}C) and acetonitrile-d_3 (1.94 ppm, ^{1}H). ^{31}H NMR data are reported as follows: chemical shift (δ, ppm), (multiplicity: s (singlet), d (doublet), t (triplet), m (multiplet), br (broad), integration). ^{13}C NMR data are reported as follows: chemical shift (δ, ppm). Mass spectrometry data were recorded on either Orbitrap Fusion™ Tribrid™ Mass Spectrometer or Q Exactive™ Focus Hybrid Quadrupole-Orbitrap™ Mass Spectrometer from ThermoFisher Scientific. Fluoresence spectra and Stern-Volmer analyses were conducted using a Ocean SR miniature spectrophotometer.

A.3. X-Ray Diffraction Details Experimental details regarding sample crystallization are included in the synthetic procedures for the relevant compounds. A Bruker APEX 2 Duo X-ray (three-circle) diffractometer was used for crystal screening, unit cell determination, and data collection for the X-ray crystal structures of **5v** and **8**. Crystal suitable for X-ray diffraction were mounted on a MiTeGen dual-thickness micro-mount and placed under a cold N_2 stream (Oxford). The X-ray radiation employed was generated from a Mo sealed X-ray tube (K_α = 0.70173 Å with a potential of 40 kV and a current of 40 mA). Bruker AXS APEX II software was used for data collection and reduction. Absorption corrections were applied using the 4 program SADABS. A solution was obtained using XT/XS in APEX2 and refined in Olex2.[4] Hydrogen atoms were placed in idealized positions and were set riding on the respective parent atoms. All non-hydrogen atoms were refined with anisotropic thermal parameters. The structure was refined (weighted least squares refinement on F2) to convergence.[4c]

B. Synthesis and Characterization

B.1 Synthesis of *N*-pyridinium Aziridine 2aa

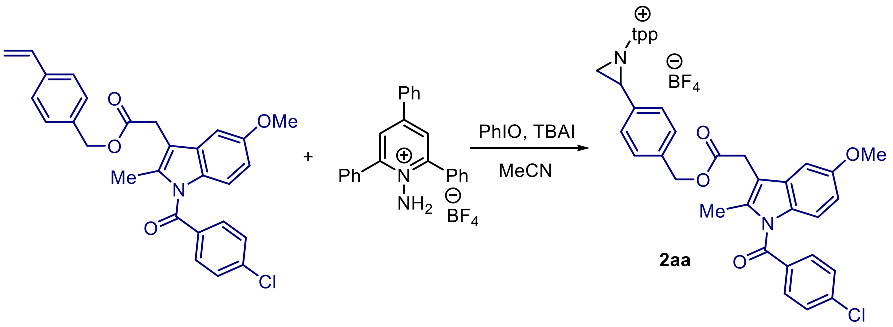

Under an N_2 atmosphere, a 100-mL round-bottom flask was charged with 1-amino-2,4,6-triphenylpyridin-1-ium tetrafluoroborate (213 mg, 0.520 mmol, 1.00 equiv) tetrabutylammonium iodide (9.6 mg, 0.026 mmol, 5.0 mol%), 4 Å molecular sieves, iodosylbenzene (116 mg, 0.520 mmol, 1.00 equiv), and 4-vinylbenzyl 2-(1-(4-chlorobenzoyl)-5-methoxy-2-methyl-1H-indol-3-yl)acetate (250 mg, 0.520 mmol, 1.00 equiv). Acetonitrile (4 mL) was added to the reaction mixture, which was then stirred at 23 °C for 12 h. Under an ambient atmosphere, the reaction mixture was filtered through a pad of Celite and concentrated *in vacuo*. The residue was purified by silica gel flash chromatography (1:1 ethyl acetate:hexanes) to afford the compound **2aa** as off white solid (254 mg, 44% yield). ^{1}H NMR (400 MHz, CD_3CN) δ 8.18 (s, 2H), 8.11–8.00 (m, 2H), 8.01–7.79 (m, 5H), 7.75–7.61 (m, 3H), 7.57 (d, J = 8.6 Hz, 2H), 7.48 (d, J = 33.5 Hz, 7H), 7.17–6.83 (m, 4H), 6.66–6.48 (m, 3H), 5.08 (s, 2H), 3.81 (s, 3H), 3.47 (dd, J = 8.3, 5.7 Hz, 1H), 2.68 (dd, J = 8.4, 3.1 Hz, 1H), 2.40 (dd, J = 5.8, 3.1 Hz, 1H), 2.31 (s, 2H), 2.18 (s, 3H). ^{13}C NMR (101 MHz, CD_3CN) δ 171.2, 168.9, 156.7, 153.9, 153.8, 139.1, 137.3, 136.6, 135.0, 134.6, 134.3, 132.6, 132.2, 132.2, 131.8, 131.4, 131.4, 130.5, 130.3, 129.7, 129.7, 129.4, 128.8, 128.0, 126.8, 126.2, 115.7, 113.4, 112.1, 102.0, 66.4, 60.6, 55.9, 54.0, 30.3, 14.2, 13.4. HRMS-ESI: calculated for $C_{51}H_{41}ClN_3O_4$ [M^+] = 794.2780, observed [M^+] = 794.2764.

B.2 Synthesis of *N*-pyridinium Aziridine 8

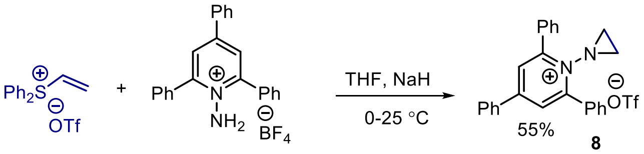

A 100-mL Schlenk tube was charged with NaH (92.0 mg, 2.30 mmol, 1.00 equiv), which was washed with pentane. Dry THF (6.0 mL) was added to the reaction vessel and the reaction mixture was cooled to 0 °C. 1-Amino-2,4,6-triphenylpyridin-1-ium tetrafluoroborate (943 mg, 2.30 mmol, 1.00 equiv) was added to the reaction as a solid and the reaction mixture was stirred at 0 °C for 15 min. A THF solution (6.0 mL) of diphenyl(vinyl)sulfonium trifluoromethanesulfonate[5] (1.00 g, 2.76 mmol, 1.20 equiv) was added dropwise. The reaction mixture was allowed to warm to 23 °C at which temperature it was stirred for 12 h. Solids were removed from the reaction mixture by filtration and were washed with EtOAc. The combined filtrate was concentrated under reduced pressure and the residue was purified using silica gel column chromatography with 50% EtOAc:Hexane solution. The product was obtained as white solid (274 mg, 55% yield). ^{1}H NMR (400 MHz, CD_3CN): δ 8.17 (s, 2H), 8.02 (m, 6H), 7.74 (dd, J = 5.2, 1.9 Hz, 6H), 7.69–7.60 (m, 3H), 2.27–2.11 (m, 4H). ^{13}C NMR (101 MHz, CD_3CN) δ 154.2, 153.9, 134.9, 132.8, 132.7, 132.5, 130.7, 130.5, 130.1, 129.0, 127.0, 41.0. ^{19}F NMR (376 MHz, CD_3CN): –79.3. HRMS-ESI: calculated for $C_{25}H_{21}N_2$ $[M^+]$ = 349.1699, observed $[M^+]$ = 349.1699.

C. Photocatalytic Olefin Hydroaziridination

C.1 General Procedure for Olefin Hydroxyaziridination

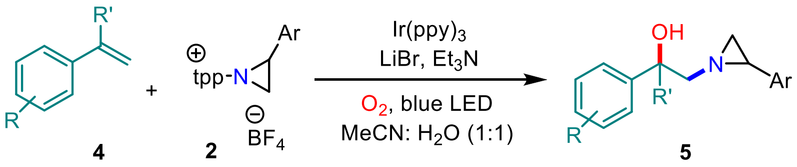

A 40-mL scintillation vial was charged with triphenylpyridinium aziridine **2** (51.0 mg, 0.100 mmol, 1.00 equiv), Ir(ppy)$_3$ (0.7 mg, 0.001 mmol, 1 mol%), and LiBr (8.70 mg, 0.100 mmol, 1.00 equiv). The solid compounds were dissolved in a 1:1 mixture of MeCN:H_2O (2 mL total). Triethylamine (28.0 µL, 0.200 mmol, 2.00 equiv) and the appropriate styrene derivative (**4**, 0.150 mmol, 1.50 equiv) were added. The reaction mixture was purged with oxygen (1 atmosphere). With stirring, the reaction was irradiated with blue LED lights for 12 h; the temperature was maintained at 23 °C using air cooling provided by a fan. The reaction mixture was diluted with distilled water (5 mL) and extracted with ethyl acetate (3 × 5 mL). The combined organic layers were washed with brine, dried over anhydrous Na_2SO_4, and concentrated under reduced pressure. The crude product was purified by column chromatography (using a Hexane:EtOAc gradient, 4:1 → 1:100) to afford the corresponding hydroxyaziridination product **5**.

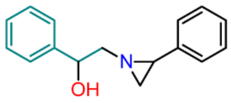

5a

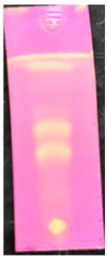

1-Phenyl-2-(2-phenylaziridin-1-yl)ethan-1-ol (5a) Prepared from styrene (**4a**, 17.0 µL, 0.150 mmol, 1.50 equiv) and 2,4,6-triphenyl-1-(2-phenylaziridin-1-yl)pyridin-1-ium tetrafluoroborate (**2a**, 51.0 mg, 0.100 mmol, 1.00 equiv), and obtained as a 1:1 mixture of two diastereomers as a pale-yellow liquid (15.5 mg, 65% yield). ^{1}H NMR (400 MHz, $CDCl_3$) δ 7.53–7.16 (m, 10H), 4.91 (dd, *J* = 9.0, 3.5 Hz, 1H), 3.36 (br, 1H), 2.88 (dd, *J* = 12.0, 8.9 Hz, 1H), 2.66–2.31 (m, 2H), 1.96 (d, *J* = 3.5 Hz, 1H), 1.78 (d, *J* = 6.5 Hz, 1H). ^{1}H NMR (400 MHz, $CDCl_3$) δ 7.60–7.11 (m, 10H), 4.88 (dd, *J* = 9.1, 3.4 Hz, 1H), 3.36 (br, 1H), 2.95 (dd, *J* = 12.0, 9.1 Hz, 1H), 2.71–2.19 (m, 2H), 2.00 (d, *J* = 3.4 Hz, 1H), 1.84 (d, *J* = 6.5 Hz, 1H). ^{13}C NMR (101 MHz, $CDCl_3$) δ 141.7, 139.6, 128.3, 128.2, 127.5, 127.0, 126.0, 125.9, 72.9, 68.5, 41.2, 37.4. ^{13}C NMR (101 MHz, $CDCl_3$) δ 141.7, 139.7, 128.3, 127.6, 127.0, 126.0, 125.9, 73.1, 68.5, 41.1, 38.0. HRMS-ESI$^+$: calculated for $C_{16}H_{18}NO$ [M+H$^+$] = 240.1383, observed [M+H$^+$] = 240.1378.[i]

[i] Analysis of the mass spectrometry data revealved the formation of depyridylated N–H aziridine and benzyl peroxide byproducts. The peroxide was formed in trace amounts based on signal intensity.

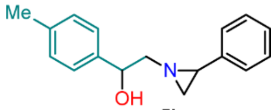

5b

2-(2-Phenylaziridin-1-yl)-1-(p-tolyl)ethan-1-ol (5b) Prepared from 4-methylstyrene (**4b**, 20.0 µL, 0.150 mmol, 1.50 equiv) and 2,4,6-triphenyl-1-(2-phenylaziridin-1-yl)pyridin-1-ium tetrafluoroborate (**2a**, 51.0 mg, 0.100 mmol, 1.00 equiv), and obtained as a 1:1 mixture of two diastereomers as a pale-yellow liquid (14.6 mg, 58% yield). ^{1}H NMR (400 MHz, $CDCl_3$): δ 7.30 (dd, *J* = 7.1, 1.2 Hz, 2H), 7.28–7.19 (m, 5H), 7.13 (d, *J* = 7.9 Hz, 2H), 4.87 (dd, *J* = 9.2, 3.4 Hz, 1H), 3.30 (br, 1H), 2.87 (dd, *J* = 11.9, 9.1 Hz, 1H), 2.50 (dd, *J* = 6.6, 3.4 Hz, 1H), 2.47–2.41 (m, 1H), 2.33 (s, 3H), 1.94 (d, *J* = 3.4 Hz, 1H), 1.77 (d, *J* = 6.5 Hz, 1H).^{1}H NMR (400 MHz, $CDCl_3$): δ 7.38–7.27 (m, 2H), 7.28–7.19 (m, 5H), 7.13 (d, *J* = 7.9 Hz, 2H), 4.87 (dd, *J* = 9.1, 3.5 Hz, 1H), 3.29 (br, 1H), 2.87 (dd, *J* = 11.9, 9.1 Hz, 1H), 2.50 (dd, *J* = 6.6, 3.5 Hz, 1H), 2.45 (dd, *J* = 12.0, 3.3 Hz, 1H), 2.34 (s, 3H), 1.94 (d, *J* = 3.4 Hz, 1H), 1.77 (d, *J* = 6.5 Hz, 1H). ^{13}C NMR (101 MHz, $CDCl_3$): δ 139.7, 138.7, 137.3, 129.0, 128. 3, 127.0, 126.0, 125.9, 73.0, 68.6, 41.1, 38.0, 21.1. ^{13}C NMR (101 MHz, $CDCl_3$): δ 139.7, 138.8, 137.2, 129.0, 128.2, 127.0, 126.1, 125.8, 72.8, 68.6, 41.2, 37.4, 21.1. HRMS-ESI^{+}: calculated for $C_{17}H_{20}NO$ [M+H^{+}] = 254.1539, observed [M+H^{+}] = 254.1531.

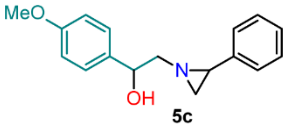

5c

1-(4-Methoxyphenyl)-2-(2-phenylaziridin-1-yl)ethan-1-ol (5c) Prepared from 4-methoxystyrene (**4c**, 20.0 µL, 0.150 mmol, 1.50 equiv) and 2,4,6-triphenyl-1-(2-phenylaziridin-1-yl)pyridine-1-ium tetrafluoroborate (**2a**, 51.0 mg, 0.100 mmol, 1.00 equiv), and obtained as a 1:1 mixture of two diastereomers as a pale-yellow liquid (13.2 mg, 49% yield). ^{1}H NMR (400 MHz, $CDCl_3$) δ 7.42–7.08 (m, 14H), 6.94–6.56 (m, 4H), 4.76 (m, 2H), 3.72 (s, 6H), 2.83 (m, 2H), 2.67–2.18 (m, 4H), 1.89 (dt, *J*=24.2, 3.1 Hz, 2H), 1.81–1.56 (m, 2H). ^{13}C NMR (101 MHz, $CDCl_3$): δ 159.1, 159.0, 139.8, 139.7, 134.0, 133.9, 128.3, 128.2, 127.2, 127.1, 127.0, 126.1, 126.0, 113.9, 113.7, 72.8, 72.5, 68.6, 68.5, 55.2, 41.2, 41.2, 38.0, 37.4. HRMS-ESI^{+}: calculated for $C_{17}H_{20}NO_2$ [M+H^{+}] = 270.1489, observed [M+H^{+}] = 270.1484.

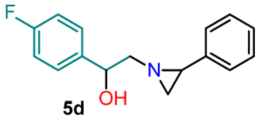

5d

1-(4-Fluorophenyl)-2-(2-phenylaziridin-1-yl)ethan-1-ol (5d) Prepared from 4-fluorostyrene (**4d**, 18.0 µL, 0.150 mmol, 1.50 equiv) and 2,4,6-triphenyl-1-(2-phenylaziridin-1-yl)pyridin-1-

ium tetrafluoroborate (**2a**, 51.0 mg, 0.100 mmol, 1.00 equiv), and obtained as a 1:1 mixture of two diastereomers as a yellow liquid (16.4 mg, 64% yield). ^{1}H NMR (400 MHz, $CDCl_3$): δ 7.31 (m, 4H), 7.28–7.18 (m, 3H), 6.99 (t, *J* = 8.7 Hz, 2H), 4.88 (dd, *J* = 8.8, 3.6 Hz, 1H), 3.41 (br, 1H), 2.84 (dd, *J* = 11.9, 8.8 Hz, 1H), 2.62–2.30 (m, 2H), 1.95 (d, *J* = 3.4 Hz, 1H), 1.77 (d, *J* = 6.5 Hz, 1H).^{1}H NMR (400 MHz, $CDCl_3$): 7.38–7.28 (m, 4H), 7.25–7.22 (m, 3H), 7.01 (t, *J* = 8.7 Hz, 2H), 4.85 (dd, *J* = 9.1, 3.5 Hz, 1H), 3.34 (br, 1H), 2.93 (dd, *J* = 12.0, 9.0 Hz, 1H), 2.42 (dd, *J* = 6.6, 3.4 Hz, 1H), 2.36 (dd, *J* = 12.0, 3.6 Hz, 1H), 2.00 (d, *J* = 3.4 Hz, 1H), 1.84 (d, *J* = 6.5 Hz, 1H).^{13}C NMR (125 MHz, $CDCl_3$): δ 162.2 (d, *J* = 245.0 Hz), 139.5, 137.5, 128.3, 127.5 (d, *J* = 8.7 Hz) , 127.1, 126.0, 115.1 (d, *J* = 20.0 Hz), 72.2, 68.4, 41.2, 37.4. ^{13}C NMR (101 MHz, $CDCl_3$): δ 162.2 (d, *J* = 195.0 Hz), 139.6, 137.5 (d, *J* = 3.0 Hz), 128.3, 127.6 (d, *J* = 7.0 Hz), 127.1, 126.0, 115.1 (d, *J* = 17.0 Hz), 72.5, 68.5, 41.2, 38.0. HRMS-ESI$^+$: calculated for $C_{16}H_{17}FNO$ [M+H$^+$] = 258.1289, observed [M+H$^+$] = 258.1279.

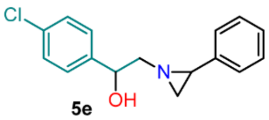

5e

1-(4-Chlorophenyl)-2-(2-phenylaziridin-1-yl)ethan-1-ol (5e) Prepared from 4-chlorostyrene (**4e**, 19.0 µL, 0.150 mmol, 1.50 equiv,) and 2,4,6-triphenyl-1-(2-phenylaziridin-1-yl)pyridin-1-ium tetrafluoroborate (**2a**, 51.0 mg, 0.100 mmol, 1.00 equiv), and obtained as a 1:1 mixture of two diastereomers as a yellow liquid (20.2 mg, 74% yield). ^{1}H NMR (400 MHz, $CDCl_3$): δ 7.40–7.28 (m, 2H), 7.28–7.22 (m, 5H), 7.22–7.15 (m, 2H), 4.87 (dd, *J* = 8.7, 3.6 Hz, 1H), 3.41 (br, 1H), 2.81 (dd, *J* = 11.9, 8.7 Hz, 1H), 2.50 (dd, *J* = 11.9, 3.6 Hz, 1H), 2.45 (dd, *J* = 6.6, 3.5 Hz, 1H), 1.96 (d, *J* = 3.4 Hz, 1H), 1.77 (d, *J* = 6.6 Hz, 1H). ^{1}H NMR (400 MHz, $CDCl_3$): δ 7.34–7.19 (m, 9H), 4.85 (dd, *J* = 8.9, 3.6 Hz, 1H), 2.91 (dd, *J* = 12.0, 8.9 Hz, 1H), 2.44–2.33 (m, 2H), 2.00 (d, *J* = 3.4 Hz, 1H), 1.83 (d, *J* = 6.5 Hz, 1H). ^{13}CNMR (101 MHz, $CDCl_3$): δ 140.3, 139.4, 133.2, 128.4, 128.3, 127.3, 127.1, 126.0, 72.2, 68.3, 41.3, 37.4. ^{13}C NMR (101 MHz, $CDCl_3$): δ 140.2, 139.5, 133.2, 128.4, 128.3, 127.3, 127.1, 125.9, 72.4, 68.3, 41.2, 38.0. HRMS-ESI$^+$: calculated for $C_{16}H_{17}ClNO$ [M+H$^+$] = 274.0993, observed [M+H$^+$] = 274.0987.

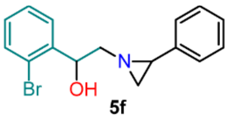

5f

1-(2-Bromophenyl)-2-(2-phenylaziridin-1-yl)ethan-1-ol (5f) Prepared from 2-bromostyrene (**4f**, 19.0 µL, 0.150 mmol, 1.50 equiv) and 2,4,6-triphenyl-1-(2-phenylaziridin-1-yl)pyridin-1-ium tetrafluoroborate (**2a**, 51.0 mg, 0.100 mmol, 1.00 equiv), and obtained as a 1:1 mixture of two diastereomers as a yellow liquid (17.8 mg, 56% yield). ^{1}H NMR (400 MHz, $CDCl_3$): δ 7.54 (m, 2H), 7.41 (dd, *J* = 7.9, 1.2 Hz, 2H), 7.31–7.01 (m, 14H), 5.13 (td, *J* = 8.8, 2.9 Hz, 2H), 2.73 (m, 2H), 2.51 (dt, *J* = 8.8, 2.7 Hz, 2H), 2.45–2.30 (m, 2H), 1.91 (dd, *J* = 8.7, 3.4 Hz, 2H), 1.82 (d, *J* = 6.5 Hz, 1H), 1.72 (d, *J* = 6.5 Hz, 1H). ^{13}C NMR (101 MHz, $CDCl_3$): δ 140.5, 140.4,

139.7, 139.5, 132.4, 132.4, 128.9, 128.8, 128.3, 128.2, 127.8, 127.7, 127.6, 127.1, 127.0, 126.1, 126.0, 121.6, 121.4, 72.0, 71.6, 66.4, 66.1, 41.4, 40.9, 38.0, 37.2. HRMS-ESI$^+$: calculated for $C_{16}H_{17}BrNO$ [M+H$^+$] = 318.0488, observed [M+H$^+$] = 318.0482.

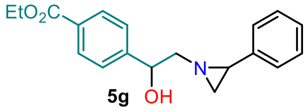

5g

Ethyl 4-(1-hydroxy-2-(2-phenylaziridin-1-yl)ethyl)benzoate (5g) Prepared from ethyl 4-vinylbenzoate (**4g**, 26.0 mg, 0.150 mmol, 1.50 equiv) and 2,4,6-triphenyl-1-(2-phenylaziridin-1-yl)pyridin-1-ium tetrafluoroborate (**2a**, 51.0 mg, 0.100 mmol, 1.00 equiv), and obtained as a 1:1 mixture of two diastereomers as a yellow liquid (17.4 mg, 56% yield). ^{1}H NMR (400 MHz, $CDCl_3$): δ 7.98 (d, J = 8.4 Hz, 2H), 7.41 (d, J = 8.3 Hz, 2H), 7.34–7.21 (m, 3H), 7.20–7.11 (m, 2H), 4.95 (dd, J = 8.7, 3.6 Hz, 1H), 4.37 (q, J = 7.1 Hz, 2H), 3.48 (br, 1H), 2.83 (dd, J = 11.9, 8.6 Hz, 1H), 2.54 (dd, J = 11.9, 3.6 Hz, 1H), 2.45 (dd, J = 6.6, 3.5 Hz, 1H), 1.96 (d, J = 3.4 Hz, 1H), 1.79 (d, J = 6.6 Hz, 1H), 1.39 (t, J = 7.1 Hz, 3H). ^{1}H NMR (400 MHz, $CDCl_3$): δ 8.01 (d, J = 8.4 Hz, 1H), 7.65–7.37 (m, 1H), 7.37–6.88 (m, 7H), 4.93 (dd, J = 8.9, 3.4 Hz, 1H), 4.37 (q, J = 7.1 Hz, 2H), 3.47 (br, s), 2.90 (dd, J = 11.9, 8.9 Hz, 1H), 2.46 (dt, J = 9.7, 3.5 Hz, 2H), 1.99 (d, J = 3.3 Hz, 1H), 1.82 (d, J = 6.6 Hz, 1H), 1.39 (t, J = 7.2 Hz, 3H). ^{13}C NMR (100 MHz, $CDCl_3$): δ 166.4, 146.8, 139.4, 129.7, 129.6, 128.3, 127.1, 126.0, 125.8, 72.4, 68.2, 60.9, 41.4, 37.4, 14.3. ^{13}C NMR (100 MHz, $CDCl_3$): δ 166.4, 146.8, 139.5, 129.7, 129.6, 128.3, 127.1, 125.9, 125.8, 72.7, 68.2, 60.9, 41.1, 38.1, 14.3. HRMS-ESI$^+$: calculated for $C_{19}H_{22}NO_3$ [M+H$^+$] = 312.1594, observed [M+H$^+$] = 312.1582.

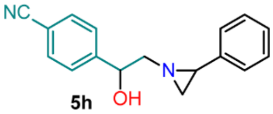

5h

4-(1-Hydroxy-2-(2-phenylaziridin-1-yl)ethyl)benzonitrile (5h) Prepared from 1-(trifluoromethyl)-4-vinylbenzene (**4h**, 19.0 mg, 0.150 mmol, 1.50 equiv) and 2,4,6-triphenyl-1-(2-phenylaziridin-1-yl)pyridin-1-ium tetrafluoroborate (**2a**, 51.0 mg, 0.100 mmol, 1.00 equiv), and obtained as a 1:1 mixture of two diastereomers as a yellow liquid (16.6 mg, 63% yield). ^{1}H NMR (400 MHz, $CDCl_3$): δ 7.49 (dd, J = 8.3, 1.8 Hz, 2H), 7.45–7.31 (m, 2H), 7.29–7.12 (m, 3H), 7.13–6.96 (m, 2H), 4.86 (dd, J = 8.2, 3.7 Hz, 1H), 3.52 (br, 1H), 2.68 (dd, J = 11.6, 8.5 Hz, 1H), 2.55 (dd, J = 11.9, 2.6 Hz, 1H), 2.31 (dd, J = 6.7, 3.4 Hz, 1H), 2.05–1.83 (m, 1H), 1.72 (dd, J = 6.6, 1.6 Hz, 1H).^{1}H NMR (400 MHz, $CDCl_3$): δ 7.61 (d, J = 8.4 Hz, 2H), 7.54–7.46 (m, 2H), 7.35–7.27 (m, 3H), 7.27–7.16 (m, 2H), 4.92 (dd, J = 8.7, 3.8 Hz, 1H), 3.53 (br, 1H), 2.90 (dd, J = 12.0, 8.7 Hz, 1H), 2.57–2.22 (m, 2H), 1.99 (d, J = 3.4 Hz, 1H), 1.82 (d, J = 6.6 Hz, 1H). ^{13}C NMR (100 MHz, $CDCl_3$): δ 147.1, 139.3, 132.1, 128.4, 127.2, 126.6, 125.9, 118.8, 111.3, 72.4, 67.9, 41.3, 38.0. ^{13}C NMR (100 MHz, $CDCl_3$): δ 147.1, 139.3, 132.1, 128.4, 127.2, 126.6, 125.9, 118.8, 111.3, 72.4, 67.9, 41.3, 38.0. HRMS-ESI$^+$: calculated for $C_{17}H_{17}N_2O$ [M+H$^+$] = 265.1335, observed [M+H$^+$] = 265.1331.

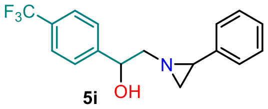

5i

2-(2-Phenylaziridin-1-yl)-1-(4-(trifluoromethyl)phenyl)ethan-1-ol (5i) Prepared from 1-(trifluoromethyl)-4-vinylbenzene (**4i**, 22.0 µL, 0.150 mmol, 1.50 equiv) and 2,4,6-triphenyl-1-(2-phenylaziridin-1-yl)pyridin-1-ium tetrafluoroborate (**2a**, 51.0 mg, 0.100 mmol, 1.00 equiv), and obtained as a 1:1 mixture of two diastereomers as a yellow liquid (19.6 mg, 64% yield). ^{1}H NMR (400 MHz, $CDCl_3$) δ 7.55 (d, J = 8.1 Hz, 2H), 7.45 (d, J = 8.1 Hz, 2H), 7.35–7.22 (m, 3H), 7.23–6.92 (m, 2H), 4.95 (dd, J = 8.4, 3.6 Hz, 1H), 3.51 (br, 1H), 2.79 (dd, J = 11.9, 8.3 Hz, 1H), 2.61 (dd, J = 12.0, 3.6 Hz, 1H), 2.40 (dd, J = 6.5, 3.5 Hz, 1H), 1.97 (d, J = 3.5 Hz, 1H), 1.80 (d, J = 6.5 Hz, 1H). ^{1}H NMR (400 MHz, $CDCl_3$) δ 7.58 (d, J = 8.1 Hz, 2H), 7.49 (d, J = 8.1 Hz, 2H), 7.39–7.26 (m, 2H), 7.25–7.09 (m, 3H), 4.93 (dd, J = 8.8, 3.7 Hz, 1H), 3.45 (br, 1H), 2.93 (dd, J = 12.0, 8.9 Hz, 1H), 2.48–2.33 (m, 2H), 2.00 (d, J = 3.4 Hz, 1H), 1.83 (d, J = 6.6 Hz, 1H). ^{13}C NMR (101 MHz, $CDCl_3$) δ 145.9, 139.3, 135.9, 129.8 (q, J = 33.0 Hz), 128.3, 127.2 (q, J = 270.0 Hz), 126.2, 126.0, 125.2 (q, J = 4.0 Hz), 72.2, 68.2, 41.5, 37.3. ^{13}C NMR (101 MHz, $CDCl_3$) δ 145.7, 139.4, 129.9 (q, J = 32.8 Hz),, 128.3, 127.1 (q, J = 272.0 Hz), 126.2, 125.4 (q, J = 3.8 Hz), 72.5, 68.2, 41.3, 38.0. HRMS-ESI^{+}: calculated for $C_{17}H_{17}F_3NO$ [M+H^{+}] = 308.1257, observed [M+H^{+}] = 308.1250.

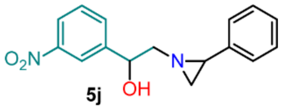

5j

1-(3-Nitrophenyl)-2-(2-phenylaziridin-1-yl)ethan-1-ol (5j) Prepared from 1-nitro-4-vinylbenzene (**4j**, 19.0 µL, 0.150 mmol, 1.50 equiv) and 2,4,6-triphenyl-1-(2-phenylaziridin-1-yl)pyridin-1-ium tetrafluoroborate (**2a**, 51.0 mg, 0.100 mmol, 1.00 equiv), and obtained as a 1:1 mixture of two diastereomers as a yellow liquid (23.0 mg, 81% yield). ^{1}H NMR (400 MHz, $CDCl_3$): δ 8.24–8.10 (m, 2H), 8.01 (dd, J = 7.3, 2.3 Hz, 2H), 7.61 (dd, J = 21.3, 7.7 Hz, 2H), 7.39 (dt, J = 12.5, 7.9 Hz, 2H), 7.32–7.02 (m, 10H), 4.90 (td, J = 7.7, 3.7 Hz, 2H), 2.87 (dd, J = 12.0, 8.6 Hz, 1H), 2.71 (dd, J = 12.0, 7.9 Hz, 1H), 2.59 (dd, J = 12.0, 3.8 Hz, 1H), 2.50–2.26 (m, 3H), 1.91 (dd, J = 9.8, 3.4 Hz, 2H), 1.74 (dd, J = 9.7, 6.6 Hz, 2H). ^{13}C NMR (126 MHz, $CDCl_3$): δ 148.2, 148.1, 144.1, 144.0, 139.2, 139.1, 132.1, 131.9, 129.2, 129.1, 128.3, 128.3, 127.2, 127.2, 125.9, 125.9, 122.5, 122.4, 121.1, 121.0, 72.1, 71.7, 68.0, 67.9, 41.6, 41.3, 37.9, 37.2. HRMS-ESI^{+}: calculated for $C_{16}H_{17}N_2O_3$ [M+H^{+}] = 285.1234, observed [M+H^{+}] = 285.1228.

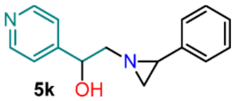

5k

2-(2-Phenylaziridin-1-yl)-1-(pyridin-2-yl)ethan-1-ol (5k) Prepared from 4-vinylpyridine (**4k**, 16.0 µL, 0.150 mmol, 1.50 equiv.) and 2,4,6-triphenyl-1-(2-phenylaziridin-1-yl)pyridin-1-ium tetrafluoroborate (**2a**, 51.0 mg, 0.100 mmol, 1.00 equiv), and obtained as a 1:1 mixture of two diastereomers as a yellow liquid (10.0 mg, 40% yield). ^{1}H NMR (400 MHz, $CDCl_3$): ^{1}H NMR (400 MHz, CD_3CN): δ 8.50–8.27 (m, 4H), 7.41–7.13 (m, 9H), 4.85–4.80 (m, 5H), 3.83 (br, 2H), 2.75 (dd, J = 12.1, 7.4 Hz, 1H), 2.66 (d, J = 4.0 Hz, 2H), 2.57 (dd, J = 12.1, 4.8 Hz, 1H), 2.48 (dd, J = 6.5, 3.4 Hz, 1H), 2.44 (dd, J = 6.5, 3.4 Hz, 1H), 1.86 (d, J = 3.4 Hz, 1H), 1.82–1.73 (m, 3H). ^{13}C NMR (101 MHz, $CDCN_3$): δ 152.9, 152.8, 150.0, 141.2, 141.2, 128.8, 127.4, 126.7, 126.7, 121.9, 121.9, 72.4, 72.1, 68.0, 68.0, 41.6, 41.1, 38.2, 37.5. HRMS-ESI$^+$: calculated for $C_{15}H_{17}N_2O$ [M+H$^+$] = 241.1335, observed [M+H$^+$] = 241.1329.

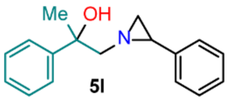

5l

2-Phenyl-1-(2-phenylaziridin-1-yl)propan-2-ol (5l) Prepared from α-methyl styrene (**4l**, 19.0 µL, 0.150 mmol, 1.50 equiv) and 2,4,6-triphenyl-1-(2-phenylaziridin-1-yl)pyridin-1-ium tetrafluoroborate (**2a**, 51.0 mg, 0.100 mmol, 1.00 equiv), and obtained as a 1.4:1 mixture of two diastereomers as a pale yellow liquid (10.5 mg, 41% yield). ^{1}H NMR (400 MHz, $CDCl_3$): δ 7.65–7.41 (m, 2H), 7.39–7.27 (m, 6H), 7.25–6.99 (m, 7H), 7.01–6.74 (m, 1H), 3.80 (br, 1H), 3.75 (br, 1H), 2.96 (d, J = 11.8 Hz, 1H), 2.76 (d, J = 2.0 Hz, 2H), 2.58 (d, J = 11.8 Hz, 1H), 2.45 (dd, J = 6.5, 3.4 Hz, 1H), 2.10 (dd, J = 6.5, 3.5 Hz, 1H), 1.95 (d, J = 3.4 Hz, 1H), 1.78 (d, J = 6.5 Hz, 1H), 1.72 (d, J = 3.5 Hz, 1H), 1.68–1.30 (m, 7H). ^{13}C NMR (126 MHz, $CDCl_3$): δ 146.6, 146.5, 139.7, 139.5, 128.3, 128.0, 127.9, 127.9, 127.0, 126.8, 126.6, 126.5, 125.9, 125.9, 124.9, 124.9, 73.7, 73.6, 72.0, 71.8, 41.7, 41.4, 38.1, 37.2, 27.8, 27.7. HRMS-ESI$^+$: calculated for $C_{17}H_{20}NO$ [M+H$^+$] = 254.1539, observed [M+H$^+$] = 254.1533.

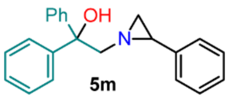

5m

1,1-Diphenyl-2-(2-phenylaziridin-1-yl)ethan-1-ol (5m) Prepared from ethene-1,1-diyldibenzene (**4m**, 27.0 µL, 0.150 mmol, 1.50 equiv) and 2,4,6-triphenyl-1-(2-phenylaziridin-1-yl)pyridin-1-ium tetrafluoroborate (**2a**, 51.0 mg, 0.100 mmol, 1.00 equiv), and obtained as a 1:1 mixture of two diastereomers as an off white solid (13.8 mg, 44% yield). ^{1}H NMR (400 MHz, $CDCl_3$): δ 7.58–7.44 (m, 2H), 7.42–7.37 (m, 2H), 7.35–7.30 (m, 2H), 7.26–7.12 (m, 7H), 7.01–6.91 (m, 2H), 4.52 (s, 1H), 3.37 (d, J = 11.8 Hz, 1H), 3.26 (d, J = 11.7 Hz, 1H), 2.30 (dd, J = 6.5, 3.5 Hz, 1H), 1.83 (d, J = 3.5 Hz, 1H), 1.72 (d, J = 6.5 Hz, 1H). ^{13}C NMR (101 MHz, $CDCl_3$): δ 145.6, 145.5, 139.4, 128.0, 128.0, 127.9, 126.9, 126.8, 126.7, 126.1, 126.1, 125.9, 77.2, 69.8, 41.6, 37.9. HRMS-ESI$^+$: calculated for $C_{22}H_{22}NO$ [M+H$^+$] = 316.1696, observed [M+H$^+$] = 316.1690.

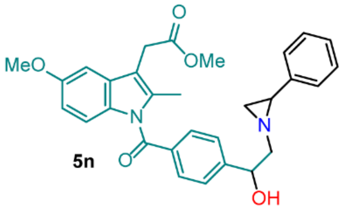

5n

Methyl 2-(1-(4-(1-hydroxy-2-(2-phenylaziridin-1-yl)ethyl)benzoyl)-5-methoxy-2-methyl-1H-indol-3-yl)acetate (5n) Prepared from methyl 2-(5-methoxy-2-methyl-1-(4-vinylbenzoyl)-1H-indol-3-yl)acetate (**4n**, 54.0 mg, 0.150 mmol, 1.50 equiv) and 2,4,6-triphenyl-1-(2-phenylaziridin-1-yl)pyridin-1-ium tetrafluoroborate (**2a**, 51.0 mg, 0.100 mmol, 1.00 equiv), and obtained as a 1:1 mixture of two diastereomers as a off white solid (24.0 mg, 48% yield). ^{1}H NMR (400 MHz, $CDCl_3$): δ 7.69 (d, *J* = 8.1 Hz, 2H), 7.51 (d, *J* = 8.0 Hz, 2H), 7.41–7.16 (m, 9H), 6.95 (d, *J* = 2.5 Hz, 1H), 6.88 (dd, *J* = 9.1, 1.8 Hz, 1H), 6.64 (dd, *J* = 9.0, 2.4 Hz, 1H), 4.97 (dd, *J* = 8.8, 3.5 Hz, 1H), 3.83 (s, 2H), 3.69 (d, *J* = 13.2 Hz, 5H), 2.92 (dd, *J* = 12.0, 8.8 Hz, 1H), 2.60–2.44 (m, 2H), 2.37 (s, 3H), 2.01 (d, *J* = 3.3 Hz, 1H), 1.84 (d, *J* = 6.8 Hz, 1H). ^{1}H NMR (400 MHz, $CDCl_3$): δ 7.66 (d, *J* = 8.2 Hz, 2H), 7.47 (d, *J* = 8.0 Hz, 2H), 7.38–7.27 (m, 4H), 7.24–7.14 (m, 5H), 6.95 (d, *J* = 2.5 Hz, 1H), 6.89 (d, *J* = 9.0 Hz, 1H), 6.65 (dd, *J* = 9.0, 2.5 Hz, 1H), 4.99 (dd, *J* = 8.6, 3.6 Hz, 1H), 3.84 (s, 2H), 3.70 (s, 3H), 3.67 (s, 2H), 2.89 (dd, *J* = 11.9, 8.7 Hz, 1H), 2.56 (dd, *J* = 11.9, 3.6 Hz, 1H), 2.50 (dd, *J* = 6.5, 3.4 Hz, 1H), 2.36 (s, 3H), 1.98 (d, *J* = 3.4 Hz, 1H), 1.82 (d, *J* = 6.6 Hz, 1H). ^{13}C NMR (126 MHz, $CDCl_3$): δ 171.4, 169.2, 155.9, 147.0, 139.3, 136.0, 134.7, 130.9, 130.5, 129.8, 128.3, 127.2, 126.2, 126.0, 115.0, 112.1, 111.5, 101.1, 72.4, 68.1, 55.7, 52.1, 41.4, 37.5, 30.2, 13.3. ^{13}C NMR (126 MHz, $CDCl_3$): δ 171.4, 169.2, 155.9, 147.0, 139.4, 136.0, 134.7, 130.9, 130.5, 129.8, 128.4, 128.3, 127.2, 126.2, 126.2, 126.0, 115.0, 112.1, 111.5, 101.1, 72.6, 68.1, 55.7, 52.1, 41.1, 38.2, 29.6, 13.3. HRMS-ESI^{+}: calculated for $C_{30}H_{31}N_2O_5$ [M+H^{+}] = 499.2227, observed [M+H^{+}] = 499.2217.

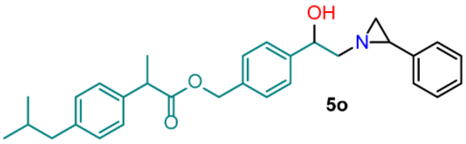

5o

4-(1-hydroxy-2-(2-phenylaziridin-1-yl)ethyl)benzyl 2-(4-isobutylphenyl)propanoate (5o) Prepared from 4-vinylbenzyl 2-(4-isobutylphenyl)propanoate (**4o**, 48.0 mg, 0.150 mmol, 1.50 equiv) and 2,4,6-triphenyl-1-(2-phenylaziridin-1-yl)pyridin-1-ium tetrafluoroborate (**2a**, 51.0 mg, 0.100 mmol, 1.00 equiv), and obtained as a 1:1 mixture of two diastereomers as an off white solid (24.7 mg, 54% yield). ^{1}H NMR (400 MHz, $CDCl_3$): δ 7.37–7.24 (m, 5H), 7.26–7.14 (m, 6H), 7.09 (d, *J* = 7.8 Hz, 2H), 5.08 (d, *J* = 5.6 Hz, 2H), 4.88 (dd, *J* = 9.0, 3.5 Hz, 1H), 3.74 (q, *J* = 7.2 Hz, 1H), 3.36 (br, 1H), 2.83 (dd, *J* = 12.0, 8.9 Hz, 1H), 2.46 (t, *J* = 6.4 Hz, 4H), 1.95 (d, *J* = 3.5 Hz, 1H), 1.85 (p, *J* = 6.7 Hz, 1H), 1.77 (d, *J* = 6.5 Hz, 1H), 1.51 (d, *J* = 7.3 Hz, 3H), 0.90 (d, *J* = 6.7 Hz, 6H). ^{1}H NMR (400 MHz, $CDCl_3$): δ 7.30 (dd, *J* = 7.8, 3.5 Hz, 4H), 7.24–7.13 (m, 6H), 7.08 (d, *J* = 7.8 Hz, 3H), 5.33–4.99 (m, 2H), 4.86 (dd, *J* = 9.2, 3.4 Hz, 1H), 3.74 (q, *J* = 7.1 Hz, 1H), 3.31 (br, 1H), 2.92 (dd, *J* = 12.0, 9.1 Hz, 1H), 2.67–2.22 (m, 5H), 1.99 (d, *J* = 3.4 Hz, 1H), 1.84 (m, 2H), 1.50 (d, *J* = 7.2 Hz, 3H), 0.90 (d, *J* = 6.6 Hz, 6H). ^{13}C NMR

(101 MHz, $CDCl_3$): δ 141.6, 140.5, 139.5, 137.5, 135.3, 129.2, 128.2, 127.8, 127.1, 127.0, 126.0, 126.0, 72.6, 68.4, 66.0, 45.1, 45.0, 41.3, 37.3, 30.1, 22.3, 18.4. ^{13}C NMR (101 MHz, $CDCl_3$): δ 174.5, 141.6, 140.5, 139.6, 137.5, 135.4, 129.3, 128.3, 127.8, 127.2, 127.0, 126.0, 126.0, 126.0, 72.8, 68.4, 66.0, 45.1, 45.0, 41.1, 38.0, 30.1, 22.3, 18.4. HRMS-ESI$^+$: calculated for $C_{30}H_{36}NO_3$ [M+H$^+$] = 458.2690, observed [M+H$^+$] = 458.2680.

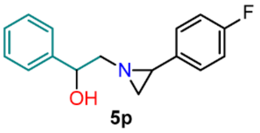

2-(2-(4-Fluorophenyl)aziridin-1-yl)-1-phenylethan-1-ol (5p) Prepared from styrene (**4a**, 17.0 μL, 0.150 mmol, 1.50 equiv) and 1-(2-(4-fluorophenyl)aziridin-1-yl)-2,4,6-triphenylpyridine-1-ium tetrafluoroborate (**2p**, 53.0, 0.100 mmol, 1.00 equiv), and obtained as a 1:1 mixture of two diastereomers as a yellow liquid (16.0 mg, 62% yield). ^1H NMR (400 MHz, $CDCl_3$): δ 7.27–7.21 (m, 5H), 7.10–7.07 (m, 2H), 6.93–6.89 (m, 2H), 4.82 (dd, J = 8.8, 3.5 Hz, 1H), 3.24 (br, 1H), 2.77 (dd, J = 12.0, 8.7 Hz, 1H), 2.45 (dd, J = 12.0, 3.5 Hz, 1H), 2.37 (dd, J = 6.5, 3.4 Hz, 1H), 1.82 (d, J = 3.4 Hz, 1H), 1.68 (d, J = 6.5 Hz, 1H) ppm. ^1H NMR (400 MHz, $CDCl_3$): δ 7.37–7.31 (m, 5H), 7.19–7.17 (m, 2H), 7.01–6.97 (m, 1H), 4.87 (dd, J = 8.9, 3.6 Hz, 1H), 3.22 (br,1H), 2.94 (dd, J = 12.1, 9.0 Hz, 1H), 2.54–2.35 (m, 2H), 1.95 (d, J = 3.4 Hz, 1H), 1.82 (d, J = 6.5 Hz, 1H) ppm. ^{13}C NMR (101 MHz, $CDCl_3$): δ 162.0 (d, J = 243.0 Hz), 141.7, 135.3 (d, J = 2.5 Hz),128.3, 127.6, 127.5, 125.9, 115.1 (d, J = 21.2 Hz), 72.9, 68.4, 40.7, 37.2. ^{13}C NMR (101 MHz, $CDCl_3$): δ 162.0 (d, J = 243.0 Hz), 141.7, 135.4 (d, J = 3.7 Hz), 128.3, 127.6, 127.5 (d, J = 8.7 Hz), 126.0, 115.6, 115.0, 73.2, 68.4, 40.4, 38.0. HRMS-ESI$^+$: calculated for $C_{16}H_{17}FNO$ [M+H$^+$]= 258.1289, observed [M+H$^+$] = 258.1284.

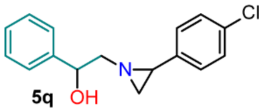

2-(2-(4-Chlorophenyl)aziridin-1-yl)-1-phenylethan-1-ol (5q) Prepared from styrene (**4a**, 17.0 μL, 0.150 mmol, 1.50 equiv) and 1-(2-(4-chlorophenyl)aziridin-1-yl)-2,4,6-triphenylpyridine-1-ium tetrafluoroborate (**2q**, 54.0 mg, 0.100 mmol, 1.00 equiv), and obtained as a 1:1 mixture of two diastereomers as a yellow liquid (16.0 mg, 59% yield). ^1H NMR (400 MHz, $CDCl_3$): δ 7.27–7.24 (m, 4H), 7.22–7.18 (m, 3H), 7.05 (d, J = 8.6 Hz, 1H), 4.82 (dd, J = 8.6, 3.5 Hz, 1H), 3.17 (br, 1H), 2.75 (dd, J = 12.0, 8.6 Hz, 1H), 2.47 (dd, J = 12.0, 3.5 Hz, 1H), 2.35 (dd, J = 6.5, 3.4 Hz, 1H), 1.82 (d, J = 3.4 Hz, 1H), 1.69 (d, J = 6.5 Hz, 1H).^1H NMR (400 MHz, $CDCl_3$): δ 7.37–7.26 (m, 7H), 7.15 (d, J = 8.5 Hz, 2H), 4.87 (dd, J = 8.9, 3.6 Hz, 1H), 3.19 (br, 1H), 2.94 (dd, J = 12.0, 8.8 Hz, 1H), 2.43–2.36 (m, 2H), 1.95 (d, J = 3.3 Hz, 1H), 1.84 (d, J = 6.5 Hz, 1H). ^{13}C NMR (101 MHz, $CDCl_3$): δ 141.7, 138.2, 132.7, 128.3, 128.3, 127.6, 127.4, 125.9, 72.9, 68.3, 40.7, 37.4. ^{13}C NMR (101 MHz, $CDCl_3$): δ 141.7, 138.3, 132.7, 128.4, 128.3, 127.7, 127.3, 125.9, 73.2, 68.4, 40.4, 38.2. HRMS-ESI$^+$: calculated for $C_{16}H_{17}ClNO$ [M+H$^+$] = 274.0993, observed [M+H$^+$] = 274.0982.

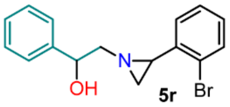

5r

2-(2-(2-Bromophenyl)aziridin-1-yl)-1-phenylethan-1-ol (5r). Prepared from styrene (**4a**, 17.0 µL, 0.150 mmol, 1.50 equiv) and 1-(2-(2-bromophenyl)aziridin-1-yl)-2,4,6-triphenylpyridine-1-ium tetrafluoroborate (**2r**, 59.0 mg, 0.100 mmol, 1.00 equiv), and obtained as a 1:1 mixture of two diastereomers as a yellow liquid (16.8 mg, 53% yield). ^{1}H NMR (400 MHz, $CDCl_3$): δ 7.52 (d, *J* = 3.3 Hz, 1H), 7.39–7.25 (m, 7H), 7.13–7.09 (m, 1H), 4.91 (dd, *J* = 9.2, 3.5 Hz, 1H), 3.32 (br, 1H), 3.07 (dd, *J* = 11.9, 9.2 Hz, 1H), 2.84 (dd, *J* = 6.6, 3.4 Hz, 1H), 2.42 (dd, *J* = 12.0, 3.5 Hz, 1H), 1.87 (d, *J* = 3.4 Hz, 1H), 1.84 (d, *J* = 6.6 Hz, 1H) ppm. ^{1}H NMR (400 MHz, $CDCl_3$): δ 7.53 (d, *J* = 8.0 Hz, 1H), 7.40–7.32 (m, 4H), 7.30–7.27 (m, 3H), 7.13–7.09 (m, 1H), 4.93 (dd, *J* = 9.0, 3.3 Hz, 1H), 3.24 (br, 1H), 2.92 (dd, *J* = 12.1, 9.0 Hz, 1H), 2.77 (dd, *J* = 6.6, 3.4 Hz, 1H), 2.56 (dd, *J* = 12.1, 3.3 Hz, 1H), 1.93 (d, *J* = 3.4 Hz, 1H), 1.90 (d, *J* = 6.6 Hz, 1H). ^{13}C NMR (101 MHz, $CDCl_3$): δ 141.6, 138.7, 132.2, 128.4, 128.3, 127.7, 127.6, 127.4, 125.9, 123.8, 72.9, 68.3, 41.1, 37.1. ^{13}C NMR (101 MHz, $CDCl_3$): δ 141.7, 138.7, 132.2, 128.4, 128.3, 127.6, 127.5, 127.4, 125.9, 123.8, 73.3, 68.3, 41.1, 37.7. HRMS-ESI^{+}: calculated for $C_{16}H_{17}BrNO$ [M+H^{+}] = 318.0488, observed [M+H^{+}] = 318.0483.

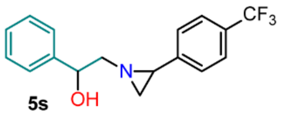

5s

1-Phenyl-2-(2-(4-(trifluoromethyl)phenyl)aziridin-1-yl)ethan-1-ol (5s): Prepared from styrene (**4a**, 17.0 µL, 0.150 mmol, 1.50 equiv) and 2,4,6-triphenyl-1-(2-(4-(trifluoromethyl)phenyl)aziridin-1-yl)pyridine-1-ium tetrafluoroborate (**2s**, 58.0 mg, 0.100 mmol, 1.00 equiv), and obtained as a 1:1 mixture of two diastereomers as a yellow liquid (15.3 mg, 50% yield). ^{1}H NMR (400 MHz, $CDCl_3$): δ 7.56–7.54 (m, 2H), 7.35–7.28 (m, 7H), 4.91 (dd, *J* = 8.6, 3.5 Hz, 1H), 3.19 (br, 1H), 2.84 (dd, *J* = 12.0, 8.5 Hz, 1H), 2.59 (dd, *J* = 12.0, 3.5 Hz, 1H), 2.50 (dd, *J* = 6.5, 3.4 Hz, 1H), 1.93 (d, *J* = 3.4 Hz, 1H), 1.83 (d, *J* = 6.5 Hz, 1H). ^{1}H NMR (400 MHz, $CDCl_3$): δ 7.55 (d, *J* = 8.0 Hz, 2H), 7.41–7.27 (m, 7H), 4.88 (dd, *J* = 8.8, 3.7 Hz, 1H), 3.05 (br,1H), 2.97 (dd, *J* = 12.1, 8.7 Hz, 1H), 2.58–2.38 (m, 2H), 1.99 (d, *J* = 3.2 Hz, 1H), 1.90 (d, *J* = 6.5 Hz, 1H). ^{13}C NMR (101 MHz, $CDCl_3$): δ 144.0, 141.7, 129.2 (q, *J* = 32.2 Hz), 128.4, 127.7, 127.1 (q, *J* = 274.0 Hz), 126.4, 125.9, 125.2 (q, *J* = 3.9 Hz), 73.0, 68.3, 40.9, 37.7. ^{13}C NMR (101 MHz, $CDCl_3$): δ 144.0, 141.6, 129.2 (q, *J* = 33.1 Hz), 128.4, 127.7, 127.1 (q, *J* = 270.0 Hz), 126.3, 126.0, 125.2 (q, *J* = 3.9 Hz), 73.3, 68.3, 40.6, 38.6. HRMS-ESI^{+}: calculated for $C_{17}H_{17}F_3NO$ [M+H^{+}] = 308.1257, observed [M+H^{+}] = 308.1248.

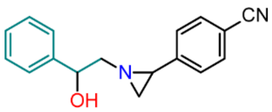

5t

4-(1-(2-Hydroxy-2-phenylethyl)aziridin-2-yl)benzonitrile (5t) Prepared from styrene (**4a**, 17.0 µL, 0.150 mmol, 1.50 equiv) and 4-(1-(2,4,6-triphenyl-pyridin-1-yl)aziridin-2-yl)benzonitrilepyridine-1-ium tetrafluoroborate (**2t**, 45.0 mg, 0.100 mmol, 1.00 equiv), and obtained as a 1:1 mixture of two diastereomers as a yellow liquid (16.5 mg, 62% yield). ^{1}H NMR (400 MHz, $CDCl_3$): δ 7.49 (d, J = 8.1 Hz, 2H), 7.28–7.14 (m, 7H), 4.83 (dd, J = 8.3, 3.6 Hz, 1H), 3.06 (br,1H), 2.74 (dd, J = 12.1, 8.3 Hz, 1H), 2.55 (dd, J = 12.1, 3.6 Hz, 1H), 2.39 (dd, J = 6.6, 3.3 Hz, 1H), 1.84 (d, J = 3.4 Hz, 1H), 1.77 (d, J = 6.5 Hz, 1H).ppm. ^{1}H NMR (400 MHz, $CDCl_3$): δ 7.54–7.45 (m, 2H), 7.33–7.19 (m, 7H), 4.80 (dd, J = 8.6, 3.9 Hz, 1H), 2.89 (dd, J = 12.1, 8.6 Hz, 1H), 2.39 (dd, J = 12.1, 3.9 Hz, 1H), 2.33 (dd, J = 6.5, 3.2 Hz, 1H), 1.90 (d, J = 3.3 Hz, 1H), 1.85 (d, J = 6.5 Hz, 1H). ^{13}C NMR (101 MHz, $CDCl_3$): δ 145.5, 141.6, 132.0, 128.3, 127.7, 126.8, 125.8, 118.9, 110.6, 72.9, 68.1, 40.9, 38.0. ^{13}C NMR (101 MHz, $CDCl_3$): δ 145.6, 141.6, 132.1, 128.4, 127.8, 126.7, 126.0, 118.9, 110.7, 73.3, 68.2, 40.6, 38.9. HRMS-ESI$^+$: calculated for $C_{17}H_{17}N_2O$ [M+H$^+$] = 265.1335, observed [M+H$^+$] = 265.1326.

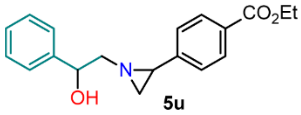

5u

Ethyl 4-(1-(2-hydroxy-2-phenylethyl)aziridin-2-yl)benzoate (5u) Prepared from styrene (**4a**, 17.0 µL, 0.150 mmol, 1.50 equiv) and 4-(1-(2,4,6-triphenyl-pyridin-1-yl)aziridin-2-yl)benzonitrilepyridine-1-ium tetrafluoroborate (**2u**, 58.0 mg, 0.100 mmol, 1.00 equiv), and obtained as a 1:1 mixture of two diastereomers as a yellow liquid (17.4 mg, 56% yield). ^{1}H NMR (400 MHz, $CDCl_3$): δ 8.11–7.78 (m, 2H), 7.45–7.18 (m, 7H), 4.91 (dd, J = 8.6, 3.5 Hz, 1H), 4.37 (q, J = 7.1 Hz, 2H), 3.22 (s, 1H), 2.85 (dd, J = 12.0, 8.5 Hz, 1H), 2.58 (dd, J = 12.0, 3.6 Hz, 1H), 2.49 (dd, J = 6.5, 3.3 Hz, 1H), 1.95 (d, J = 3.3 Hz, 1H), 1.83 (d, J = 6.6 Hz, 1H), 1.40 (t, J = 7.1 Hz, 3H). ^{1}H NMR (400 MHz, $CDCl_3$): δ 8.02–7.81 (m, 2H), 7.36–7.19 (m, 7H), 4.81 (dd, J = 8.8, 3.7 Hz, 1H), 3.10 (br, 1H), 2.89 (dd, J = 12.0, 8.8 Hz, 1H), 2.43–2.24 (m, 2H), 1.93 (d, J = 3.2 Hz, 1H), 1.82 (d, J = 6.6 Hz, 1H). ^{13}C NMR (126 MHz, $CDCl_3$): δ 166.5, 145.0, 141.7, 129.5, 129.2, 128.3, 127.6, 125.9, 125.8, 72.9, 68.3, 60.8, 41.1, 37.8, 14.3. ^{13}C NMR (126 MHz, $CDCl_3$): δ 166.4, 145.1, 141.6, 129.6, 129.2, 128.3, 127.7, 126.0, 125.9, 73.2, 68.4, 60.8, 40.8, 38.6, 14.3. HRMS-ESI$^+$: calculated for $C_{19}H_{22}NO_3$ [M+H$^+$] = 312.1594, observed [M$^+$] = 312.1585.

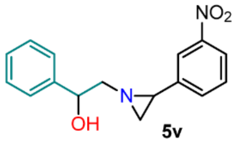

2-(2-(3-Nitrophenyl)aziridin-1-yl)-1-phenylethan-1-ol (5v) Prepared from styrene (**4a**, 17.0 µL, 0.150 mmol, 1.50 equiv) and 1-(2-(3-nitrophenyl)aziridin-1-yl)-2,4,6-triphenylpyridine-1-ium tetrafluoroborate (**2v**, 56.0 mg, 0.100 mmol, 1.00 equiv), and obtained as a 1:1 mixture of two diastereomers as yellow liquid (11.3 mg, 40% yield). ^{1}H NMR (400 MHz, $CDCl_3$): δ 8.10–8.07 (m, 1H), 8.04 (t, *J* = 2.0 Hz, 1H), 7.55–7.40 (m, 2H), 7.38–7.27 (m, 5H), 4.93 (dd, *J* = 8.3, 3.5 Hz, 1H), 2.84 (dd, *J* = 12.0, 8.3 Hz, 1H), 2.65 (dd, *J* = 12.0, 3.5 Hz, 1H), 2.53 (dd, *J* = 6.5, 3.3 Hz, 1H), 1.96 (d, *J* = 3.3 Hz, 1H), 1.85 (d, *J* = 6.5 Hz, 1H). ^{1}H NMR (400 MHz, $CDCl_3$): δ 8.19–8.05 (m, 1H), 8.05 (s, 1H), 7.53–7.42 (m, 2H), 7.41–7.27 (m, 5H), 4.90 (dd, *J* = 8.5, 4.0 Hz, 1H), 2.98 (dd, *J* = 12.1, 8.5 Hz, 1H), 2.60–2.36 (m, 2H), 2.01 (d, *J* = 3.2 Hz, 1H), 1.92 (d, *J* = 6.5 Hz, 1H). ^{13}C NMR (126 MHz, $CDCl_3$): δ 148.3, 142.2, 141.7, 132.2, 129.1, 128.3, 127.7, 125.8, 122.0, 121.2, 72.9, 68.2, 40.6, 37.6. ^{13}C NMR (126 MHz, $CDCl_3$): δ 148.3, 142.3, 141.6, 132.0, 129.2, 128.4, 127.8, 126.0, 122.0, 121.1, 73.3, 68.2, 40.2, 38.6. HRMS-ESI$^+$: calculated for $C_{16}H_{17}N_2O_3$ [M+H$^+$] = 285.1234, observed [M+H$^+$] = 285.1227.

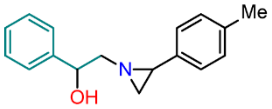

(1S)-1-Phenyl-2-(2-(p-tolyl)aziridin-1-yl)ethan-1-ol (5w) Prepared from styrene (**4a**, 17.0 µL, 0.150 mmol, 1.50 equiv) and 2,4,6-triphenyl-1-(2-(p-tolyl)aziridin-1-yl)pyridine-1-ium tetrafluoroborate (**2w**, 52.0 mg, 0.100 mmol, 1.00 equiv), and obtained as a 1:1 mixture of two diastereomers as a yellow liquid (11.1 mg, 44% yield). ^{1}H NMR (400 MHz, $CDCl_3$): δ 7.38–7.31(m, 3H), 7.29–7.26 (m, 2H), 7.15–7.11 (m, 4H), 4.87 (dd, *J* = 9.1, 3.4 Hz, 1H), 3.36 (br, 1H), 2.94 (dd, *J* = 11.9, 9.1 Hz, 1H), 2.42-2.34 (m, 5H), 1.98 (d, *J* = 3.4 Hz, 1H), 1.81 (d, *J* = 6.4 Hz, 1H). ^{1}H NMR (400 MHz, $CDCl_3$): δ 7.40–7.28 (m, 5H), 7.11 (m, 4H), 4.90 (dd, *J* = 9.1, 3.4 Hz, 1H), 3.35 (br, 1H), 2.86 (dd, *J* = 12.0, 9.1 Hz, 1H), 2.55–2.41 (m, 1H), 2.33 (s, 4H), 1.93 (d, *J* = 3.6 Hz, 1H), 1.75 (d, *J* = 6.5 Hz, 1H). ^{13}C NMR (101 MHz, $CDCl_3$): δ 141.8, 136.7, 136.5, 128.9, 128.3, 127.5, 126.0, 125.9, 72.9, 68.5, 41.1, 37.2, 21.0. ^{13}C NMR (101 MHz, $CDCl_3$): δ 141.7, 136.7, 136.7, 129.0, 128.3, 127.6, 125.9, 125.9, 73.1, 68.6, 40.9, 37.8, 21.0. HRMS-ESI$^+$: calculated for $C_{17}H_{20}NO$ [M+H$^+$] = 254.1539, observed [M+H$^+$] = 254.1530.

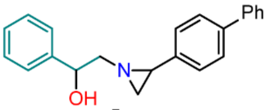

5x

2-(2-([1,1'-Biphenyl]-4-yl)aziridin-1-yl)-1-phenylethan-1-ol (5x) Prepared from styrene (**4a**, 17.0 μL, 0.150 mmol, 1.50 equiv) and 1-(2-([1,1'-biphenyl]-4-yl)aziridin-1-yl)-2,4,6-triphenylpyridine-1-ium tetrafluoroborate (**2x**, 59.0 mg, 0.100 mmol, 1.00 equiv), and obtained as a 1:1 mixture of two diastereomers as a yellow liquid (13.2 mg, 42% yield). ^{1}H NMR (400 MHz, $CDCl_3$): δ 7.60–7.53 (m, 4H), 7.46–7.42 (m, 2H), 7.38–7.27 (m, 8H), 4.93 (dd, *J* = 9.0, 3.4 Hz, 1H), 3.32 (br, 1H), 2.89 (dd, *J* = 11.9, 9.0 Hz, 1H), 2.55–2.51 (m, 2H), 2.00 (d, *J* = 3.4 Hz, 1H), 1.81 (d, *J* = 6.6 Hz, 1H) ppm. ^{13}C NMR (101 MHz, $CDCl_3$): δ 141.8, 140.9, 140.0, 138.7, 128.7, 128.3, 127.6, 127.1, 127.0, 127.0, 126.5, 125.9, 72.9, 68.5, 41.0, 37.4. HRMS-ESI$^+$: calculated for $C_{22}H_{22}NO$ [M+H$^+$] = 316.1696, observed [M+H$^+$] = 316.1688.

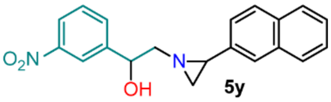

5y

2-(2-(Naphthalen-2-yl)aziridin-1-yl)-1-(3-nitrophenyl)ethan-1-ol (5y) Prepared from 1-nitro-3-vinylbenzene (**4a**, 17.0 μL, 0.150 mmol, 1.50 equiv) and 1-(2-(naphthalen-2-yl)aziridin-1-yl)-2,4,6-triphenylpyridine-1-ium tetrafluoroborate (**2y**, 56.0 mg, 0.100 mmol, 1.00 equiv), and obtained as a 1:1 mixture of two diastereomers as a yellow liquid (23.0 mg, 69% yield). ^{1}H NMR (400 MHz, $CDCl_3$): δ 8.22 (d, *J* = 2.1 Hz, 1H), 8.09 (m, 1H), 7.86–7.72 (m, 3H), 7.71–7.63 (m, 1H), 7.53–7.33 (m, 3H), 7.32–7.20 (m, 2H), 5.02 (dd, *J* = 8.1, 3.8 Hz, 1H), 2.84 (dd, *J* = 12.0, 8.1 Hz, 1H), 2.72 (dd, *J* = 12.0, 3.8 Hz, 1H), 2.58 (dd, *J* = 6.6, 3.5 Hz, 1H), 2.09 (d, *J* = 3.4 Hz, 1H), 1.89 (d, *J* = 6.6 Hz, 1H). ^{1}H NMR (500 MHz, $CDCl_3$): δ 8.27 (s, 1H), 8.19–8.02 (m, 1H), 7.94–7.65 (m, 5H), 7.47 (m, 3H), 7.31 (dd, *J* = 8.4, 1.8 Hz, 1H), 5.01 (dd, *J* = 8.7, 3.8 Hz, 1H), 3.58 (br, 1H), 3.00 (m, 1H), 2.73–2.56 (m, 1H), 2.52 (dd, *J* = 12.1, 3.8 Hz, 1H), 2.11 (d, *J* = 3.4 Hz, 1H), 1.91 (d, *J* = 6.6 Hz, 1H). ^{13}C NMR (101 MHz, $CDCl_3$): δ 148.2, 144.1, 136.6, 133.2, 132.7, 132.0, 129.2, 128.1, 127.6, 127.5, 127.5, 126.2, 126.2, 125.6, 124.9, 123.8, 123.7, 122.4, 121.0, 71.9, 68.0, 41.9, 37.3. ^{13}C NMR (101 MHz, $CDCl_3$): δ 148.2, 144.0, 136.8, 133.2, 132.8, 132.1, 129.2, 128.2, 127.7, 127.5, 126.2, 125.7, 124.9, 123.8, 122.5, 121.1, 72.2, 68.0, 41.6, 38.0. HRMS-ESI$^+$: calculated for $C_{20}H_{19}N_2O_3$ [M+H$^+$] = 335.1390, observed [M+H$^+$] = 335.1378.

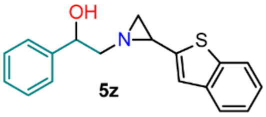

5z

2-(2-(Benzo[b]thiophen-2-yl)aziridin-1-yl)-1-phenylethan-1-ol (5z) Prepared from styrene (**4a**, 17.0 μL, 0.150 mmol, 1.50 equiv) and 1-(2-(benzo[b]thiophen-6-yl)aziridin-1-yl)-2,4,6-triphenylpyridine-1-ium tetrafluoroborate (**2z**, 57.0 mg, 0.100 mmol, 1.00 equiv), and obtained as a 1:1 mixture of two diastereomers as a yellow liquid (16.8 mg, 57% yield). ^{1}H NMR (400 MHz, $CDCl_3$): δ 7.85–7.74 (m, 1H), 7.74–7.58 (m, 1H), 7.44–7.27 (m, 7H), 7.16 (s, 1H), 4.95

(dd, J = 8.7, 3.5 Hz, 1H), 3.22 (br, 1H), 2.87–2.72 (m, 2H), 2.65 (dd, J = 12.0, 3.6 Hz, 1H), 2.12 (d, J = 3.3 Hz, 1H), 1.85 (d, J = 6.4 Hz, 1H). ^{1}H NMR (400 MHz, $CDCl_3$): δ 7.80–7.73 (m, 1H), 7.72–7.64 (m, 1H), 7.45–7.27 (m, 7H), 7.18 (s, 1H), 4.94 (dd, J = 9.0, 3.5 Hz, 1H), 3.26 (br, 1H), 2.99 (dd, J = 12.1, 9.0 Hz, 1H), 2.76 (dd, J = 6.4, 3.3 Hz, 1H), 2.43 (dd, J = 12.1, 3.5 Hz, 1H), 2.19 (d, J = 3.2 Hz, 1H), 1.93 (d, J = 6.3 Hz, 1H). ^{13}C NMR (126 MHz, $CDCl_3$): δ 145.3, 141.7, 139.9, 138.8, 128.3, 127.6, 125.9, 124.2, 123.8, 122.9, 122.3, 120.6, 73.0, 68.2, 38.2, 37.9. ^{13}C NMR (126 MHz, $CDCl_3$): δ 145.4, 141. 6, 140.0, 138.8, 128.4, 127.7, 126.0, 124.3, 123.9, 122.9, 122.3, 120.5, 73.2, 68.2, 38.7, 37.7. HRMS-ESI$^+$: calculated for $C_{18}H_{18}NOS$ [M+H$^+$] = 296.1104, observed [M+H$^+$] = 296.1099.

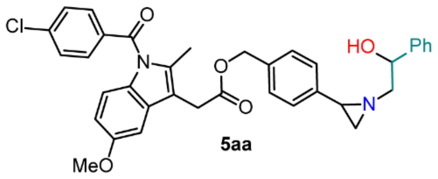

5aa

4-(1-(2-Hydroxy-2-phenylethyl)aziridin-2-yl)benzyl 2-(1-(4-chlorobenzoyl)-5-methoxy-2-methyl-1H-indol-3-yl)acetate (5aa) Prepared from styrene (**4a**, 17.0 µL, 0.150 mmol, 1.50 equiv) and 4-(1-(2,4,6-triphenylpyridin-1-yl)aziridin-2-yl)benzyl 2-(1-(4-chlorobenzoyl)-5-methoxy-2-methyl-1H-indol-3-yl)acetate tetrafluoroborate (**2aa**, 88.0 mg, 0.100 mmol, 1.00 equiv), and obtained as a 1:1 mixture of two diastereomers as a yellow liquid (29.0 mg, 48% yield). δ ^{1}H NMR (400 MHz, $CDCl_3$): δ 7.64 (d, J = 8.5 Hz, 2H), 7.46 (d, J = 8.5 Hz, 2H), 7.41–7.22 (m, 7H), 7.17 (d, J = 8.1 Hz, 2H), 6.98–6.81 (m, 2H), 6.67 (dd, J = 9.0, 2.6 Hz, 1H), 5.12 (s, 2H), 4.90 (dd, J = 9.3, 3.3 Hz, 1H), 3.77 (s, 3H), 3.70 (s, 2H), 3.29 (br, 1H), 2.85 (dd, J = 12.0, 8.9 Hz, 1H), 2.55–2.43 (m, 2H), 2.36 (s, 3H), 1.92 (d, J = 3.4 Hz, 1H), 1.78 (d, J = 6.5 Hz, 1H). ^{1}H NMR (400 MHz, $CDCl_3$): δ 7.65 (d, J = 8.5 Hz, 2H), 7.46 (d, J = 8.5 Hz, 2H), 7.39–7.14 (m, 9H), 7.03–6.77 (m, 2H), 6.67 (dd, J = 9.0, 2.6 Hz, 1H), 5.12 (s, 2H), 4.86 (dd, J = 9.1, 3.4 Hz, 1H), 3.77 (s, 3H), 3.70 (s, 2H), 3.27 (br, 1H), 2.95 (dd, J = 12.0, 9.1 Hz, 1H), 2.51–2.26 (m, 6H), 1.98 (d, J = 3.3 Hz, 1H), 1.85 (d, J = 6.6 Hz, 1H). ^{13}C NMR (101 MHz, $CDCl_3$): δ 170.6, 168.2, 156.0, 141.7, 140.0, 139.2, 135.9, 134.5, 133.8, 131.1, 130.7, 130.5, 129.1, 128.3, 128.2, 127.6, 126.2, 125.9, 114.9, 112.4, 111.8, 101.1, 72.9, 68.4, 66.5, 55.6, 41.0, 37.4, 30.4, 13.3. ^{13}C NMR (126 MHz, $CDCl_3$): δ 170.6, 168.2, 156.0, 141.6, 140.1, 139.2, 135.9, 134.5, 133.8, 131.1, 130.7, 130.5, 129.1, 128.3, 128.3, 127.6, 126.2, 125.9, 114.9, 112.4, 111.8, 101.1, 73.2, 68.5, 66.5, 55.6, 40.7, 38.2, 30.4, 13.3. HRMS-ESI$^+$: calculated for $C_{36}H_{34}ClN_2O_5$ [M+H$^+$] = 609.2151, observed [M+H$^+$] = 609.21412.

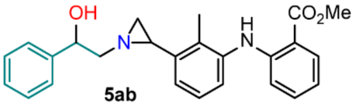

5ab

Methyl 2-((3-(1-(2-hydroxy-2-phenylethyl)aziridin-2-yl)-2-methylphenyl)amino)-benzoate (5ab) Prepared from styrene (**4a**, 17.0 µL, 0.150 mmol, 1.50 equiv) and 1-methyl-2-((2-methyl-3-(1-(2,4,6-triphenylpyridin-1-yl)aziridin-2-yl)phenyl)amino)benzoate tetrafluoroborate (**2ab**, 67.0 mg, 0.100 mmol, 1.00 equiv), and obtained as a 1:1 mixture of two diastereomers as a yellow liquid (26.0 mg, 65% yield). ^{1}H NMR (400 MHz, $CDCl_3$): δ 9.25 (s, 1H), 7.96 (dd, *J* = 8.0, 1.7 Hz, 1H), 7.61–7.29 (m, 5H), 7.24–7.03 (m, 4H), 6.76 (dd, *J* = 8.6, 1.1 Hz, 1H), 6.70–6.66 (m, 1H), 4.94 (dd, *J* = 9.1, 3.4 Hz, 1H), 3.92 (s, 3H), 3.41 (br, 1H), 3.04 (dd, *J* = 11.9, 9.2 Hz, 1H), 2.64 (dd, *J* = 6.6, 3.6 Hz, 1H), 2.43 (dd, *J* = 11.9, 3.4 Hz, 1H), 2.27 (s, 3H), 1.91 (d, *J* = 3.5 Hz, 1H), 1.81 (d, *J* = 6.6 Hz, 1H). ^{1}H NMR (500 MHz, $CDCl_3$): δ 9.26 (s, 1H), 7.97 (dd, *J* = 8.1, 1.7 Hz, 1H), 7.46–7.38 (m, 2H), 7.38–7.32 (m, 2H), 7.31–7.15 (m, 5H), 6.78 (dd, *J* = 8.5, 1.1 Hz, 1H), 6.68 (m, 1H), 4.95 (dd, *J* = 8.9, 3.4 Hz, 1H), 3.92 (s, 3H), 3.37 (br, 1H), 2.92 (dd, *J* = 12.1, 8.9 Hz, 1H), 2.64–2.49 (m, 2H), 2.30 (s, 3H), 1.92 (d, *J* = 3.4 Hz, 1H), 1.86 (d, *J* = 6.6 Hz, 1H). ^{13}C NMR (101 MHz, $CDCl_3$): δ 169.1, 149.2, 141.8, 139.3, 138.7, 134.1, 131.9, 131.4, 128.3, 127.6, 126.4, 125.9, 123.9, 122.8, 116.2, 113.7, 110.9, 73.3, 68.6, 51.7, 39.7, 37.2, 13.3. ^{13}C NMR (101 MHz, $CDCl_3$): δ 169.1, 149.2, 141.7, 139.2, 138.7, 134.1, 132.2, 131.4, 128.3, 127.6, 126.3, 125.9, 124.0, 122.9, 116.2, 113.6, 110.9, 72.9, 68.6, 51.7, 39.6, 36.4, 13.4. HRMS-ESI^{+}: calculated for $C_{25}H_{27}N_2O_3$ [M+H^{+}] = 403.2016, observed [M+H^{+}] = 403.2008.

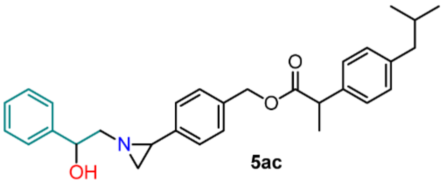

5ac

4-(1-(2-Hydroxy-2-phenylethyl)aziridin-2-yl)benzyl-2-(4-isobutylphenyl)propanoate (5ac) Prepared from styrene (**4a**, 17.0 µL, 0.150 mmol, 1.50 equiv) and 1-methyl-4-(1-(2,4,6-triphenylpyridin-1-yl)aziridin-2-yl)benzyl 2-(4-isobutylphenyl)propanoate tetrafluoroborate (**2ac**, 73.0 mg, 0.100 mmol, 1.00 equiv), and obtained as a 1:1 mixture of two diastereomers as a yellow liquid (29.0 mg, 63% yield).^{1}H NMR (400 MHz, $CDCl_3$): δ 7.39–7.27 (m, 4H), 7.23–7.06 (m, 9H), 5.21–4.97 (m, 2H), 4.90 (dd, *J* = 8.9, 3.4 Hz, 1H), 3.74 (q, *J* = 7.2 Hz, 1H), 3.29 (br, 1H), 2.85 (dd, *J* = 11.9, 8.9 Hz, 1H), 2.60–2.38 (m, 4H), 1.92 (d, *J* = 3.4 Hz, 1H), 1.85 (m, 1H), 1.77 (d, *J* = 6.5 Hz, 1H), 1.51 (d, *J* = 7.2 Hz, 3H), 0.91 (d, *J* = 6.6 Hz, 6H). ^{1}H NMR (400 MHz, $CDCl_3$): δ 7.42–7.26 (m, 5H), 7.24–7.02 (m, 8H), 5.15–5.00 (m, 2H), 4.86 (dd, *J* = 9.2, 3.4 Hz, 1H), 3.74 (q, *J* = 7.2 Hz, 1H), 3.26 (br, 1H), 2.94 (dd, *J* = 12.0, 9.1 Hz, 1H), 2.45 (d, *J* = 7.2 Hz, 2H), 2.43–2.35 (m, 2H), 1.97 (d, *J* = 3.3 Hz, 1H), 1.90–1.80 (m, 2H), 1.50 (d, *J* = 7.2 Hz, 3H), 0.90 (d, *J* = 6.6 Hz, 6H). ^{13}C NMR (101 MHz, CDC13): δ 174.5, 141.7, 140.5, 139.6, 137.5, 134.9, 129.2, 128.3, 127.8, 127.6, 127.2, 126.1, 125.9, 72.9, 68.4, 66.1, 45.1, 45.0, 41.0, 37.4, 30.1, 22.3, 18.4. ^{13}C NMR (126 MHz, $CDCl_3$): δ 174.5, 141.6, 140.5, 139.7, 137.5, 134.9, 129.3, 129.3, 128.3, 127.9, 127.6, 127.2, 126.1, 125.9, 73.1, 68.5, 66.1, 45.1, 45.0, 40.8, 38.1,

30.1, 22.3, 18.4. HRMS-ESI+: calculated for $C_{30}H_{36}NO_3$ [M+H+] = 458.2690, observed [M+H+] = 458.2676.

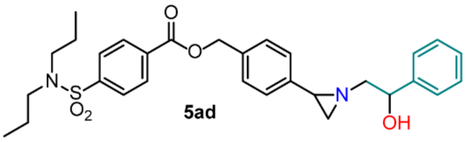

5ad

4-(1-(2-Hydroxy-2-phenylethyl)aziridin-2-yl)benzyl 4-(*N*,*N*-dipropylsulfamoyl)benzoate (5ad) Prepared from styrene (**4a**, 17.0 μL, 0.150 mmol, 1.50 equiv) and 4-(1-(2,4,6-triphenylpyridin-1-yl)aziridin-2-yl)benzyl-4-(*N*,*N*-dipropylsulfamoyl)benzoate tetrafluoroborate (**2ad**, 81.0 mg, 0.100 mmol, 1.00 equiv), and obtained as a 1:1 mixture of two diastereomers as a yellow liquid (31.6 mg, 59% yield). ^{1}H NMR (400 MHz, $CDCl_3$): δ 8.17 (dd, *J* = 8.5, 1.9 Hz, 2H), 7.98–7.77 (m, 2H), 7.46–7.18 (m, 9H), 5.36 (s, 2H), 4.90 (dd, *J* = 8.9, 3.3 Hz, 1H), 3.35–2.95 (m, 4H), 2.85 (dd, *J* = 11.9, 9.0 Hz, 1H), 2.67–2.36 (m, 2H), 1.94 (d, *J* = 3.3 Hz, 1H), 1.79 (dd, *J* = 6.6, 1.7 Hz, 1H), 1.54 (m, 4H), 0.86 (m, 6H)·. ^{1}H NMR (400 MHz, $CDCl_3$): δ 8.24–8.11 (m, 2H), 7.86 (dt, *J* = 8.6, 2.0 Hz, 2H), 7.54–7.09 (m, 9H), 5.36 (s, 2H), 4.86 (dd, *J* = 9.1, 3.2 Hz, 1H), 3.29 (br, 1H), 3.18–3.02 (m, 4H), 2.95 (dd, *J* = 12.0, 9.2 Hz, 1H), 2.43 (m, 2H), 2.00 (d, *J* = 3.2 Hz, 1H), 1.86 (d, *J* = 6.6 Hz, 1H), 1.54 (m, 4H), 0.86 (t, *J* = 7.4 Hz, 6H). ^{13}C NMR (101 MHz, $CDCl_3$): δ 165.0, 144.3, 141.7, 140.2, 134.2, 133.4, 130.2, 128.5, 128.3, 127.6, 126.9, 126.4, 125.9, 72.9, 68.4, 67.1, 49.8, 41.0, 37.4, 21.8, 11.1. ^{13}C NMR (126 MHz, $CDCl_3$): δ 165.1, 144.3, 141.6, 140.4, 134.3, 133.4, 130.3, 128.6, 128.5, 128.4, 127.7, 127.0, 126.4, 126.0, 126.0, 73.2, 68.5, 68.1, 49.9, 40.8, 38.2, 21.9, 11.1. HRMS-ESI+: calculated for $C_{30}H_{37}N_2O_5S$ [M+H+] = 537.2418, observed [M+H+] = 537.2410.

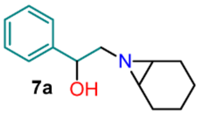

7a

2-(7-Azabicyclo[4.1.0]heptan-7-yl)-1-phenylethan-1-ol (7a) Prepared from styrene **4a** (17.0 μL, 0.150 mmol, 1.50 equiv) and 7-(2,4,6-triphenyl-pyridin-1-yl)-7-azabicyclo[4.1.0]heptane tetrafluoroborate **6** (49.0 mg, 0.100 mmol, 1.00 equiv), and obtained as a yellow liquid (9.5 mg, 42% yield). ^{1}H NMR (400 MHz, $CDCl_3$): δ ^{1}H NMR (400 MHz, $CDCl_3$) δ 7.65–7.17 (m, 5H), 4.79 (dd, *J* = 9.0, 3.6 Hz, 1H), 3.69 (br, 1H), 2.74 (dd, *J* = 11.9, 9.0 Hz, 1H), 2.19 (dd, *J* = 11.9, 3.6 Hz, 1H), 1.91–1.58 (m, 6H), 1.36 (m, 2H), 1.28–1.11 (m, 2H). ^{13}C NMR (101 MHz, $CDCl_3$): δ 142.1, 128.2, 127.3, 125.9, 72.7, 68.1, 38.4, 38.1, 24.5, 24.2, 20.4, 20.3. HRMS-ESI+: calculated for $C_{14}H_{20}NO$ [M+H+] = 218.1539, observed [M+H+] = 218.1534.

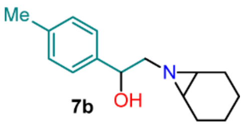

7b

2-(7-Azabicyclo[4.1.0]heptan-7-yl)-1-(p-tolyl)ethan-1-ol (7b) Prepared from 1-methyl-4-vinylbenzene **4b** (20.0 µL, 0.150 mmol, 1.50 equiv) and 7-(2,4,6-triphenyl-pyridin-1-yl)-7-azabicyclo[4.1.0]heptane tetrafluoroborate **6** (49.0 mg, 0.100 mmol, 1.00 equiv), and obtained as a yellow liquid (8.0 mg, 33% yield). ^{1}H NMR (400 MHz, $CDCl_3$): δ 7.49–7.20 (m, 2H), 7.14 (d, J = 7.8 Hz, 2H), 4.76 (dd, J = 9.2, 3.5 Hz, 1H), 3.63 (br, s), 2.74 (dd, J = 11.8, 9.2 Hz, 1H), 2.33 (s, 3H), 2.15 (dd, J = 11.9, 3.6 Hz, 1H), 1.84–1.67 (m, 4H), 1.66–1.56 (m, 2H), 1.36 (m, 2H), 1.24–1.07 (m, 2H). ^{13}C NMR (126 MHz, $CDCl_3$): δ 139.1, 137.0, 128.9, 125.8, 72.6, 68.1, 38.3, 38.1, 24.5, 24.2, 21.1, 20.4, 20.3. HRMS-ESI$^+$: calculated for $C_{15}H_{22}NO$ [M+H$^+$] = 232.1696, observed [M+H$^+$] = 232.1693.

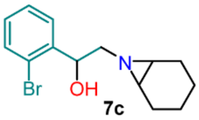

7c

2-(7-Azabicyclo[4.1.0]heptan-7-yl)-1-(2-bromophenyl)ethan-1-ol (7c) Prepared from 1-bromo-2-vinylbenzene **4f** (19.0 µL, 0.150 mmol, 1.50 equiv) and 7-(2,4,6-triphenyl-pyridin-1-yl)-7-azabicyclo[4.1.0]heptane tetrafluoroborate **6** (49.0 mg, 0.100 mmol, 1.00 equiv), and obtained as a yellow liquid (14.2 mg, 48% yield). ^{1}H NMR (400 MHz, $CDCl_3$): δ 7.66 (dd, J = 7.7, 1.7 Hz, 1H), 7.47 (dd, J = 7.9, 1.2 Hz, 1H), 7.33 (m, 1H), 7.11 (m, 1H), 5.09 (dd, J = 8.4, 3.2 Hz, 1H), 4.03 (br, 1H), 2.62 (dd, J = 11.9, 8.4 Hz, 1H), 2.34 (dd, J = 11.9, 3.3 Hz, 1H), 1.90–1.60 (m, 5H), 1.56 (m, 1H), 1.36 (m, 2H), 1.20 (m, 2H).^{13}C NMR (126 MHz, $CDCl_3$): δ 141.0, 132.3, 128.6, 127.7, 127.5, 121.5, 71.4, 65.6, 38.7, 37.8, 24.5, 24.1, 20.4, 20.3. HRMS-ESI$^+$: calculated for $C_{14}H_{19}BrNO$ [M+H$^+$] = 296.0645, observed [M+H$^+$] = 296.0642.

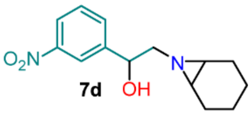

7d

2-(7-Azabicyclo[4.1.0]heptan-7-yl)-1-(3-nitrophenyl)ethan-1-ol (7d) Prepared from 1-nitro-3-vinylbenzene **4j** (19.0 µL, 0.150 mmol, 1.50 equiv) and 7-(2,4,6-triphenyl-pyridin-1-yl)-7-azabicyclo[4.1.0]heptane tetrafluoroborate **6** (49.0 mg, 0.100 mmol, 1.00 equiv), and obtained as an off white solid (9.2 mg, 35% yield). ^{1}H NMR (400 MHz, $CDCl_3$): δ 8.24 (t, J = 2.0 Hz, 1H), 8.12 (m, 1H), 7.76–7.66 (m, 1H), 7.51 (t, J = 7.9 Hz, 1H), 4.90 (dd, J = 8.6, 3.7 Hz, 1H), 2.68 (dd, J = 11.9, 8.6 Hz, 1H), 2.35 (dd, J = 11.9, 3.8 Hz, 1H), 1.86–1.62 (m, 6H), 1.36 (m, 2H), 1.24–1.13 (m, 2H).^{13}C NMR (126 MHz, $CDCl_3$): δ 148.2, 144.5, 132.0, 129.2, 122.4, 120.9, 71.5, 67.3, 39.0, 38.5, 24.2, 23.9, 20.2, 20.1. HRMS-ESI$^+$: calculated for $C_{14}H_{19}N_2O_3$ [M+H$^+$] = 263.1390, observed [M+H$^+$] = 263.1387.

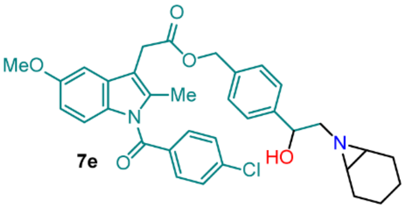

7e

4-((1S)-2-(7-azabicyclo[4.1.0]heptan-7-yl)-1-hydroxyethyl)benzyl 2-(1-(4-chlorobenzoyl)-5-methoxy-2-methyl-1H-indol-3-yl)acetate (7e) Prepared from 4-vinylbenzyl 2-(1-(4-chlorobenzoyl)-5-methoxy-2-methyl-1H-indol-3-yl)acetate **4p** (71.0 mg, 0.150 mmol, 1.50 equiv) and 7-(2,4,6-triphenyl-pyridin-1-yl)-7-azabicyclo[4.1.0]heptane tetrafluoroborate **6** (49.0 mg, 0.100 mmol, 1.00 equiv), and obtained as an off white solid (11.0 mg, 18% yield). ^{1}H NMR (400 MHz, $CDCl_3$): δ 7.64 (d, *J* = 8.1 Hz, 2H), 7.46 (d, *J* = 8.2 Hz, 2H), 7.32 (d, *J* = 8.1 Hz, 1H), 7.28–7.21 (m, 2H), 7.06–6.77 (m, 2H), 6.66 (dd, *J* = 9.0, 2.6 Hz, 2H), 5.12 (s, 2H), 4.90 (d, *J* = 9.1 Hz, 1H), 3.76 (s, 3H), 3.70 (s, 2H), 2.67 (dd, *J* = 12.1, 9.2 Hz, 1H), 2.35 (s, 3H), 1.98–1.68 (m, 7H), 1.39 (dd, *J* = 13.5, 7.3 Hz, 2H), 1.23–1.07 (m, 2H). ^{13}C NMR (126 MHz, $CDCl_3$): δ 170.6, 168.2, 156.0, 142.4, 139.2, 135.9, 134.8, 133.8, 131.1, 130.7, 130.5, 129.1, 128.1, 126.1, 114.9, 112.4, 111.8, 101.1, 72.3, 68.0, 66.5, 55.6, 38.4, 38.2, 30.4, 24.5, 24.2, 20.4, 20.3, 13.3. HRMS-ESI$^+$: calculated for $C_{34}H_{36}ClN_2O_5$ [M+H$^+$] = 587.2307, observed [M+H$^+$] = 587.2298.

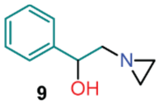

9

2-(Aziridin-1-yl)-1-phenylethan-1-ol (9) Prepared from styrene **4a** (17.0 µL, 1.50 equiv, 0.150 mmol) and 1-(aziridin-1-yl)-2,4,6-triphenylpyridin-1-ium trifluoromethanesulfonate **8** (50.0 mg, 0.100 mmol, 1.00 equiv), and obtained as a yellowish liquid (5.2 mg, 25% yield). ^{1}H NMR (400 MHz, $CDCl_3$): δ 7.65–7.01 (m, 5H), 4.85 (dd, *J* = 9.1, 3.4 Hz, 1H), 2.75 (dd, *J* = 11.9, 9.1 Hz, 1H), 2.22–2.12 (m, 1H), 1.80 (m, 2H), 1.33–1.16 (m, 2H).HRMS-ESI$^+$: calculated for $C_{10}H_{14}NO$ [M+H$^+$] = 164.1070, observed [M+H$^+$] = 164.1069. The obtained spectral data are in good agreement with those reported in literature.[6]

C.2 Optimization Studies for Photocatalyzed Olefin Hydroxyaziridination

Table S1. Impact of photocatalyst structure on photocatalytic hydroxyaziridination of styrene.

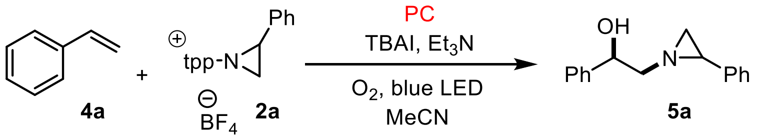

entry	*PC*	*NMR yield*
1	$(Ir[dF(CF_3)ppy]_2(dtbpy))PF_6$	15%
2	$[Ir(dtbbpy)(ppy)_2]PF_6$	26%
3	$Ir(ppy)_3$	35%
4	$Ru(bpy)_3Cl_2$. $6H_2O$	16%
5	4-CzIPN	29%
6	Eosin Y	27%

Table S2. Impact of solvent on photocatalytic hydroxyaziridination of styrene.

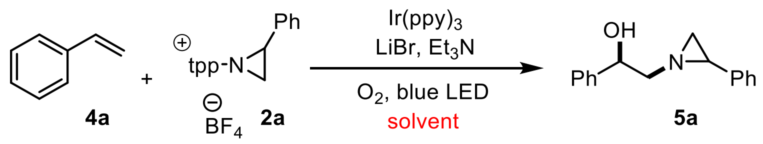

entry	*solvent*	*NMR yield*
1	DCE	26%
2	THF	22%
3	DMF	14%
4	DMA	19%
5	CH_3CN:H_2O (1:1)	35%
6	MeOH	trace

Table S3. Impact of base on photocatalytic hydroxyaziridination of styrene.

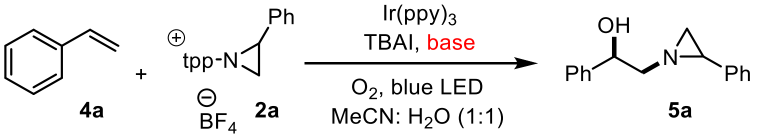

4a + 2a → 5a

entry	*base*	*NMR yield*
1	hunig base	23%
2	DABCO	trace
3	DBU	28%
4	Ph_3N	10%
5	Et_3N	35%

Table S4. Impact of additives on photocatalytic hydroxyaziridination of styrene.

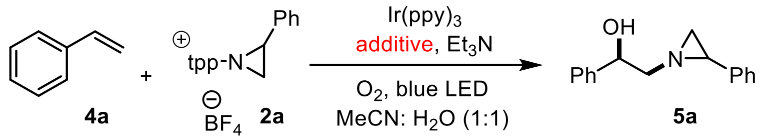

entry	*additive*	*NMR yield*
1	TBAI	35%
2	NaI	52%
3	PIDA	49%
4	$LiBF_4$	47%
5	LiOTf	52%
6	LiBr	65%
7[a]	LiBr	42%
8	TBABr	36%
9	NaBr	47%
10	KBr	40%

[a]50 mol% LiBr was used.

Table S5. Impact of Lewis acids on hydroxyaziridination of styrene.

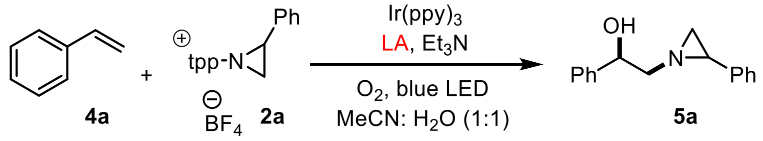

entry	*Lewis acid*	*NMR yield*
1	$Sc(OTf)_3$	0%
2	$La(OTf)_3$	0%
3	$Bi(OTf)_3$	~10%
4	$BF_3.OEt_2$	0%

For all the cases water mediated ring opening of the starting aziridine salt **2a** has been found in the HRMS.

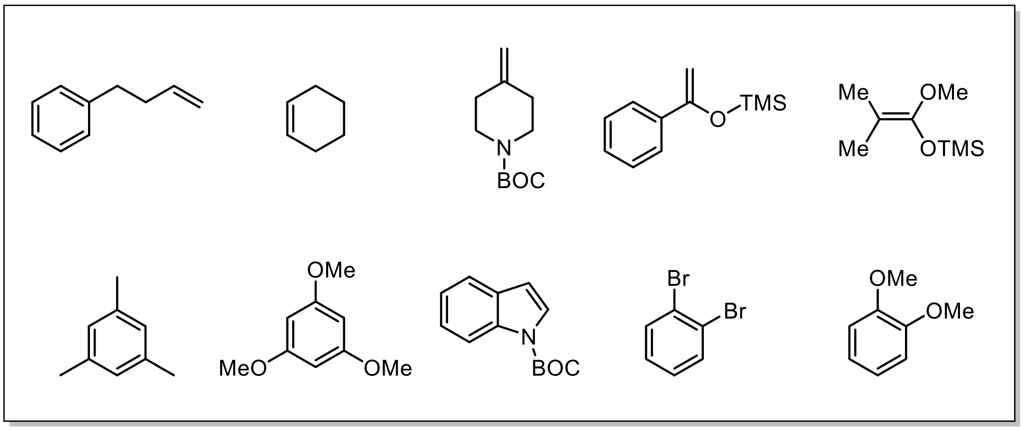

Figure S1. List of unsuccessful olefins and arenes that failed to deliver the azirinated products under the optimized condition of styrene hydroxyazirination. Reactions were carried out with the reaction conditions optimized for the hydroxyaziridination of styrene. These reactions were completely unproductive.

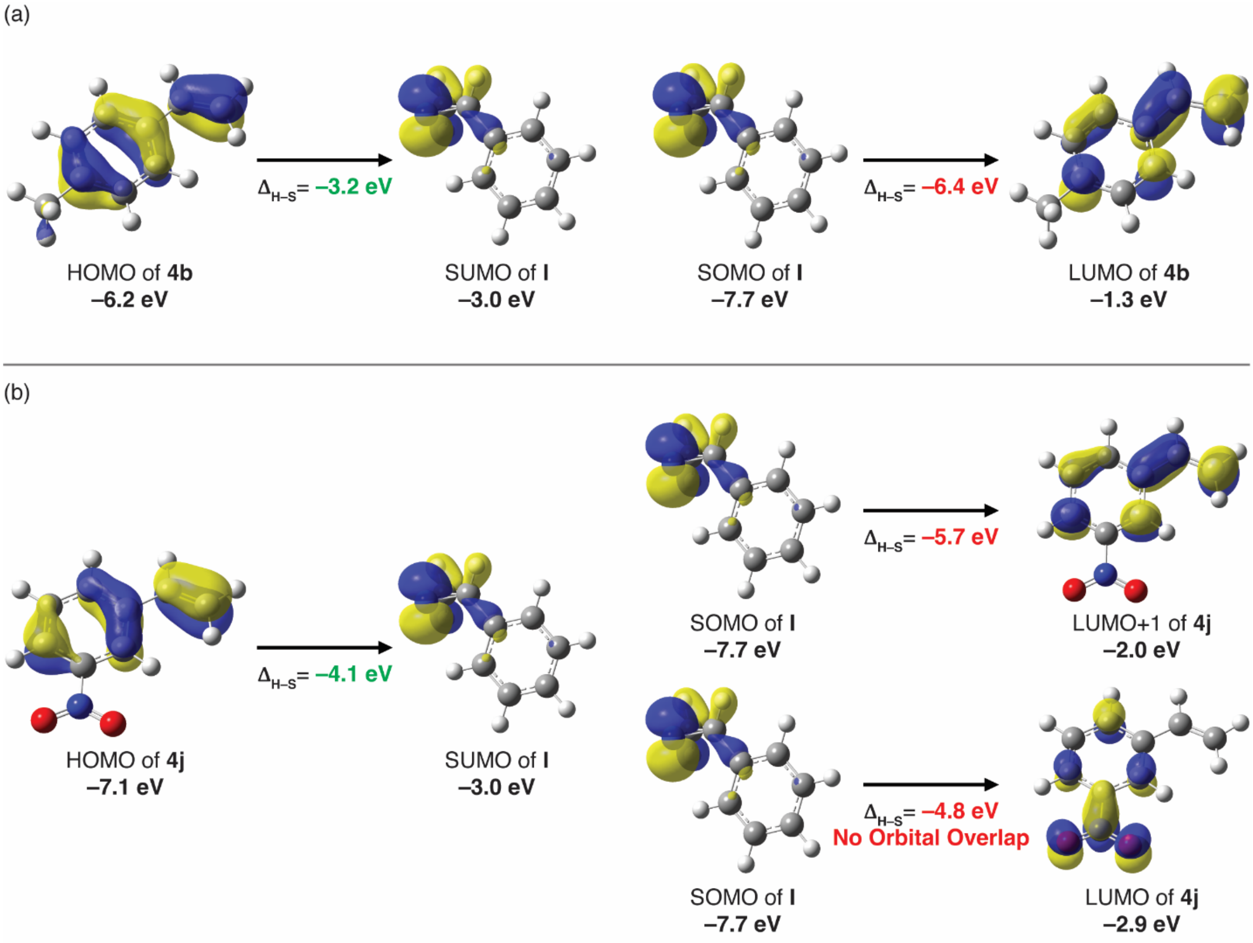

Figure S2. (a) orbital overlap between **4b** and aziridinyl radical I where **I** is the electrophile (left) vs. nucleophile (right). (b) orbital overlap between **4j** and aziridinyl radical **I** where **I** is the electrophile (left) vs. nucleophile (top right). The LUMO of **4j** does not show C=C π-character (bottom right).

D. Mechanistic Investigations

D.1. Examination of Radical Acceptors

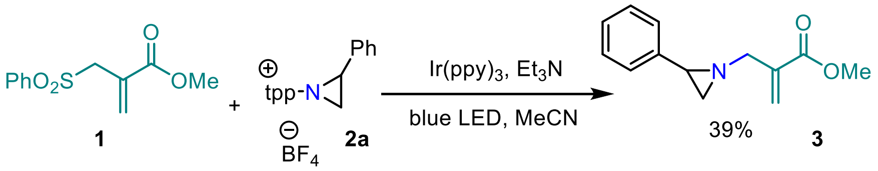

A 40-mL scintillation vial was charged with radical acceptor[7] **1** (24.0 mg, 0.100 mmol, 1.00 equiv), triphenylpyridinium aziridine **2a** (51.0 mg, 0.100 mmol, 1.00 equiv), and Ir(ppy)$_3$ (0.7 mg, 0.001 mmol, 1.00 mol%). In an N_2-filled dry box, the solid compounds were dissolved in MeCN (1 mL). Into that reaction mixture, Et_3N (28.0 µL, 0.200 mmol, 2.00 equiv) was added. With stirring, the reaction was irradiated with blue LED lights for 12 h; the temperature was maintained at 23 °C using air cooling provided by a fan. The reaction mixture was diluted with distilled water (5.0 mL) and extracted with ethyl acetate (3 × 5.0 mL). The combined organic layer was washed with brine, dried over anhydrous Na_2SO_4, and concentrated under reduced pressure. The crude product was purified by column chromatography (hexanes : EtOAc 70:30) to afford the desired aminated olefin **3** *via* radical addition elimination protocol.

^{1}H NMR (400 MHz, $CDCl_3$): δ 7.33–7.05 (m, 5H), 6.20 (d, *J* = 1.6 Hz, 1H), 5.86 (q, *J* = 1.9 Hz, 1H), 3.68 (s, 3H), 3.45 (dt, *J* = 16.5, 1.8 Hz, 1H), 3.04 (dt, *J* = 16.5, 1.8 Hz, 1H), 2.32 (dd, *J* = 6.5, 3.4 Hz, 1H), 1.93 (d, *J* = 3.4 Hz, 1H), 1.75 (d, *J* = 6.6 Hz, 1H). ^{13}C NMR (101 MHz, $CDCl_3$) δ 166.7, 139.9, 137.7, 128.3, 126.9, 126.0, 125.8, 60.7, 51.8, 41.6, 38.0. HRMS-ESI$^+$: calculated for $C_{13}H_{16}NO_2$ [M+H$^+$] = 218.1176, observed [M+H$^+$] = 218.1174.

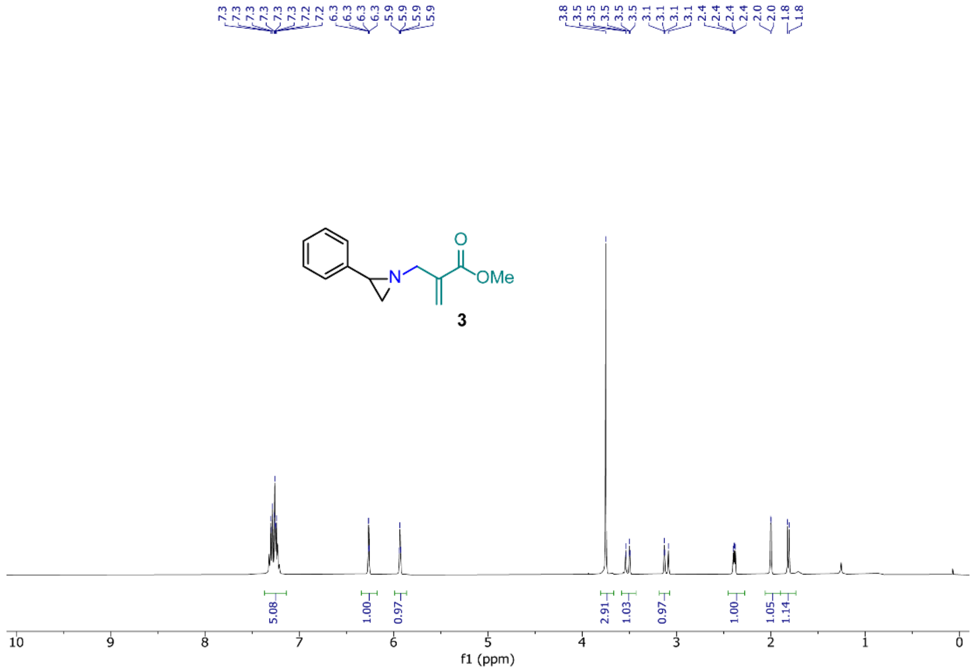

Figure S3. ^{1}H NMR spectrum of addition elimination product **3** in $CDCl_3$ (400 MHz) at 23 °C.

D.2. Spin-Trapping Experiments

A 25-mL Schlenk tube was charged with triphenylpyridinium aziridine (**2a**, 51.0 mg, 0.100 mmol, 1.00 equiv), Ir(ppy)$_3$ (0.7 mg, 0.001 mmol, 1.00 mol%), and *N*-tert-butyl-a-phenylnitrone (PBN, 21.2 mg, 0.120 mmol, 1.20 equiv). In an N_2-filled dry box, the solid compounds were dissolved in MeCN (1.0 mL) and Et_3N (28.0 µL, 0.200 mmol, 2.00 equiv) was added. The resulting solution was irradiated for 2 h with a blue LED. An aliquot of the resulting solution (50 µL) was transferred to a 2 mm EPR tube and the EPR spectrum was collected (pictured below).

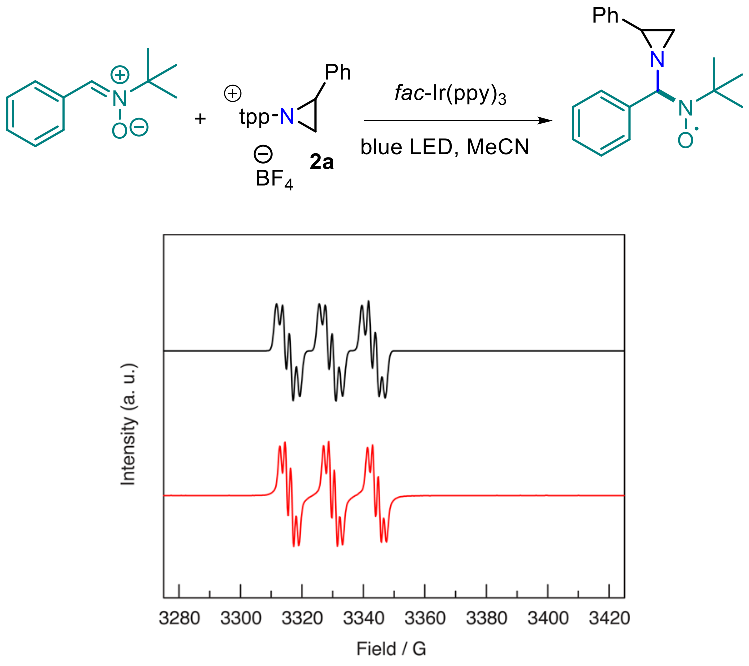

Figure S4. EPR spectra for photochemically generated aziridinyl radical in the presence of PBN was obtained in CH_3CN : (—) Experimental spectrum and (—) simulated spectrum. The observed triplet of quartet in the photolyzed samples is attributed to PBN-trapped aziridnyl radical with $a_{N(PBN)}$ = 14.0 G, a_H = 1.8 G, and $a_{N(aziridinyl)}$ = 2.1 G. The apparent triplet of quartet is due to the unresolved/overlapped hyperfine couplings from a_H and $a_{N(aziridinyl)}$. Formation of PBN-trapped aziridinyl radical was further confirmed by mass analysis of the EPR sample where HRMS-ESI: calculated for $C_{19}H_{23}N_2O$ $[M+H]^+$ = 295.1805, observed $[M+H]^+$ = 295.1797.

D.3. Radical Inhibition Experiments

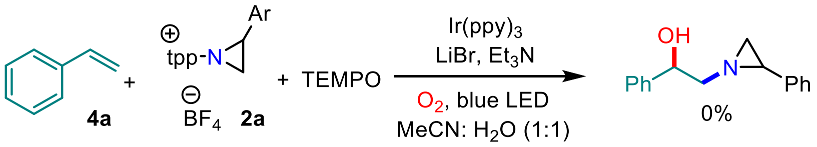

A 40-mL scintillation vial was charged with triphenylpyridinium aziridine (**2a**, 51.0 mg, 0.100 mmol, 1.00 equiv), Ir(ppy)$_3$ (0.7 mg, 0.001 mmol, 1.0 mol%), LiBr (8.7 mg, 0.100 mmol, 1.00 equiv) and TEMPO (31.2 mg, 0.200 mmol, 2.00 equiv). The solid compounds were dissolved in a MeCN:H_2O (1:1, 2 mL). Into that reaction mixture, styrene (**4a**, 17.0 µL, 0.150 mmol, 1.50 equiv) and Et_3N (28.0 µL, 0.200 mmol, 2.00 equiv) were added. The crude reaction mixture was analysed by ^{1}HNMR and HRMS. The analysis of the crude reaction mixture revealed no desired product **5a** formation.

D.4. Radical Clock Experiments

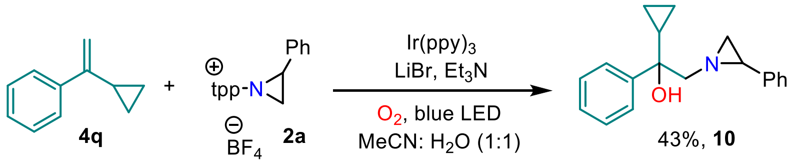

A 40-mL scintillation vial was charged with triphenylpyridinium aziridine (**2a**, 51.0 mg, 0.100 mmol, 1.00 equiv), Ir(ppy)$_3$ (0.7 mg, 0.001 mmol, 1.00 mol%) and LiBr (8.6 mg, 0.100 mmol, 1.00 equiv). The solid compounds were dissolved in a MeCN:H_2O (2 mL). Into that reaction mixture, (1-cyclopropylvinyl)benzene (**4q**)[8] (21.6 mg, 0.150 mmol, 1.50 equiv) and Et_3N (28.0 µL, 0.200 mmol, 2.00 equiv) were added. The reaction mixture was purged with oxygen. With stirring, the reaction was irradiated with blue LED lights for 12 h; the temperature was maintained at 23 °C using air cooling provided by a fan. The reaction mixture was diluted with distilled water (5.0 mL) and extracted with ethyl acetate (3 × 5.0 mL). The combined organic layer was washed with brine, dried over anhydrous Na_2SO_4, and concentrated under reduced pressure. The crude product was purified by column chromatography (hexanes : EtOAc 85:15) and hydroxyazirinated product **10** was observed with 1:1 diastereomeric mixture as major product with isolated yield of 43% where cyclopropyl ring remain intact.

^{1}H NMR (400 MHz, $CDCl_3$) δ: 7.32 (d, J = 7.5 Hz, 2H), 7.21–6.93 (m, 16H), 6.79–6.50 (m, 2H), 3.49 (s, 1H), 3.45 (s, 1H), 2.76 (d, J = 11.8 Hz, 1H), 2.69 (d, J = 2.7 Hz, 2H), 2.61 (d, J = 11.7 Hz, 1H), 2.34 (dd, J = 6.4, 3.4 Hz, 1H), 2.01 (dd, J = 6.5, 3.5 Hz, 1H), 1.77 (d, J = 3.5 Hz, 1H), 1.66 (d, J = 6.5 Hz, 1H), 1.52 (d, J = 3.4 Hz, 1H), 1.41 (d, J = 6.5 Hz, 1H), 1.17–0.93 (m, 2H), 0.47 (dd, J = 9.8, 4.8 Hz, 1H), 0.44–0.27 (m, 1H), 0.27–0.19 (m, 2H), 0.17–0.00 (m, 4H). ^{13}C NMR (101 MHz, $CDCl_3$) δ: 146.2, 146.1, 139.9, 139.5, 128.2, 127.9, 127.8, 127.8, 126.9, 126.8, 126.6, 126.5, 125.9, 125.8, 125.3, 73.2, 73.1, 71.1, 70.9, 41.6, 41.5, 38.3, 37.8, 19.7, 1.6, 1.5, 0.2, -0.11. HRMS-ESI$^+$: calculated for $C_{19}H_{22}NO$ [M+H$^+$] = 280.1696, observed [M+H$^+$] = 280.1691.

The same reaction was repeated under inert atmosphere where cyclopropyl ring opened product **11** was found with 26% yield.[9] This result proves that the reaction proceeds via aziridinyl radical. Product **10** was majorly observed under oxygen atmosphere may be due to the higher rate of O_2 addition by benzylic radical over the cyclopropyl ring opening.

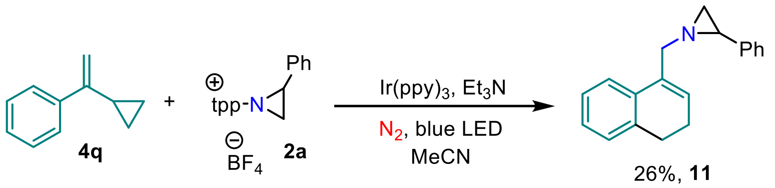

^{1}H NMR (400 MHz, $CDCl_3$) δ: 7.42–7.30 (m, 1H), 7.33 – 7.14 (m, 6H), 7.13–7.00 (m, 2H), 6.11 (td, J = 4.5, 2.2 Hz, 1H), 3.41 (dq, J = 5.4, 1.8 Hz, 2H), 2.73 (t, J = 8.0 Hz, 2H), 2.43 (dd, J = 6.5, 3.4 Hz, 1H), 2.33–2.16 (m, 2H), 1.95 (d, J = 3.4 Hz, 1H), 1.79 (d, J = 6.5 Hz, 1H). ^{13}C NMR (101 MHz, $CDCl_3$) δ: 140.4, 136.3, 134.2, 133.6, 128.6, 128.2, 127.5, 126.7, 126.3,

126.1, 126.0, 122.5, 62.7, 41.5, 38.2, 28.0, 23.0. HRMS-ESI+: calculated for $C_{19}H_{20}N$ [M+H+] = 262.1590, observed [M+H+] = 262.1586.

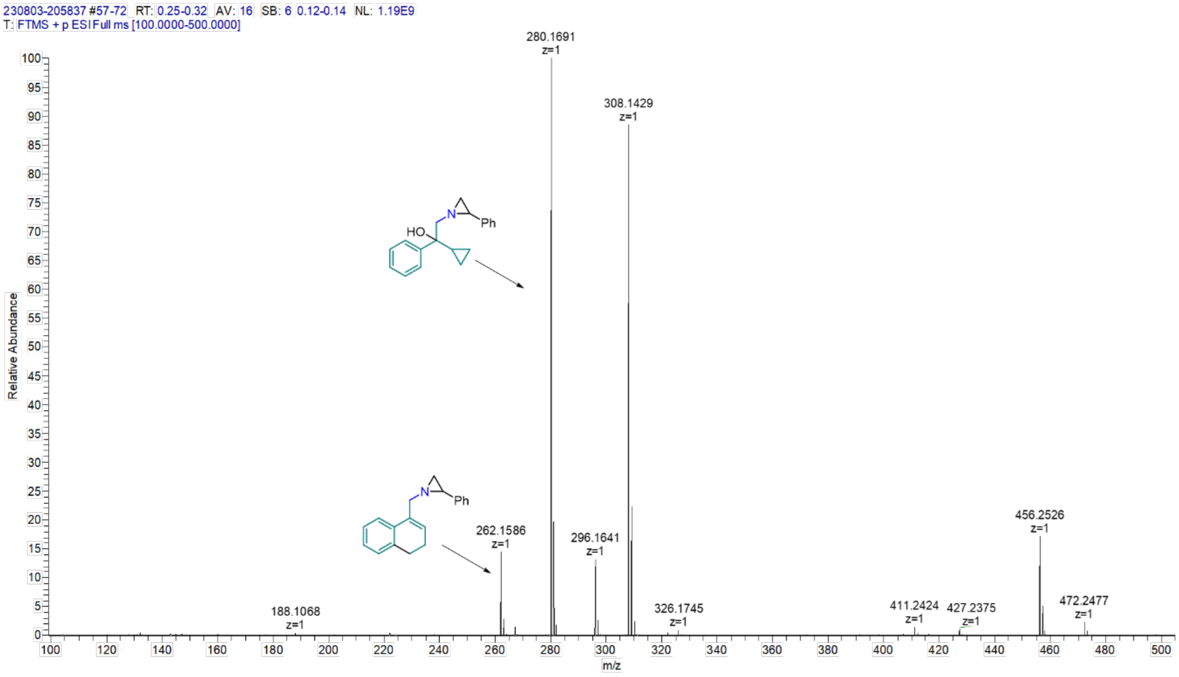

Figure S5. HR-MS data for radical clock reaction under oxygen environment. Both the hydroxy-azirinated **10** and ring opened product **11** were observed.

D.5. Stern-Volmer Quenching Experiments

We performed fluorescence quenching experiments to identify the primary quencher of the excited photocatalyst.[10] A glass cuvette with a B-14 joint and screw cap was used. A solution of Ir(ppy)$_3$ in anhydrous CH_2Cl_2 (8.4 × 10^{-5} M) was prepared. An aliquot (2.0 mL) of the stock solution was taken in the glass cuvette and purged with N_2. Samples were excited at 360 nm and the emission was collected at 515 nm. The Stern-Volmer analysis was conducted according to the following relationship:

$$I_0/I = 1 + [Q]$$

where, I_0 = fluorescence intensity of photocatalyst (PC) in absence of quencher; I = fluorescence intensity of PC in presence of quencher; [Q] = concentration of quencher; Ksv = quenching rate constant

The fluorescence quenching study was performed using pyridinium aziridine **2a**, triethylamine as quenchers independently. With increasing concentration of pyridinium salt **2a**, an appreciable decrease in the fluorescence intensity of PC was observed, however only a slight decrease in fluorescence intensity was observed upon addition of triethylamine.

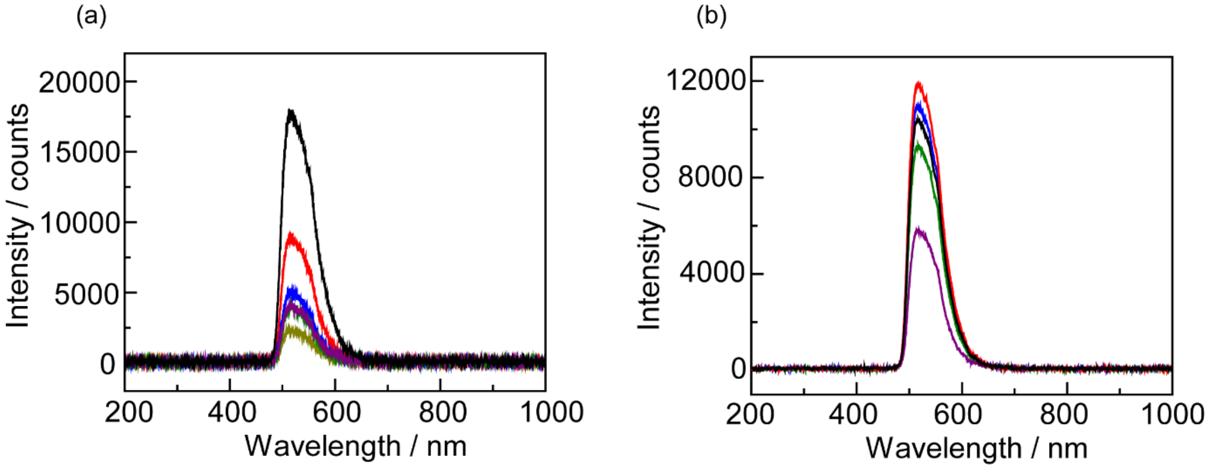

Figure S6. (a) Emission spectrum of solution of Ir(ppy)$_3$ (8.4 × 10^{-5} M) with different concentrations of **2a** in MeCN under N_2 atmosphere at 20 °C. (—) 0.0 mM, (—) 0.69 mM, (—) 1.03 mM, (—) 1.30 mM, (—) 2.07 mM, and (—) 2.70 mM. (b) Emission spectrum of solution of PC (8.4 × 10^{-5} M) with different concentrations of Et_3N in MeCN under N_2 atmosphere at 20 °C. (—) 0.0 mM, (—) 0.69 mM, (—) 1.03 mM, (—) 1.30 mM, and (—) 2.70 mM.

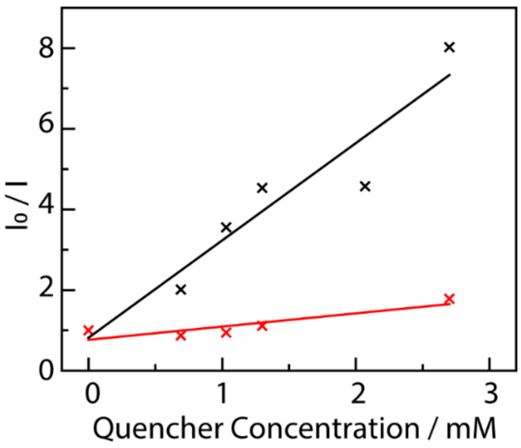

Figure S7. Stern-Volmer plot in the quenching study of PC using **2a** or Et_3N in CH_2Cl_2 under N_2 atmosphere at 20 °C with emission wavelength fixed at 515 nm. Plot of emission intensity ratio using **2a** (—) and liner fit (×) *vs.* **2a** concentration (R^2 = 0.91); plot of emission intensity ratio using Et_3N (—) and linear fit (×) *vs.* Et_3N concentration (R^2 = 0.78).

D.6. Cyclic Voltammetry of *N*-pyridinium Aziridine 2a

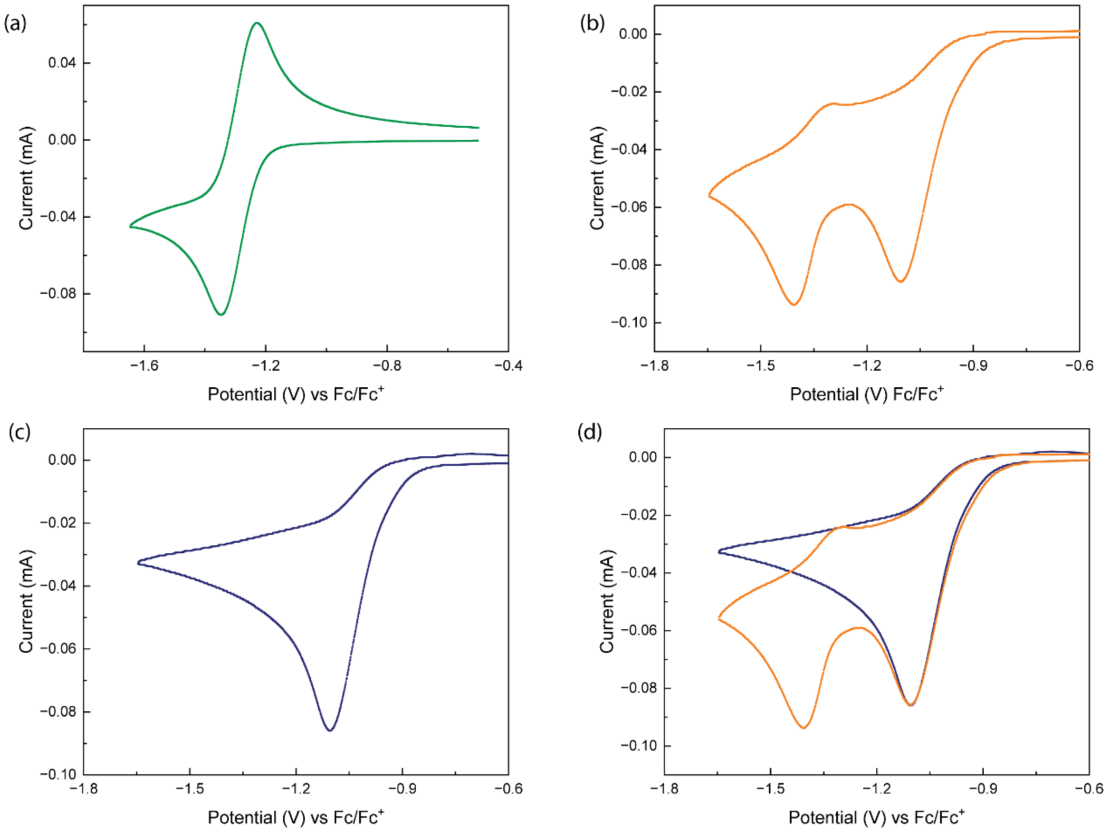

Figure S8. (a) Cyclic voltammogram of dissolved molecular oxygen in MeCN (b) Cyclic voltammogram of 2,4,6-triphenyl-1-(2-phenylaziridin-1-yl)pyridin-1-ium (**2a**) under ambient conditions. (c) Cyclic voltammogram of 2,4,6-triphenyl-1-(2-phenylaziridin-1-yl)pyridin-1-ium (**2a**) under a nitrogen atmosphere. (d) Overlay of the CV data from parts (b) and (c). CV conditions: substrate 5.00 mM, 0.200 M [TBA]PF_6 solution of CH_3CN, glassy carbon working electrode, Pt counter electrode, and scan rate = 0.10 V/s.

D.7. Isotope Labelling Experiments

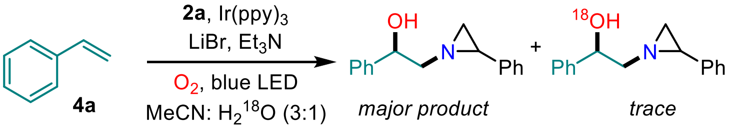

A 40-mL scintillation vial was charged with triphenylpyridinium aziridine (**2a**, 51.0 mg, 0.100 mmol, 1.00 equiv), Ir(ppy)$_3$ (0.7 mg, 0.001 mmol, 1.00 mol%) and LiBr (8.6 mg, 0.100 mmol, 1.00 equiv). The solid compounds were dissolved in a MeCN:H_2O (3:1, 0.400 mL). Into that reaction mixture, styrene (**4a**) (51.0 mg, 0.150 mmol, 1.50 equiv) and Et$_3$N (28.0 µL, 0.200 mmol, 2.00 equiv) were added. The reaction mixture was purged with oxygen. With stirring, the reaction was irradiated with blue LED lights for 12 h; the temperature was maintained at 23 °C using air cooling provided by a fan. HRMS data of the crude mixture show ^{16}OH product is the major product, justify molecular oxygen to be the major hydroxyl source.

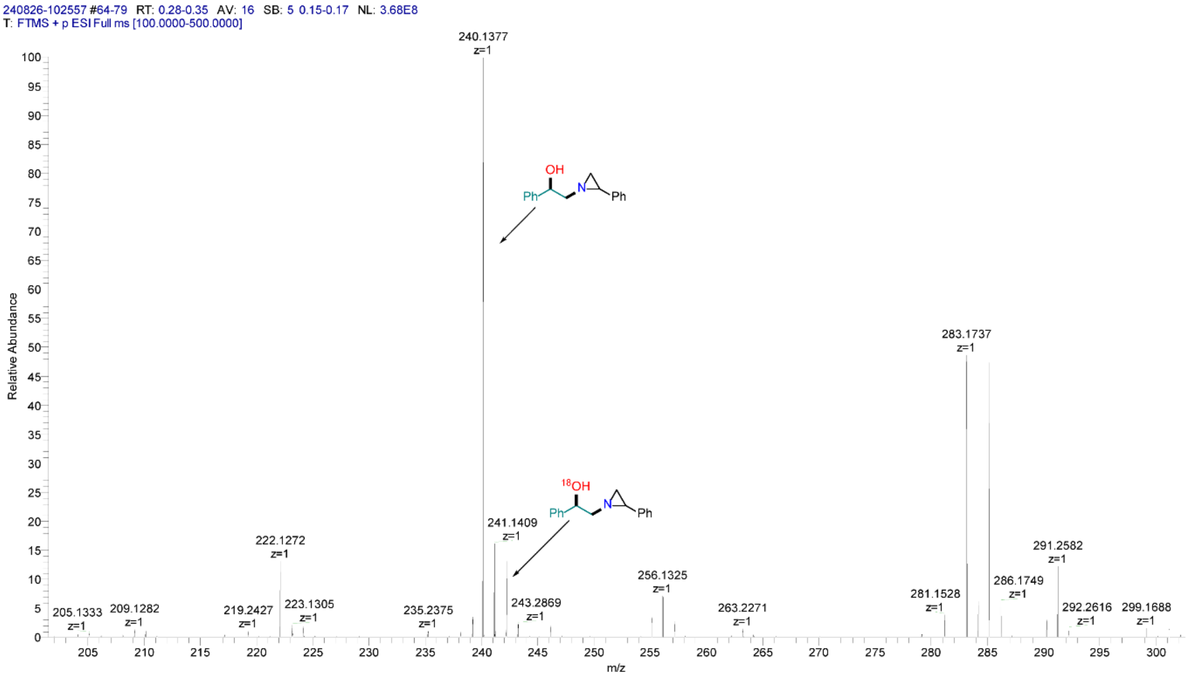

Figure S9. HR-MS data for isotope labelling experiment performed using $H_2{}^{18}O$.

D.8. Experiments to Check the Potential Role of Oxygenated Intermediates

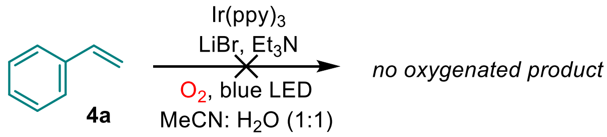

A 40-mL scintillation vial was charged with Ir(ppy)$_3$ (0.7 mg, 0.001 mmol, 1 mol%), and LiBr (8.70 mg, 0.100 mmol, 1.00 equiv). The solid compounds were dissolved in a 1:1 mixture of MeCN:H_2O (2 mL total). Triethylamine (28.0 µL, 0.200 mmol, 2.00 equiv) and styrene **4a** (17.0 µL, 0.150 mmol, 1.50 equiv) were added. The reaction mixture was purged with oxygen and stirred under blue LED. The reaction was monitored for 3 h but periodic acquisition of HR-MS data. At no time were oxygenated products observed.

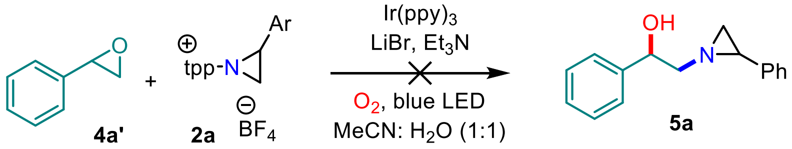

A 40-mL scintillation vial was charged with triphenylpyridinium aziridine **2a** (51.0 mg, 0.100 mmol, 1.00 equiv), Ir(ppy)$_3$ (0.7 mg, 0.001 mmol, 1 mol%), and LiBr (8.70 mg, 0.100 mmol, 1.00 equiv). The solid compounds were dissolved in a 1:1 mixture of MeCN:H_2O (2 mL total). Triethylamine (28.0 µL, 0.200 mmol, 2.00 equiv) and styrene oxide (**4a'**) (17.0 µL, 0.150 mmol, 1.50 equiv) were added. The reaction mixture was purged with oxygen and stirred under blue LED. No products of aziridine addition were detected following photolysis; only N–H aziridine and unreacted epoxide were observed.

E. Optimized Coordinates

All computations were carried out using Revision C.01 of Gaussian 16 suite of programs,[11] All geometries were optimized in the gas phase using the B3LYP [12, 13] functional in conjunction with Grimme's D3 empirical dispersion[14] and Becke-Johnson damping[15] and the basis set of 6–31G(d').[16] All minima were confirmed by analytical frequency computations. For the calculation of the electrophilicity parameter, ω, and orbital energies, single point energy corrections were performed over the optimized geometries using the B3LYP functional and the 6-311+G(d,p) [17,18] basis set [B3LYP-D3BJ/6-311G+(d,p)//B3LYP-D3BJ/6-31G(d')]. All orbital images were generated using GaussView6[19] with an isovalue of 0.05.

Table S6. Optimized Coordinates of azirdinyl radical generated from **9.**

C	0.000000	0.739237	–0.345267
C	0.000000	–0.739237	–0.345267
H	0.917200	1.287029	–0.570635
H	–0.917200	1.287029	–0.570635
H	0.917200	–1.287029	–0.570635
H	–0.917200	–1.287029	–0.570635
N	0.000000	0.000000	0.917963

HF = –133.269218684

Table S7. Optimized Coordinates of aziridinyl radical **I**.

C	–2.654045	–0.115424	–0.683083
C	–1.633528	0.595358	0.129520
H	–3.545030	0.418505	–1.007343
H	–2.350789	–0.931080	–1.337493
H	–1.854351	1.627645	0.394710
N	–2.542576	–0.380495	0.739758
C	–0.182005	0.269071	0.071038
C	0.759958	1.300873	0.022086
C	0.265120	–1.057209	0.060704
C	2.120843	1.015640	–0.051282
H	0.424177	2.332227	0.039594
C	1.625575	–1.341337	–0.008587
H	–0.452794	–1.866768	0.123262
C	2.558371	–0.307031	–0.068033
H	2.839004	1.826497	–0.092061
H	1.958740	–2.372824	–0.010718
H	3.617348	–0.530375	–0.122432

HF= –364.448303082

Table S8. ω Parameter SCF Energies.

Aziridinyl Radical	SCF Energy
I	–364.4483031
I – e^{-}	–364.1121153
I + e^{-}	–364.4848994

F. X-Ray Diffraction Data

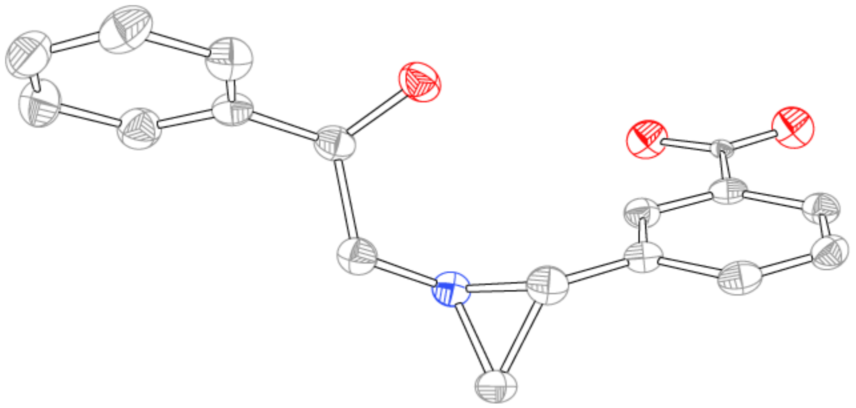

Figure S10. Displacement ellipsoid plot of **5v** plotted at 50% probability. H-atoms are removed for clarity. The crystalline sample used for the diffraction experiment was obtained via pentane diffusion into EtOAc.

Table S9. X-ray experimental details of **5v** (CCDC 2362719).

**Crystal data**	
Chemical formula	$C_{16}H_{16}N_2O_3$
M_r	284.31
Crystal system, space group	Monoclinic, $P2_1/c$
Temperature (K)	100
a, *b*, *c* (Å)	25.3664(5), 8.0897(2), 6.9110(2)
β (°)	96.433(2)
V (Å^3)	1409.25(6)
Z	4
Radiation type	Cu $K\alpha$
μ (mm^{-1})	0.77
Crystal size (mm)	0.18 × 0.03 × 0.01
**Data collection**	
Diffractometer	XtaLAB Synergy, Dualflex, HyPix
Absorption correction	Multi-scan<br>*CrysAlis PRO* 1.171.43.101a (Rigaku Oxford Diffraction, 2023) Empirical absorption correction using spherical harmonics, implemented in SCALE3 ABSPACK scaling algorithm.
T_{min}, T_{max}	0.780, 1.000
No. of measured, independent and observed [$I > 2\sigma(I)$] reflections	10366, 2204, 1996
R_{int}	0.029
(sin θ/λ)$_{max}$ (Å^{-1})	0.577
**Refinement**	
$R[F^2 > 2\sigma(F^2)]$, $wR(F^2)$, S	0.049, 0.120, 1.16
No. of reflections	2204
No. of parameters	191
H-atom treatment	H-atom parameters constrained
$\Delta\rho_{max}$, $\Delta\rho_{min}$ (e Å^{-3})	0.24, −0.24

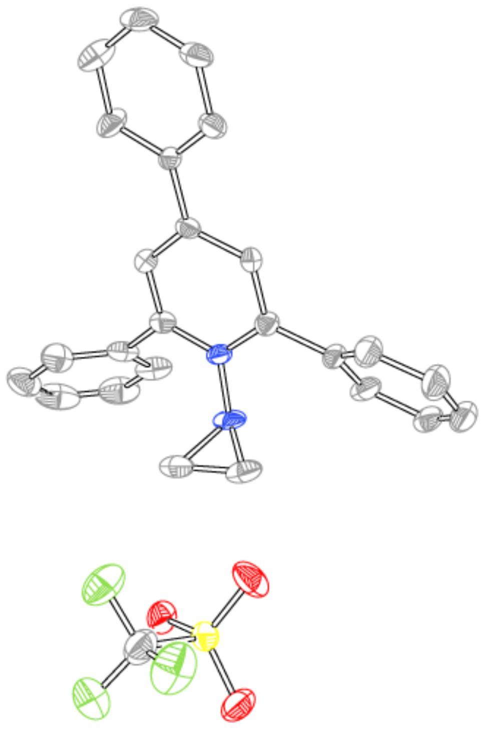

Figure S11. Displacement ellipsoid plot of **8** plotted at 50% probability. H-atoms are removed for clarity. The crystalline sample used for the diffraction experiment was obtained via diethylether diffusion into CH_3CN. For clarity only one molecule in the unit cell is depicted here.

Table S10. X-ray experimental details of **8** (CCDC 2366346).

**Crystal data**	
Chemical formula	$2(CF_3O_3S)\cdot 2(C_{25}H_{21}N_2)$
M_r	997.01
Crystal system, space group	Triclinic, *P*–1
Temperature (K)	110
a, *b*, *c* (Å)	10.876(1), 14.650(2), 14.927(2)
α, β, γ (°)	99.836(3), 100.193(4), 98.521(3)
V ($Å^3$)	2267.0(5)
Z	2
Radiation type	Mo *K*α
μ (mm^{-1})	0.20
Crystal size (mm)	0.3 × 0.24 × 0.15
**Data collection**	
Diffractometer	Bruker *APEX*-II CCD
Absorption correction	Multi-scan<br>SADABS2016/2 (Bruker,2016/2) was used for absorption
No. of measured, independent and observed [$I > 2\sigma(I)$] reflections	58688, 9246, 7132
R_{int}	0.050
$(\sin \theta/\lambda)_{max}$ ($Å^{-1}$)	0.627
**Refinement**	
$R[F^2 > 2\sigma(F^2)]$, $wR(F^2)$, S	0.093, 0.223, 1.10
No. of reflections	9246
No. of parameters	665
H-atom treatment	H-atom parameters constrained
$\Delta\rho_{max}$, $\Delta\rho_{min}$ (e $Å^{-3}$)	1.61, −0.93

G. NMR Spectra for New Compounds

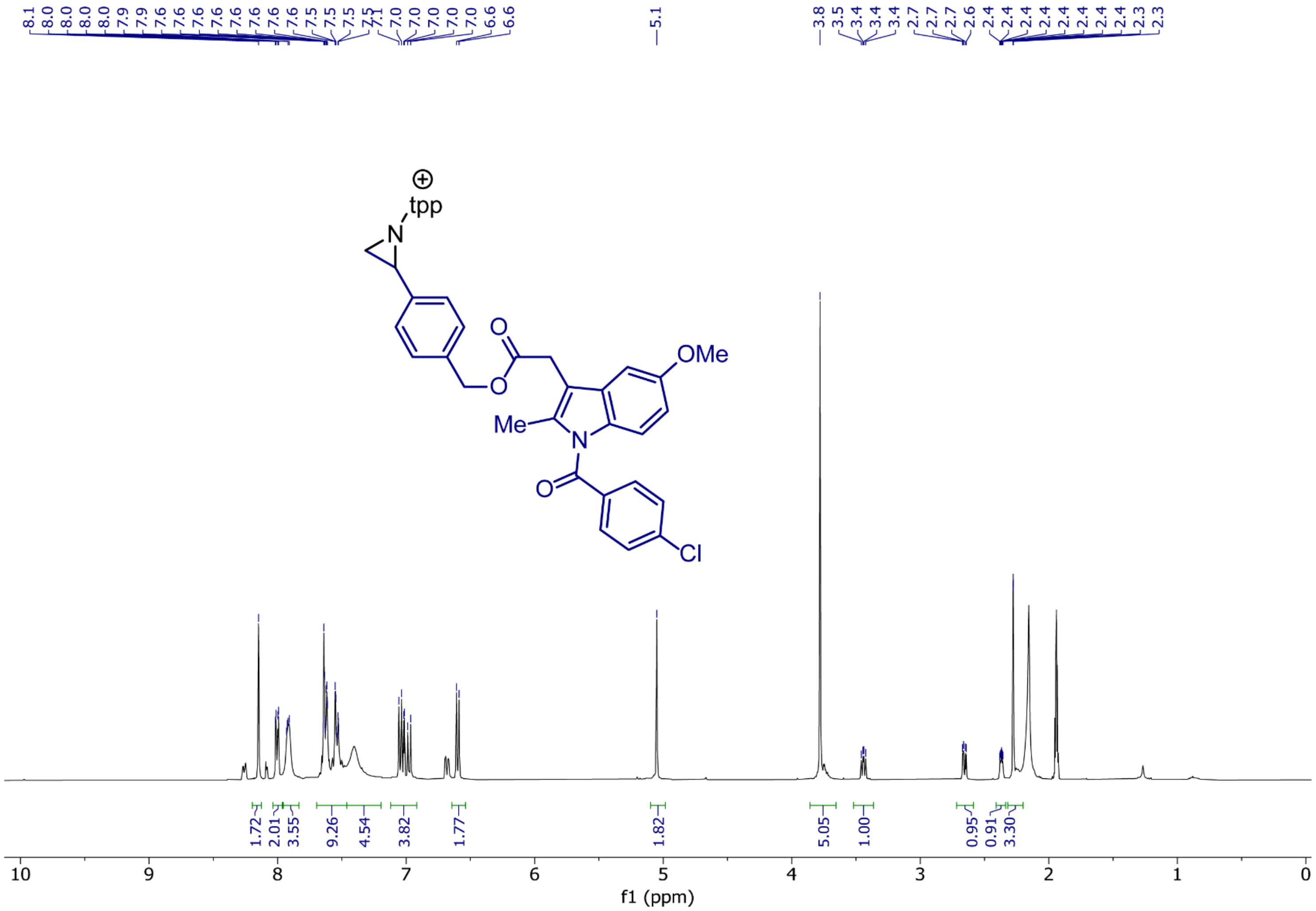

Figure S12. ^{1}H NMR spectrum of **2aa** in CD_3CN (400 MHz) at 23 °C.

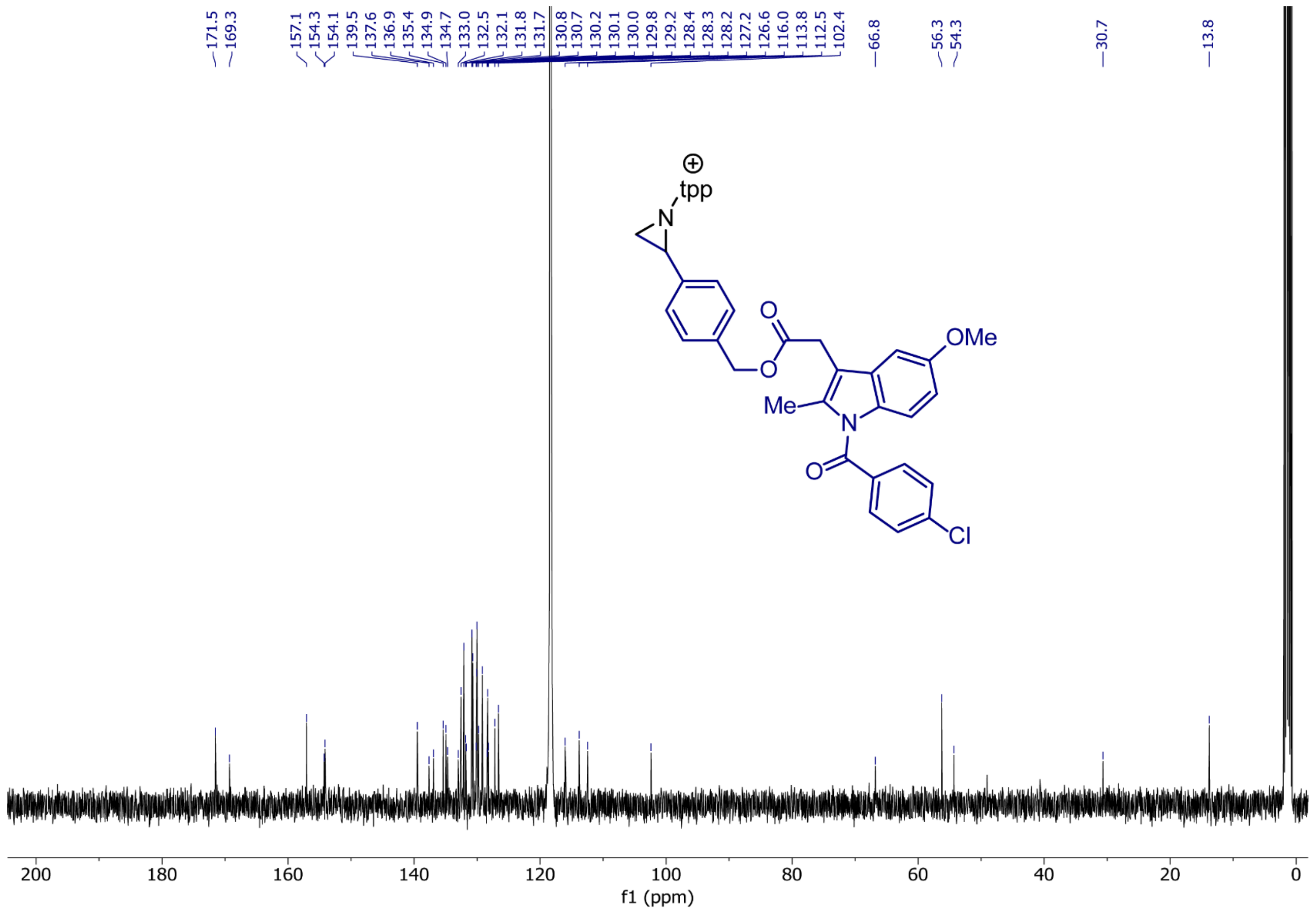

Figure S13. ^{13}C NMR spectrum of **2aa** in CD_3CN (101MHz) at 23 °C.

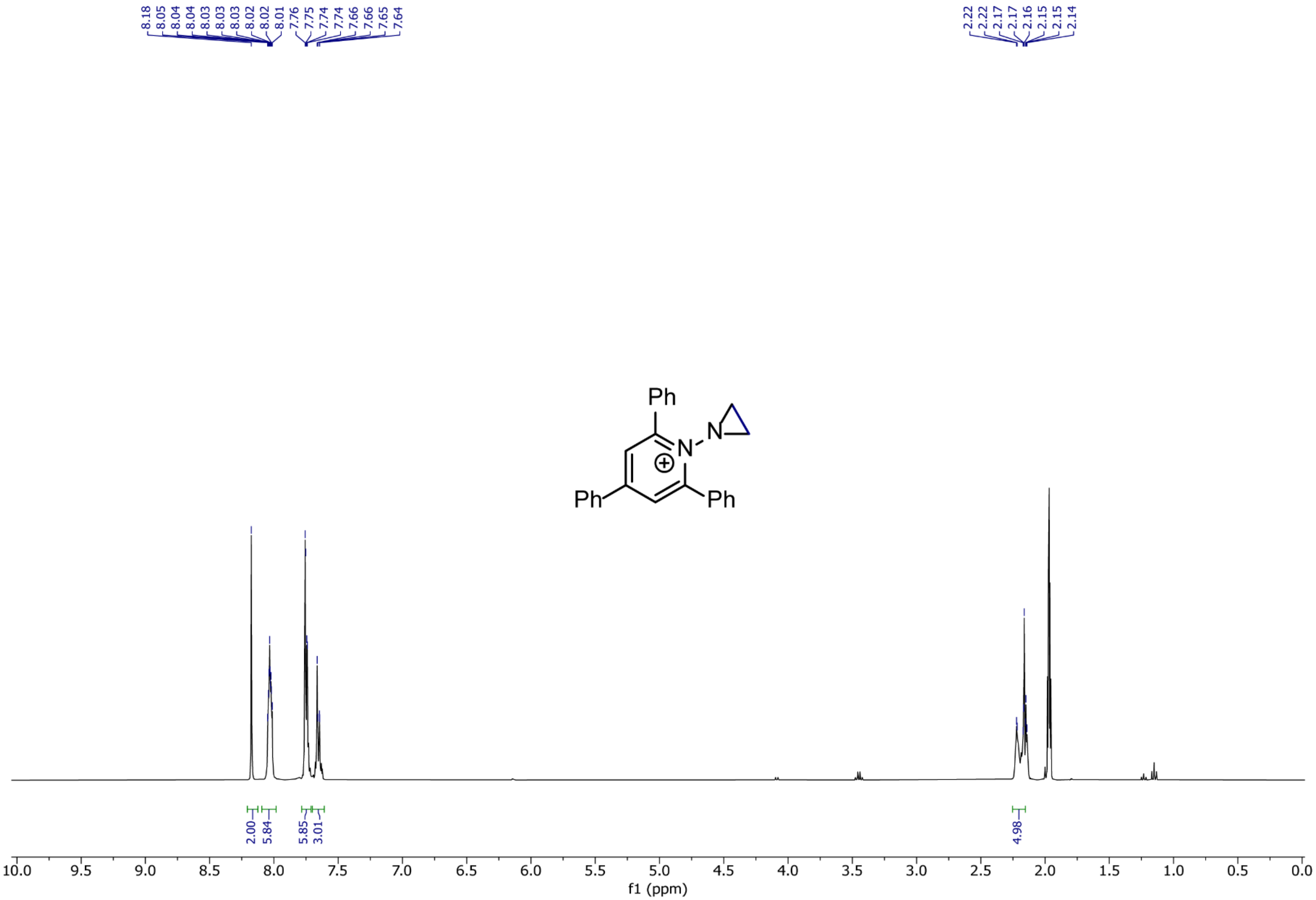

Figure S14. ^{1}H NMR spectrum of **8** in CD_3CN (400 MHz) at 23 °C.

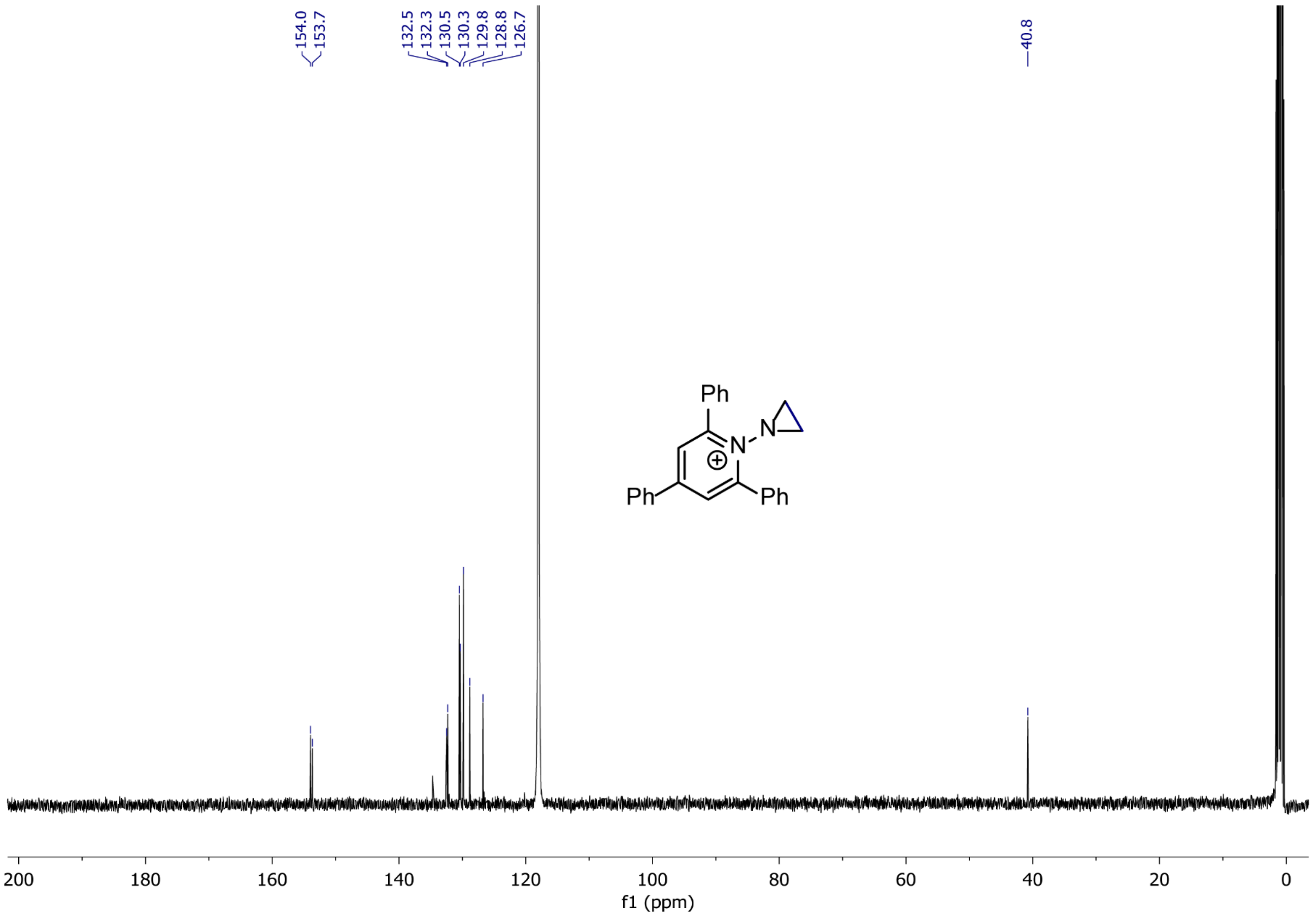

Figure S15. ^{13}C NMR spectrum of **8** in CD_3CN (101MHz) at 23 °C.

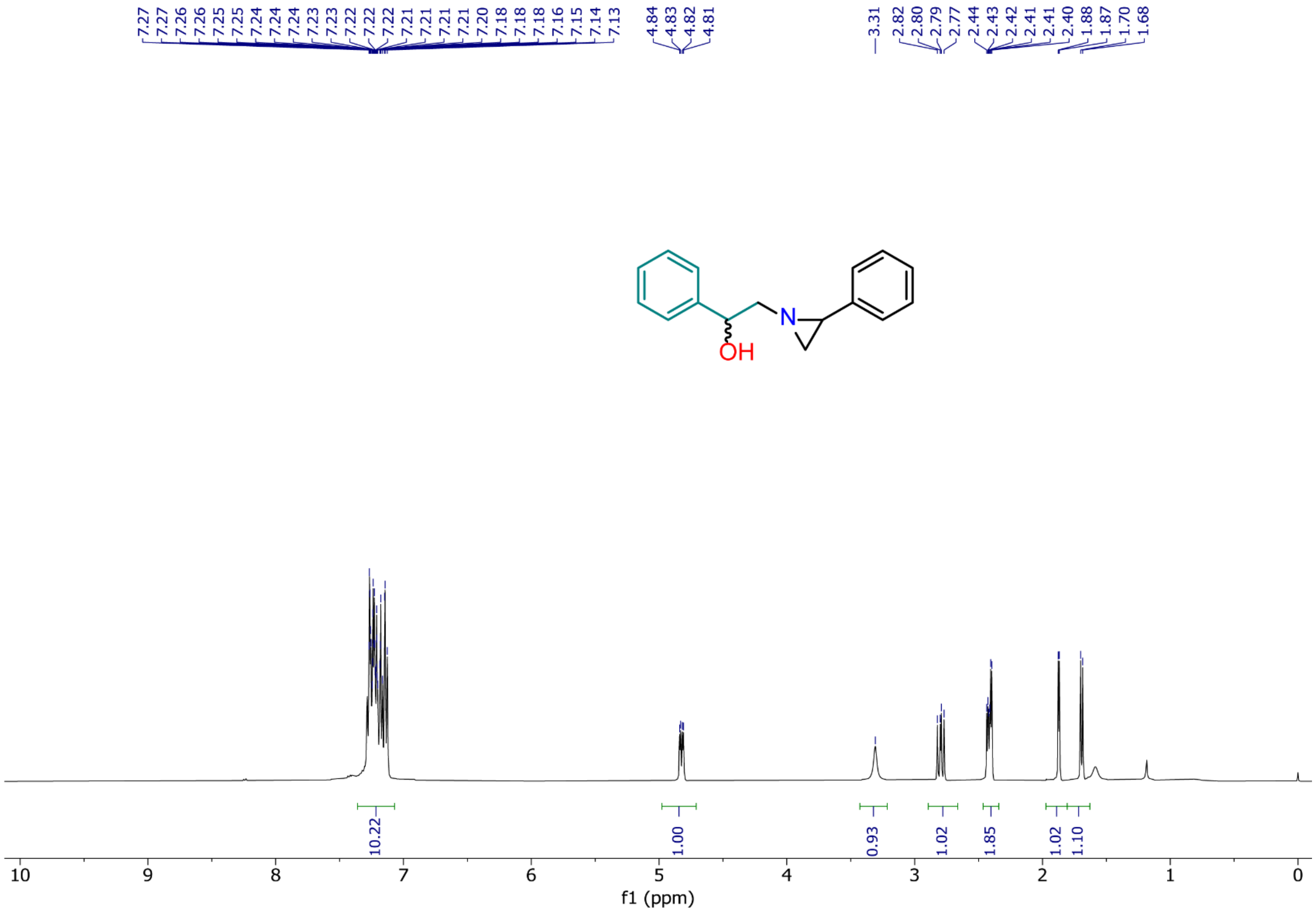

Figure S16. ^{1}H NMR spectrum of 1-phenyl-2-(2-phenylaziridin-1-yl)ethan-1-ol (**5a**) in $CDCl_3$ (400 MHz) at 23 °C.

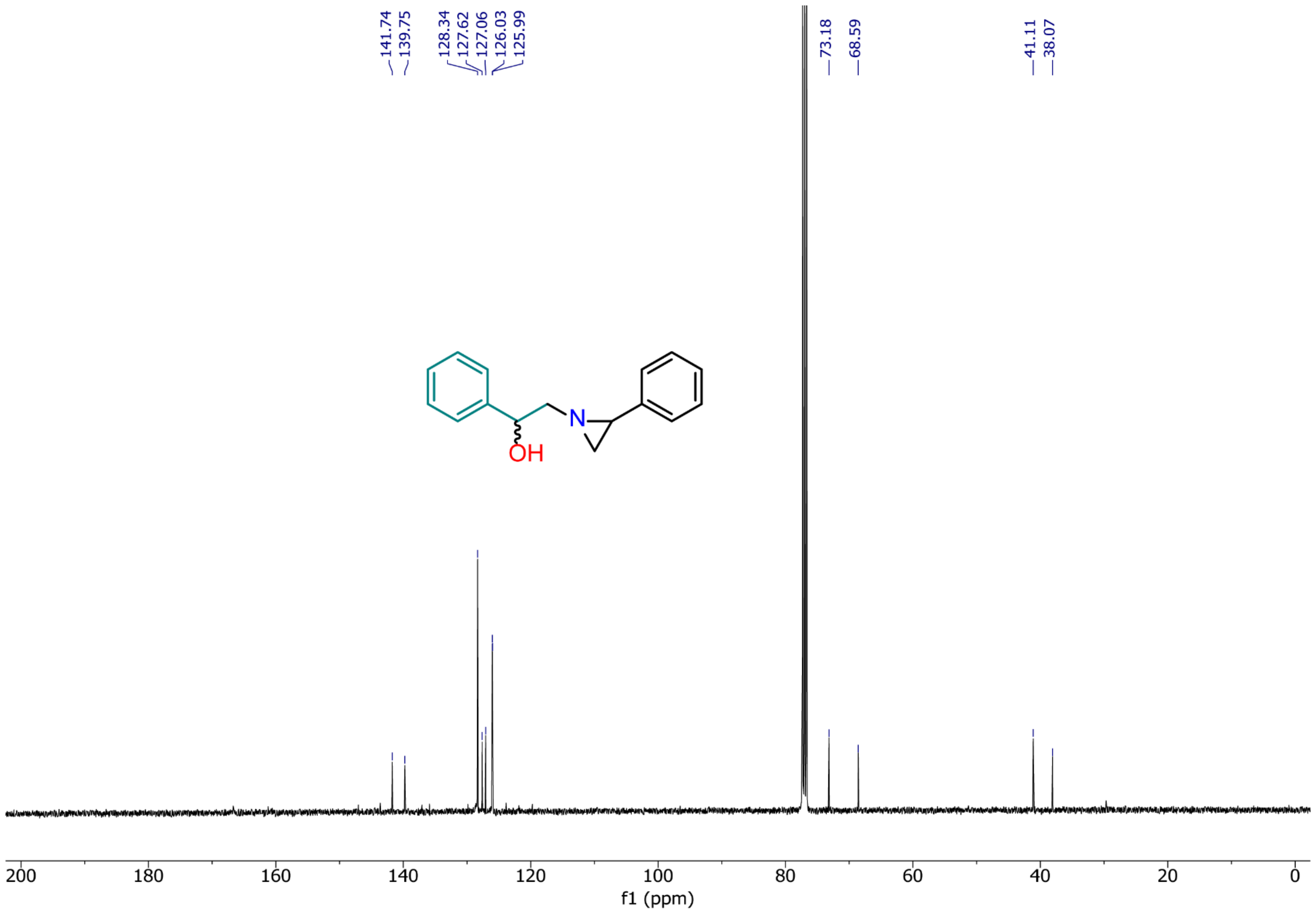

Figure S17. ^{13}C NMR spectrum of 1-phenyl-2-(2-phenylaziridin-1-yl)ethan-1-ol (**5a**) in $CDCl_3$ (101MHz) at 23 °C.

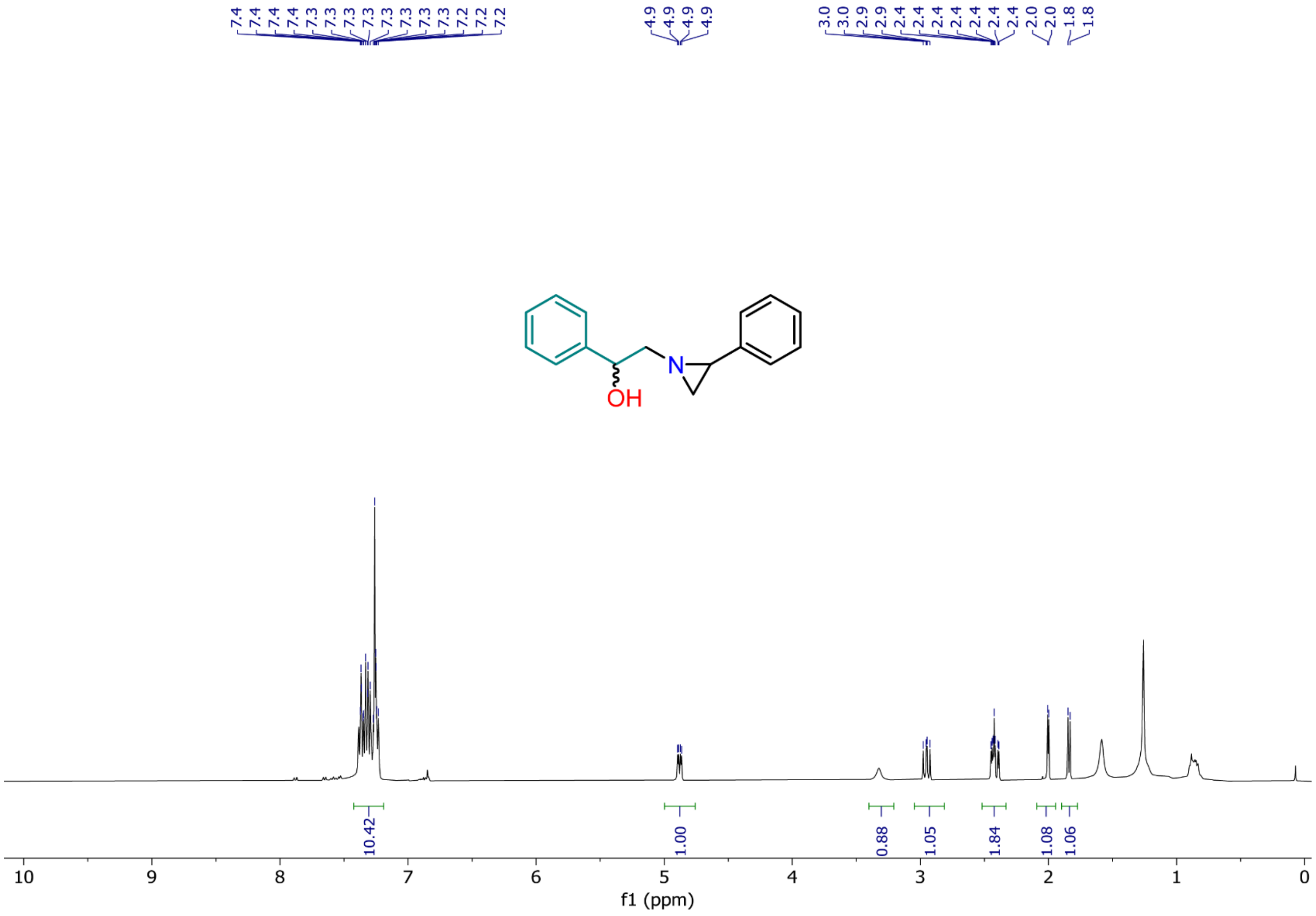

Figure S18. ^{1}H NMR spectrum of 1-phenyl-2-(2-phenylaziridin-1-yl)ethan-1-ol (**5a**) in $CDCl_3$ (400 MHz) at 23 °C.

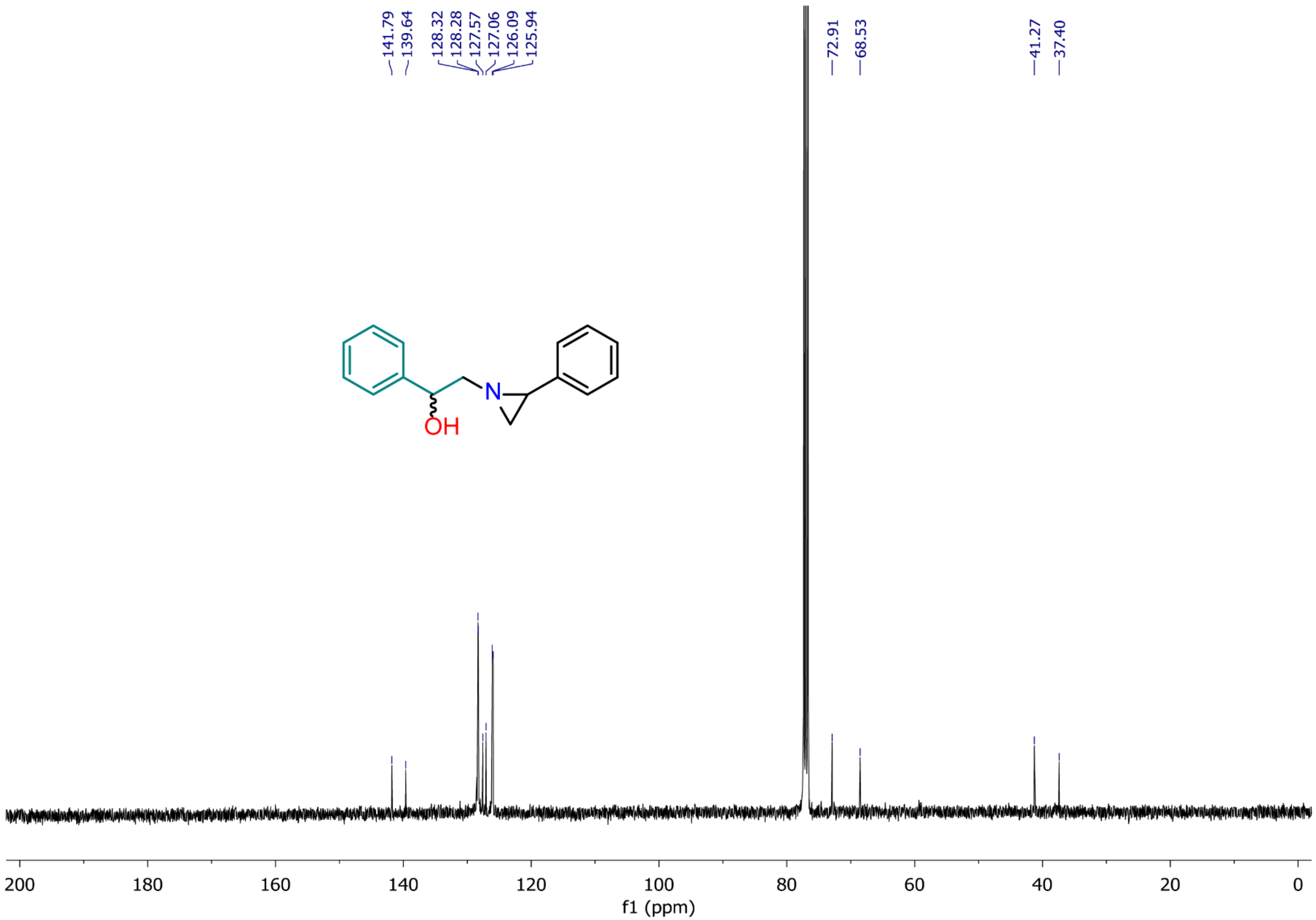

Figure S19. ^{13}C NMR spectrum of 1-phenyl-2-(2-phenylaziridin-1-yl)ethan-1-ol (**5a**) in $CDCl_3$ (101MHz) at 23 °C.

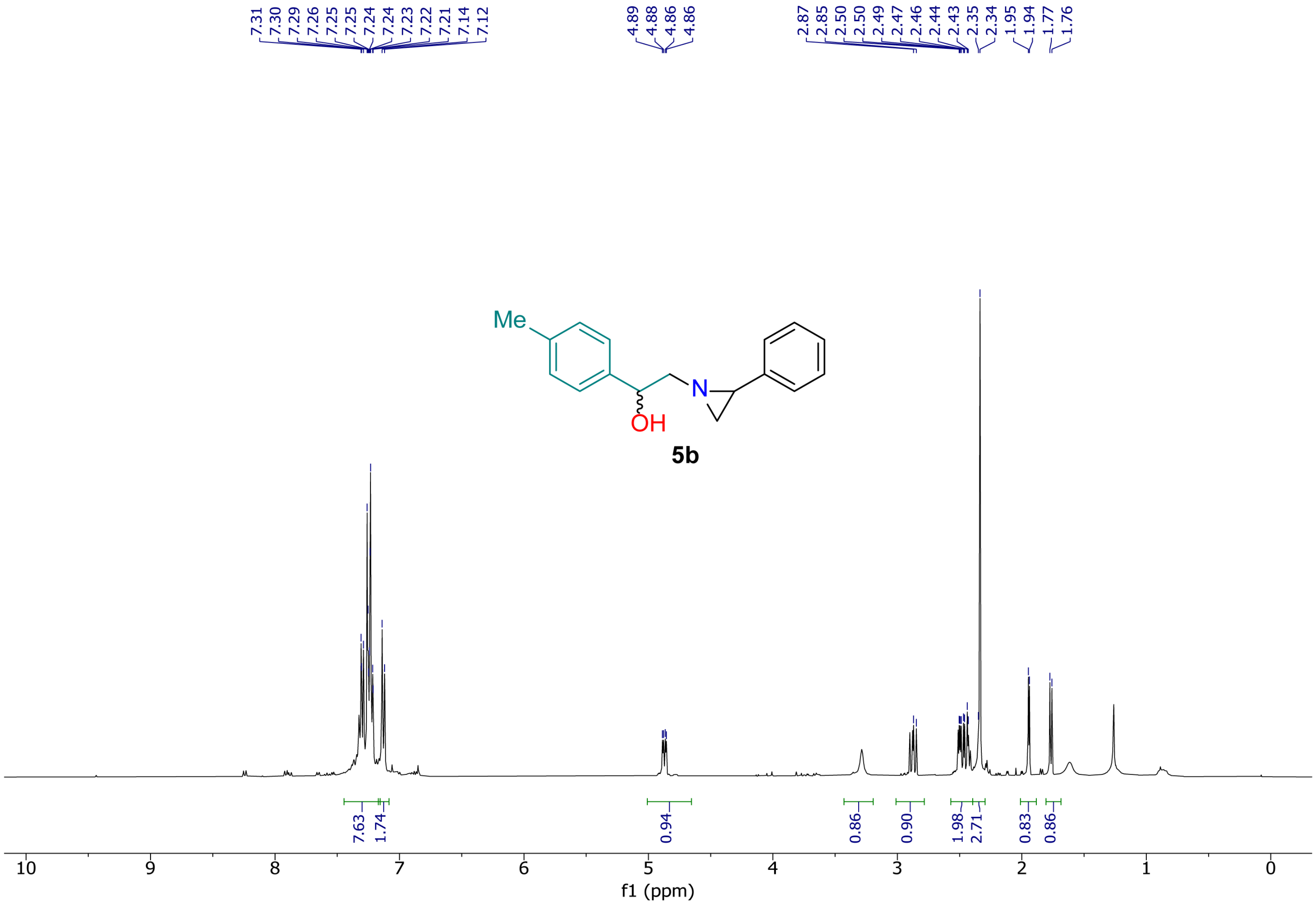

Figure S20. ^{1}H NMR spectrum of 2-(2-phenylaziridin-1-yl)-1-(p-tolyl)ethan-1-ol (**5b**) in $CDCl_3$ (400 MHz) at 23 °C.

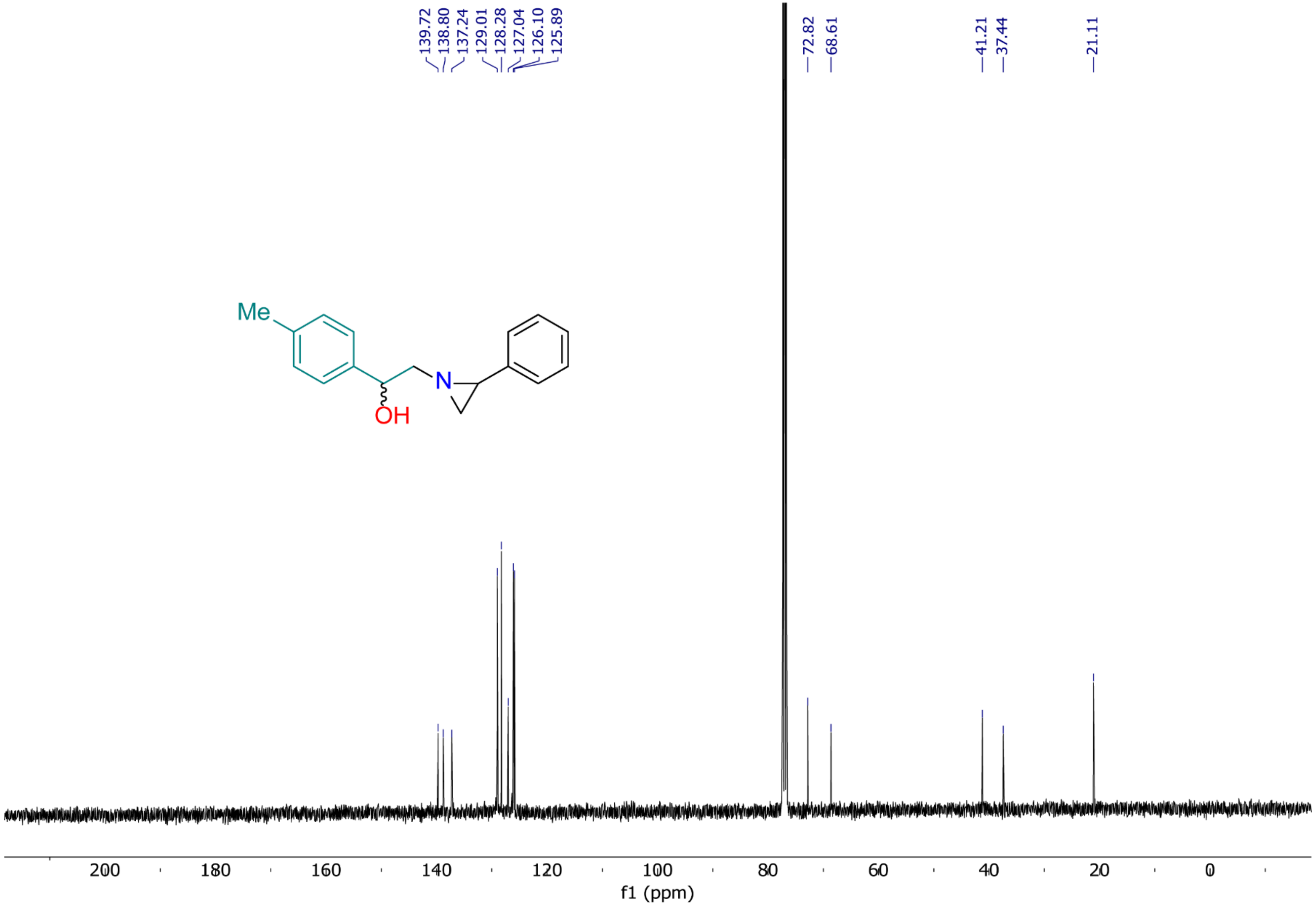

Figure S21. ^{13}C NMR spectrum of of 2-(2-phenylaziridin-1-yl)-1-(p-tolyl)ethan-1-ol (**5b**) in $CDCl_3$ (101MHz) at 23 °C.

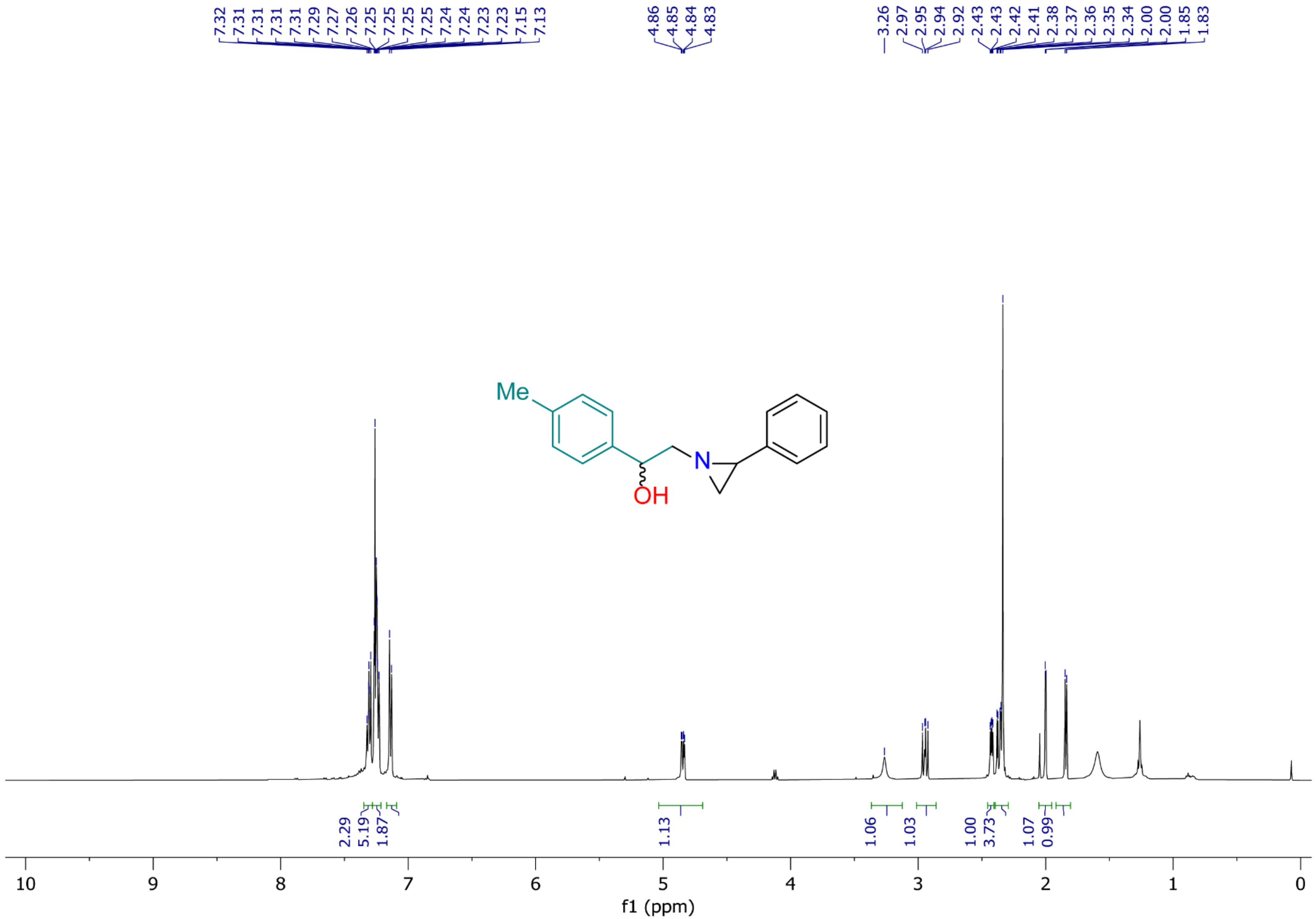

Figure S22. ¹H NMR spectrum of 2-(2-phenylaziridin-1-yl)-1-(p-tolyl)ethan-1-ol (**5b**) in $CDCl_3$ (400 MHz) at 23 °C.

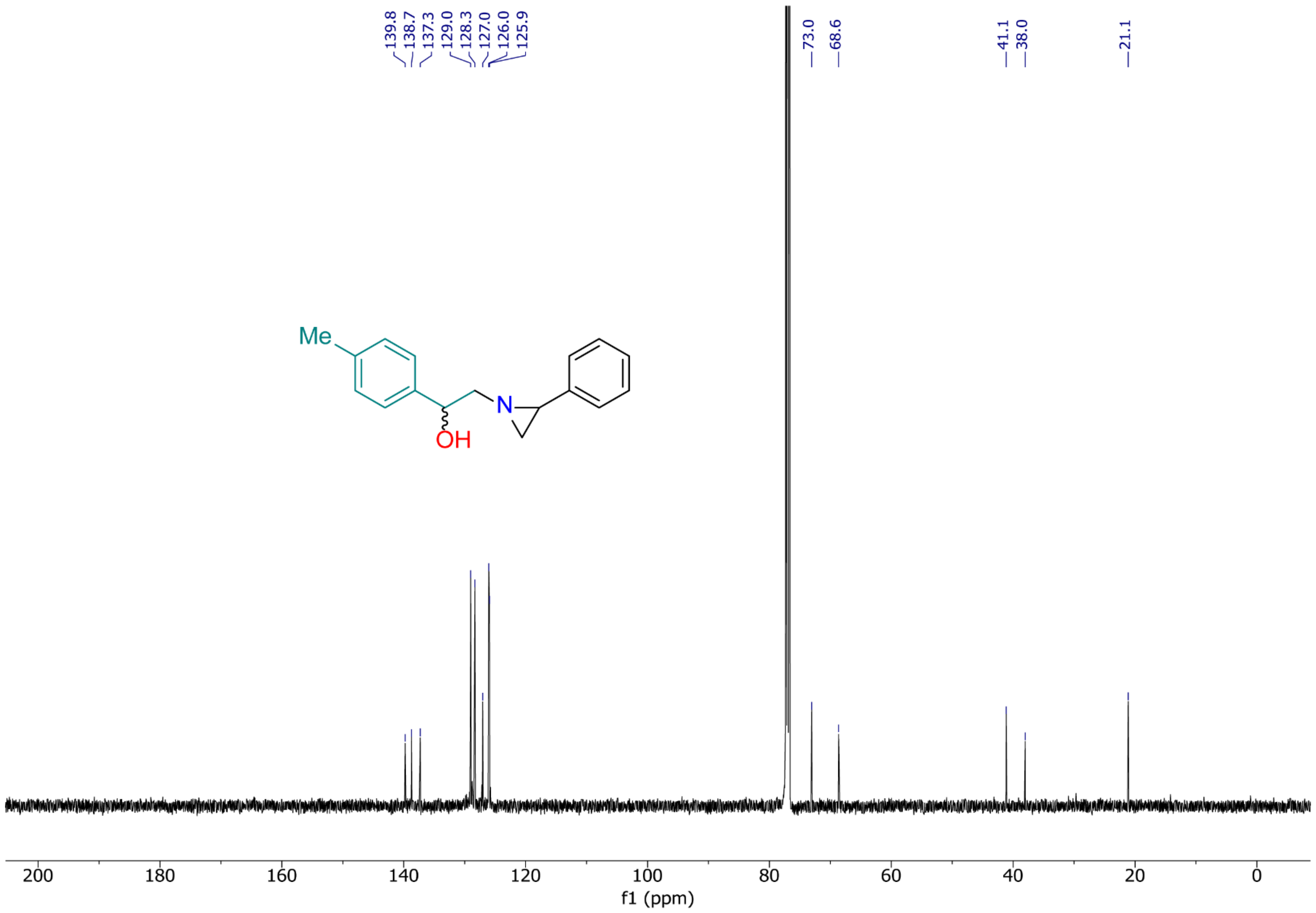

Figure S23. ^{13}C NMR spectrum of of 2-(2-phenylaziridin-1-yl)-1-(p-tolyl)ethan-1-ol (**5b**) in $CDCl_3$ (101MHz) at 23 °C.

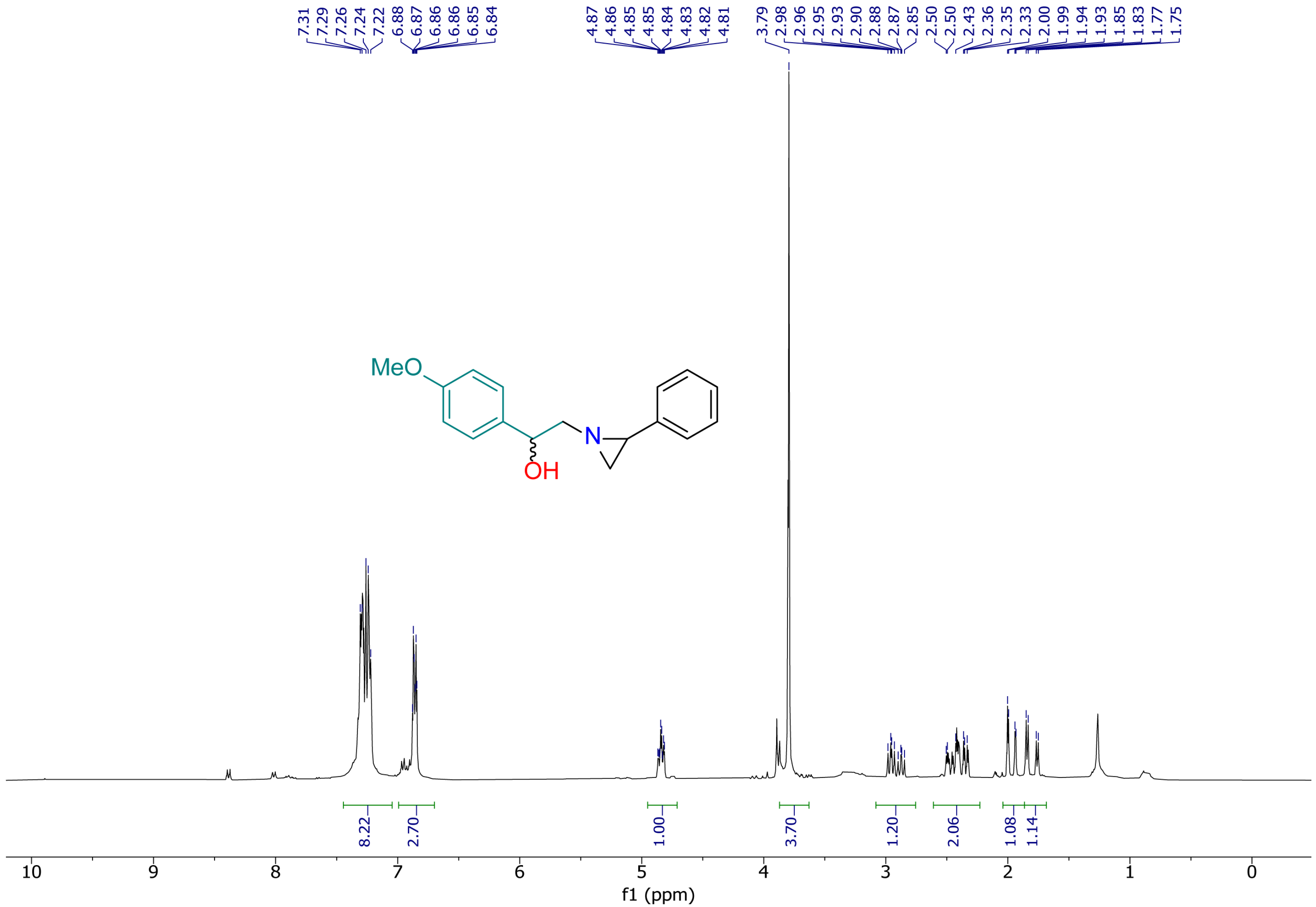

Figure S24. ^{1}H NMR spectrum of 1-(4-Methoxyphenyl)-2-(2-phenylaziridin-1-yl)ethan-1-ol (**5c**) in $CDCl_3$ (400 MHz) at 23 °C.

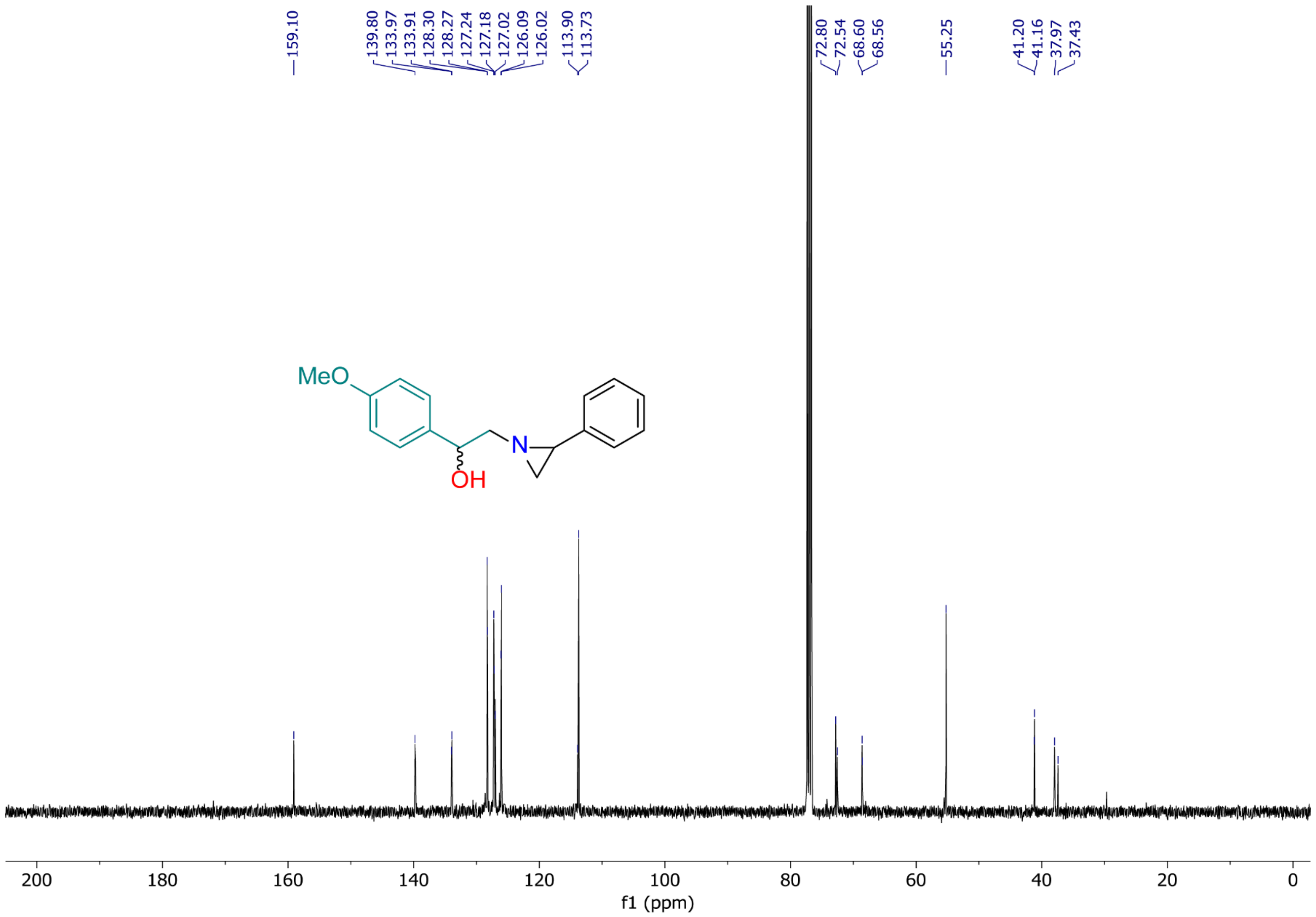

Figure S25. ^{13}C NMR spectrum of 1-(4-methoxyphenyl)-2-(2-phenylaziridin-1-yl)ethan-1-ol (**5c**) in $CDCl_3$ (101 MHz) at 23 °C.

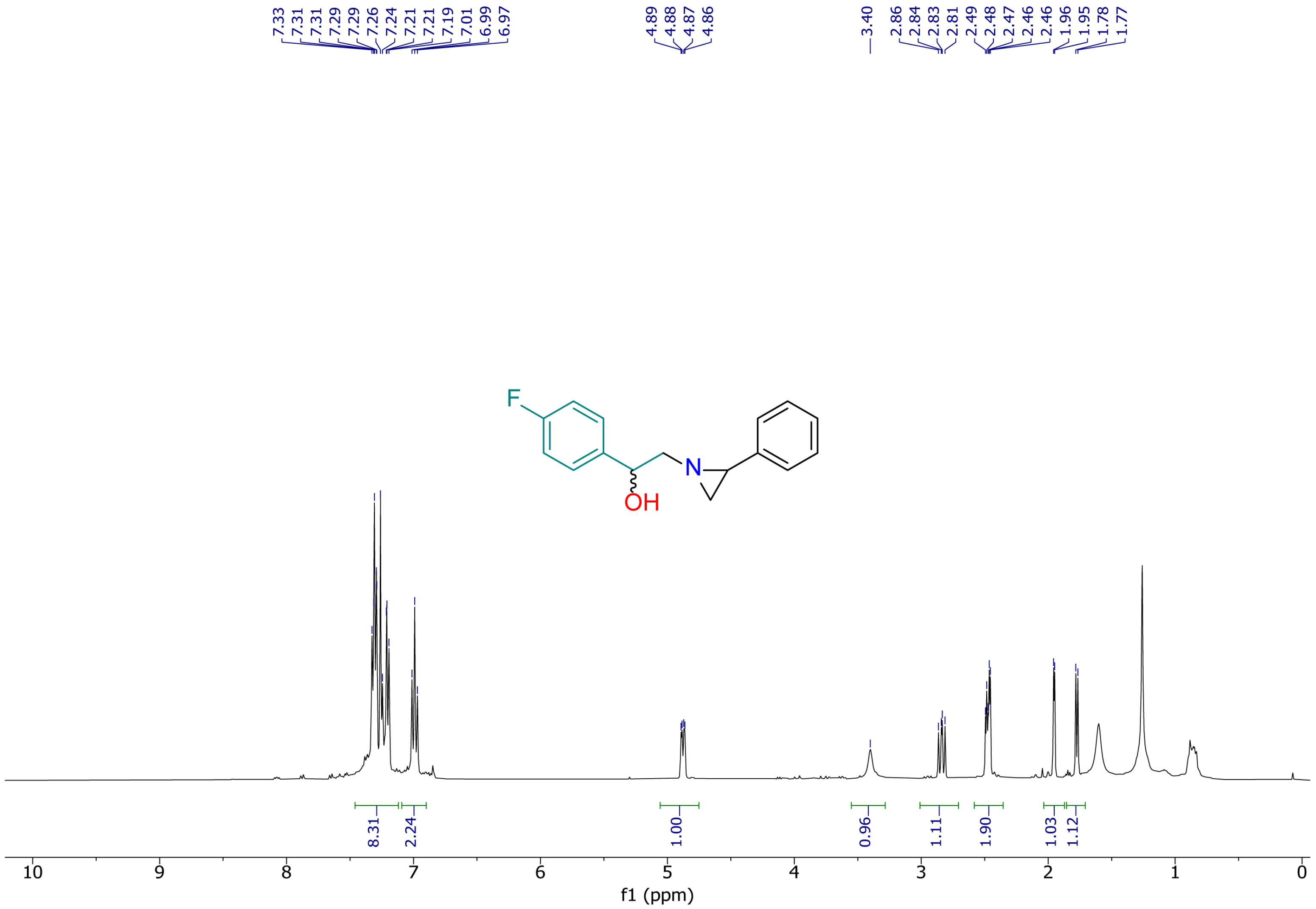

Figure S26. ^{1}H NMR spectrum of 1-(4-fluorophenyl)-2-(2-phenylaziridin-1-yl)ethan-1-ol (**5d**) in $CDCl_3$ (400 MHz) at 23 °C.

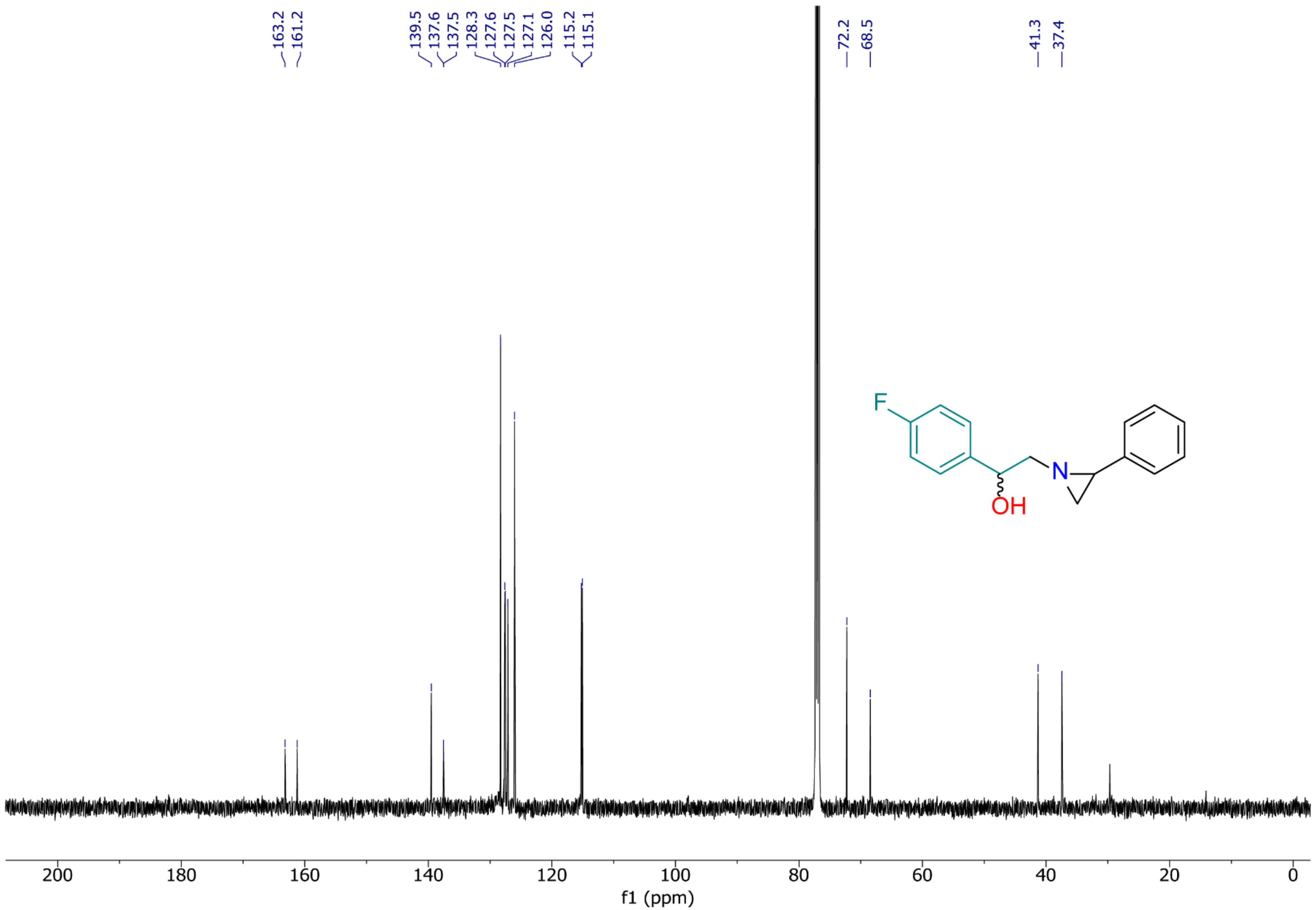

Figure S27. ^{13}C NMR spectrum of 1-(4-fluorophenyl)-2-(2-phenylaziridin-1-yl)ethan-1-ol (**5d**) in $CDCl_3$ (126 MHz) at 23 °C.

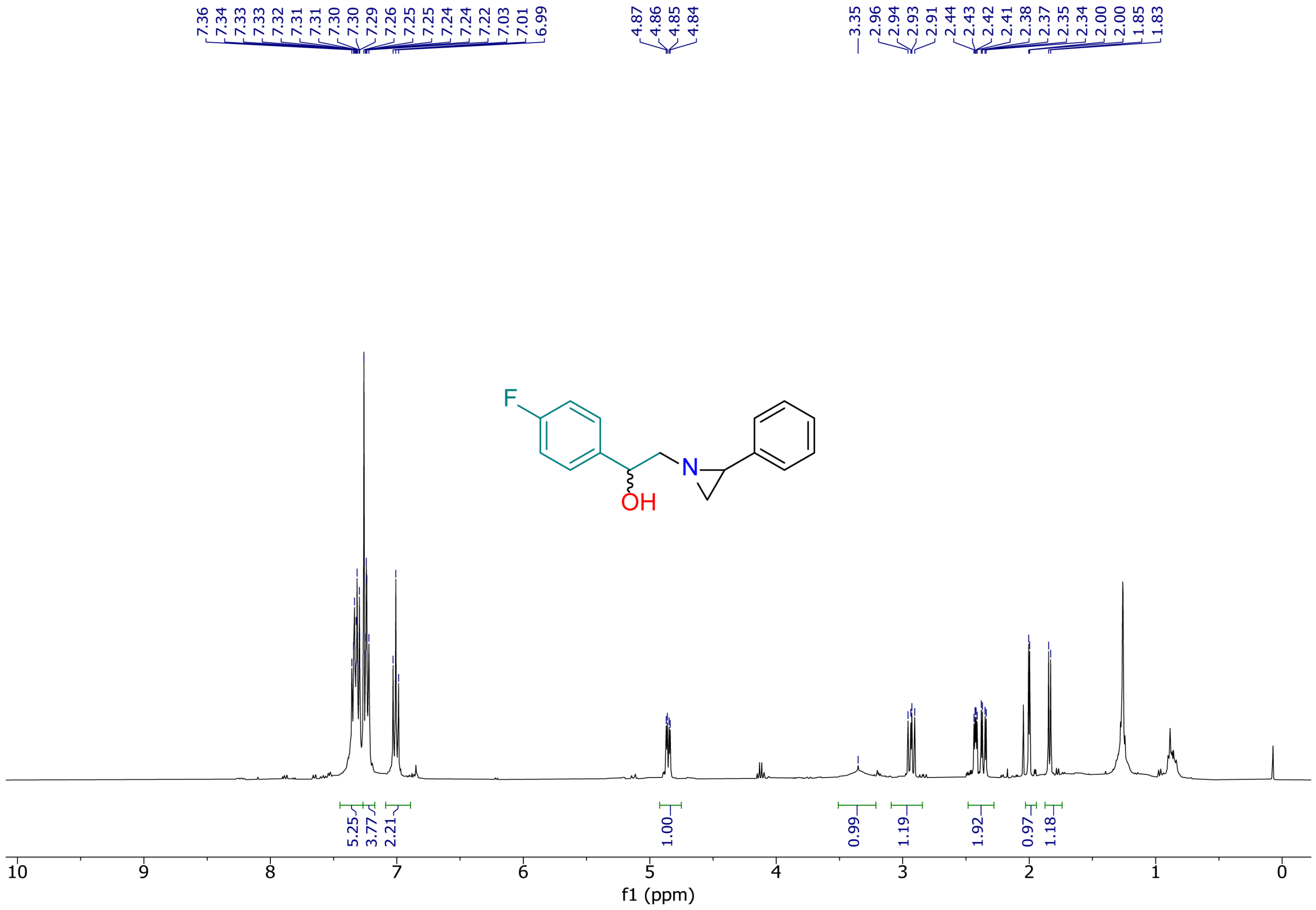

Figure S28. ^{1}H NMR spectrum of 1-(4-fluorophenyl)-2-(2-phenylaziridin-1-yl)ethan-1-ol (**5d**) in $CDCl_3$ (400 MHz) at 23 °C.

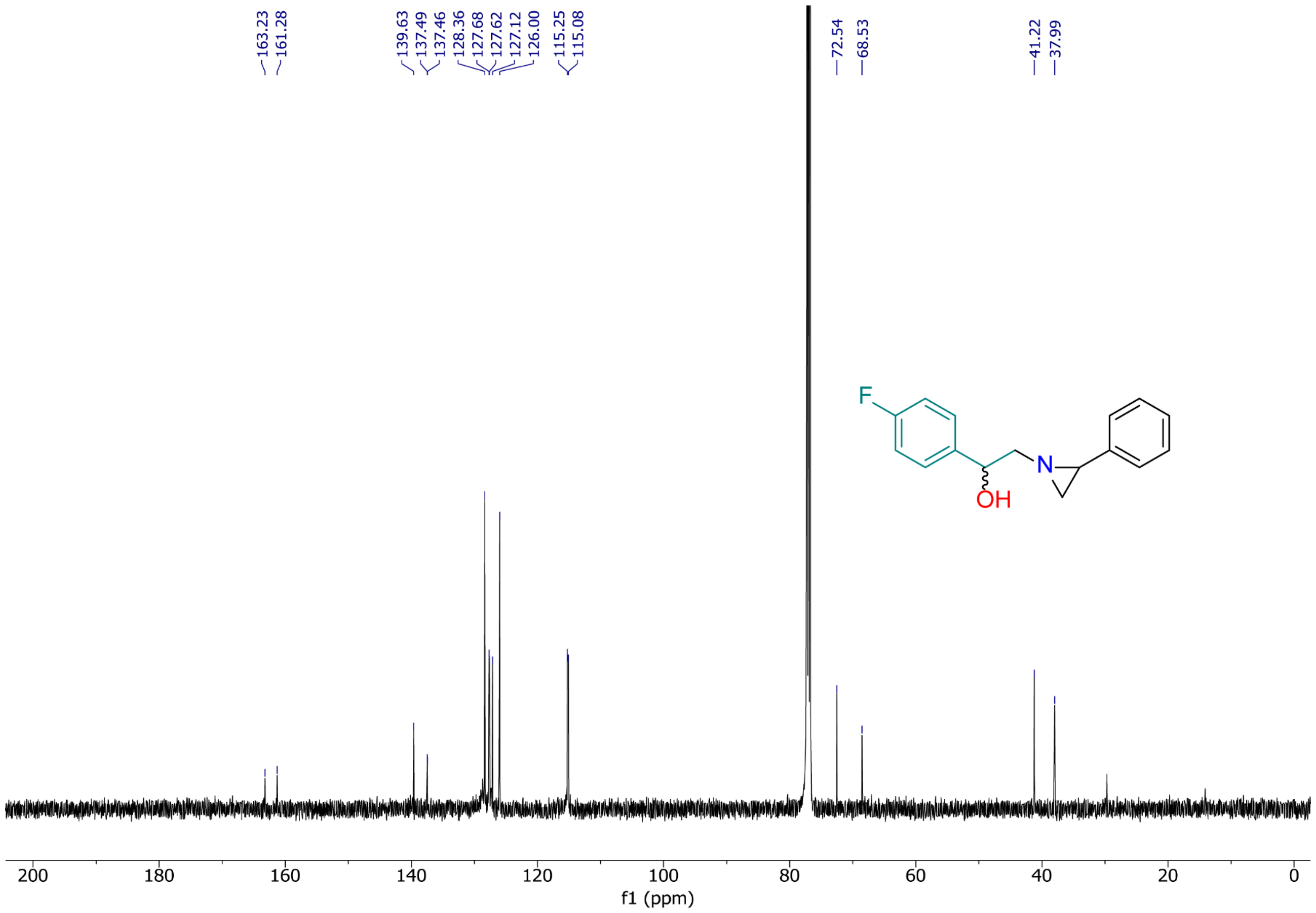

Figure S29. ^{13}C NMR spectrum of 1-(4-fluorophenyl)-2-(2-phenylaziridin-1-yl)ethan-1-ol (**5d**) in $CDCl_3$ (101 MHz) at 23 °C.

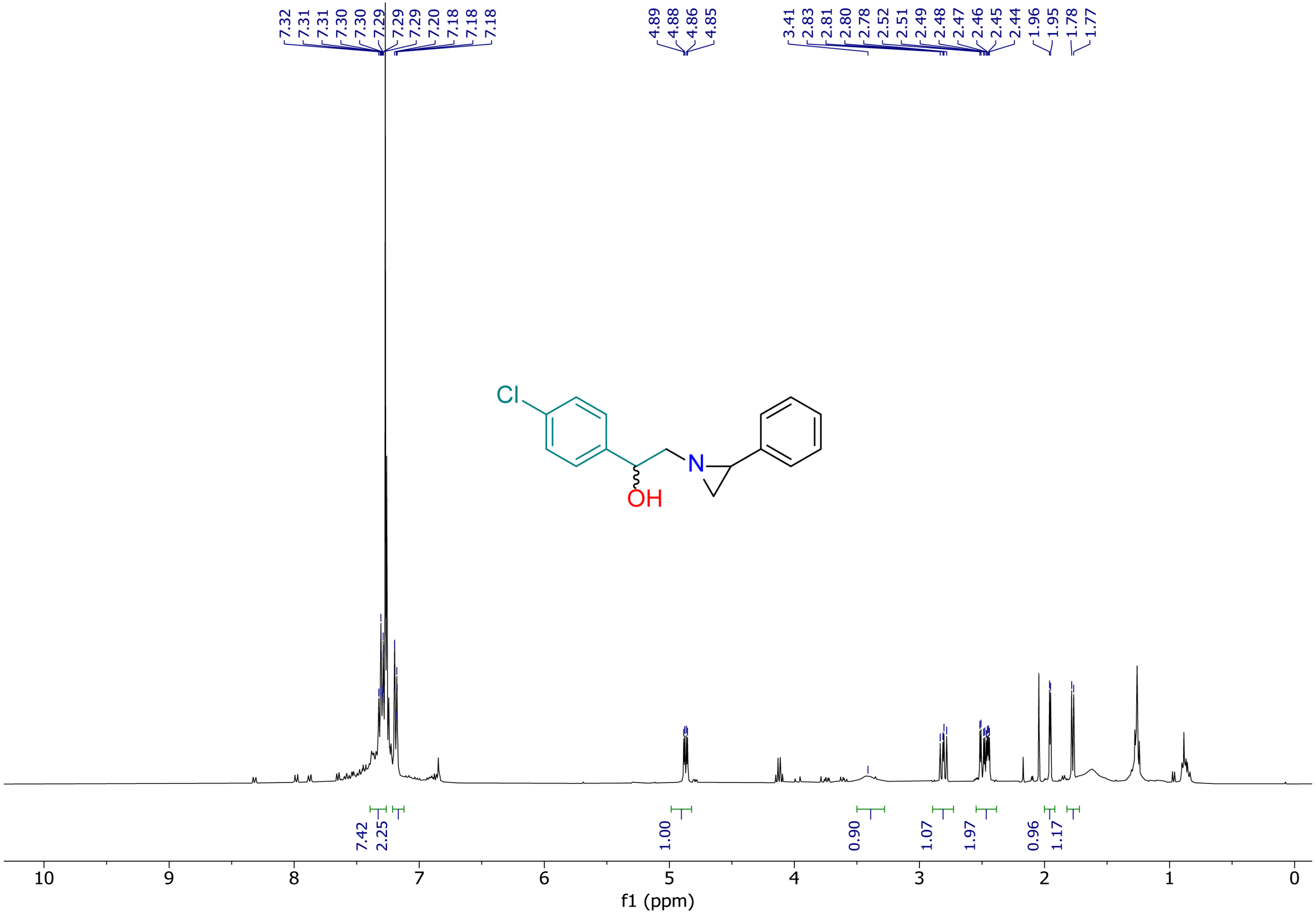

Figure S30. ^{1}H NMR spectrum of 1-(4-chlorophenyl)-2-(2-phenylaziridin-1-yl)ethan-1-ol (**5e**) in $CDCl_3$ (400 MHz) at 23 °C.

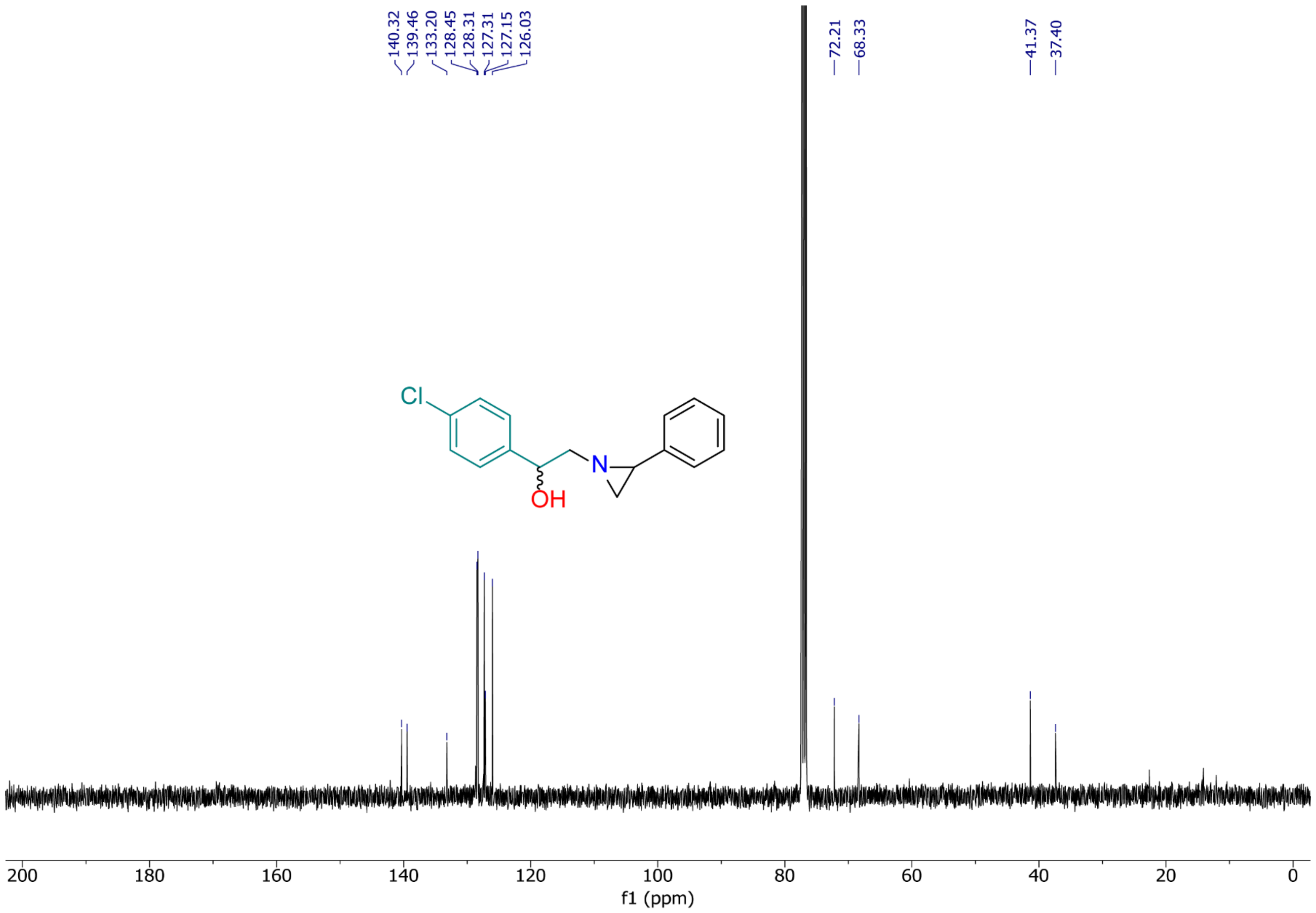

Figure S31. ^{13}C NMR spectrum of 1-(4-chlorophenyl)-2-(2-phenylaziridin-1-yl)ethan-1-ol (**5e**) in $CDCl_3$ (101 MHz) at 23 °C.

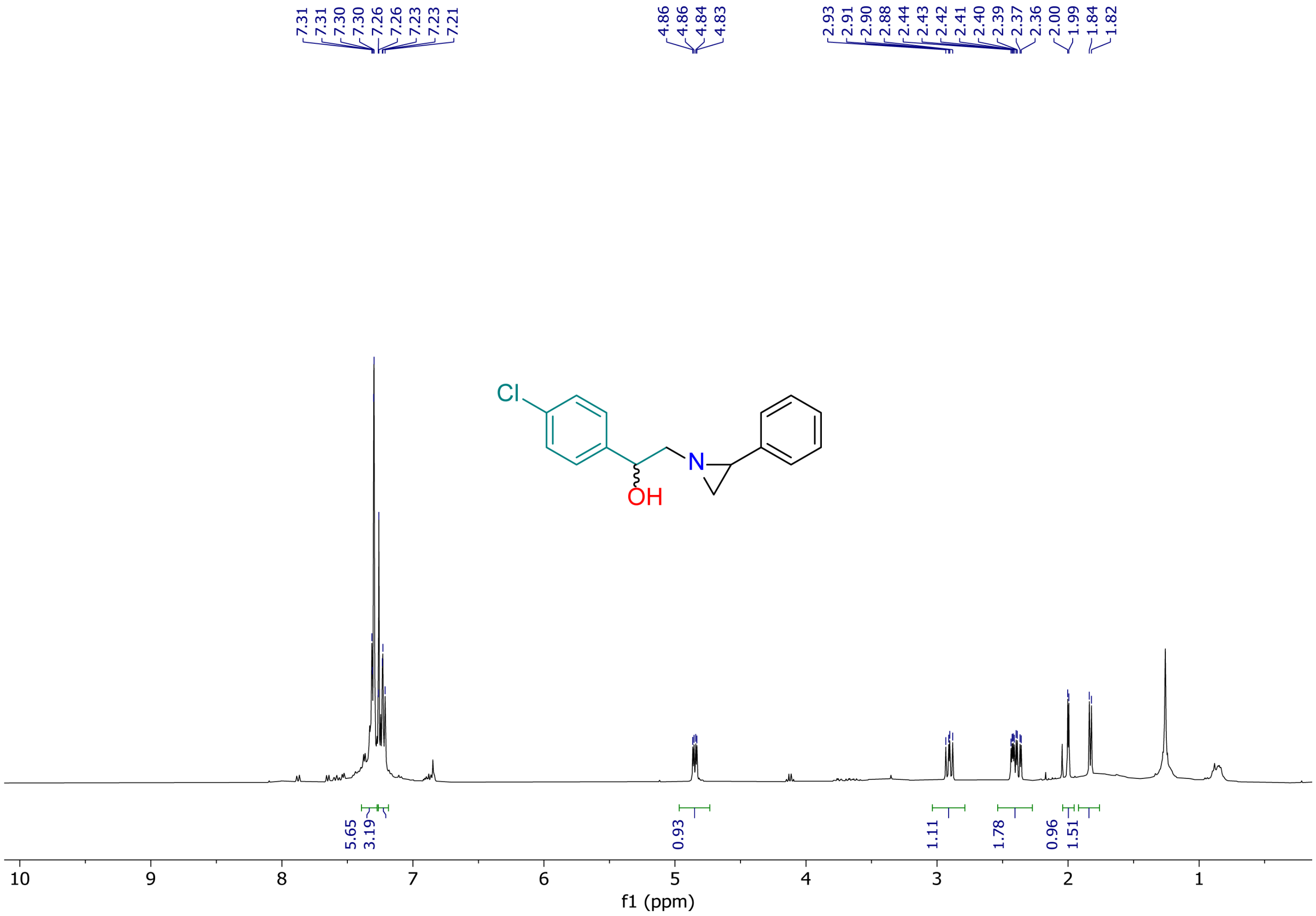

Figure S32. ^{1}H NMR spectrum of 1-(4-chlorophenyl)-2-(2-phenylaziridin-1-yl)ethan-1-ol (**5e**) in $CDCl_3$ (400 MHz) at 23 °C.

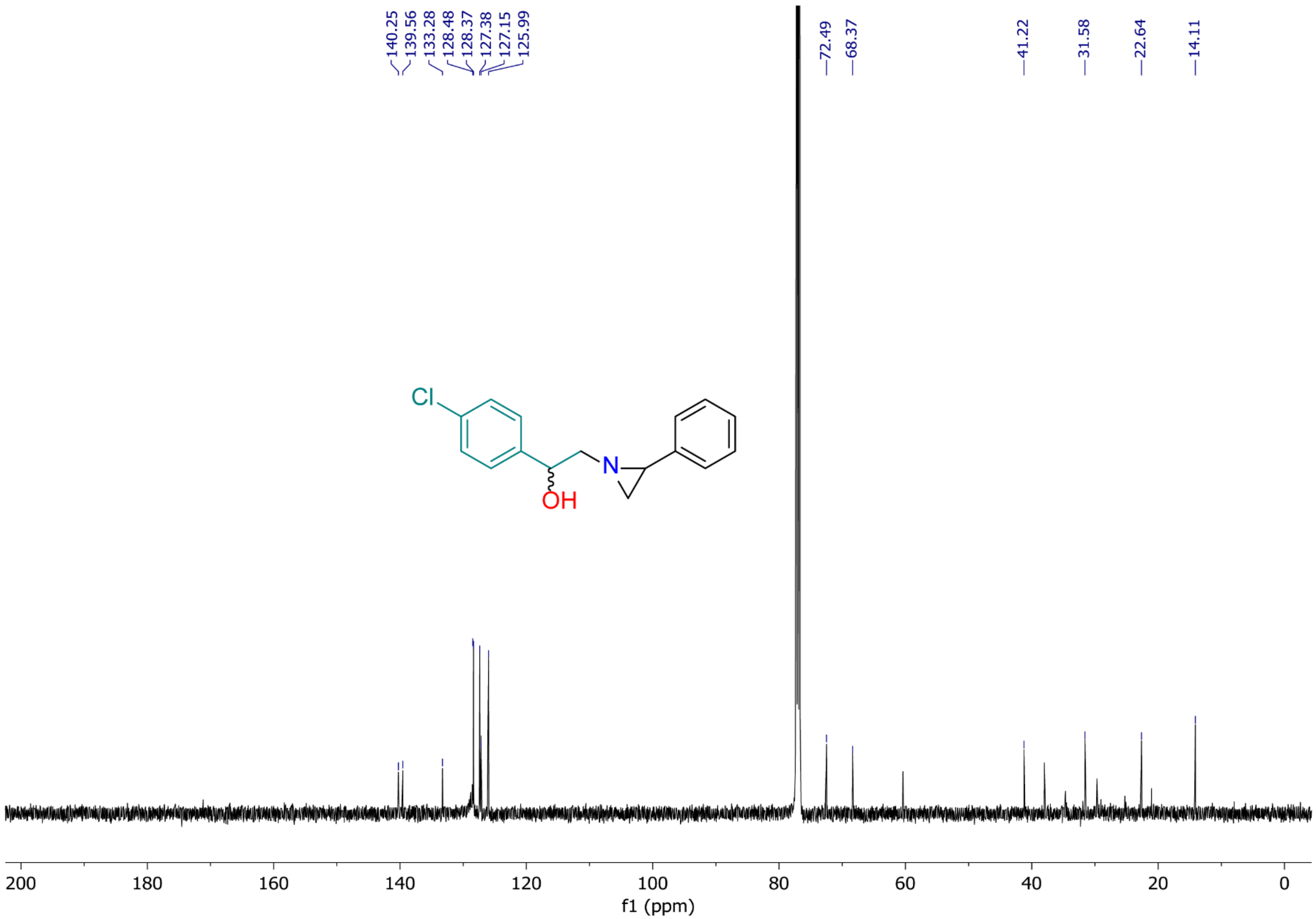

Figure S33. ^{13}C NMR spectrum of 1-(4-chlorophenyl)-2-(2-phenylaziridin-1-yl)ethan-1-ol (**5e**) in $CDCl_3$ (101 MHz) at 23 °C.

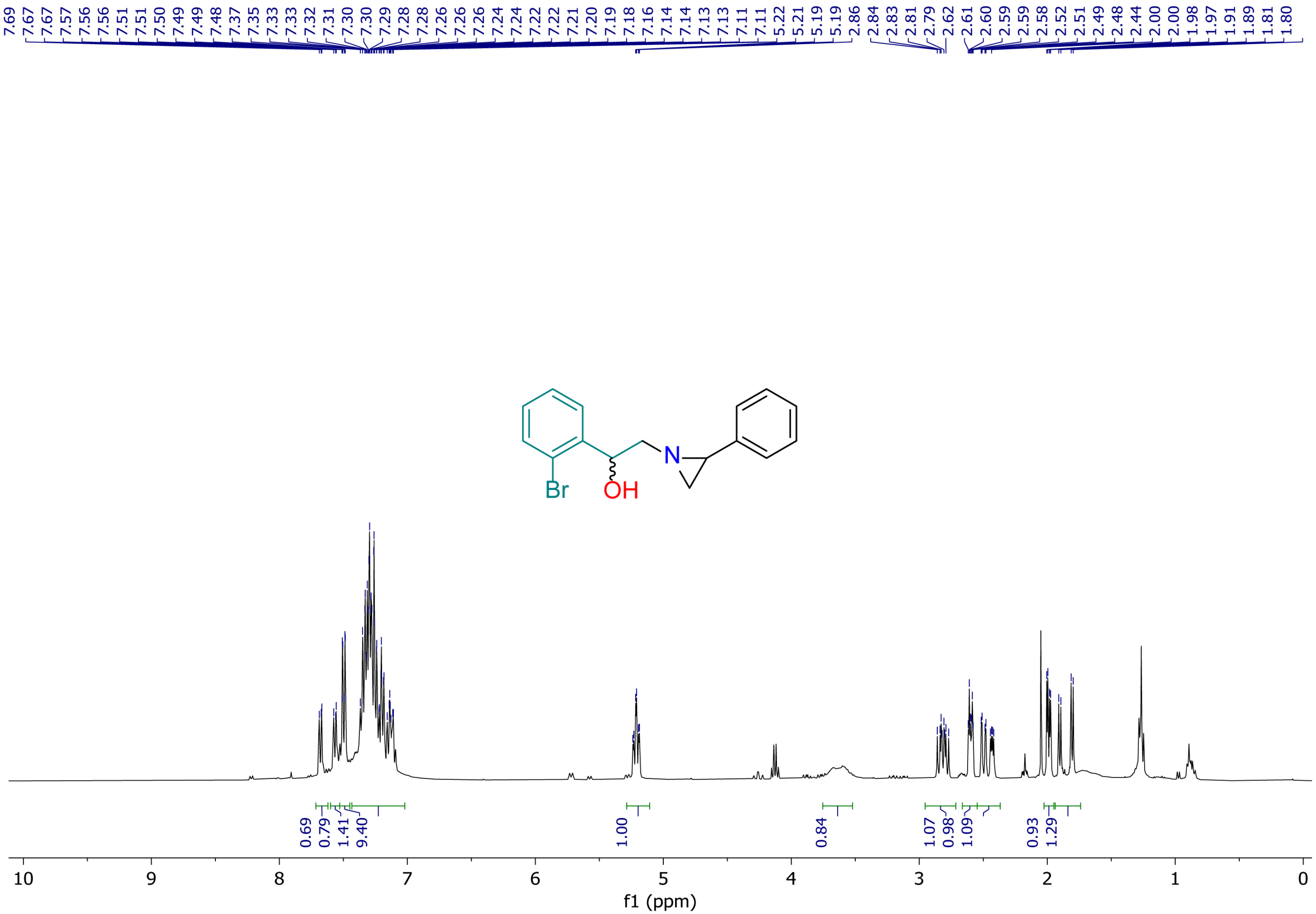

Figure S34. ^{1}H NMR spectrum of 1-(2-bromophenyl)-2-(2-phenylaziridin-1-yl)ethan-1-ol (**5f**) in $CDCl_3$ (400 MHz) at 23 °C.

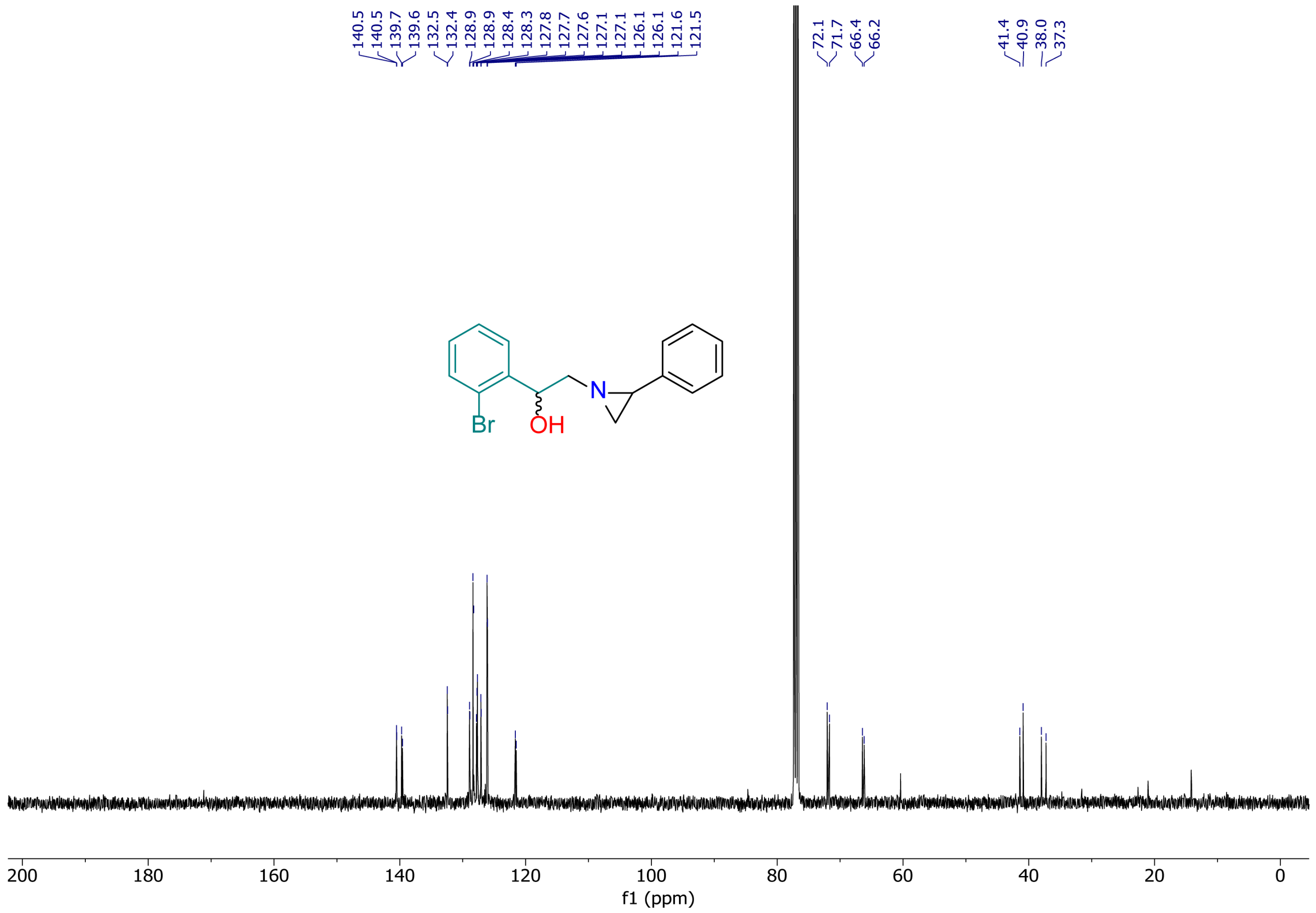

Figure S35. ^{13}C NMR spectrum of 1-(2-bromophenyl)-2-(2-phenylaziridin-1-yl)ethan-1-ol (**5f**) in $CDCl_3$ (101 MHz) at 23 °C.

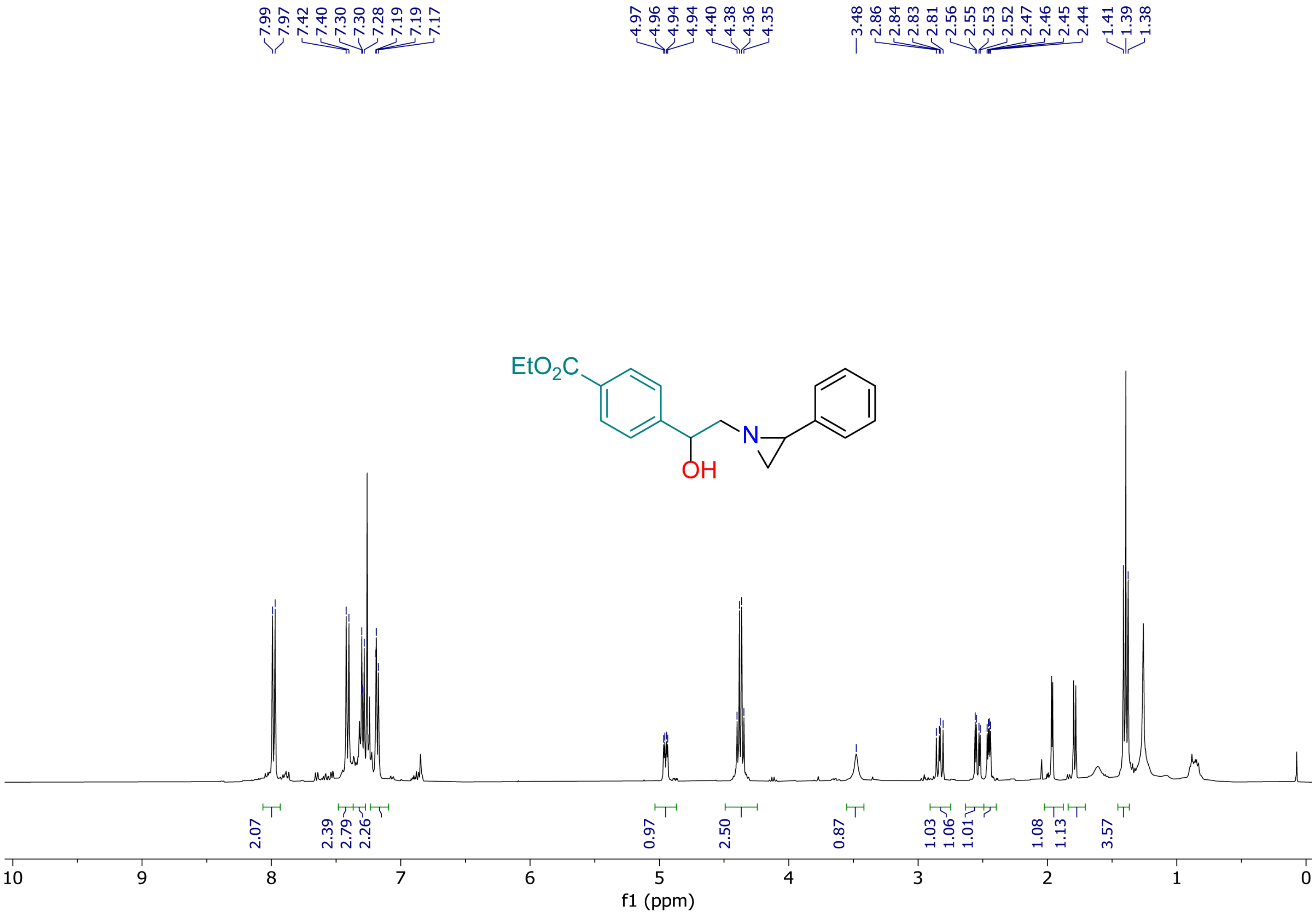

Figure S36. ^{1}H NMR spectrum of ethyl 4-(1-hydroxy-2-(2-phenylaziridin-1-yl)ethyl)benzoate (**5g**) in $CDCl_3$ (400 MHz) at 23 °C.

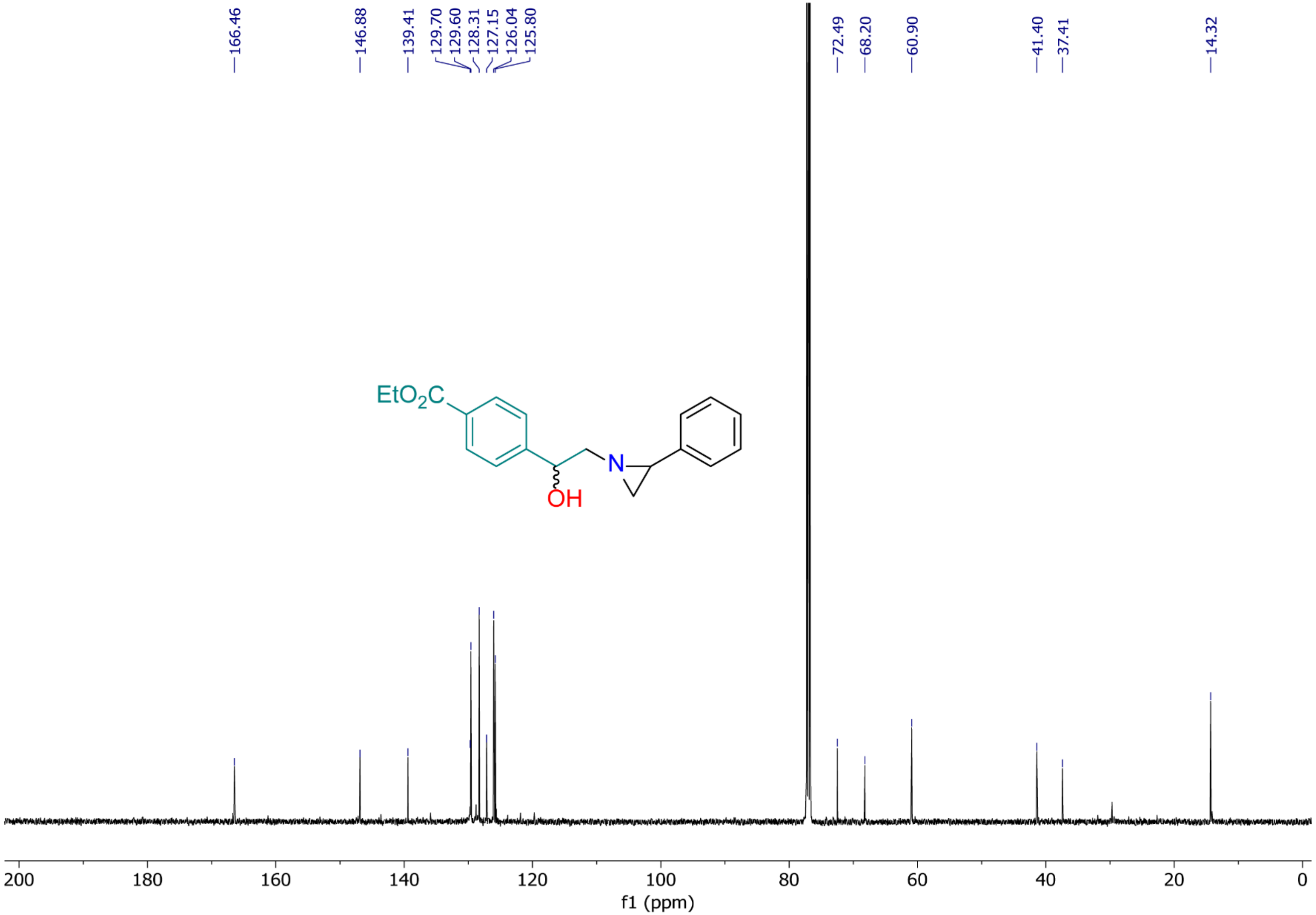

Figure S37. ^{13}C NMR spectrum of ethyl 4-(1-hydroxy-2-(2-phenylaziridin-1-yl)ethyl)benzoate (**5g**) in $CDCl_3$ (101 MHz) at 23 °C.

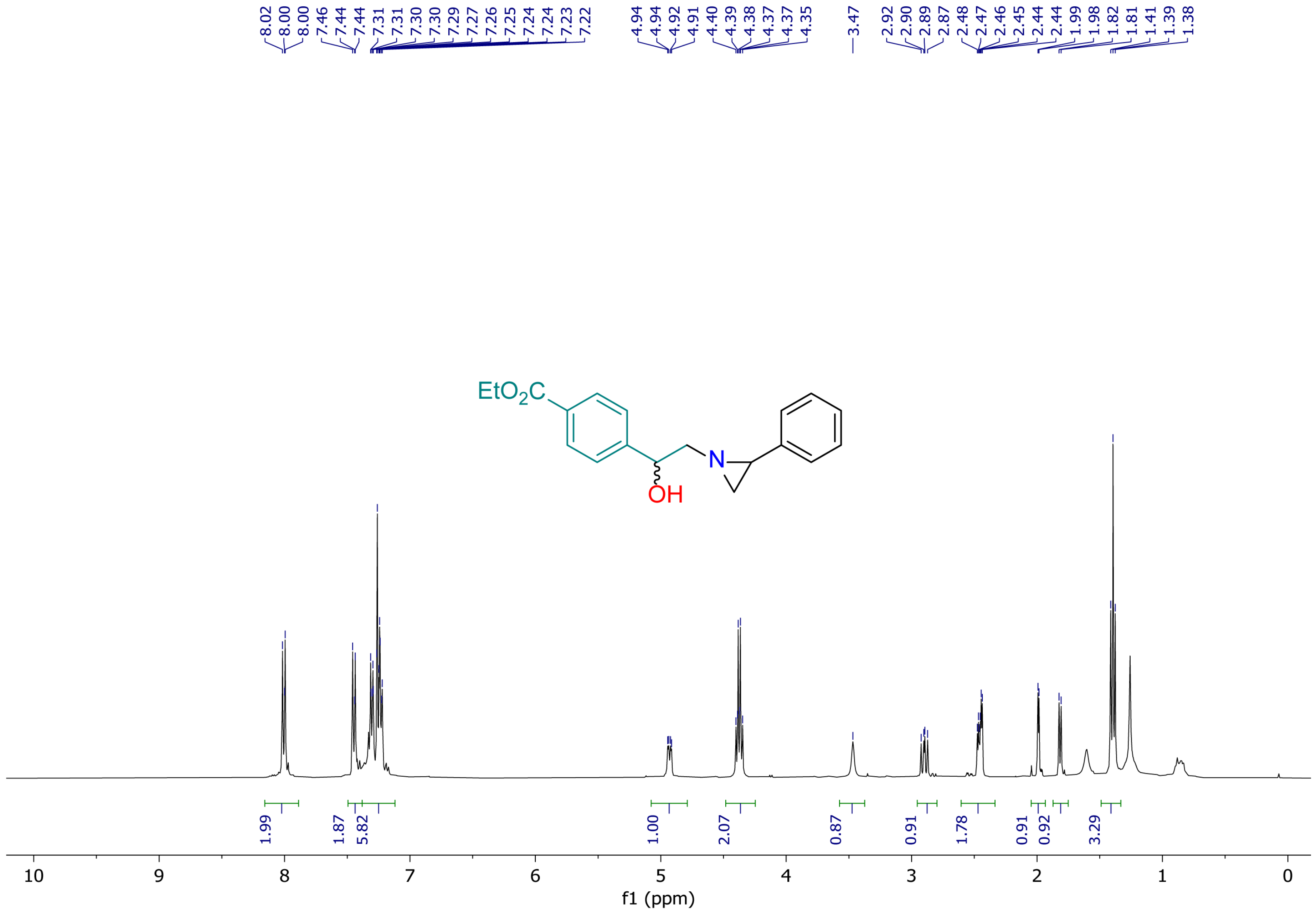

Figure S38. ^{1}H NMR spectrum of ethyl 4-(1-hydroxy-2-(2-phenylaziridin-1-yl)ethyl)benzoate (**5g**) in $CDCl_3$ (400 MHz) at 23 °C.

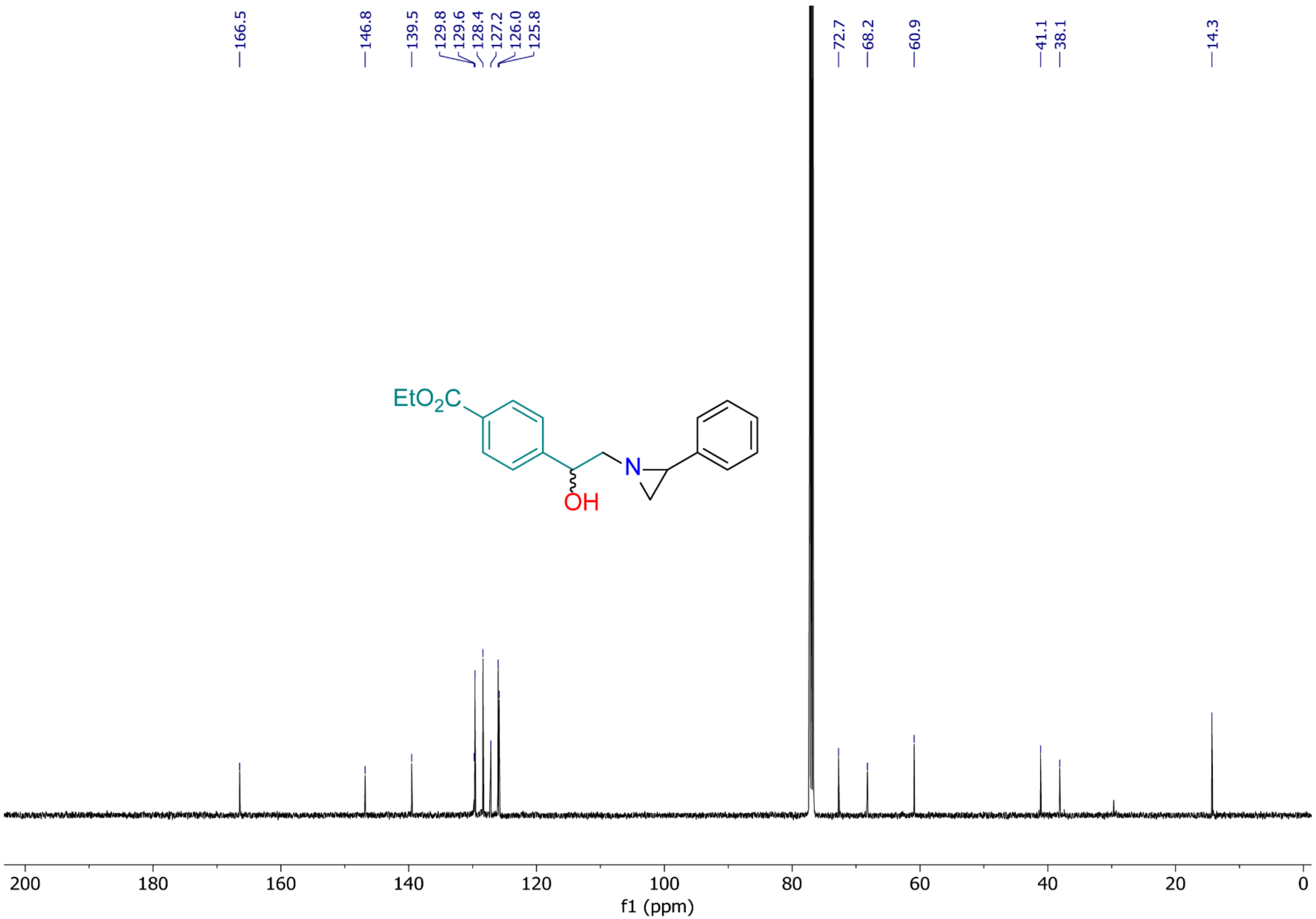

Figure S39. ^{13}C NMR spectrum of ethyl 4-(1-hydroxy-2-(2-phenylaziridin-1-yl)ethyl)benzoate (**5g**) in $CDCl_3$ (101 MHz) at 23 °C.

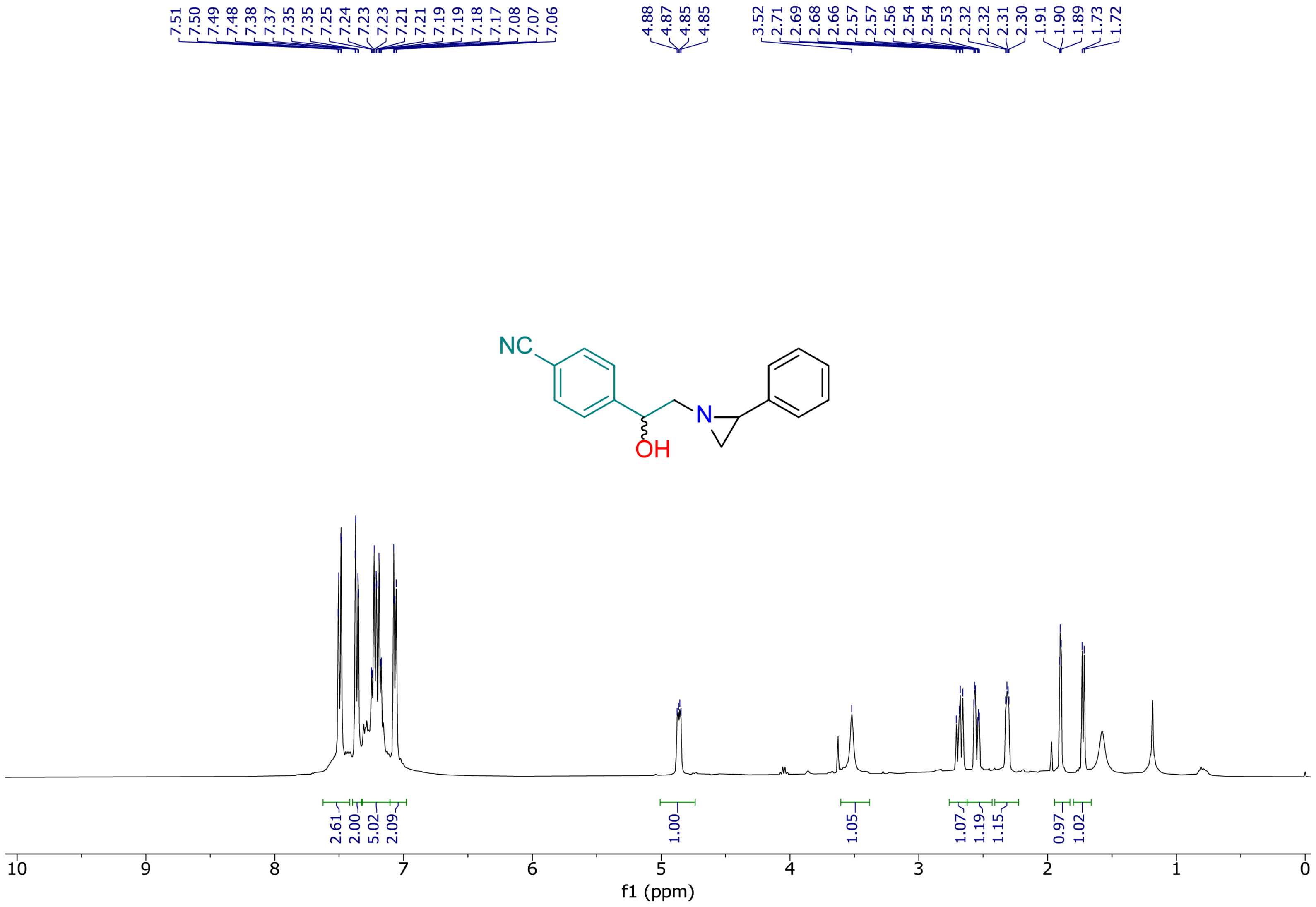

Figure S40. ^{1}H NMR spectrum of 4-(1-hydroxy-2-(2-phenylaziridin-1-yl)ethyl)benzonitrile (**5h**) in $CDCl_3$ (400 MHz) at 23 °C.

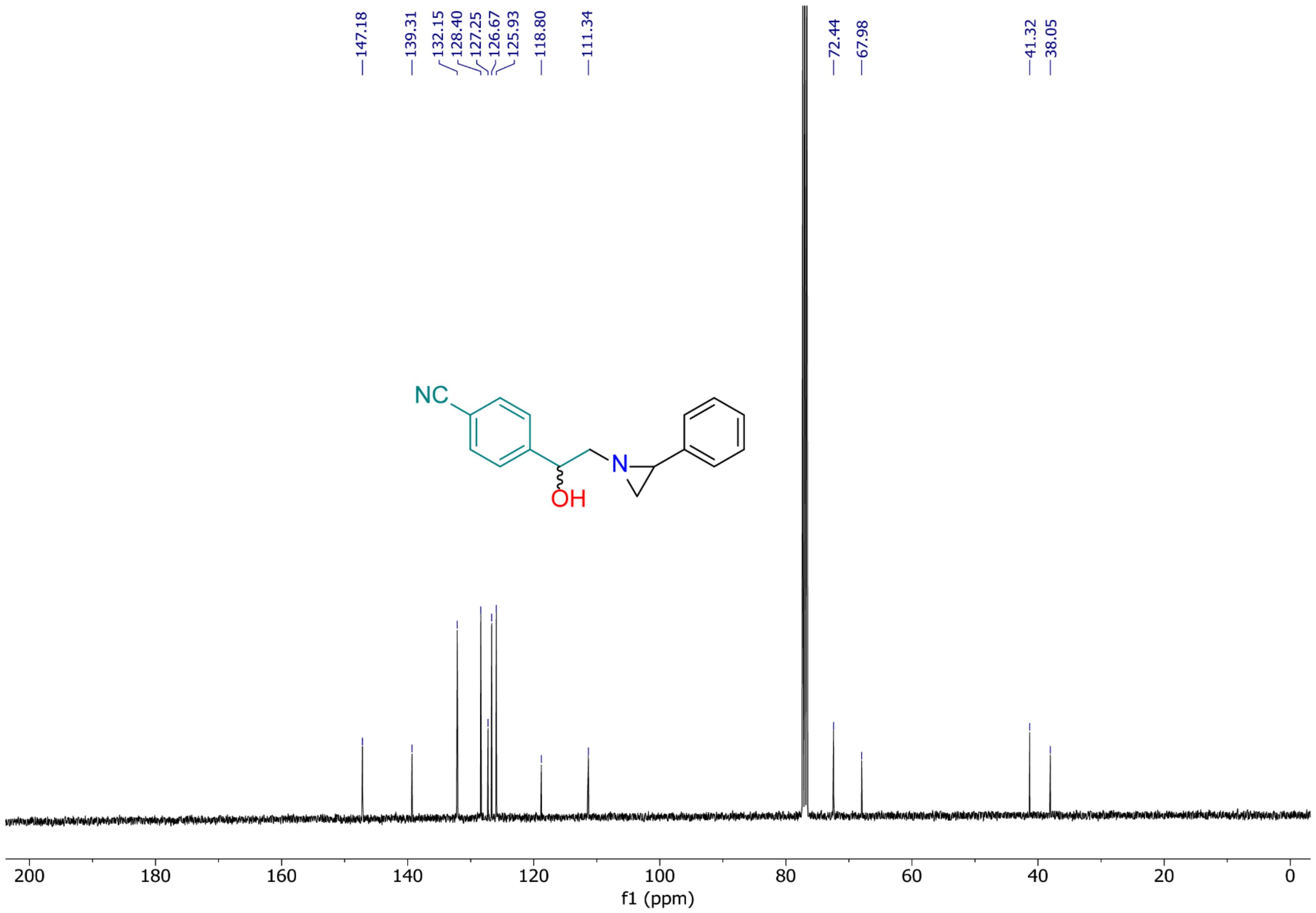

Figure S41. ^{13}C NMR spectrum of 4-(1-hydroxy-2-(2-phenylaziridin-1-yl)ethyl)benzonitrile (**5h**) in $CDCl_3$ in $CDCl_3$ (101 MHz) at 23 °C.

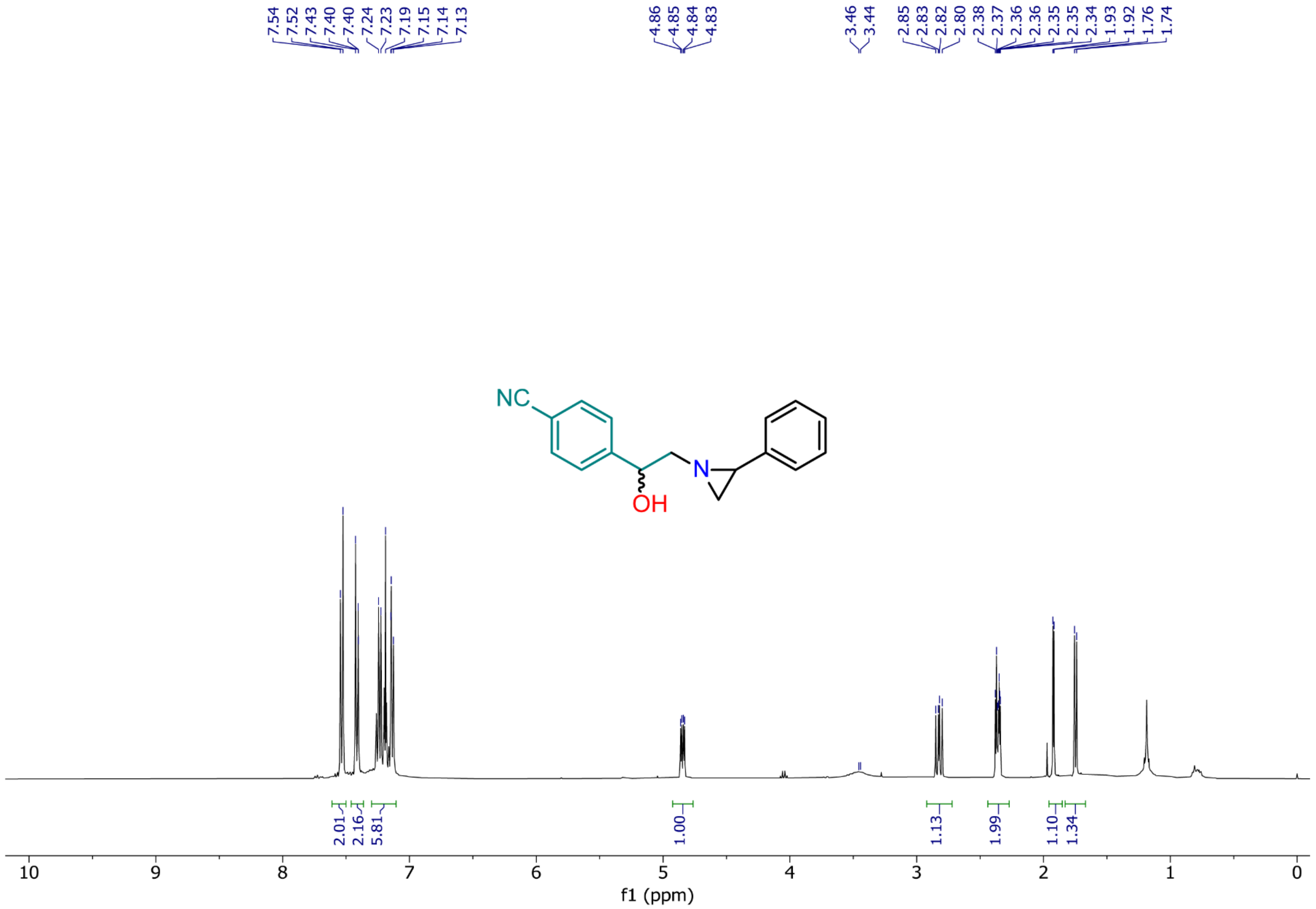

Figure S42. ^{1}H NMR spectrum of 4-(1-hydroxy-2-(2-phenylaziridin-1-yl)ethyl)benzonitrile (**5h**) in $CDCl_3$ (400 MHz) at 23 °C.

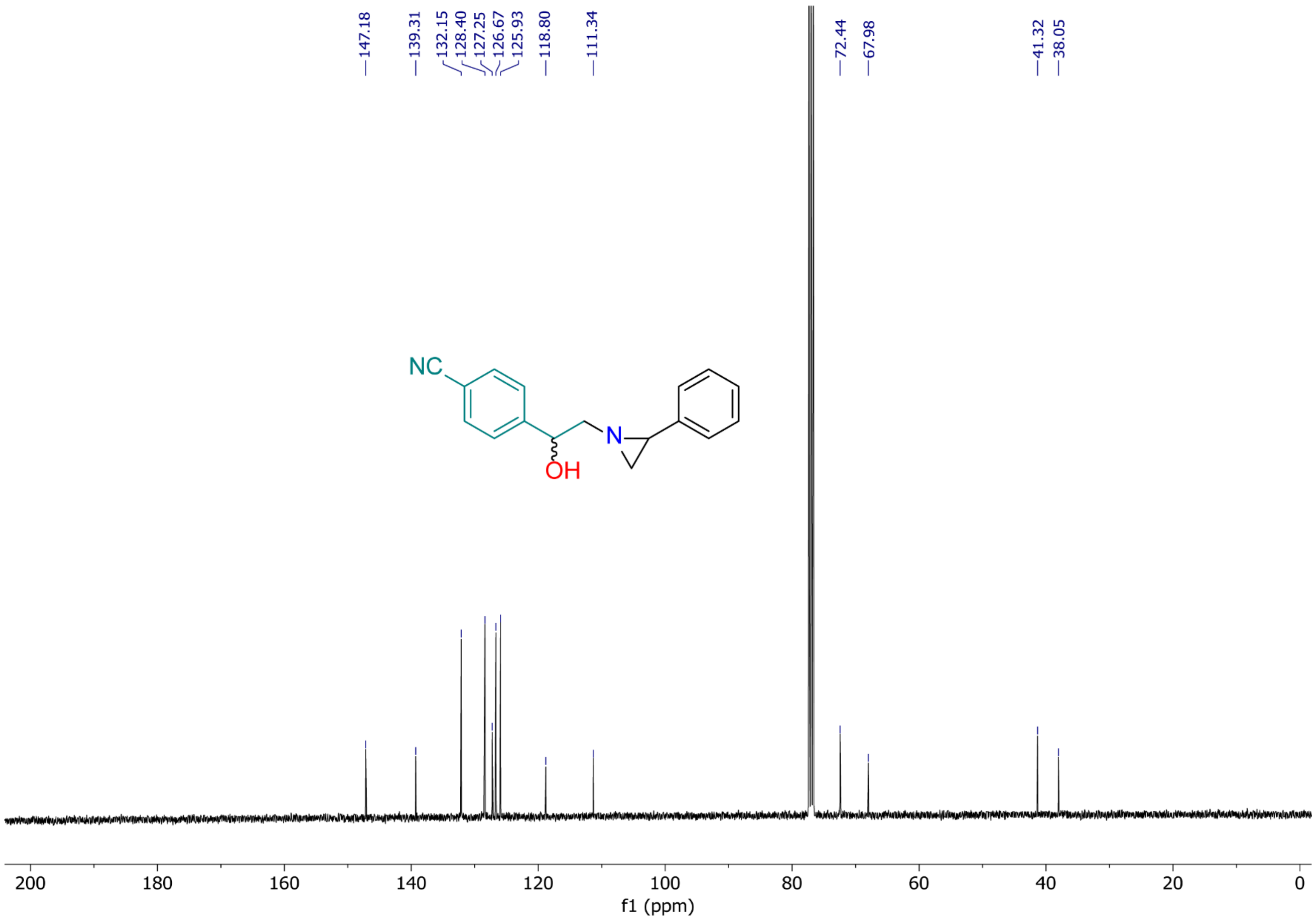

Figure S43. ^{13}C NMR spectrum of 4-(1-hydroxy-2-(2-phenylaziridin-1-yl)ethyl)benzonitrile (**5h**) in $CDCl_3$ in $CDCl_3$ (101 MHz) at 23 °C.

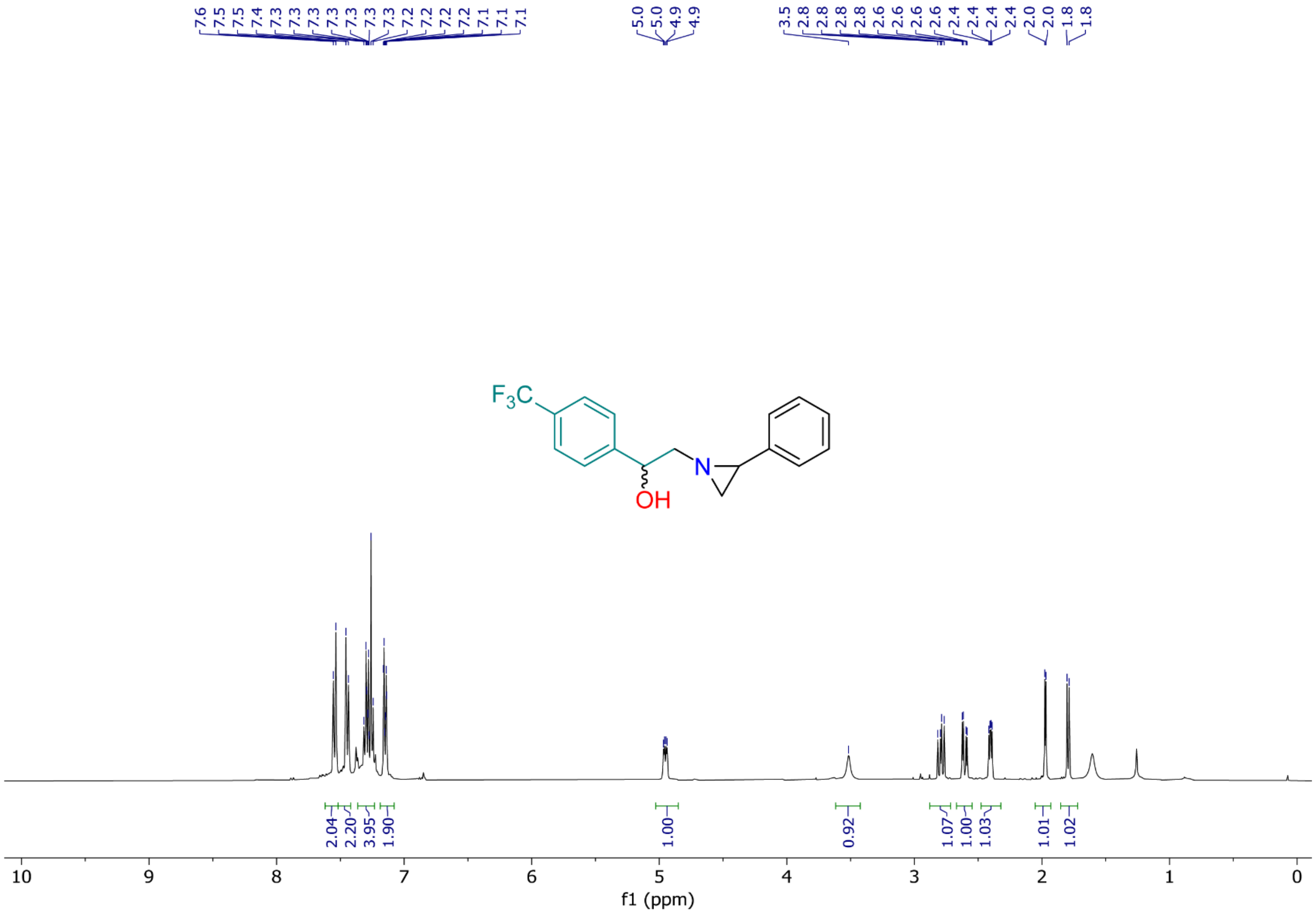

Figure S44. ^{1}H NMR spectrum of 2-(2-phenylaziridin-1-yl)-1-(4-(trifluoromethyl)phenyl)ethan-1-ol (**5i**) in $CDCl_3$ (400 MHz) at 23 °C.

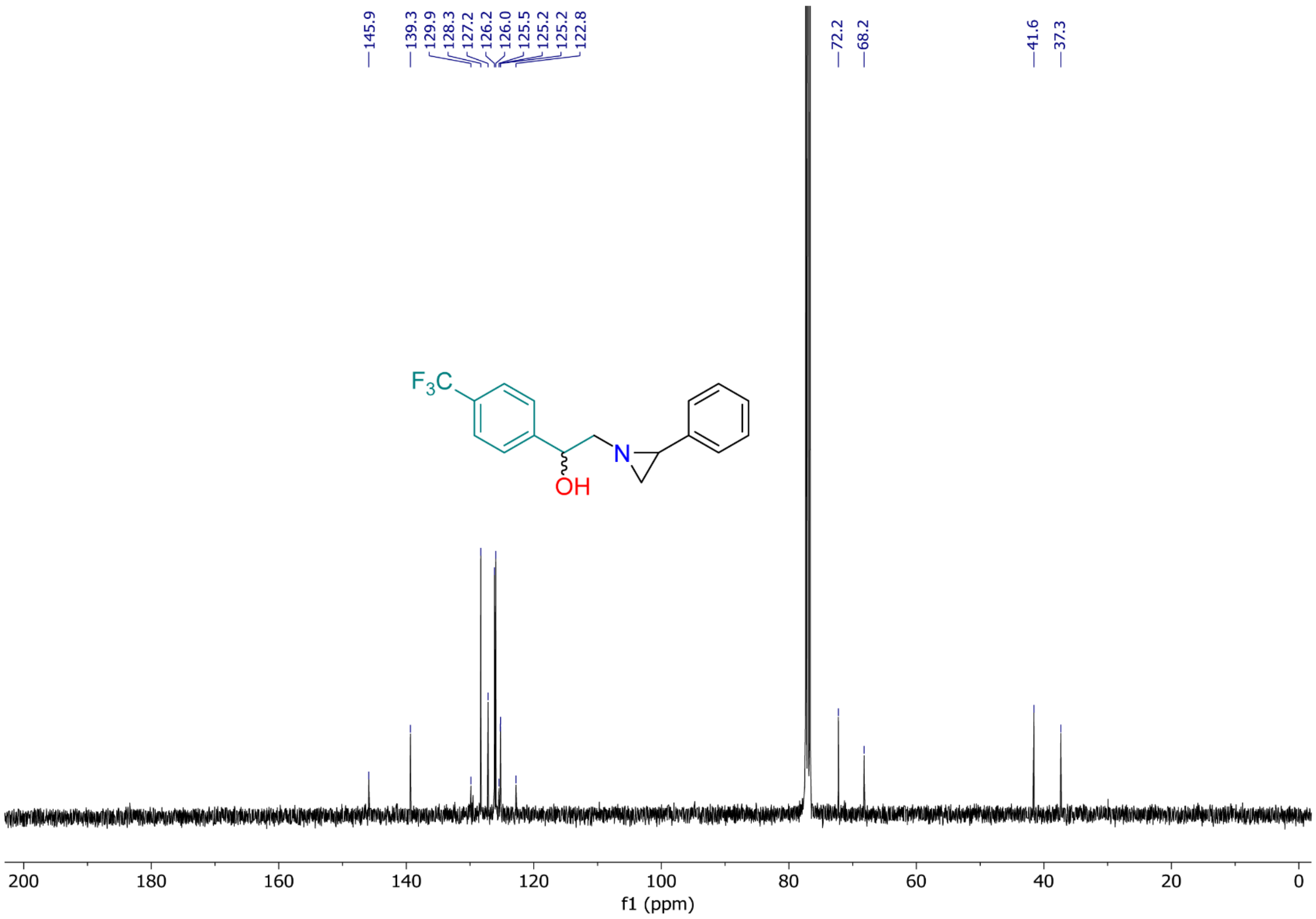

Figure S45. ^{13}C NMR spectrum of 2-(2-phenylaziridin-1-yl)-1-(4-(trifluoromethyl)phenyl)ethan-1-ol (**5i**) in $CDCl_3$ (101 MHz) at 23 °C.

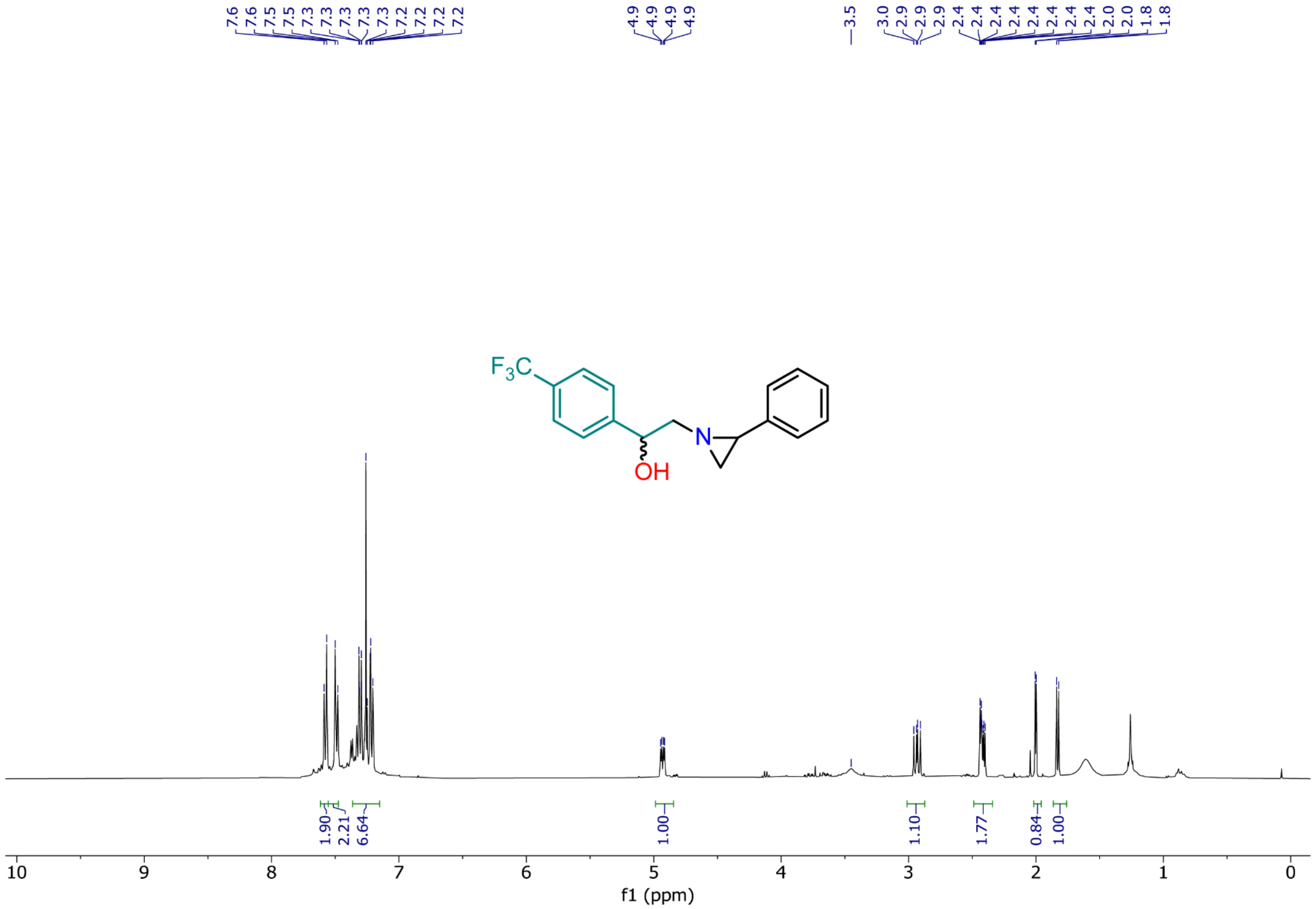

Figure S46. ^{1}H NMR spectrum of 2-(2-phenylaziridin-1-yl)-1-(4-(trifluoromethyl)phenyl)ethan-1-ol (**5i**) in $CDCl_3$ (400 MHz) at 23 °C.

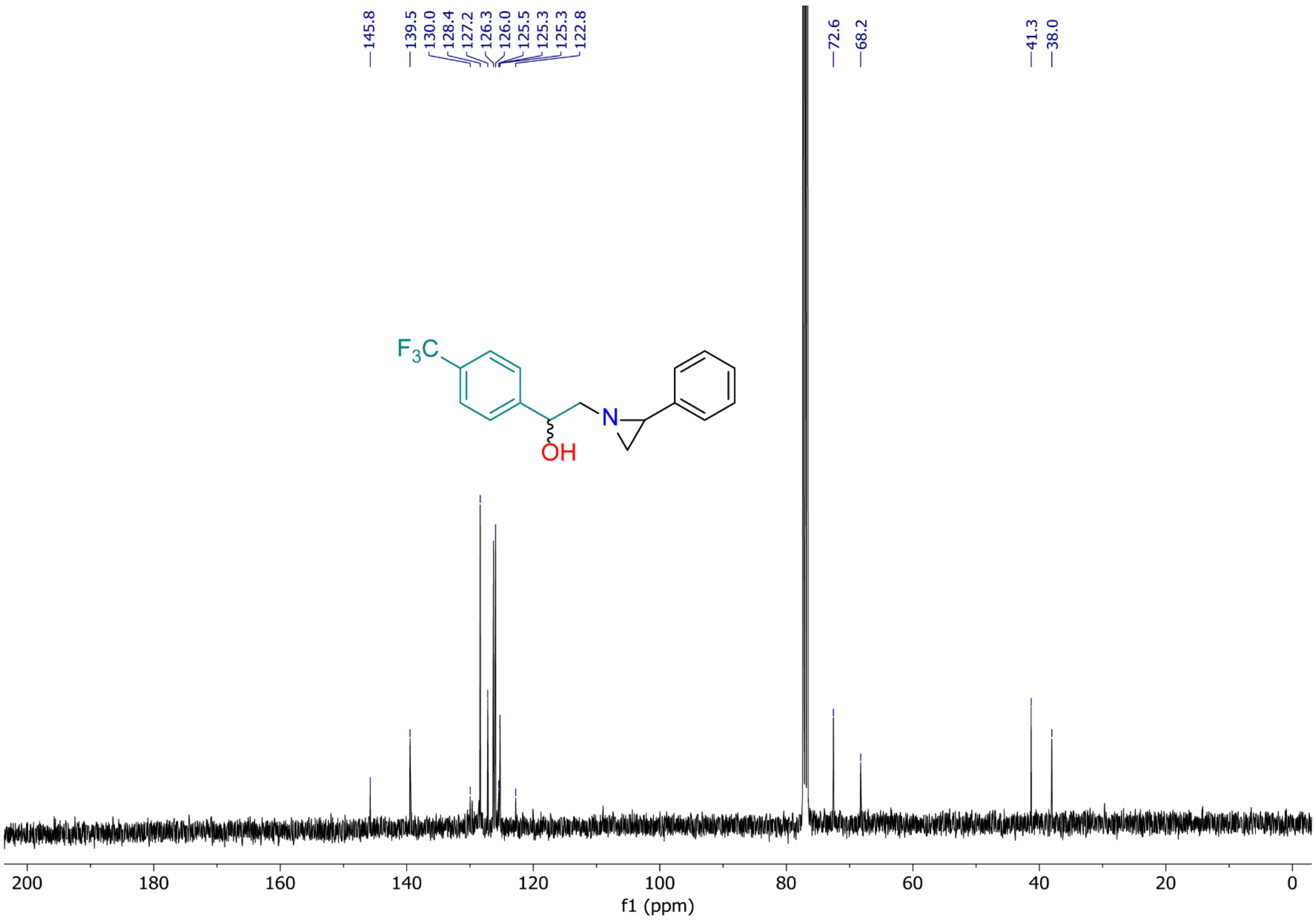

Figure S47. ^{13}C NMR spectrum of 2-(2-phenylaziridin-1-yl)-1-(4-(trifluoromethyl)phenyl)ethan-1-ol (**5i**) in $CDCl_3$ (101 MHz) at 23 °C.

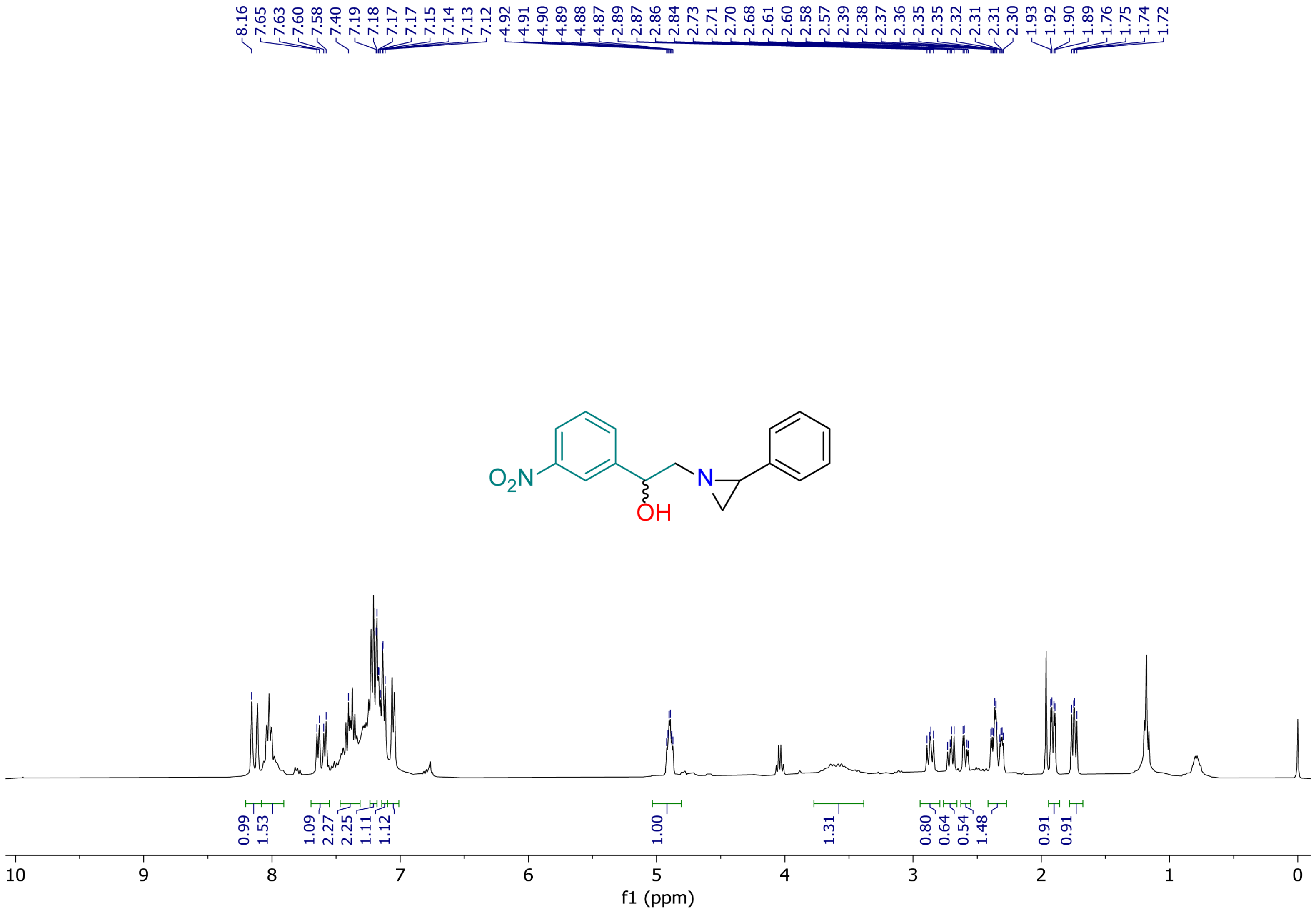

Figure S48. ^{1}H NMR spectrum of 1-(3-nitrophenyl)-2-(2-phenylaziridin-1-yl)ethan-1-ol (**5j**) in $CDCl_3$ (400 MHz) at 23 °C.

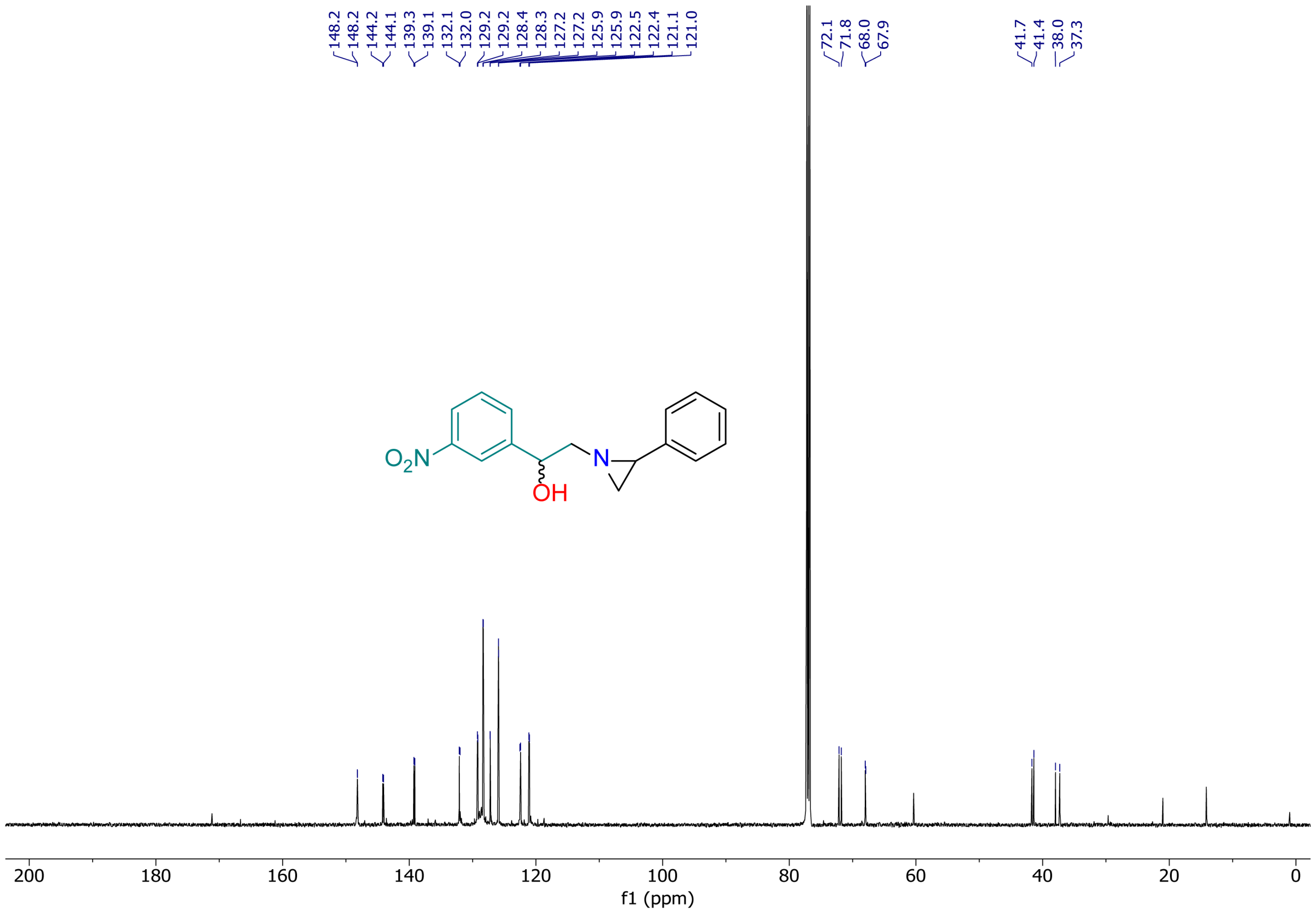

Figure S49. ^{13}C NMR spectrum of 1-(3-nitrophenyl)-2-(2-phenylaziridin-1-yl)ethan-1-ol (**5j**) in $CDCl_3$ (126 MHz) at 23 °C.

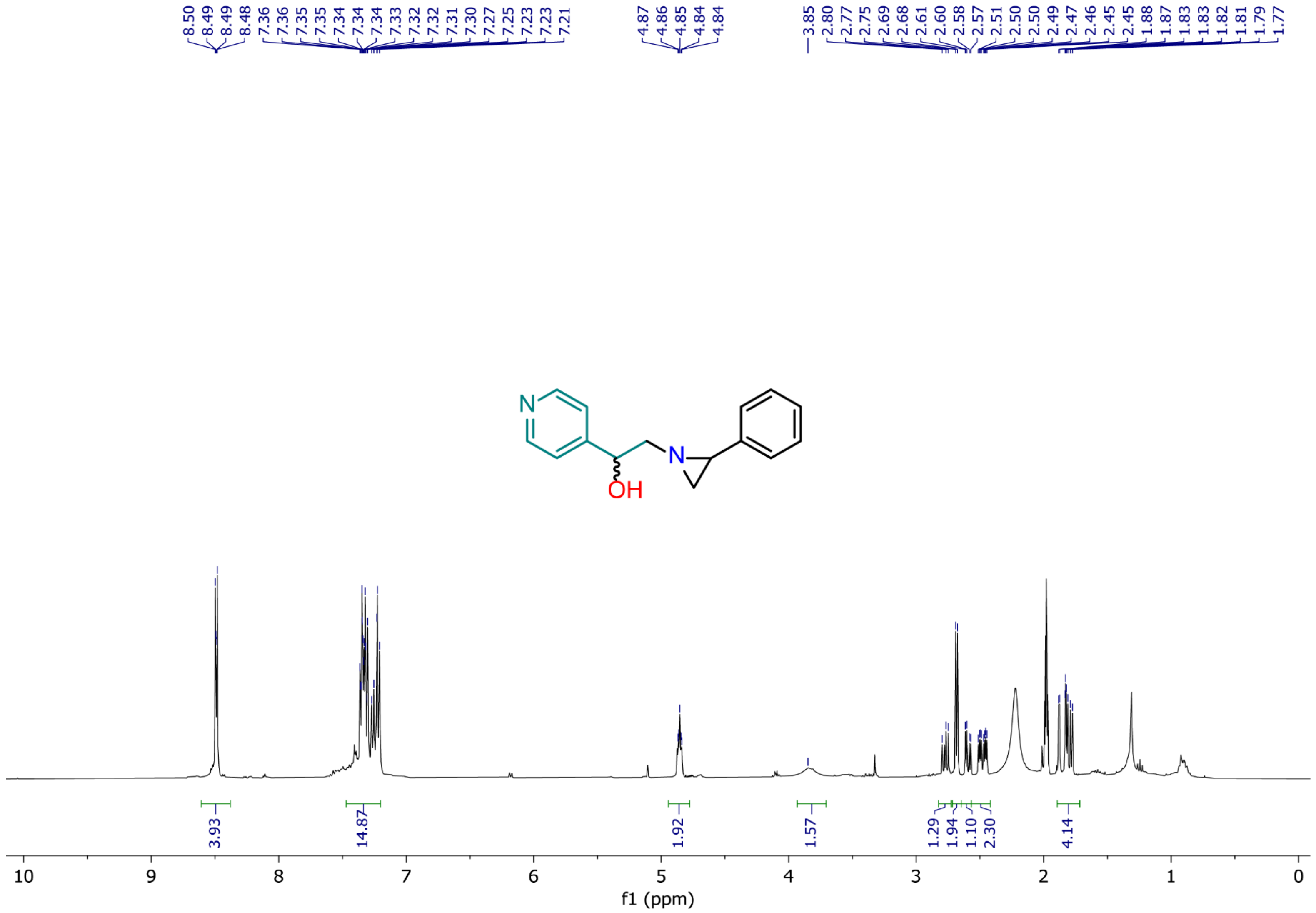

Figure S50. ^{1}H NMR spectrum of 2-(2-phenylaziridin-1-yl)-1-(pyridin-2-yl)ethan-1-ol (**5k**) in CD_3CN (400 MHz) at 23 °C.

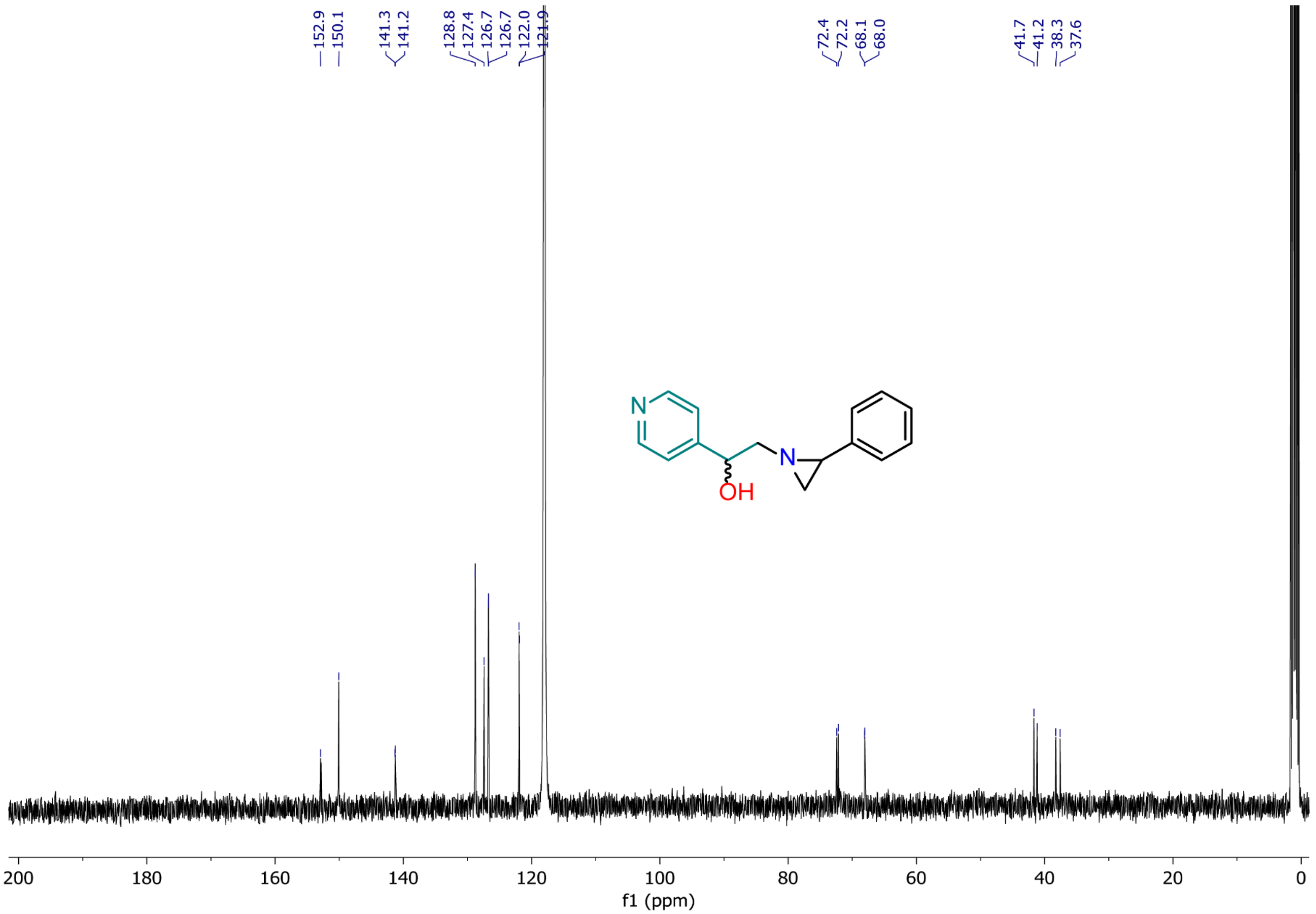

Figure S51. ^{13}C NMR spectrum of 2-(2-phenylaziridin-1-yl)-1-(pyridin-2-yl)ethan-1-ol (**5k**) in CD_3CN (126 MHz) at 23 °C.

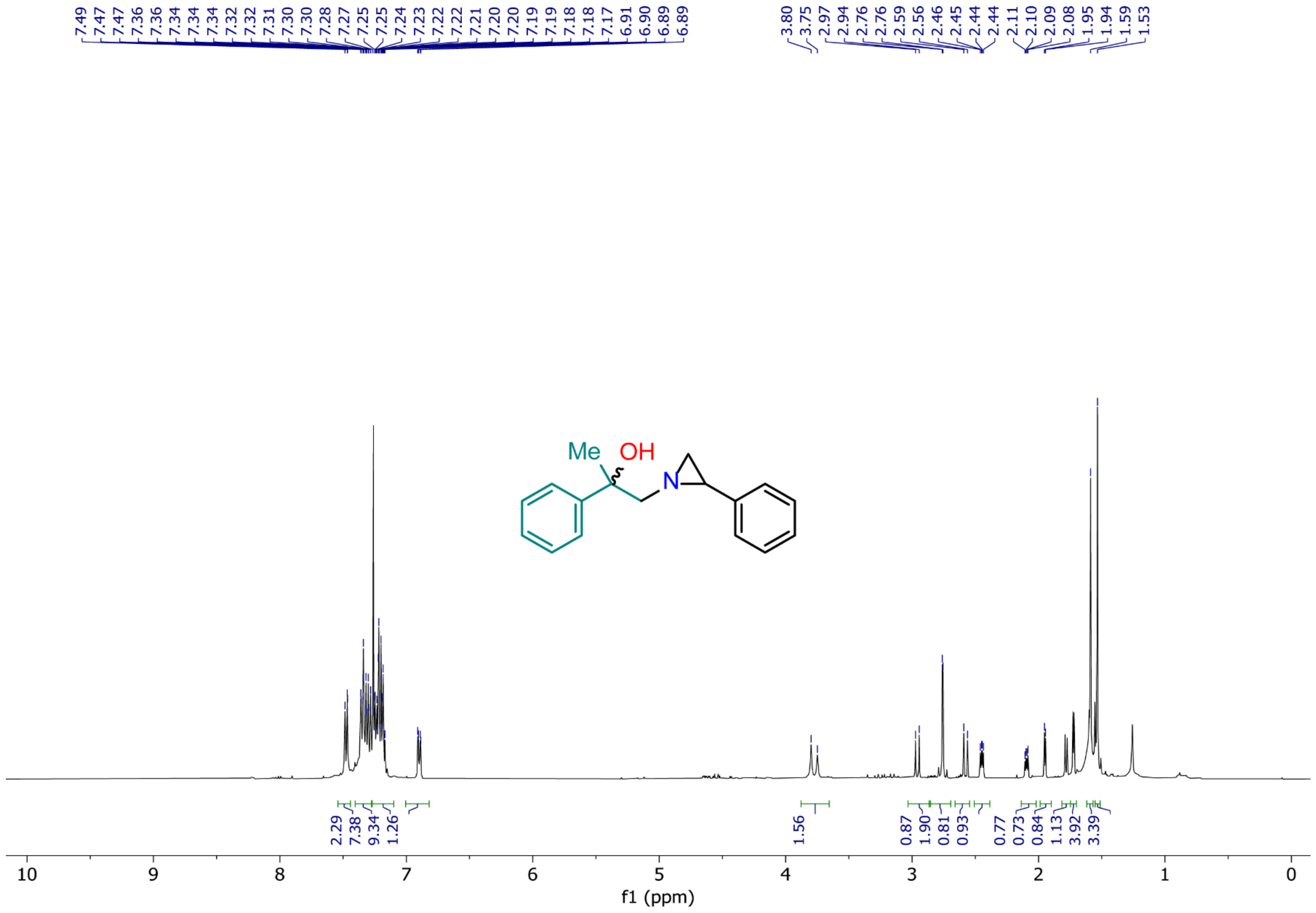

Figure S52. ^{1}H NMR spectrum of 2-phenyl-1-(2-phenylaziridin-1-yl)propan-2-ol (**5l**) in $CDCl_3$ (400 MHz) at 23 °C.

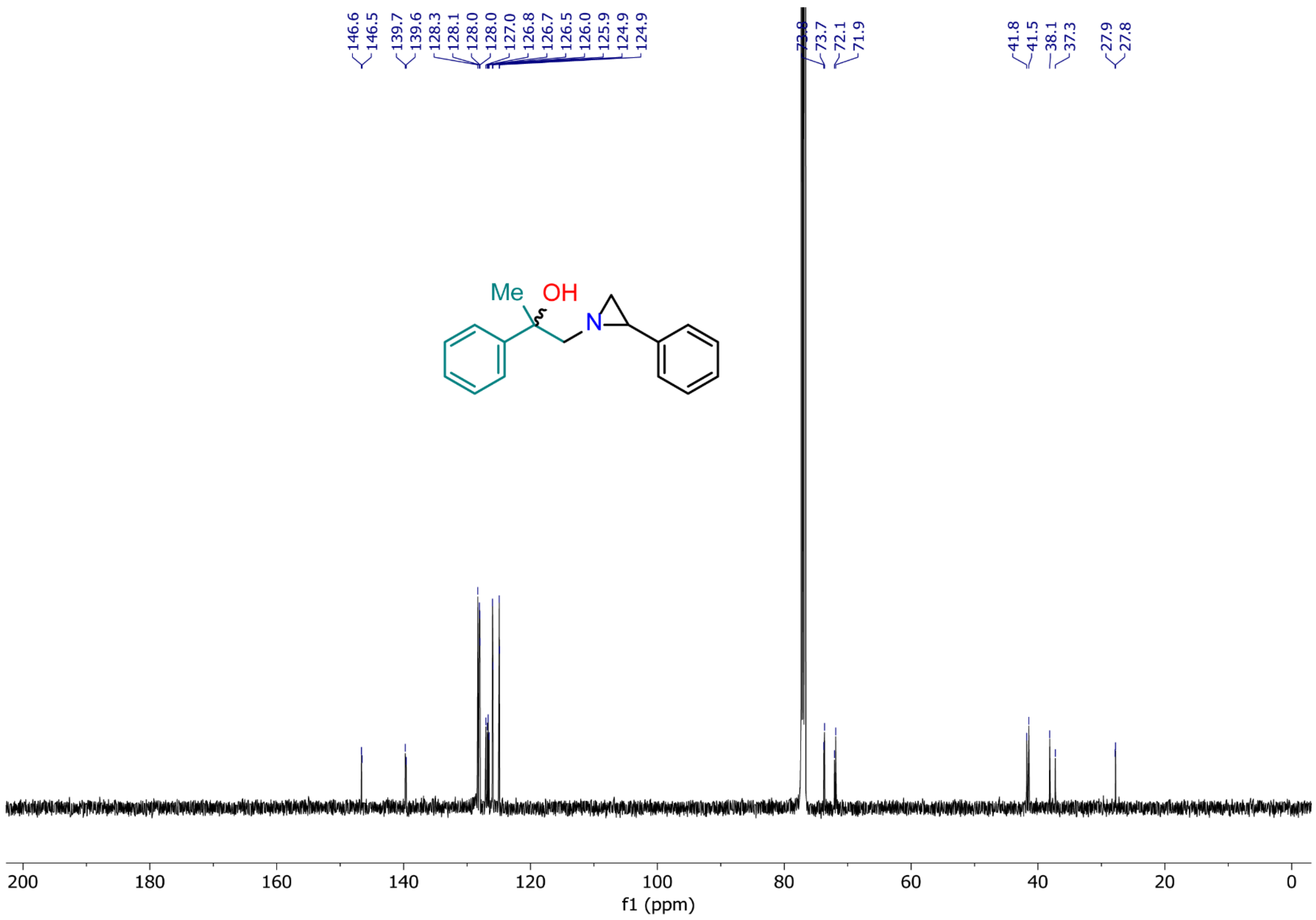

Figure S53. ^{13}C NMR spectrum of 2-phenyl-1-(2-phenylaziridin-1-yl)propan-2-ol (**5l**) in $CDCl_3$ (126 MHz) at 23 °C.

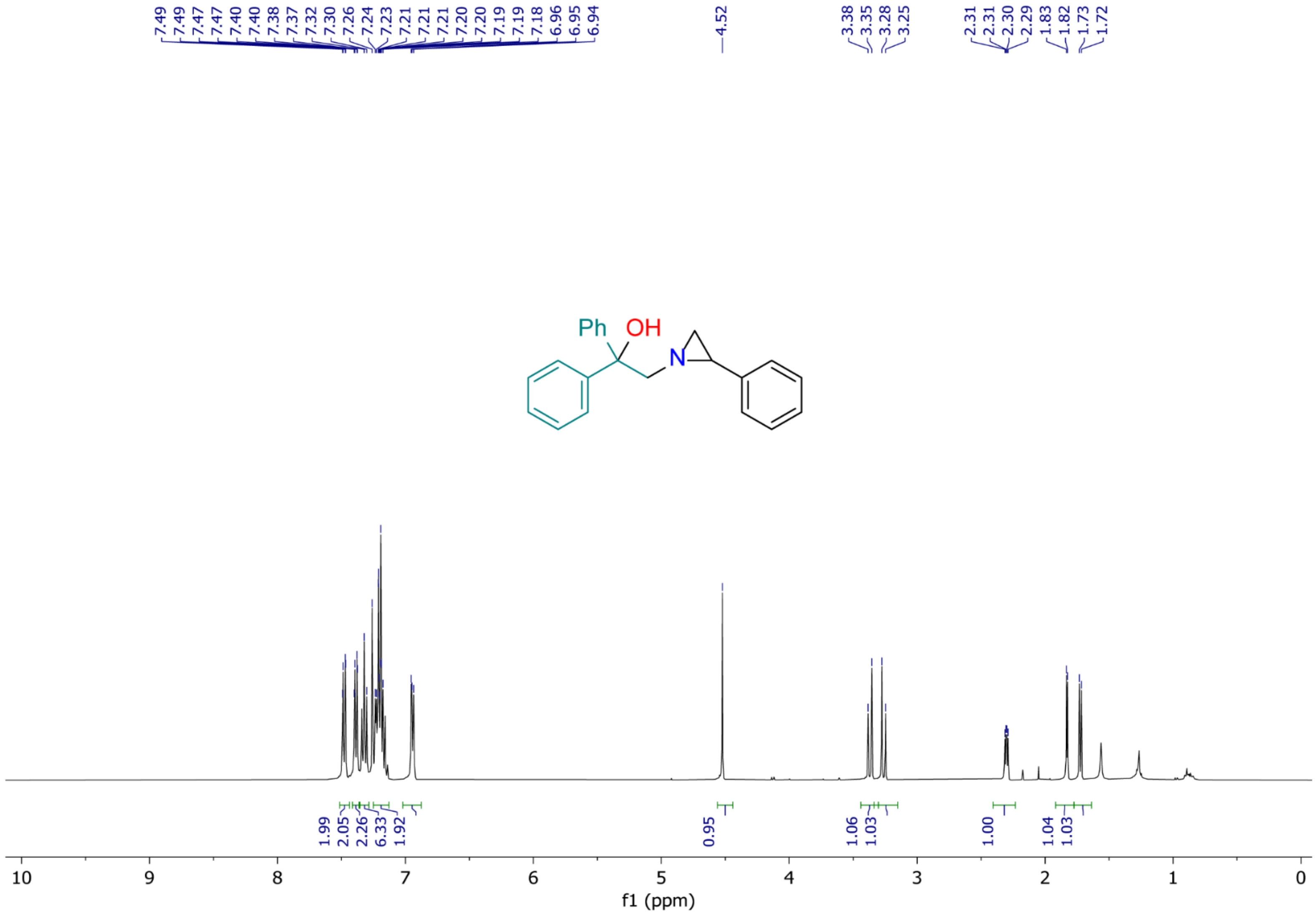

Figure S54. ^{1}H NMR spectrum of 1,1-diphenyl-2-(2-phenylaziridin-1-yl)ethan-1-ol (**5m**) in $CDCl_3$ (400 MHz) at 23 °C.

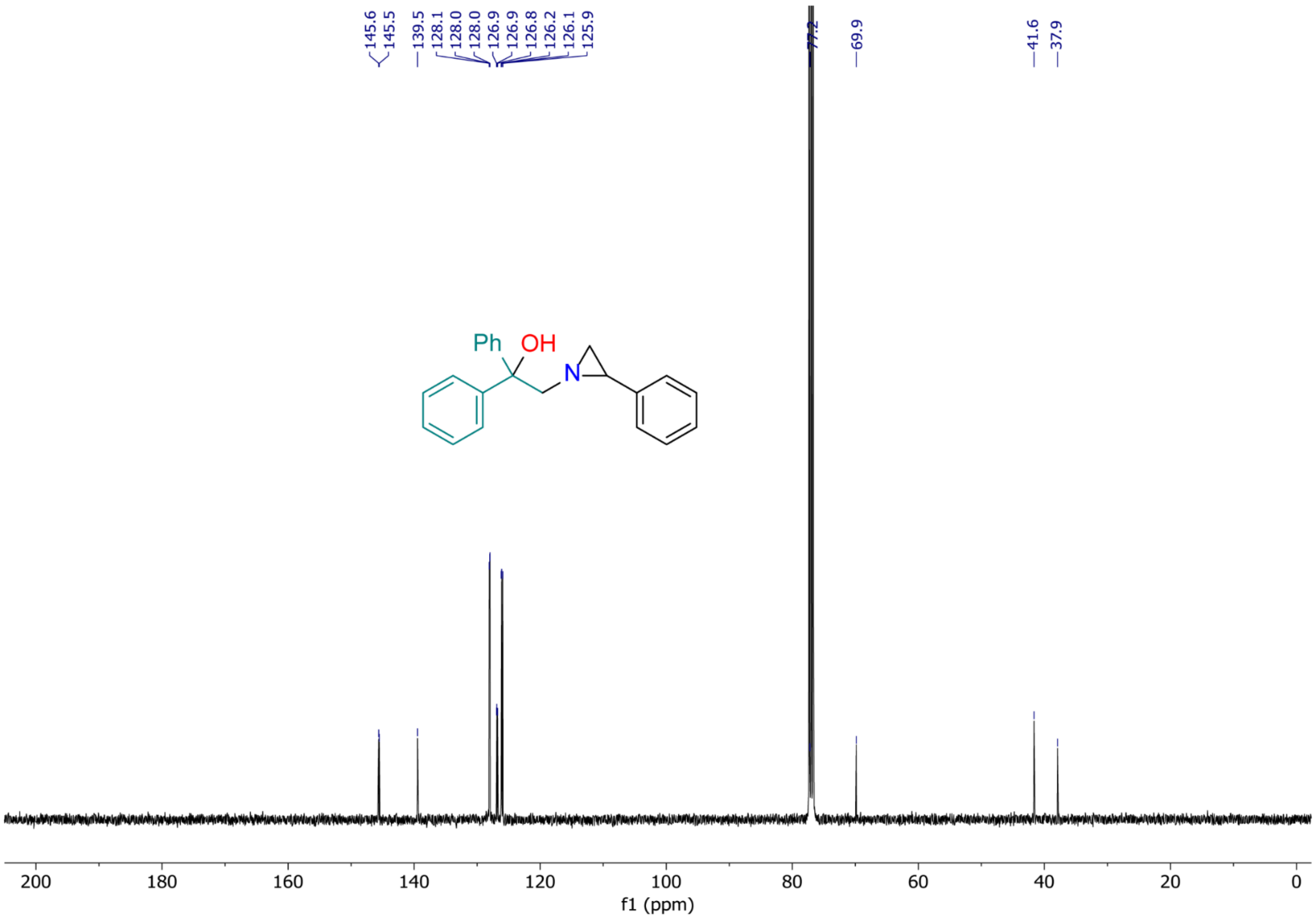

Figure S55. ^{13}C NMR spectrum of 1,1-diphenyl-2-(2-phenylaziridin-1-yl)ethan-1-ol (**5m**) in $CDCl_3$ (101 MHz) at 23 °C.

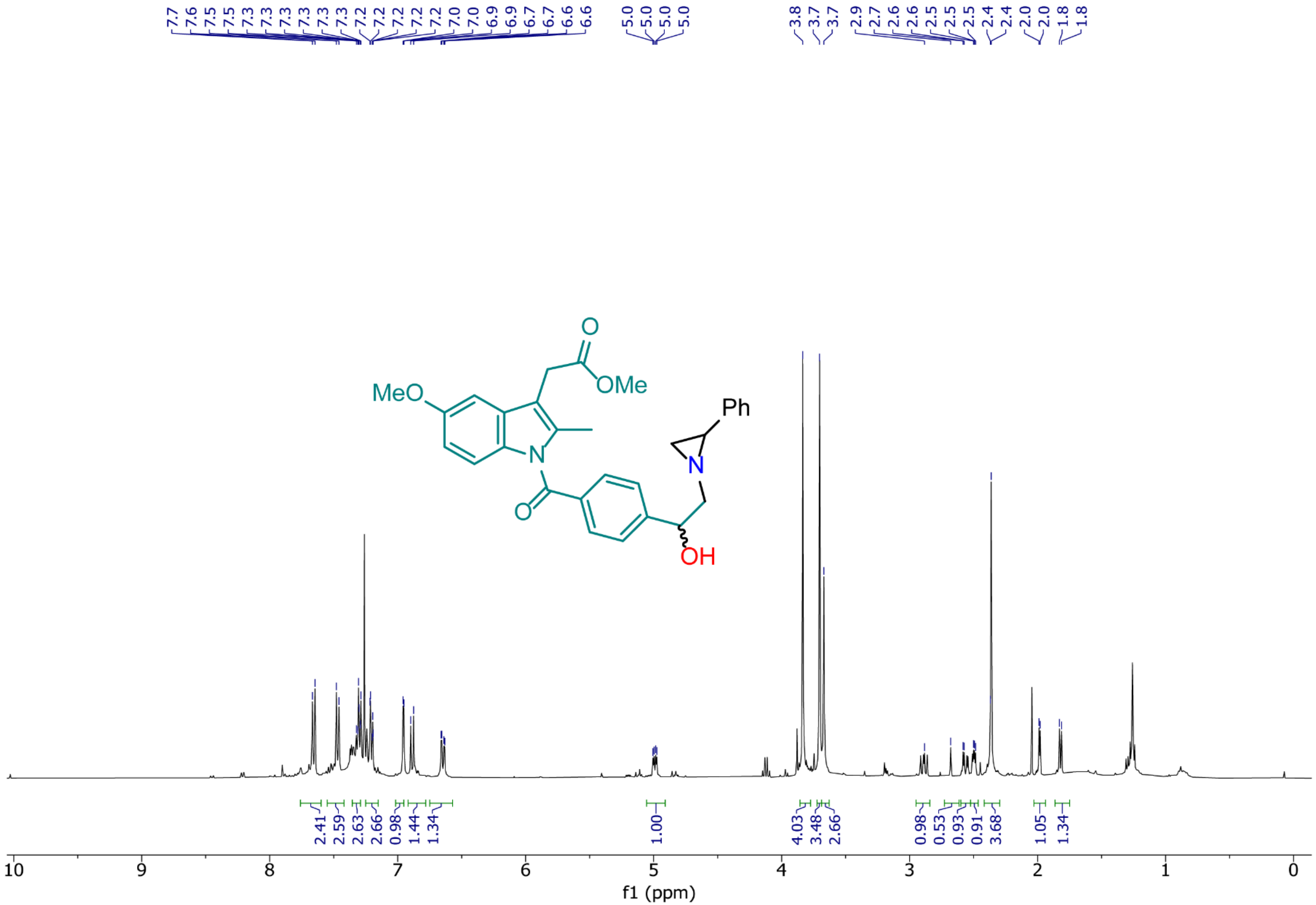

Figure S56. ^{1}H NMR spectrum of methyl 2-(1-(4-(1-hydroxy-2-(2-phenylaziridin-1-yl)ethyl)benzoyl)-5-methoxy-2-methyl-1H-indol-3-yl)acetate (**5n**) in $CDCl_3$ (400 MHz) at 23 °C.

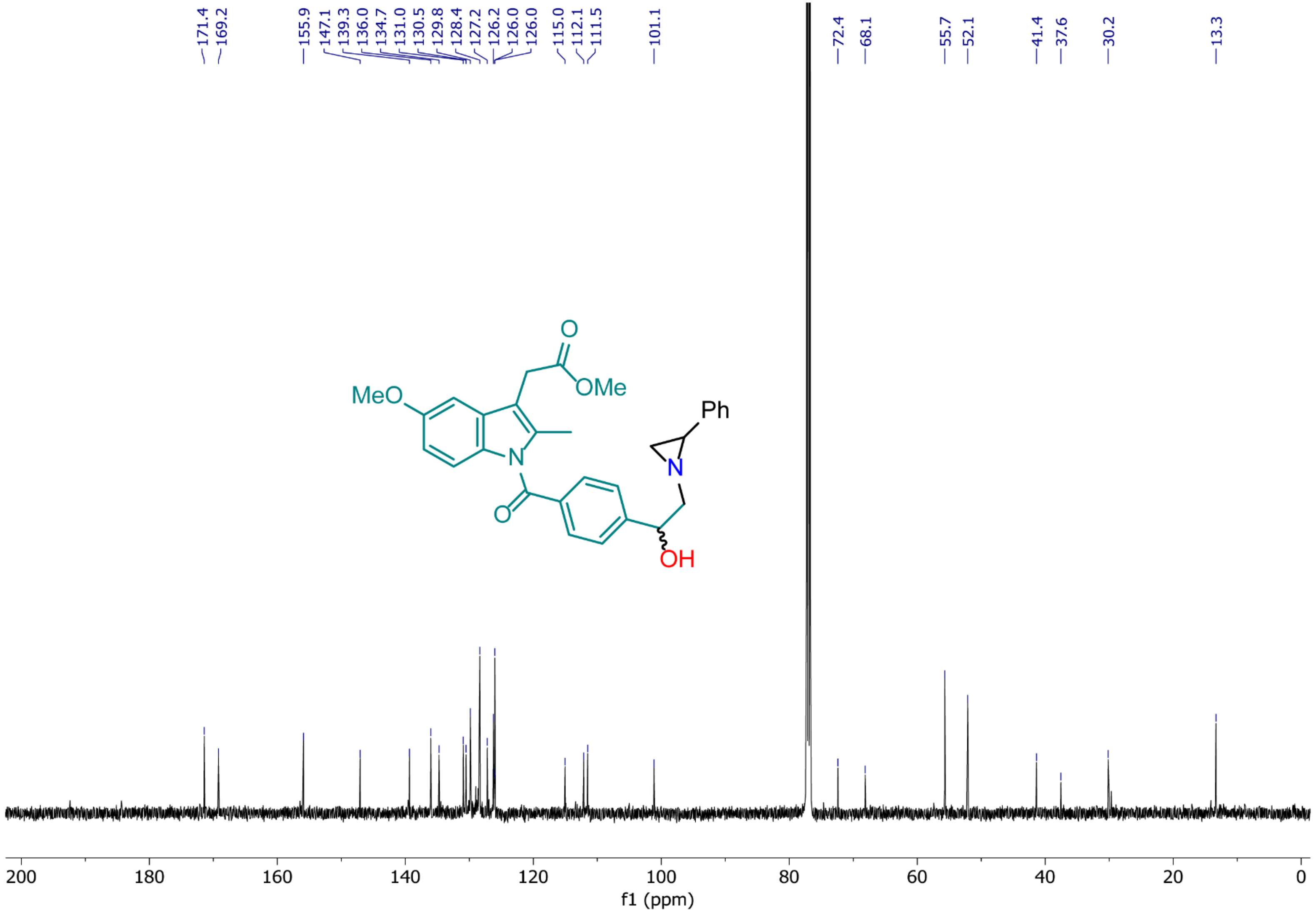

Figure S57. ^{13}C NMR spectrum of methyl 2-(1-(4-(1-hydroxy-2-(2-phenylaziridin-1-yl)ethyl)benzoyl)-5-methoxy-2-methyl-1H-indol-3-yl)acetate (**5n**) in $CDCl_3$ (126 MHz) at 23 °C.

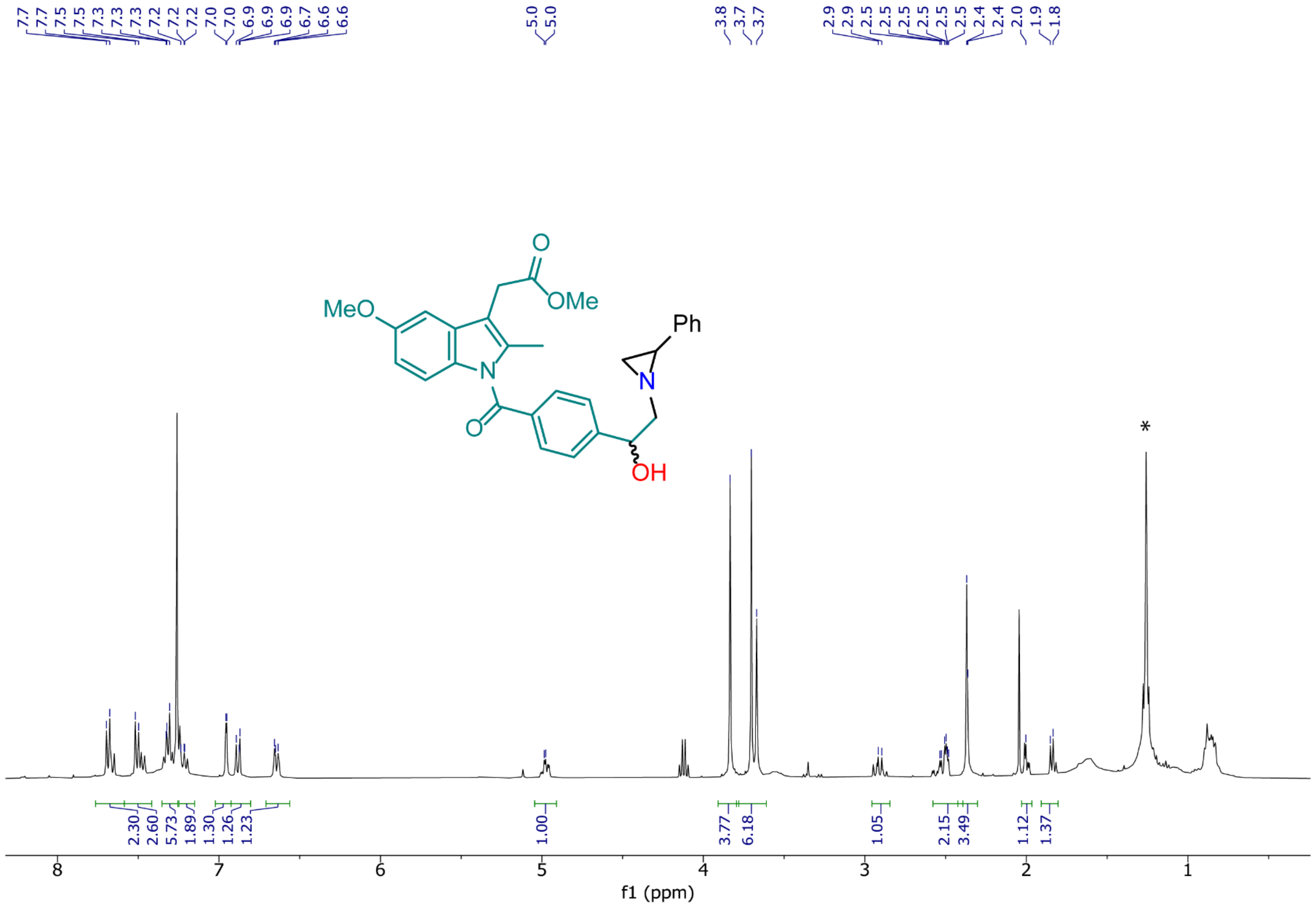

Figure S58. ^{1}H NMR spectrum of methyl 2-(1-(4-(1-hydroxy-2-(2-phenylaziridin-1-yl)ethyl)benzoyl)-5-methoxy-2-methyl-1H-indol-3-yl)acetate (**5n**) in $CDCl_3$ (400 MHz) at 23 °C.

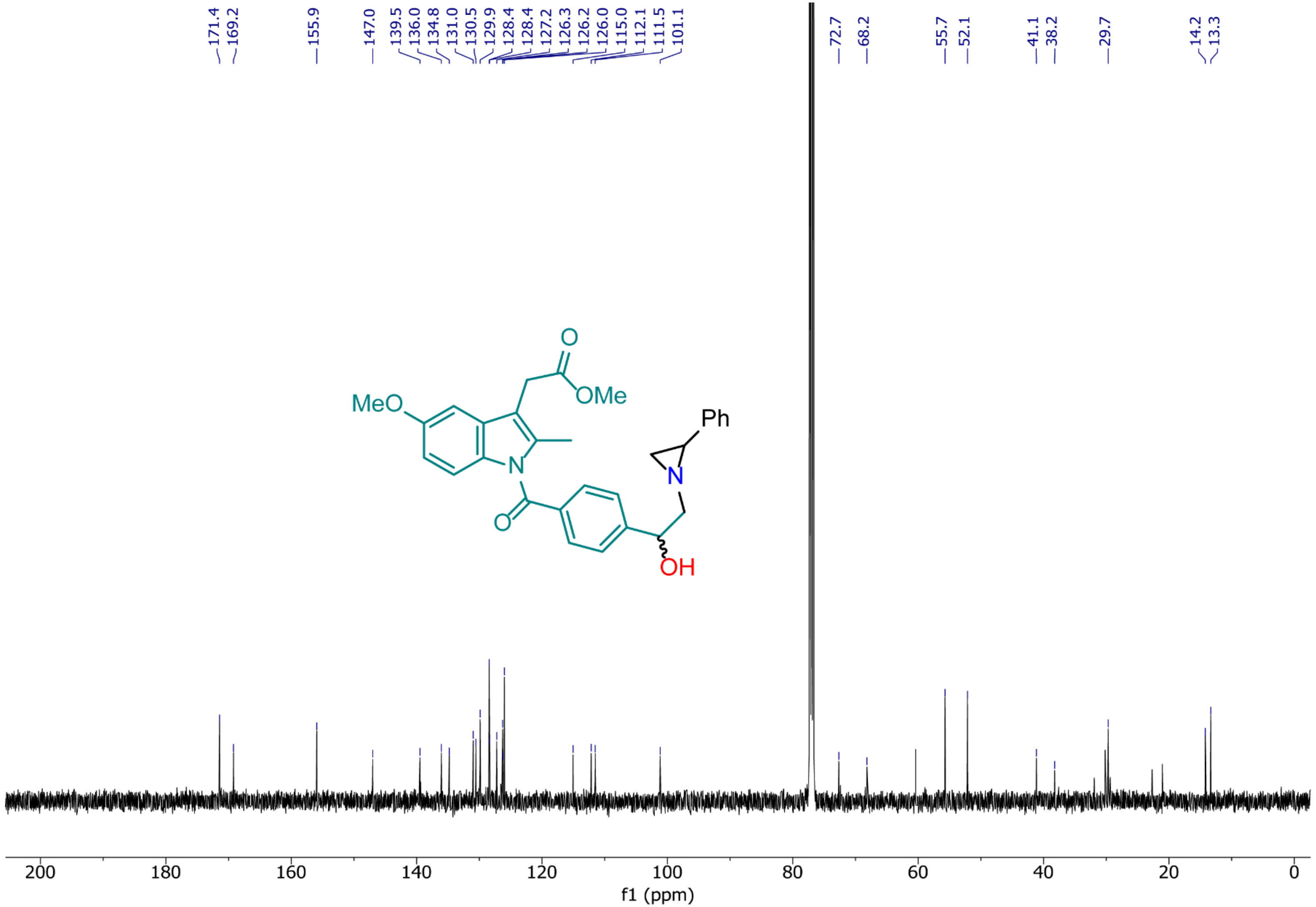

Figure S59. ^{13}C NMR spectrum of methyl 2-(1-(4-(1-hydroxy-2-(2-phenylaziridin-1-yl)ethyl)benzoyl)-5-methoxy-2-methyl-1H-indol-3-yl)acetate (**5n**) in $CDCl_3$ (126 MHz) at 23 °C.

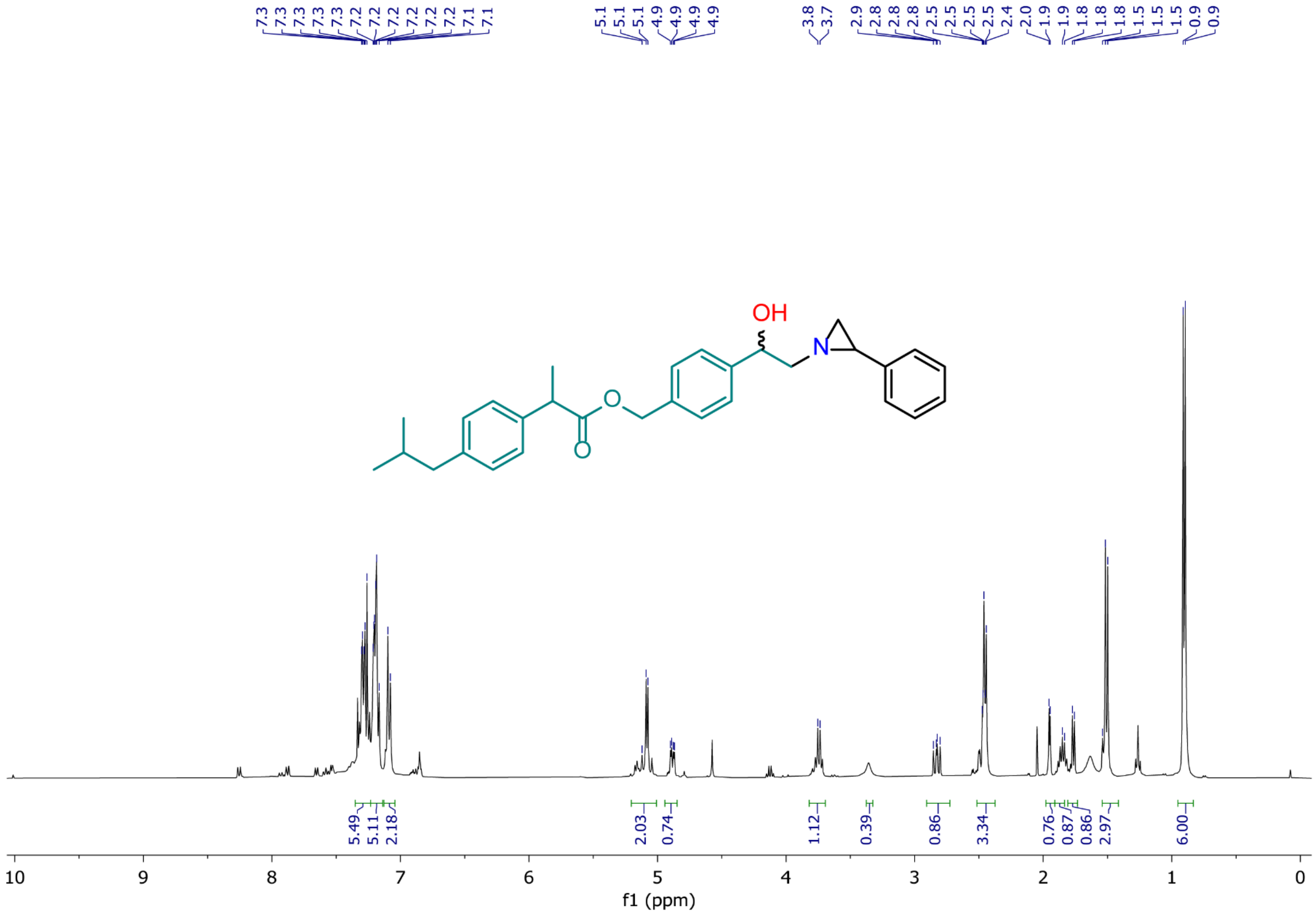

Figure S60. ^{1}H NMR spectrum of 4-(1-hydroxy-2-(2-phenylaziridin-1-yl)ethyl)benzyl 2-(4-isobutylphenyl)propanoate (**5o**) in $CDCl_3$ (400 MHz) at 23 °C.

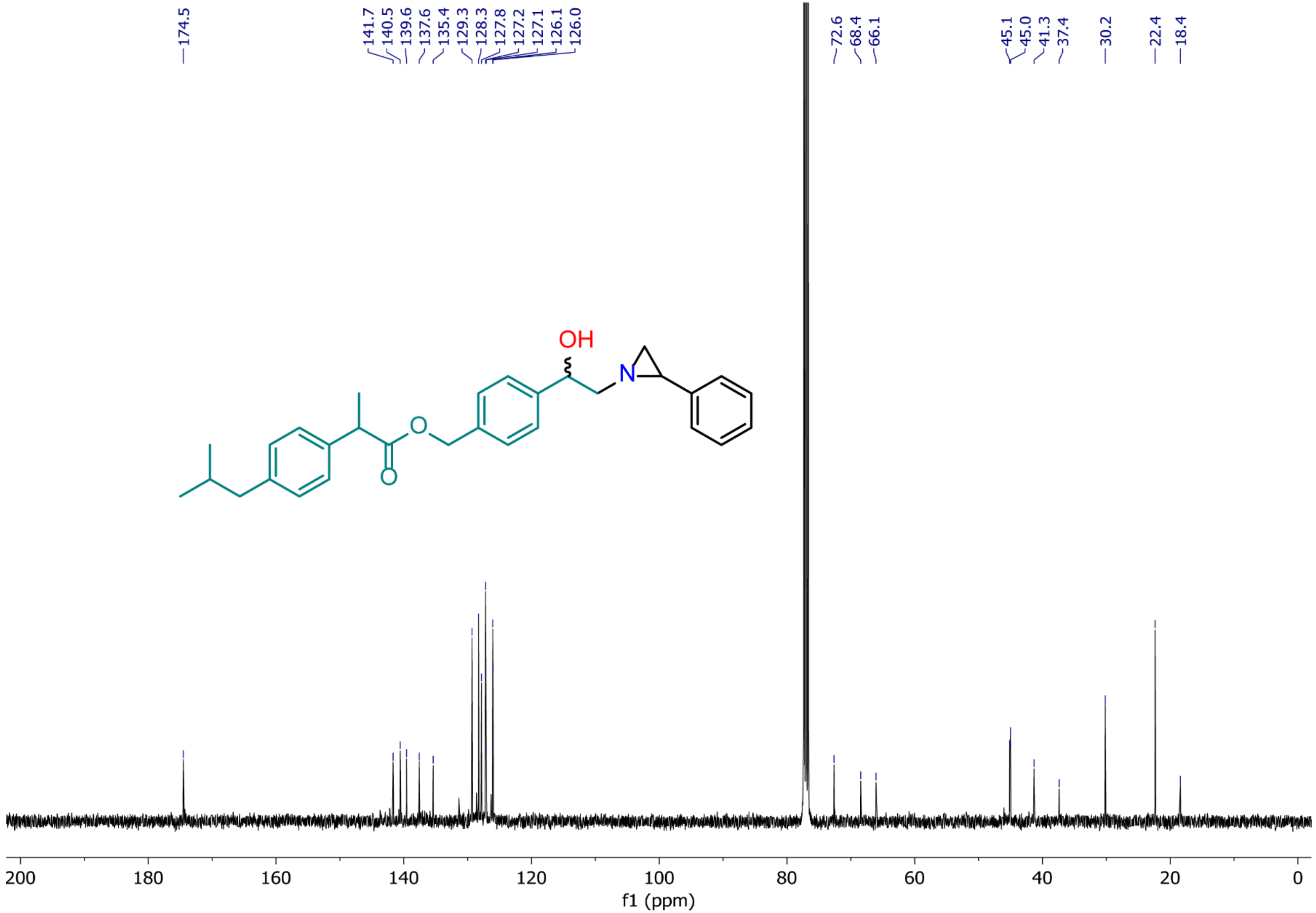

Figure S61. ^{13}C NMR spectrum of 4-(1-hydroxy-2-(2-phenylaziridin-1-yl)ethyl)benzyl 2-(4-isobutylphenyl)propanoate (**5o**) in $CDCl_3$ (101 MHz) at 23 °C.

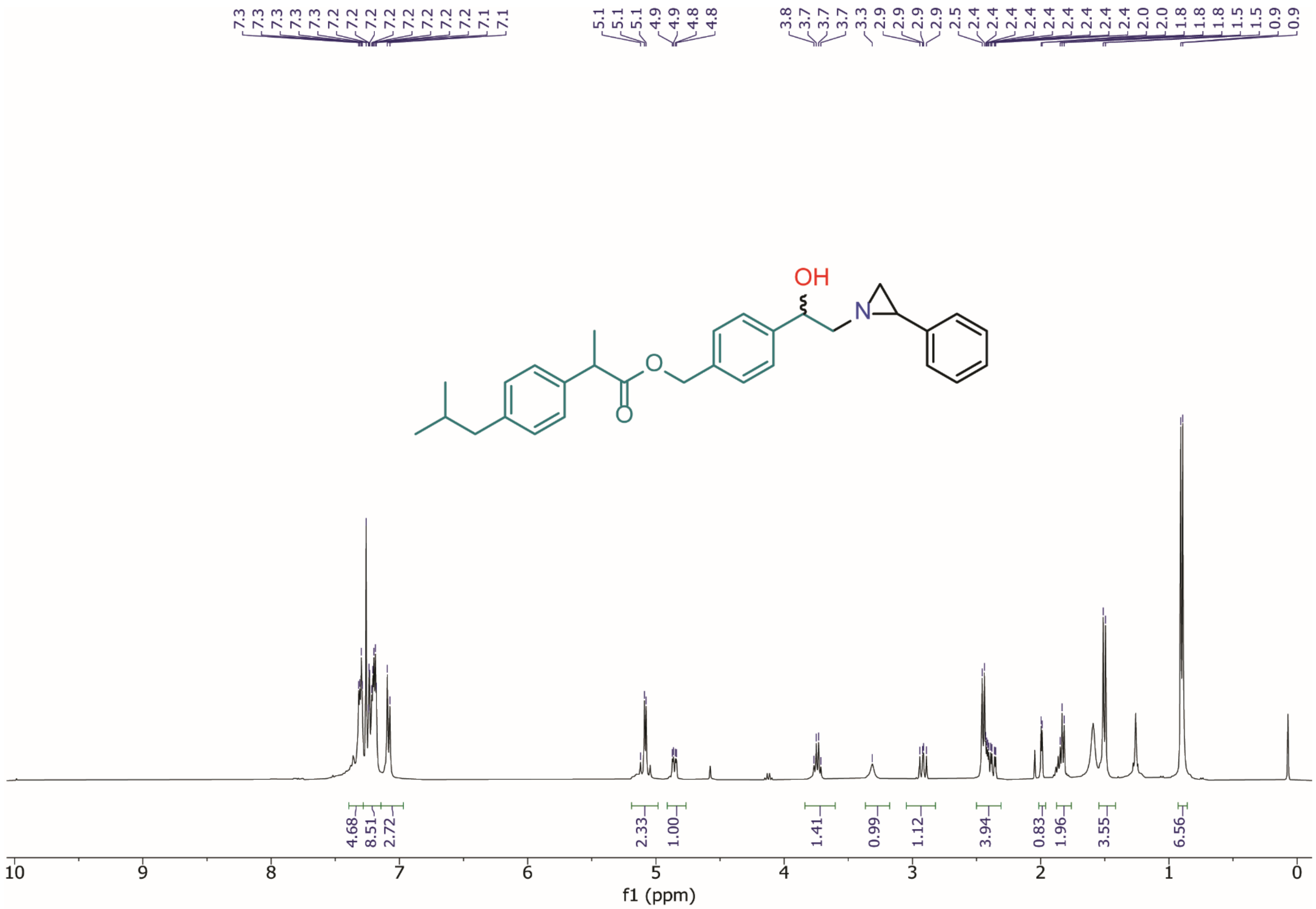

Figure S62. ^{1}H NMR spectrum of 4-(1-hydroxy-2-(2-phenylaziridin-1-yl)ethyl)benzyl 2-(4-isobutylphenyl)propanoate (**5o**) in $CDCl_3$ (400 MHz) at 23 °C.

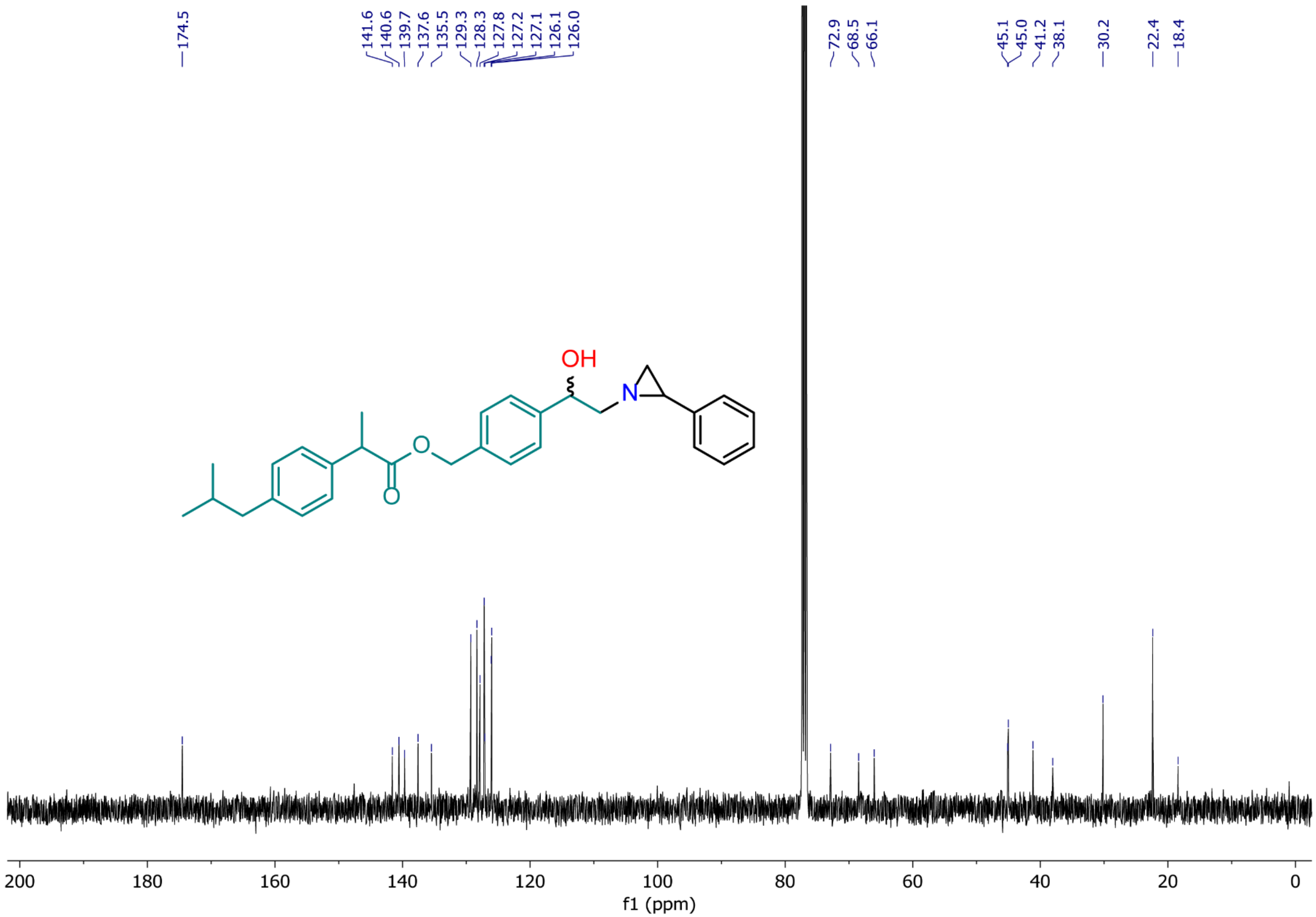

Figure S63. ^{13}C NMR spectrum of 4-(1-hydroxy-2-(2-phenylaziridin-1-yl)ethyl)benzyl 2-(4-isobutylphenyl)propanoate (**5o**) in $CDCl_3$ (101 MHz) at 23 °C.

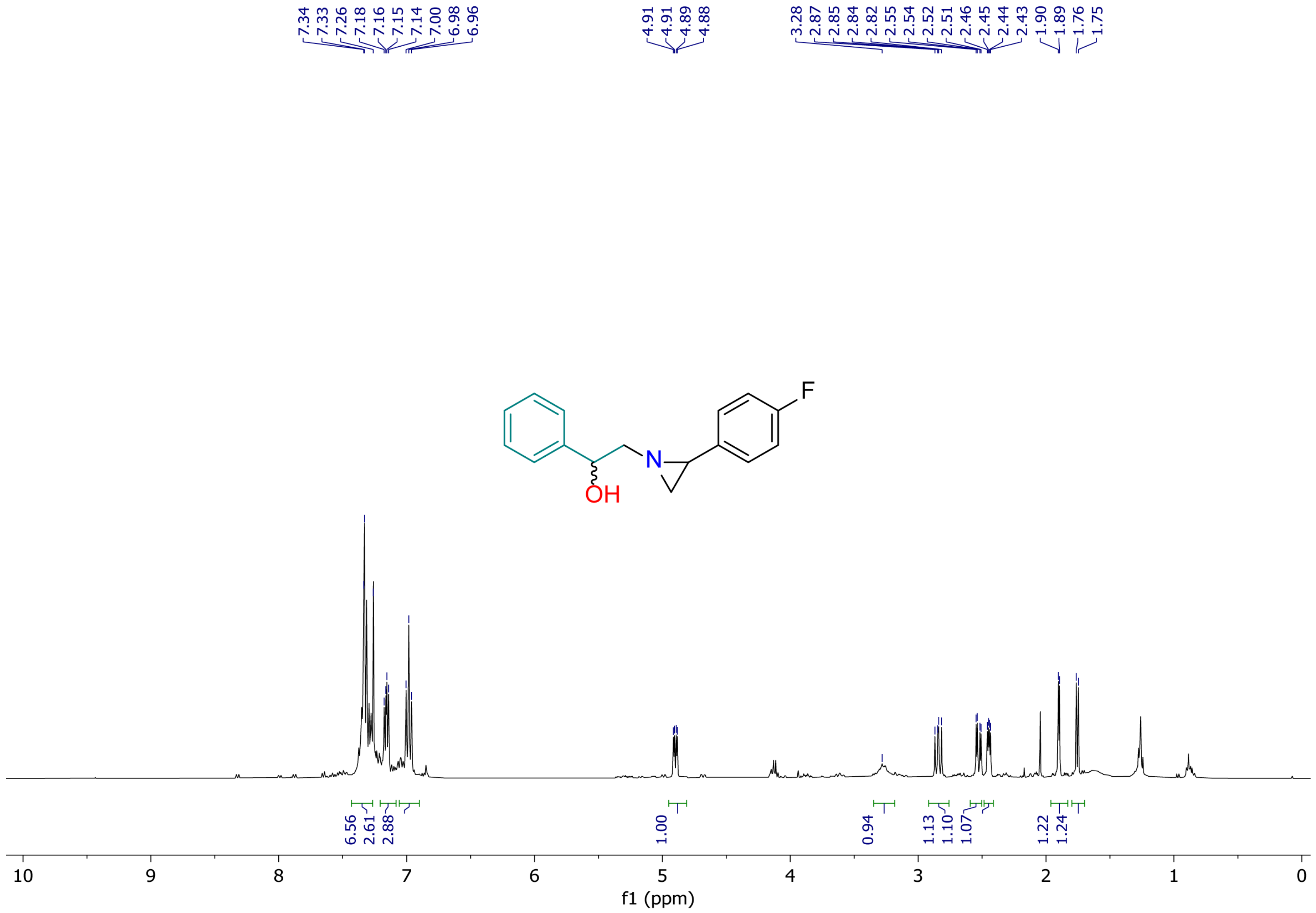

Figure S64. ^{1}H NMR spectrum of 2-(2-(4-fluorophenyl)aziridin-1-yl)-1-phenylethan-1-ol (**5p**) in $CDCl_3$ (400 MHz) at 23 °C

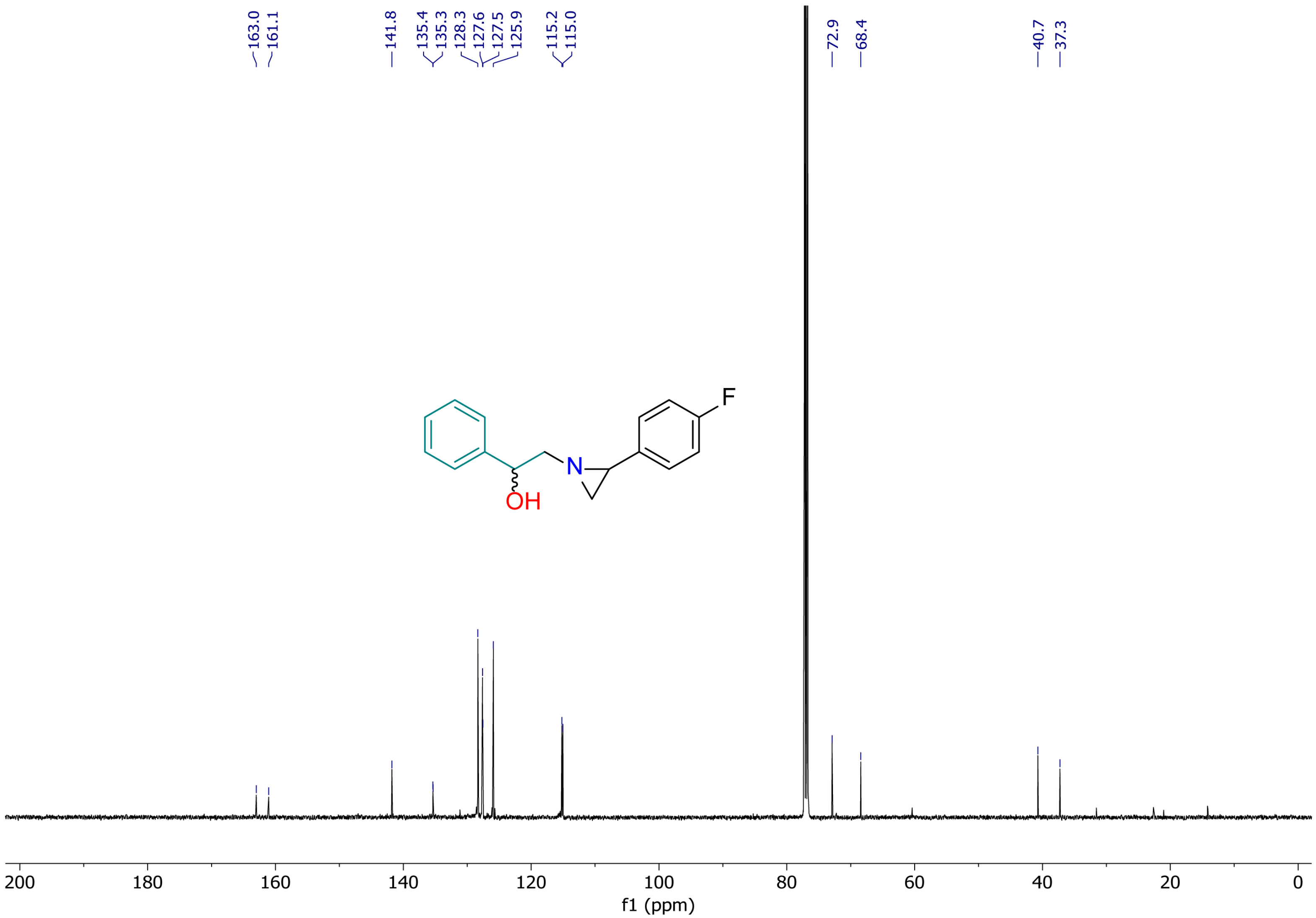

Figure S65. ^{13}C NMR spectrum of 2-(2-(4-fluorophenyl)aziridin-1-yl)-1-phenylethan-1-ol (**5p**) in $CDCl_3$ (101 MHz) at 23 °C

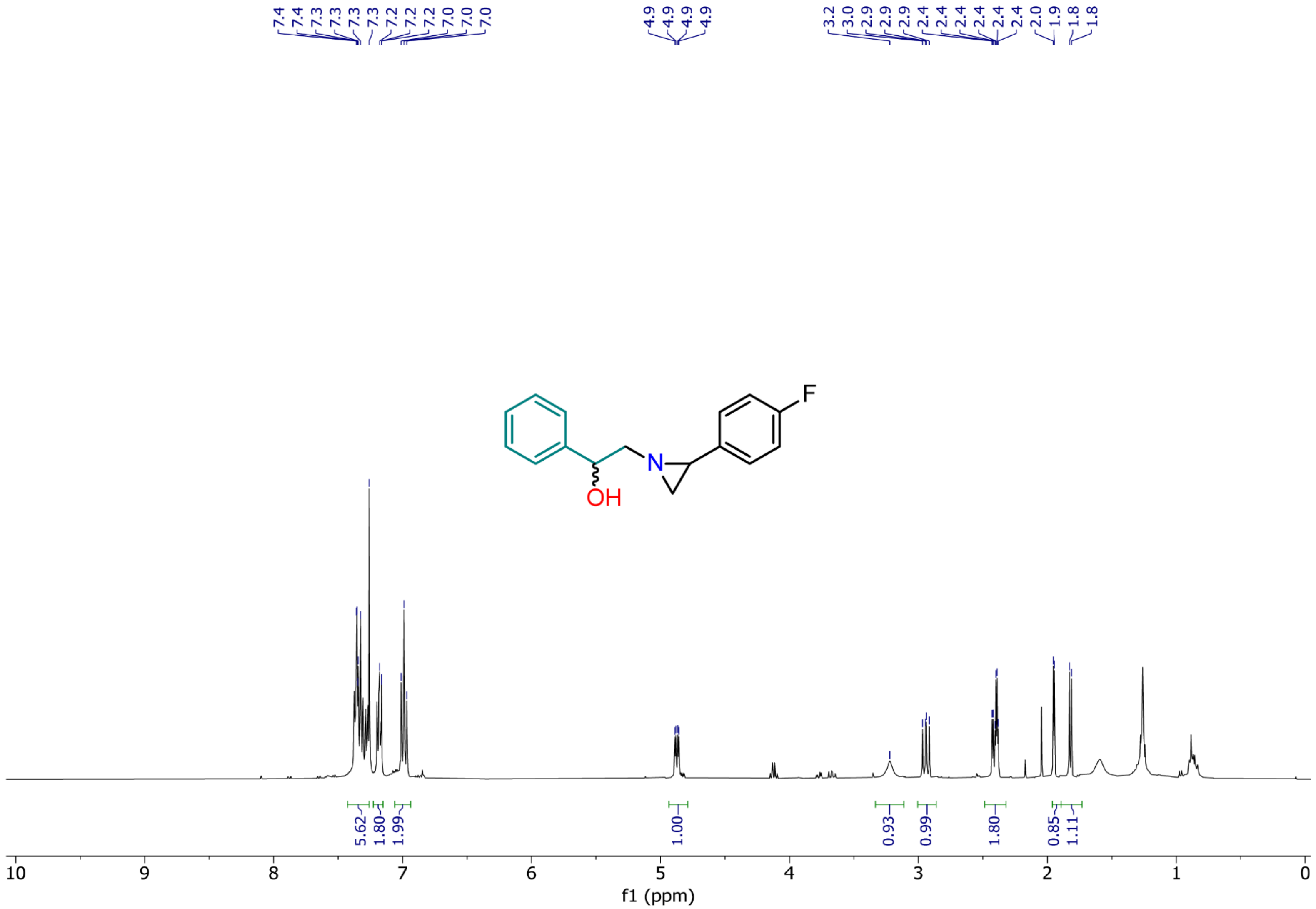

Figure S66. ^{1}H NMR spectrum of 2-(2-(4-fluorophenyl)aziridin-1-yl)-1-phenylethan-1-ol (**5p**) in $CDCl_3$ (400 MHz) at 23 °C.

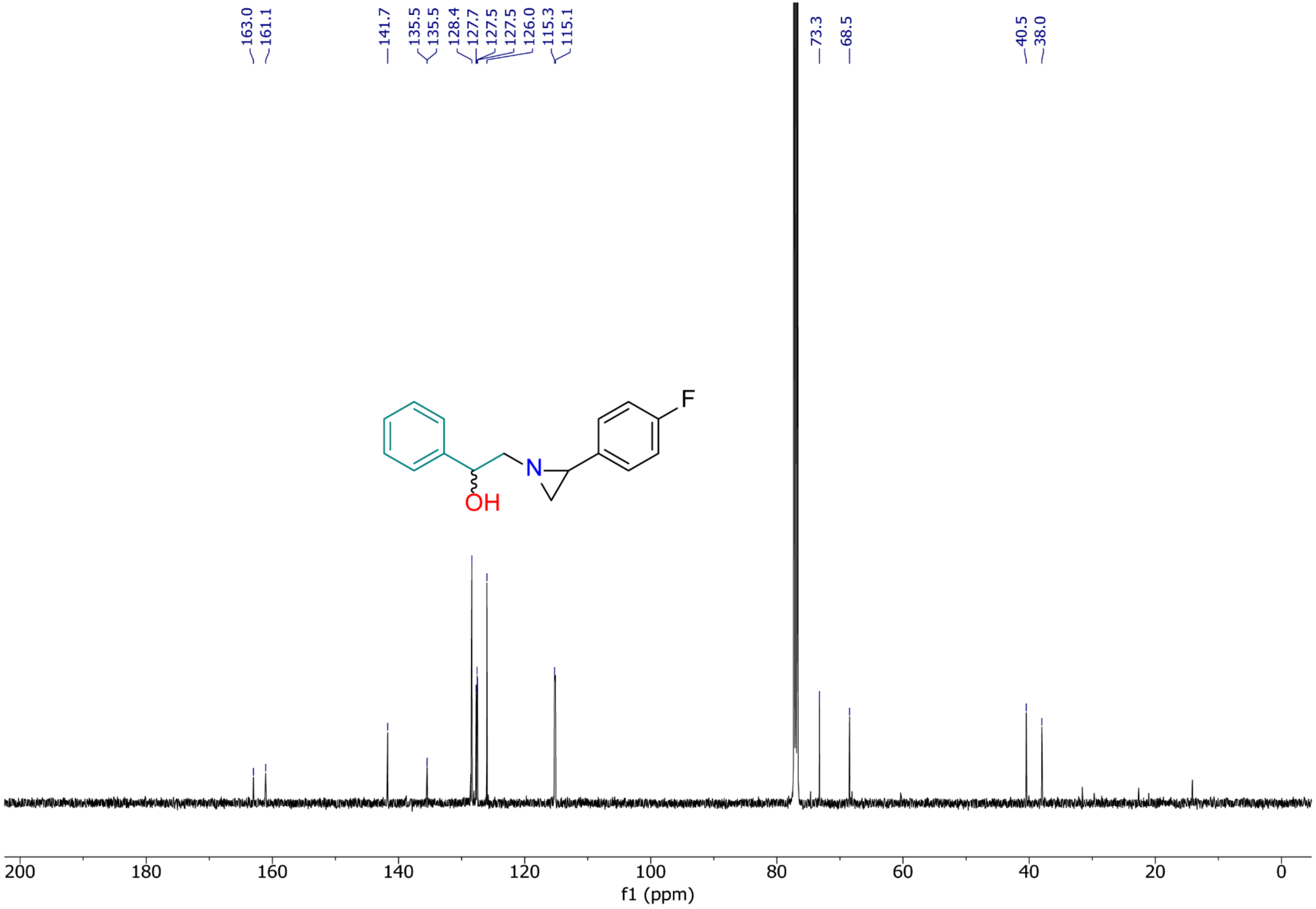

Figure S67. ^{13}C NMR spectrum of 2-(2-(4-fluorophenyl)aziridin-1-yl)-1-phenylethan-1-ol (**5p**) in $CDCl_3$ (101 MHz) at 23 °C.

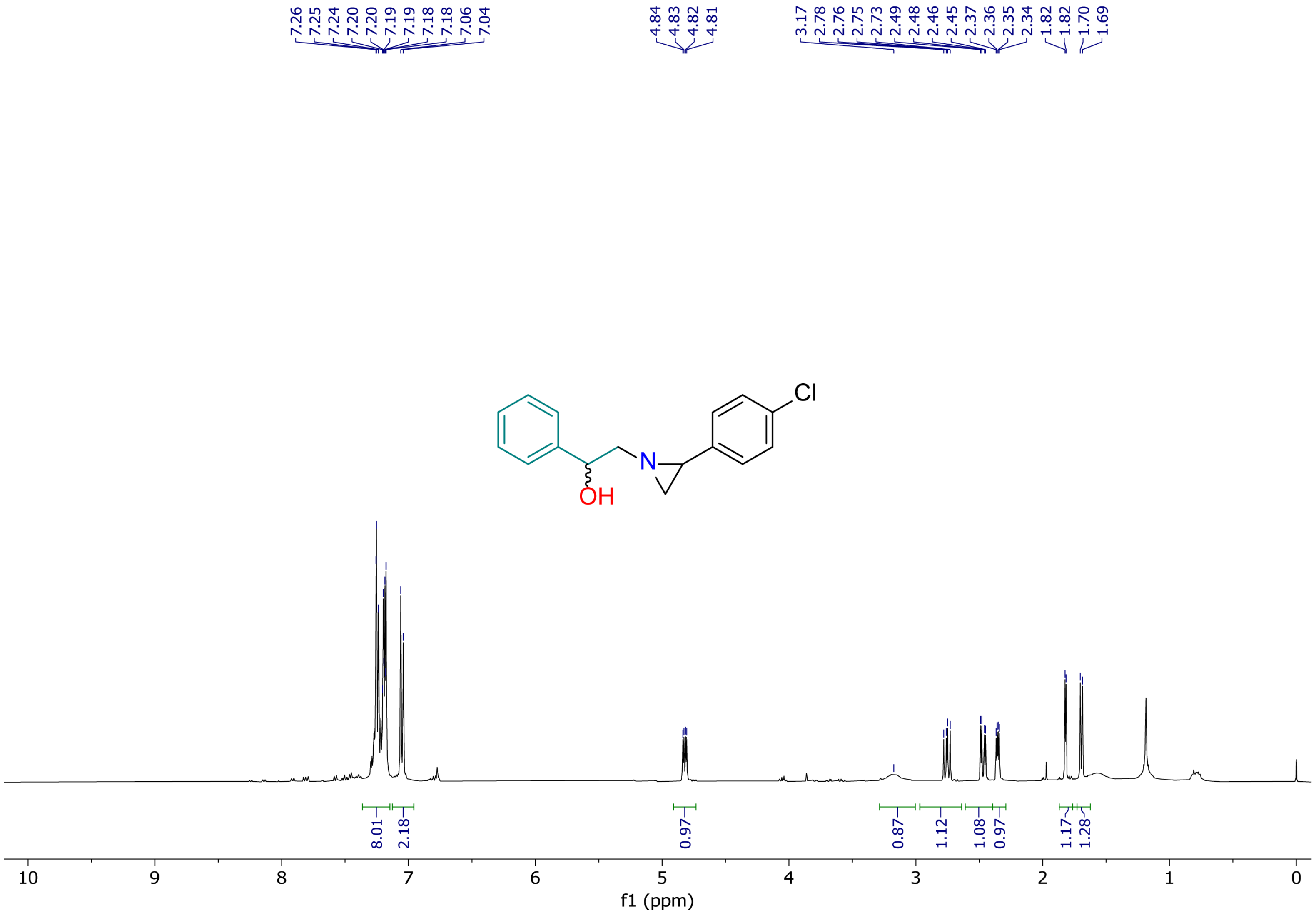

Figure S68. ^{1}H NMR spectrum of 2-(2-(4-chlorophenyl)aziridin-1-yl)-1-phenylethan-1-ol (**5q**) in $CDCl_3$ (400 MHz) at 23 °C.

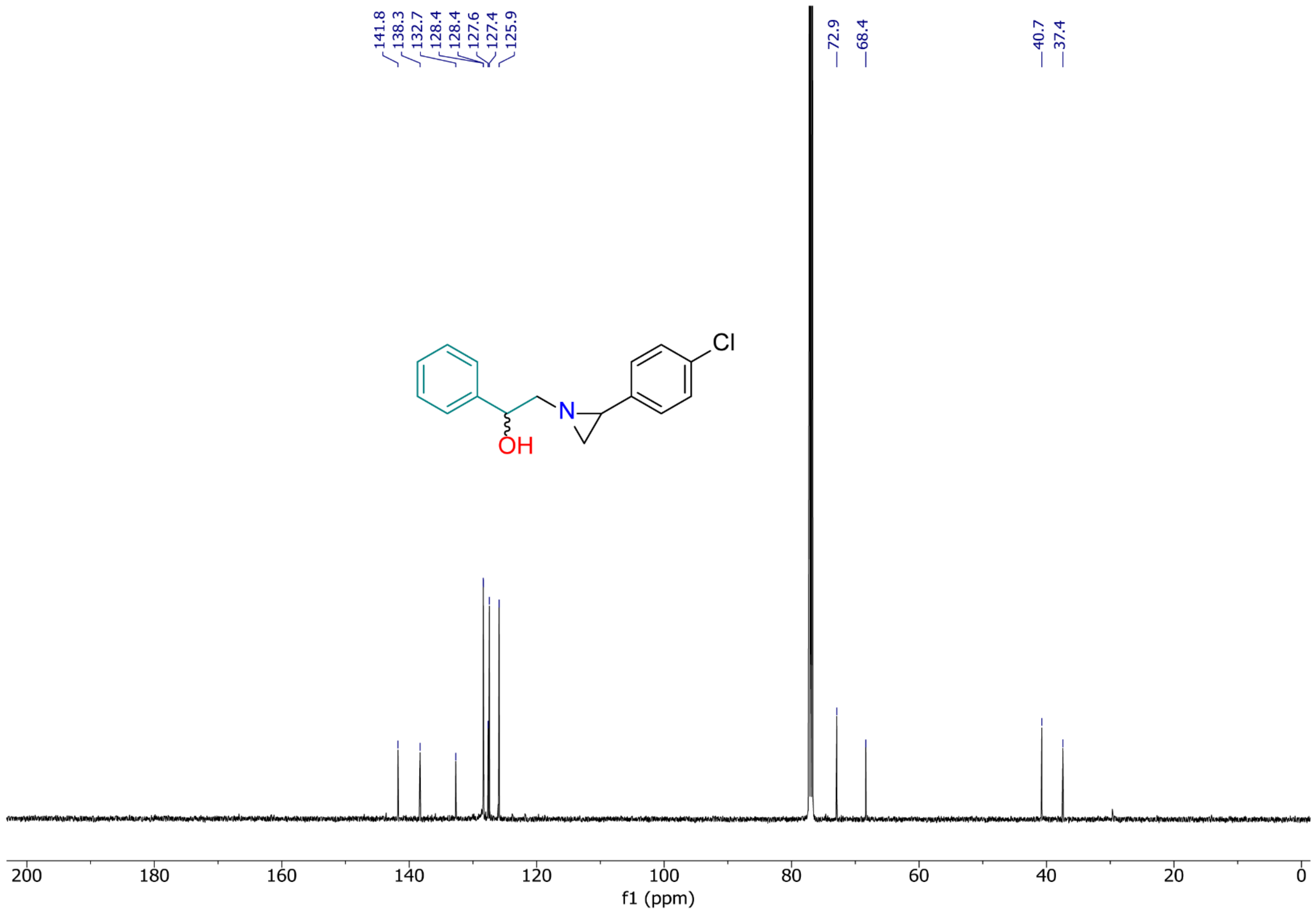

Figure S69. ^{13}C NMR spectrum of 2-(2-(4-chlorophenyl)aziridin-1-yl)-1-phenylethan-1-ol (**5q**) in $CDCl_3$ (101 MHz) at 23 °C.

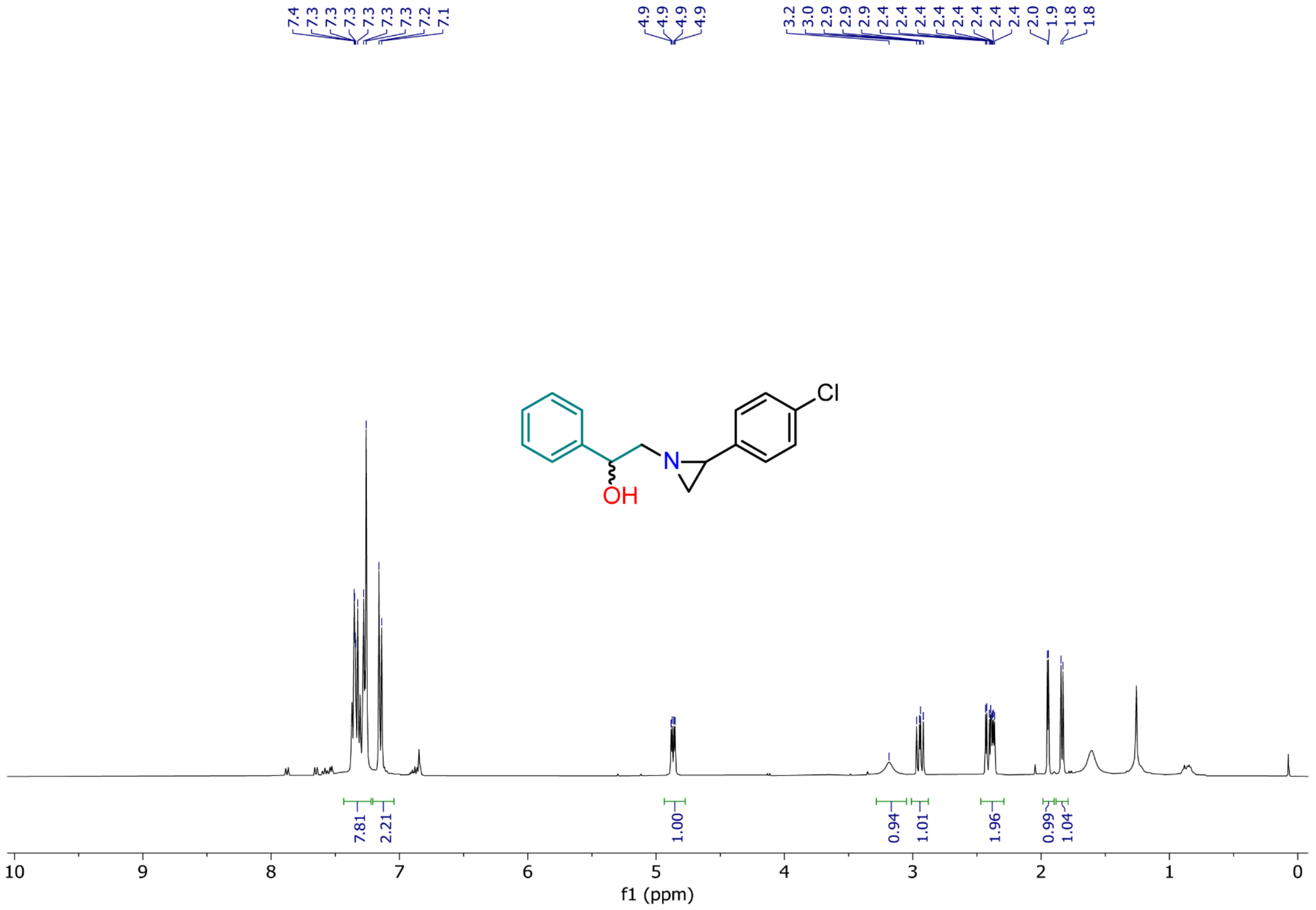

Figure S70. ^{1}H NMR spectrum of 2-(2-(4-chlorophenyl)aziridin-1-yl)-1-phenylethan-1-ol (**5q**) in $CDCl_3$ (400 MHz) at 23 °C.

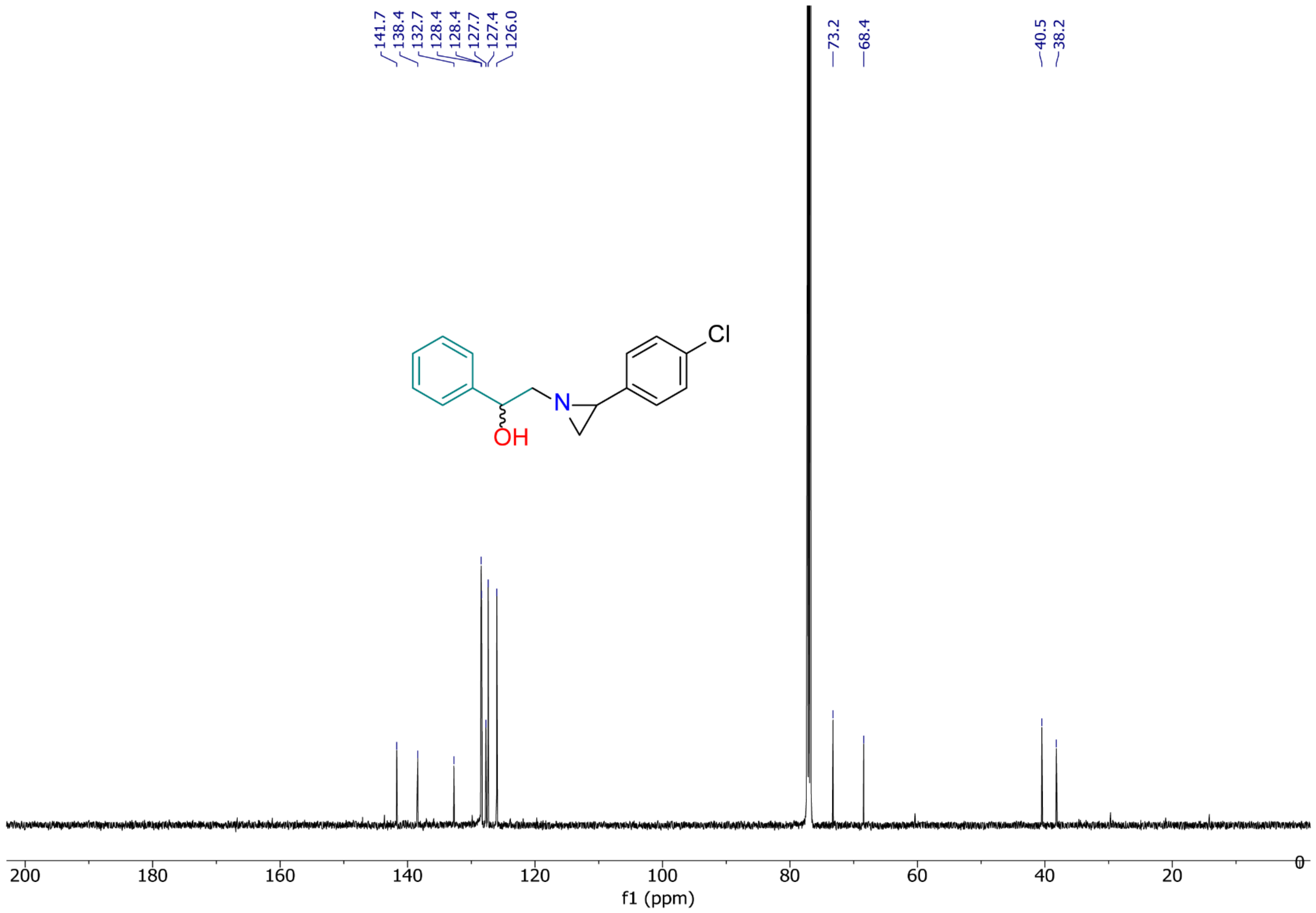

Figure S71. ^{13}C NMR spectrum of 2-(2-(4-chlorophenyl)aziridin-1-yl)-1-phenylethan-1-ol (**5q**) in $CDCl_3$ (101 MHz) at 23 °C.

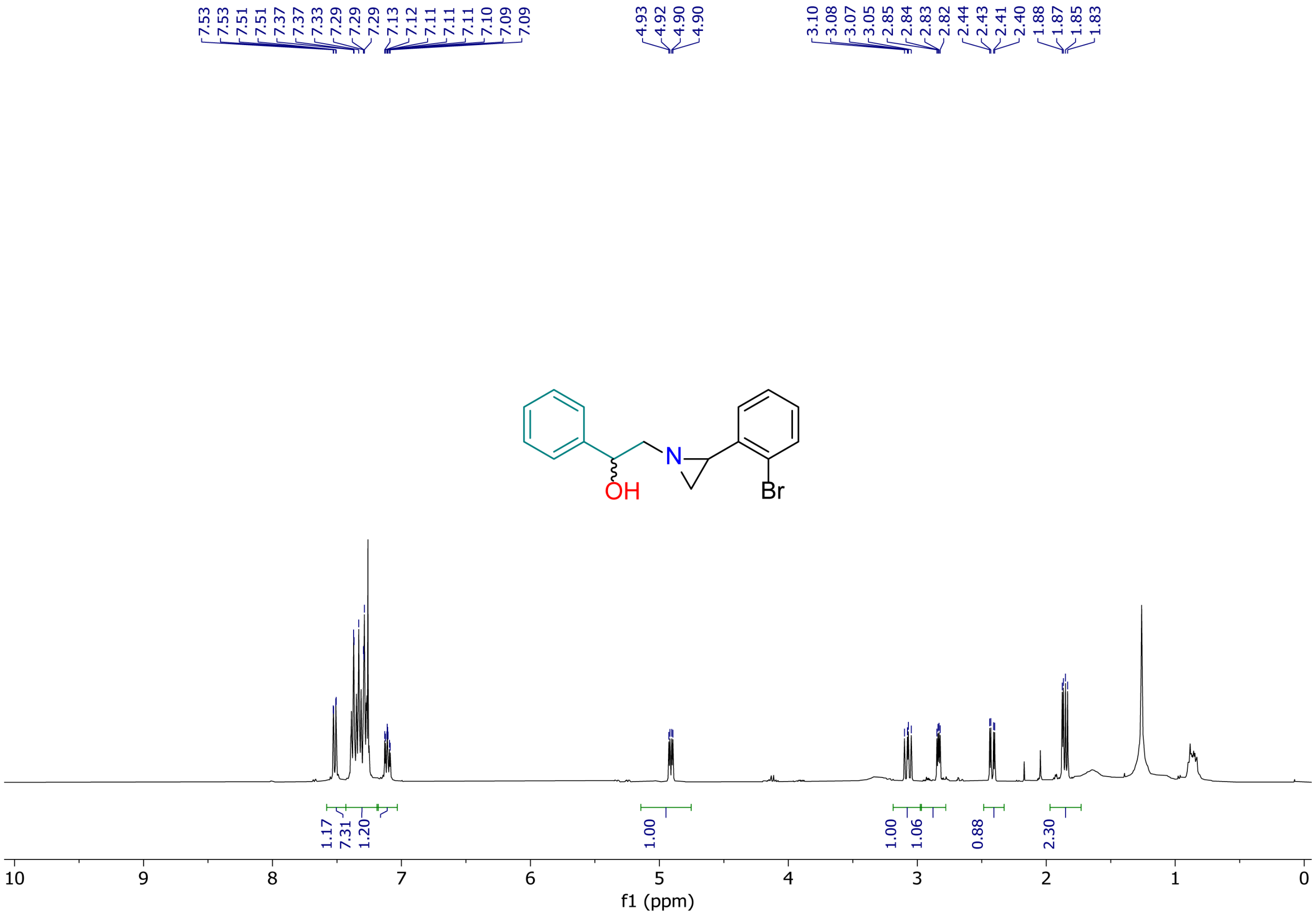

Figure S72. ^{1}H NMR spectrum of 2-(2-(2-bromophenyl)aziridin-1-yl)-1-phenylethan-1-ol (**5r**) in $CDCl_3$ (400 MHz) at 23 °C.

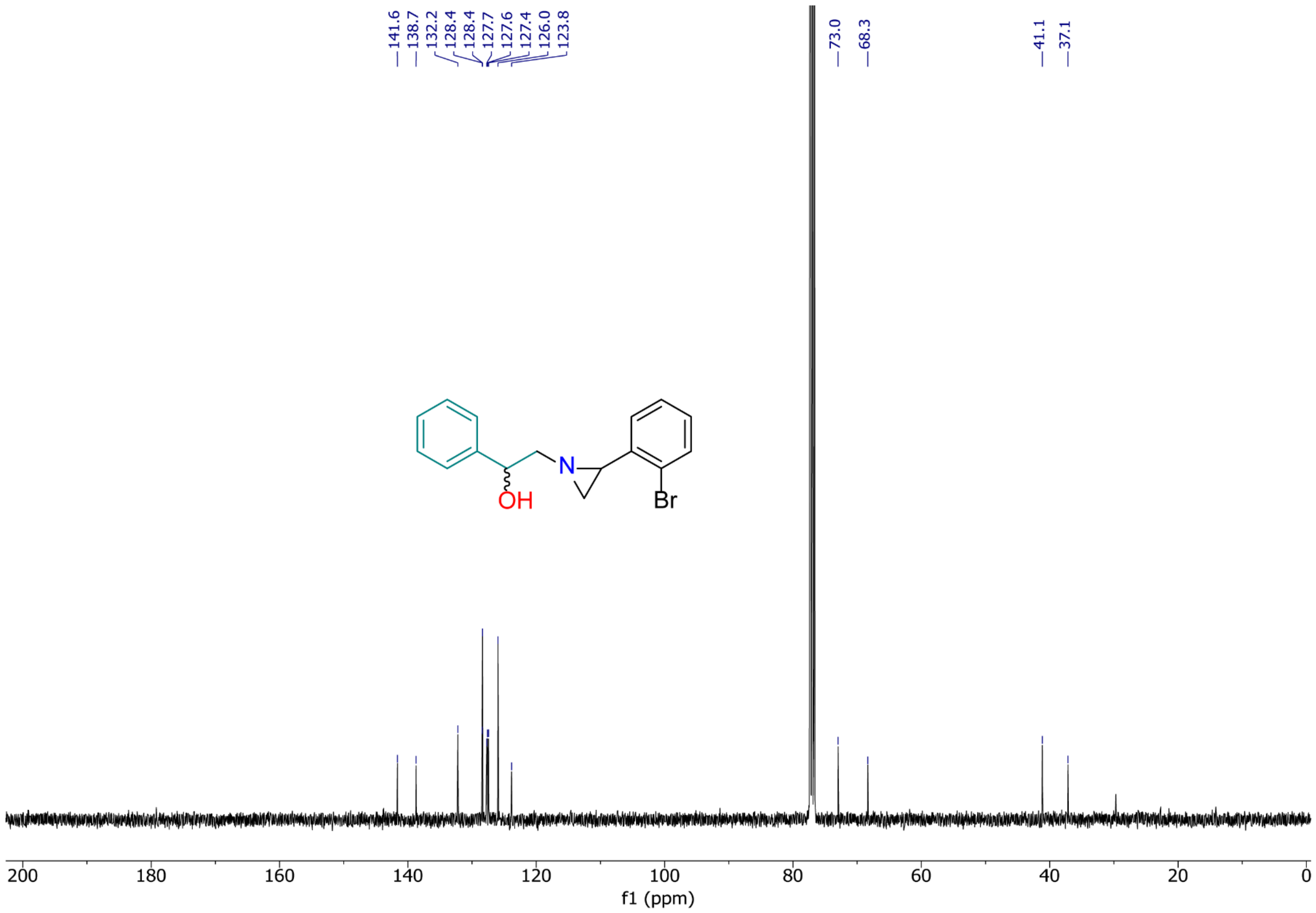

Figure S73. ^{13}C NMR spectrum of 2-(2-(2-Bromophenyl)aziridin-1-yl)-1-phenylethan-1-ol (**5r**) in $CDCl_3$ (101 MHz) at 23 °C.

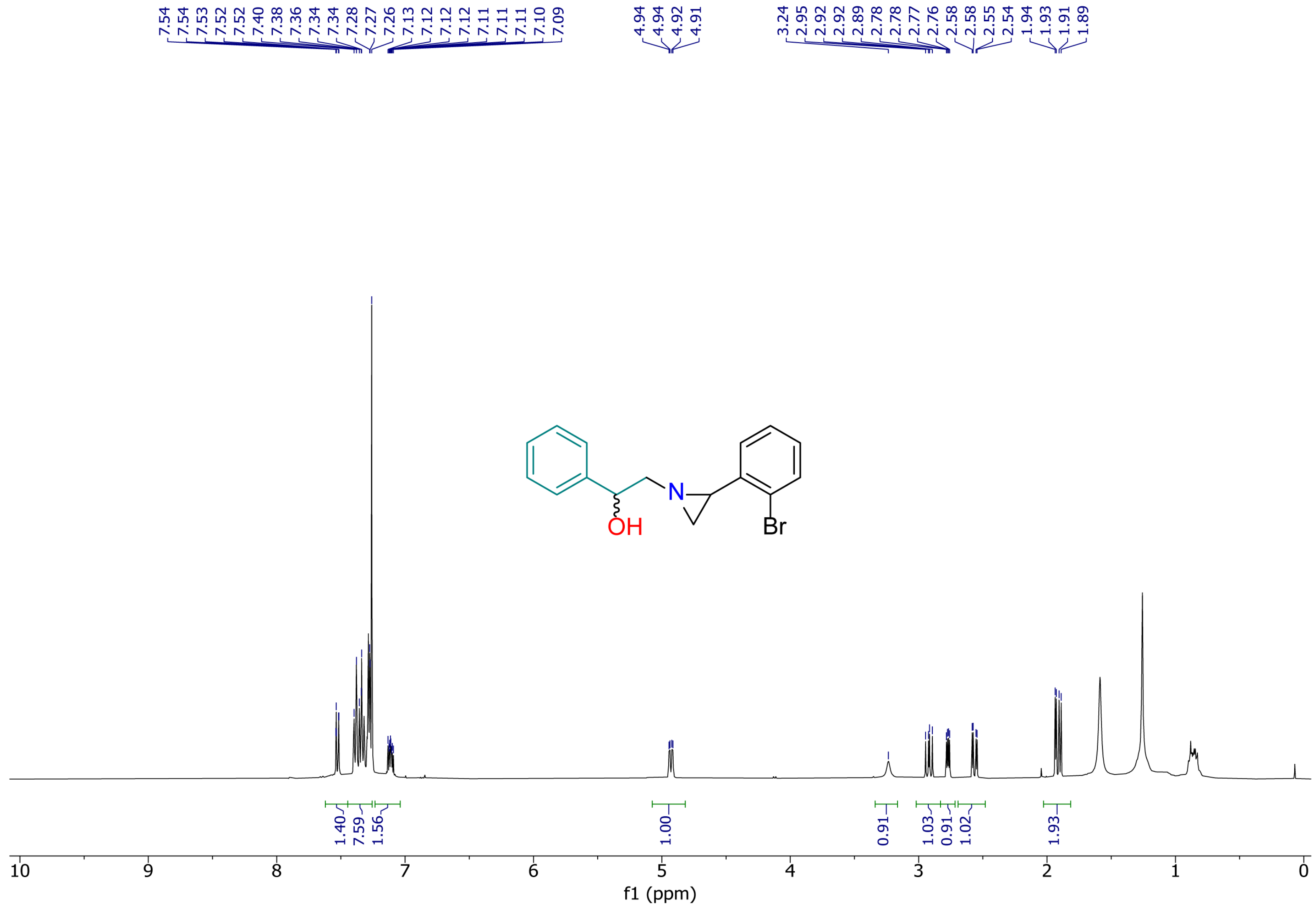

Figure S74. ^{1}H NMR spectrum of 2-(2-(2-bromophenyl)aziridin-1-yl)-1-phenylethan-1-ol (**5r**) in $CDCl_3$ (400 MHz) at 23 °C.

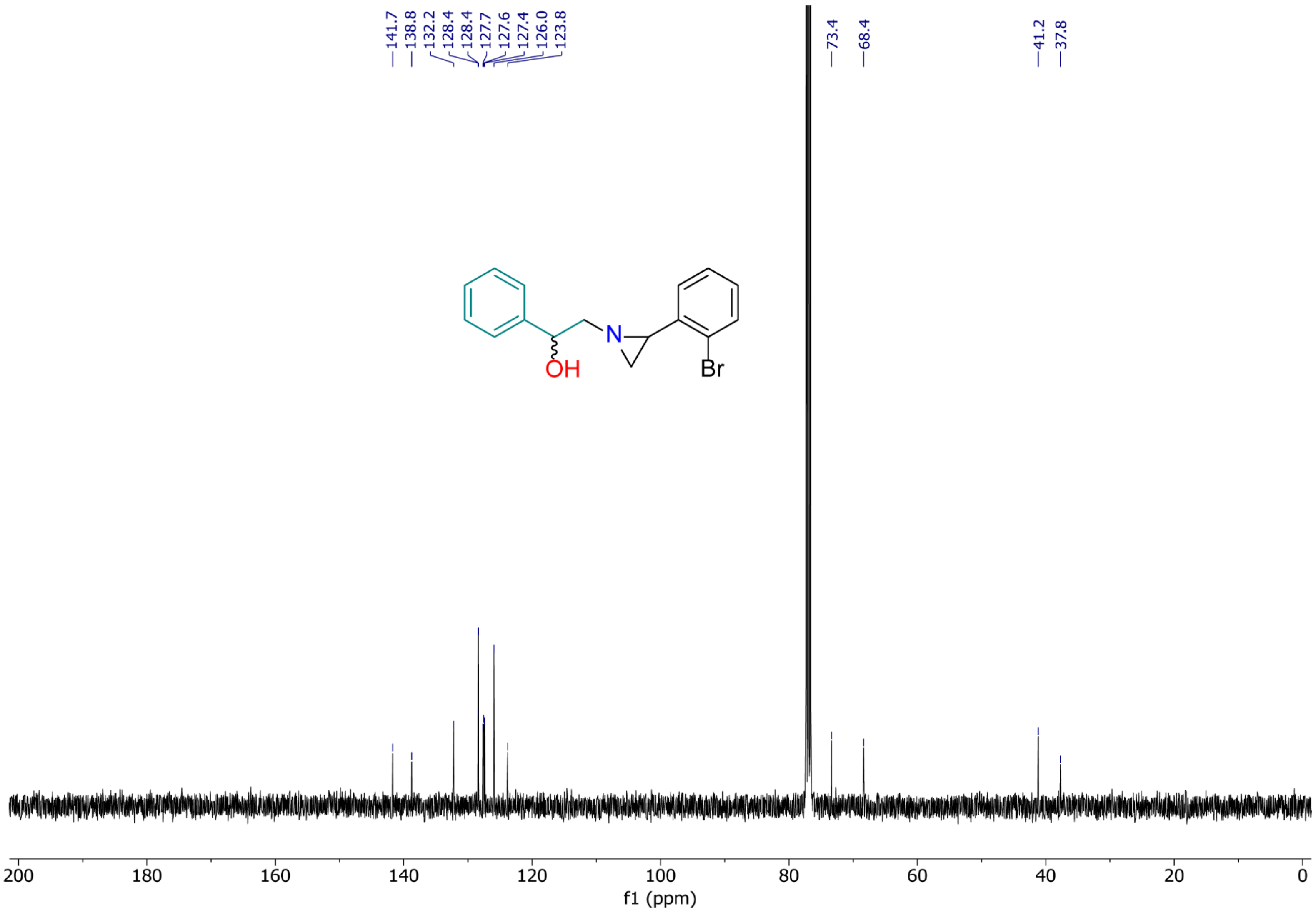

Figure S75. ^{13}C NMR spectrum of 2-(2-(2-bromophenyl)aziridin-1-yl)-1-phenylethan-1-ol (**5r**) in $CDCl_3$ (101 MHz) at 23 °C.

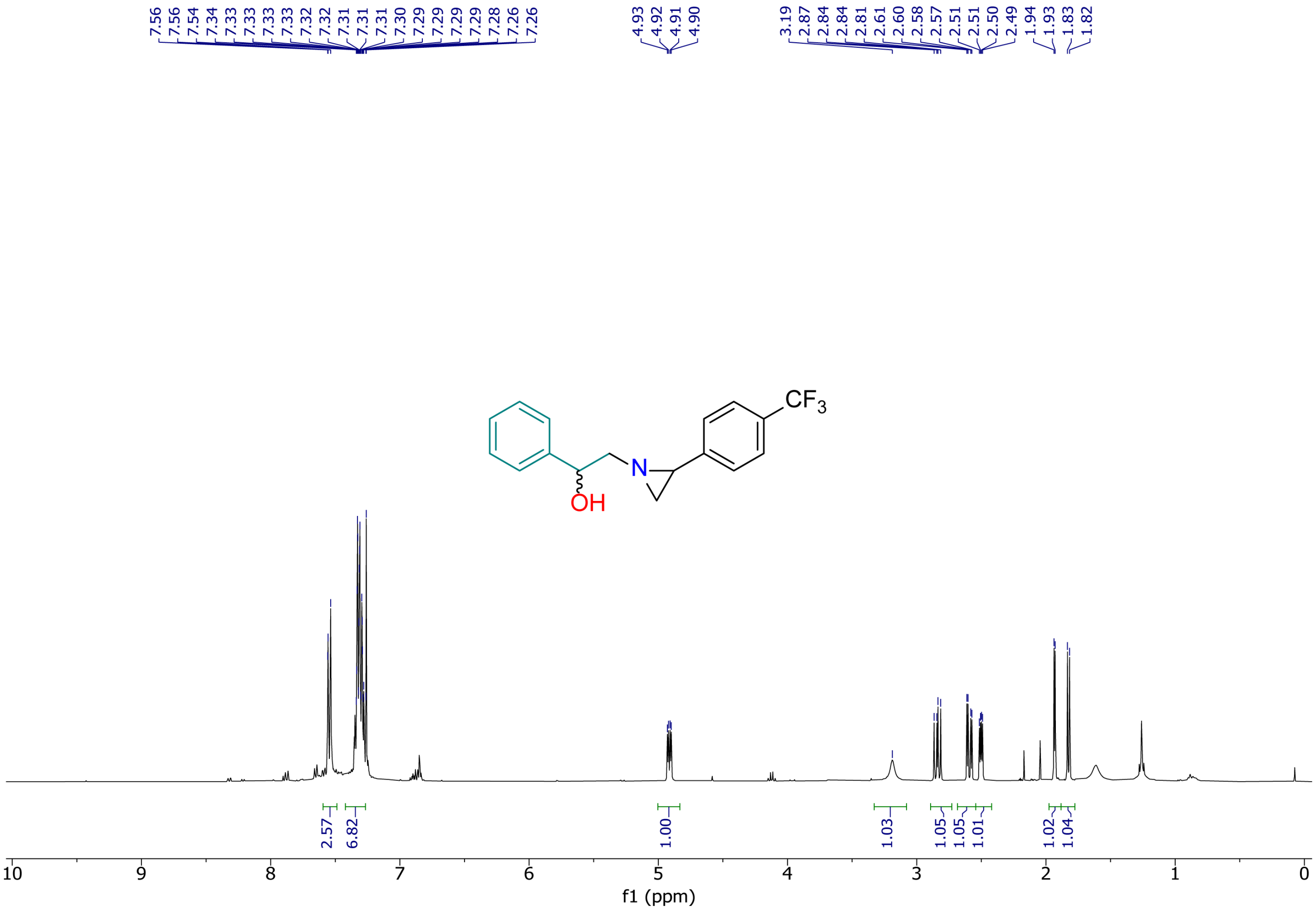

Figure S76. ^{1}H NMR spectrum of 1-phenyl-2-(2-(4-(trifluoromethyl)phenyl)aziridin-1-yl)ethan-1-ol(**5s**) in $CDCl_3$ (400 MHz) at 23 °C.

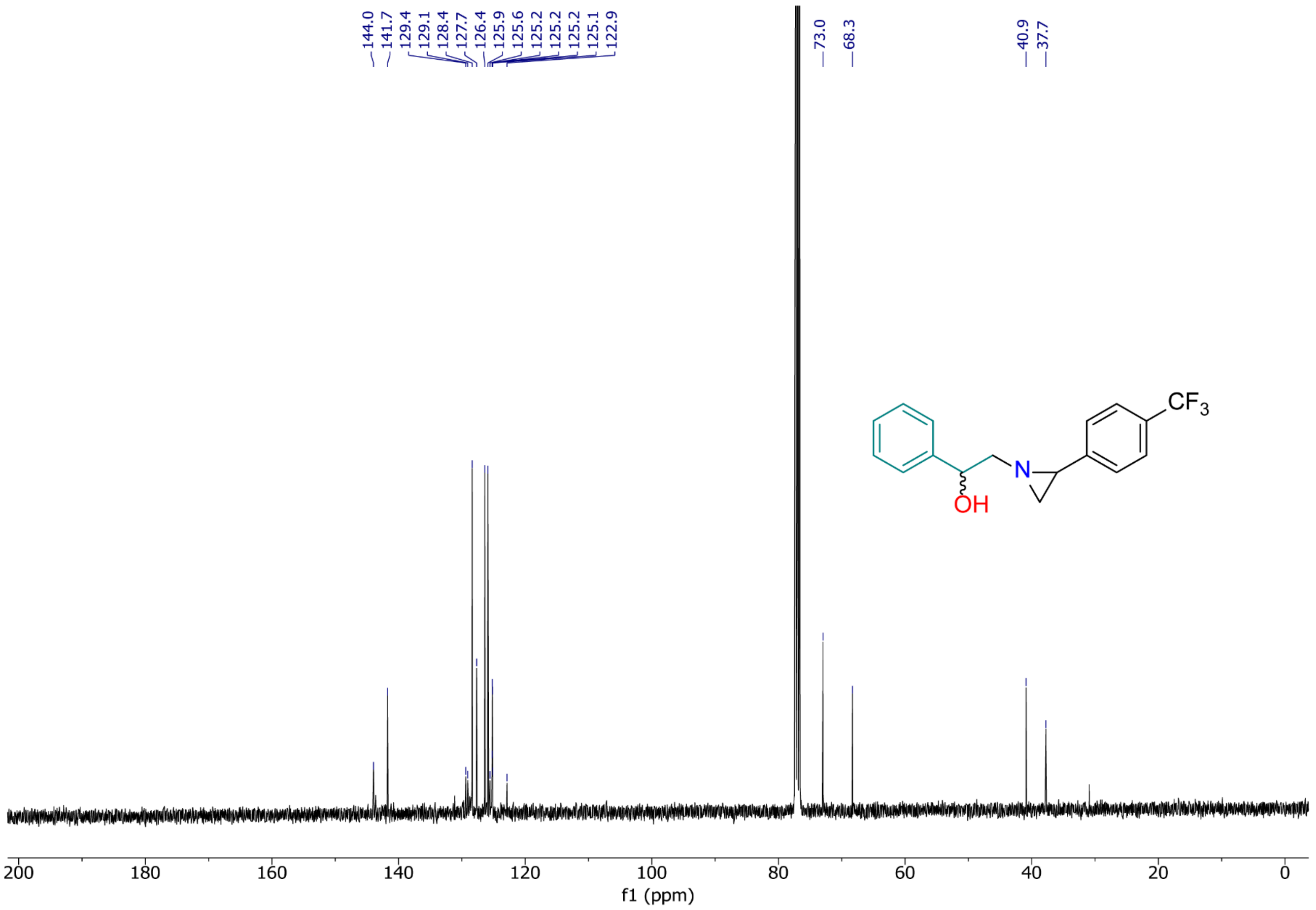

Figure S77. ^{13}C NMR spectrum of 1-phenyl-2-(2-(4-(trifluoromethyl)phenyl)aziridin-1-yl)ethan-1-ol(**5s**) in $CDCl_3$ (101 MHz) at 23 °C.

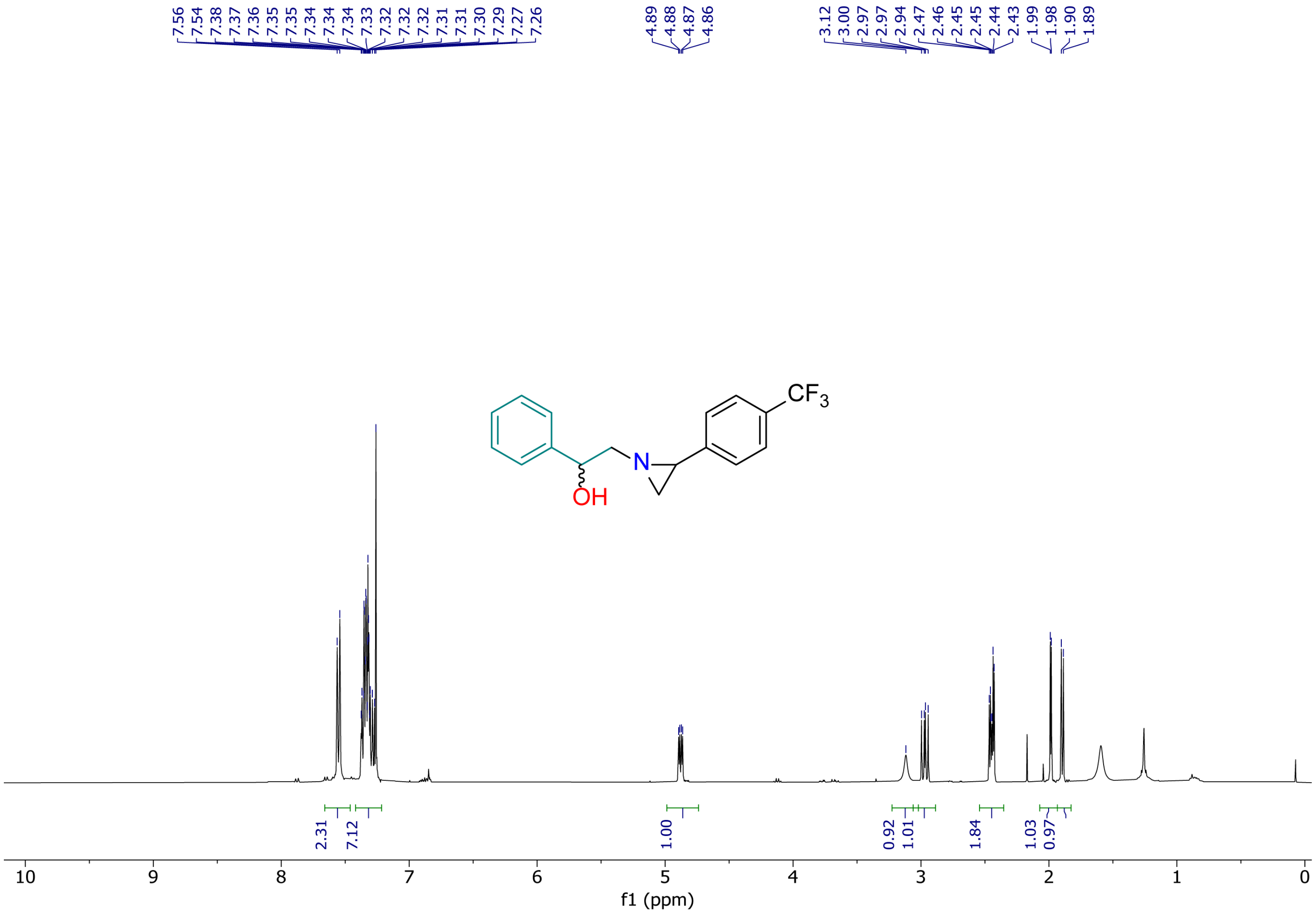

Figure S78. ^{1}H NMR spectrum of 1-phenyl-2-(2-(4-(trifluoromethyl)phenyl)aziridin-1-yl)ethan-1-ol(**5s**) in $CDCl_3$ (400 MHz) at 23 °C.

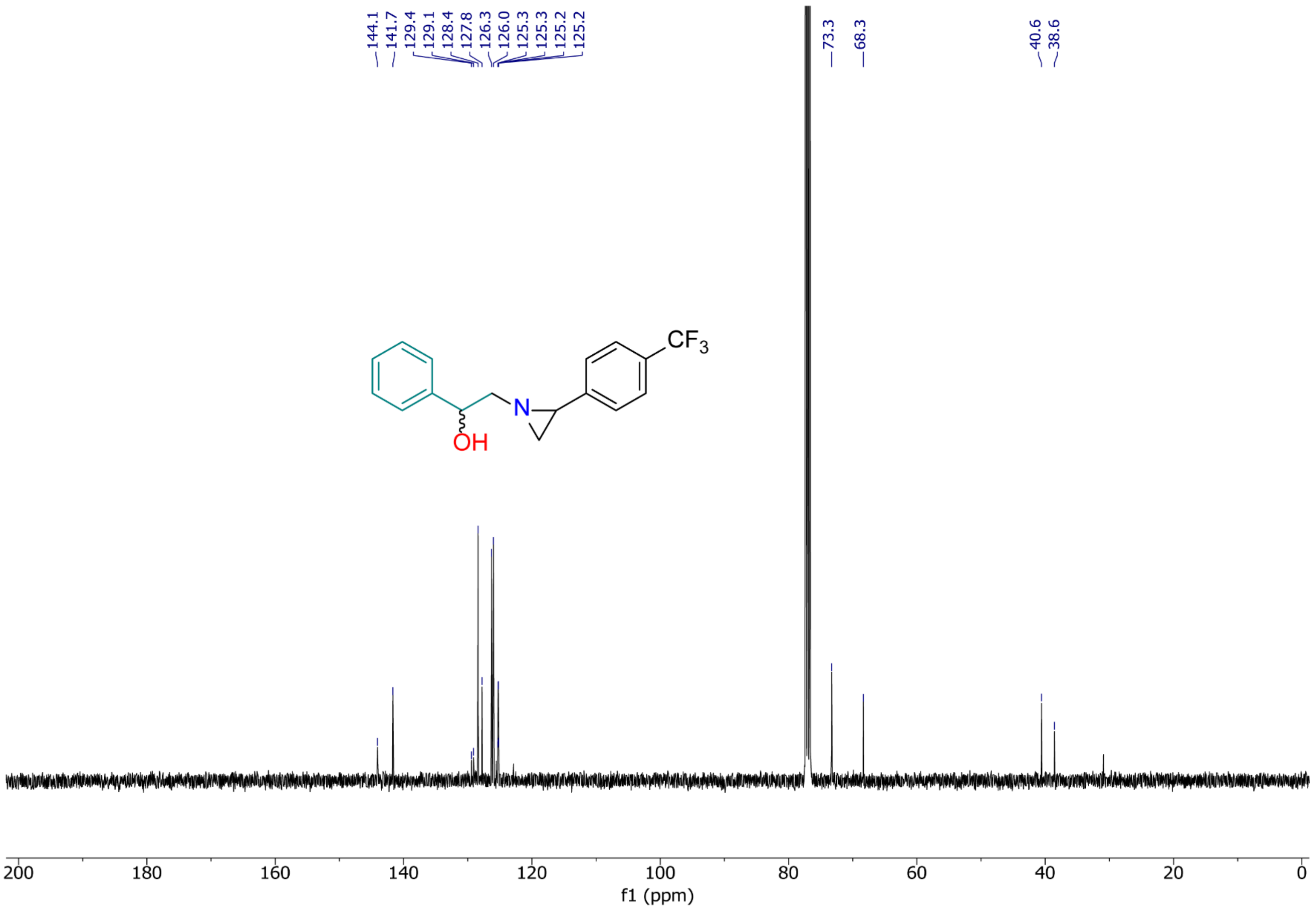

Figure S79. ^{13}C NMR spectrum of 1-phenyl-2-(2-(4-(trifluoromethyl)phenyl)aziridin-1-yl)ethan-1-ol(**5s**) in $CDCl_3$ (101 MHz) at 23 °C.

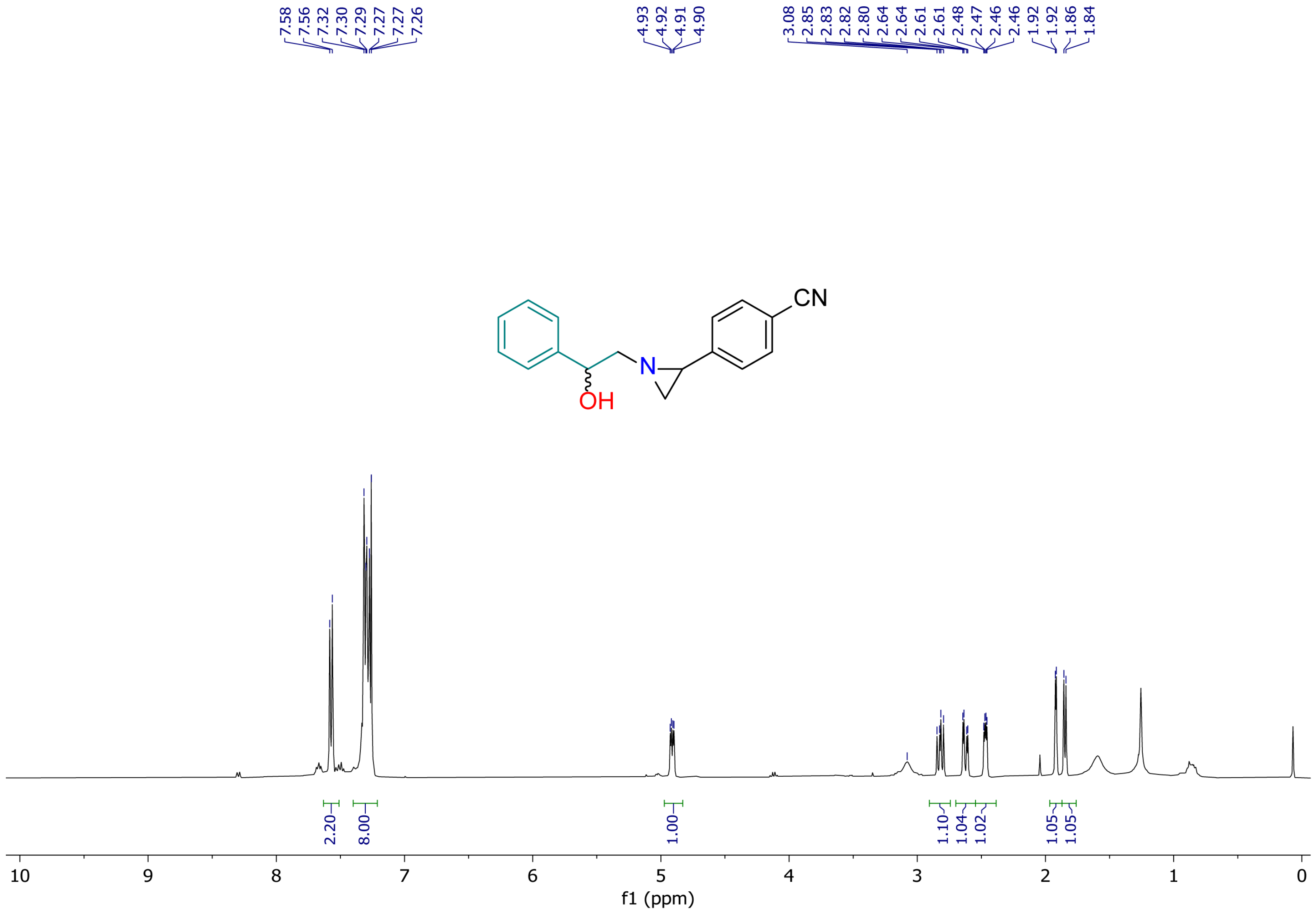

Figure S80. ^{1}H NMR spectrum of 4-(1-(2-hydroxy-2-phenylethyl)aziridin-2-yl)benzonitrile(**5t**) in $CDCl_3$ (400 MHz) at 23 °C.

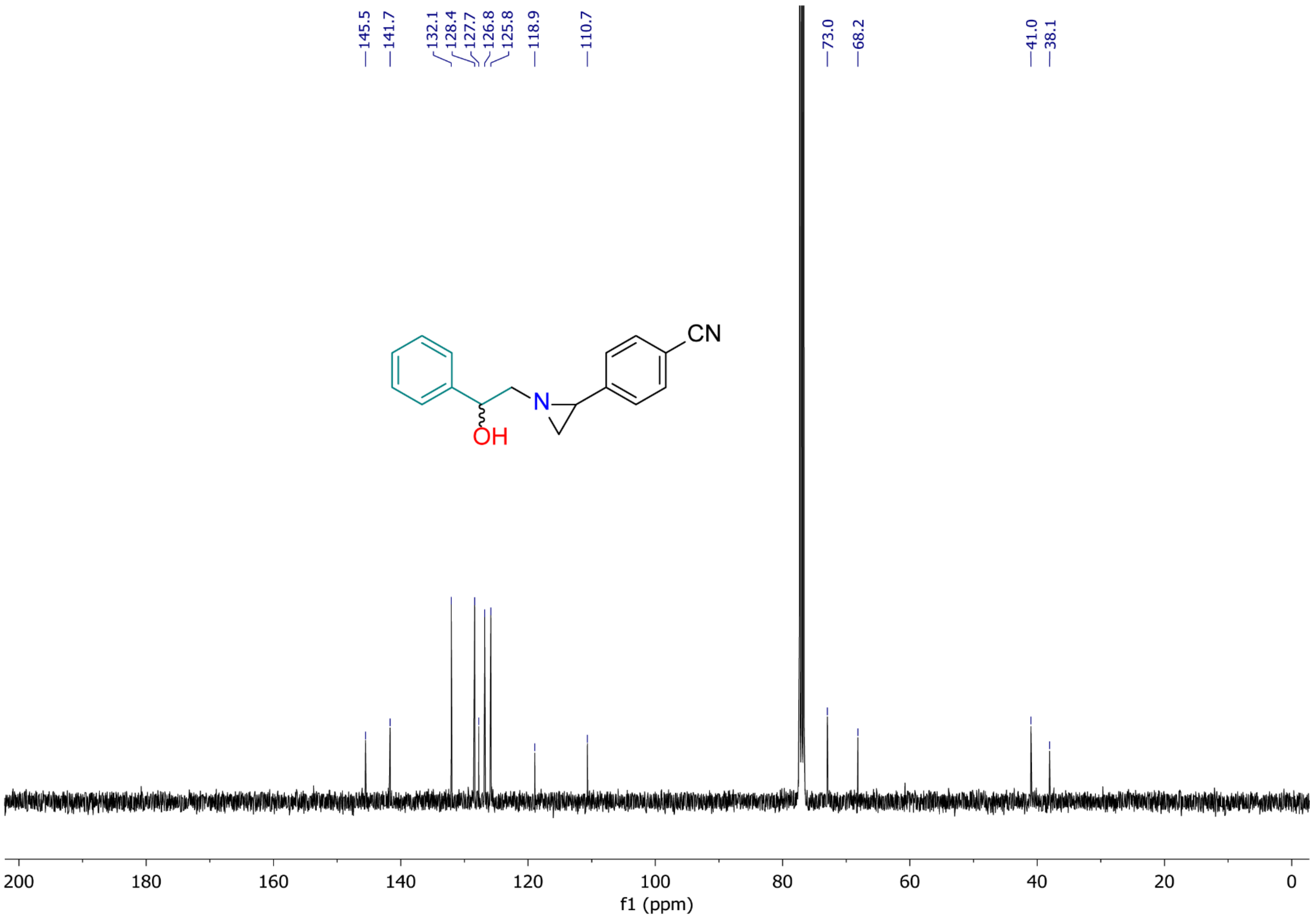

Figure S81. ^{13}C NMR spectrum of 4-(1-(2-hydroxy-2-phenylethyl)aziridin-2-yl)benzonitrile(**5t**) in $CDCl_3$ (101 MHz) at 23 °C.

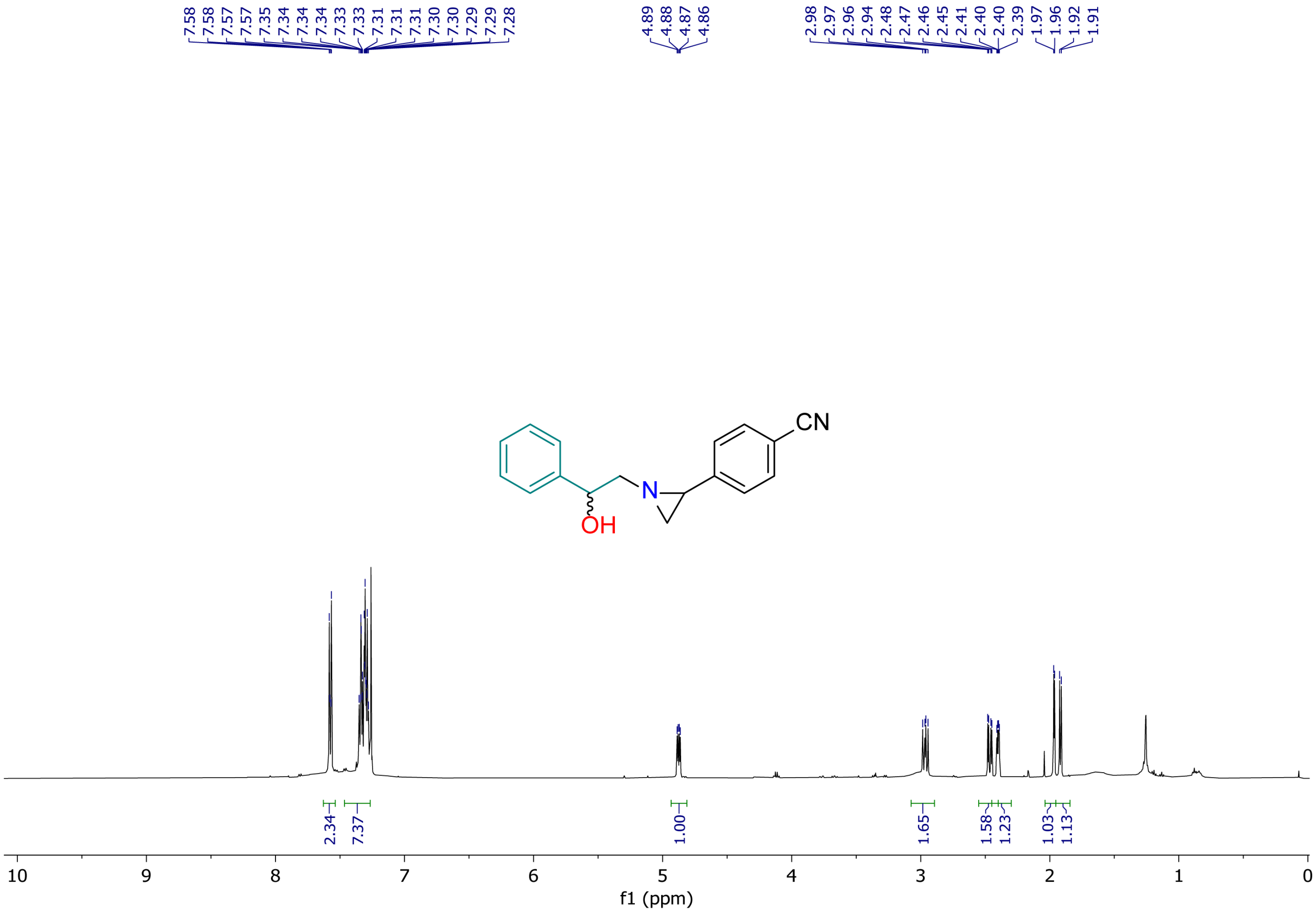

Figure S82. ^{1}H NMR spectrum of 4-(1-(2-hydroxy-2-phenylethyl)aziridin-2-yl)benzonitrile(**5t**) in $CDCl_3$ (400 MHz) at 23 °C.

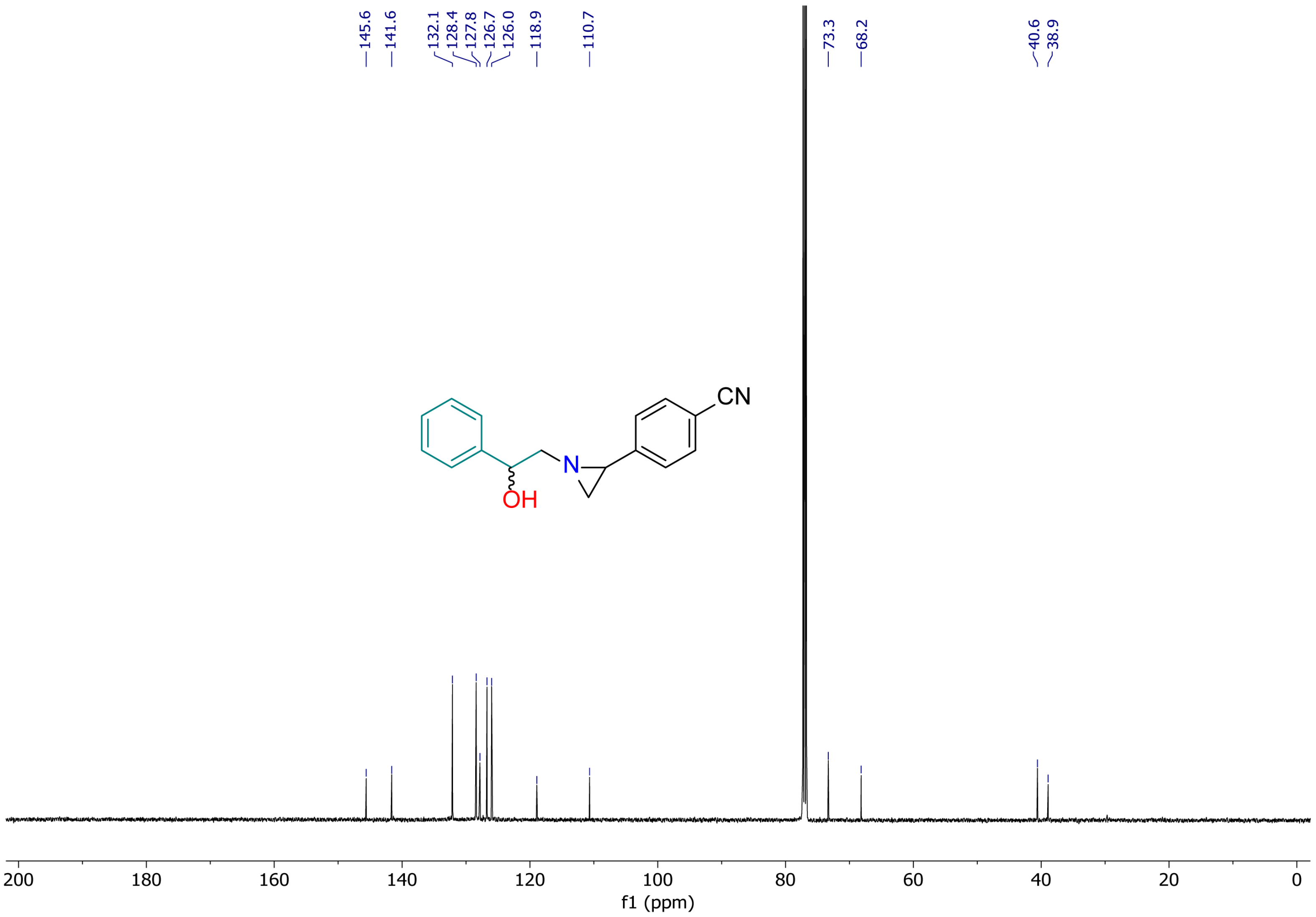

Figure S83. ^{13}C NMR spectrum of 4-(1-(2-hydroxy-2-phenylethyl)aziridin-2-yl)benzonitrile(**5t**) in $CDCl_3$ (101 MHz) at 23 °C.

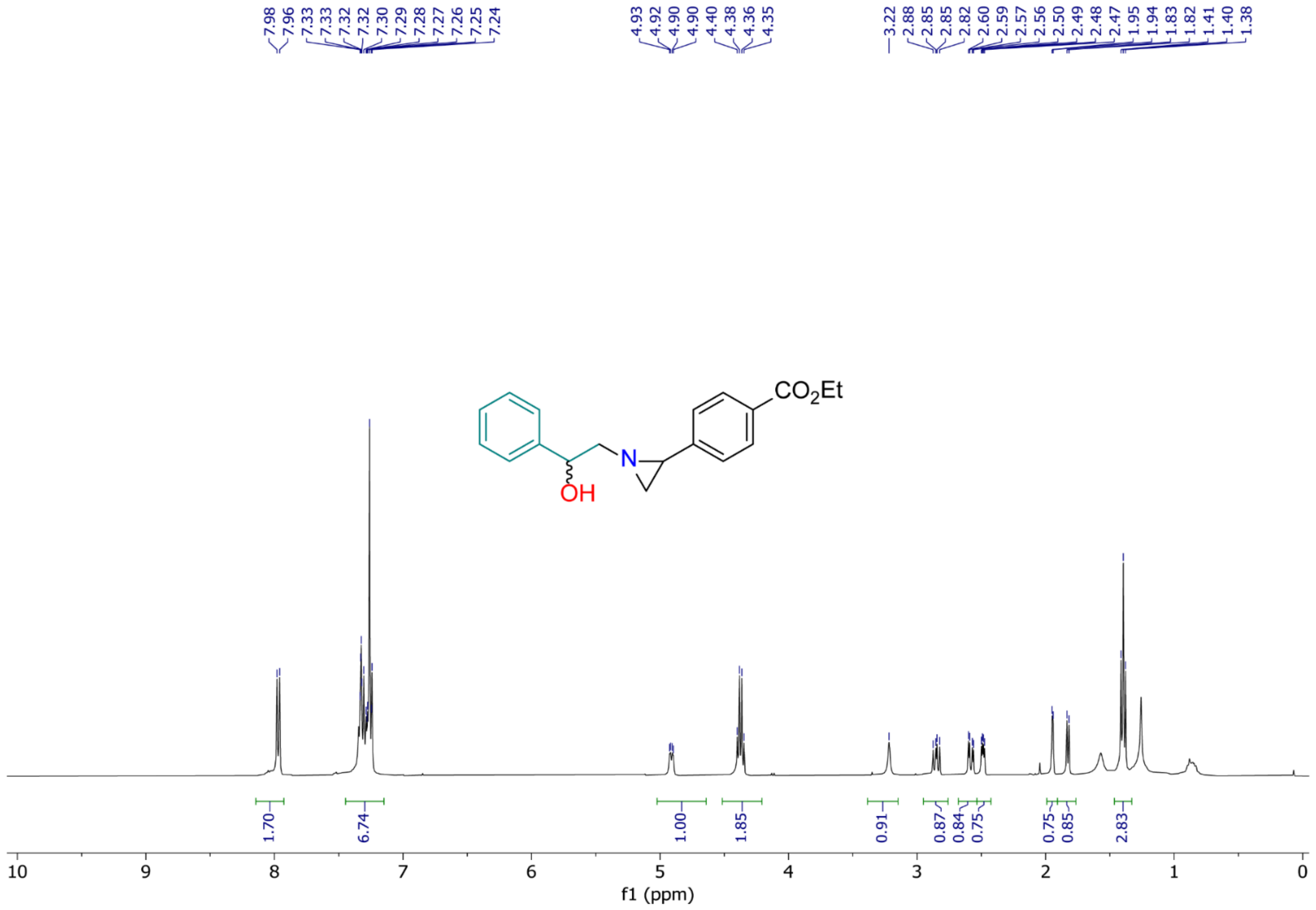

Figure S84. ^{1}H NMR spectrum of ethyl 4-(1-(2-hydroxy-2-phenylethyl)aziridin-2-yl)benzoate (**5u**) in $CDCl_3$ (400 MHz) at 23 °C.

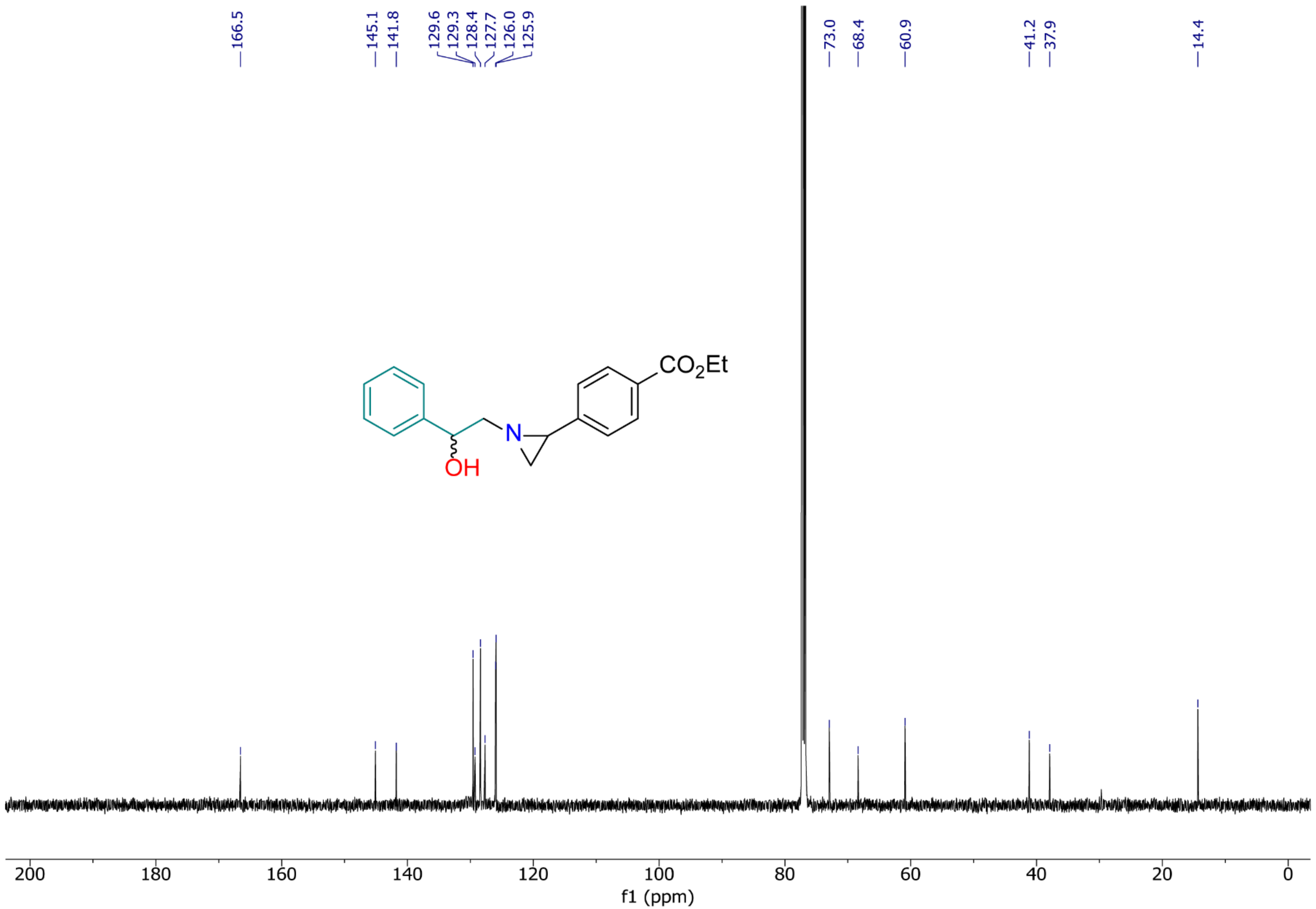

Figure S85. ^{13}C NMR spectrum of ethyl 4-(1-(2-hydroxy-2-phenylethyl)aziridin-2-yl)benzoate (**5u**) in $CDCl_3$ (101 MHz) at 23 °C.

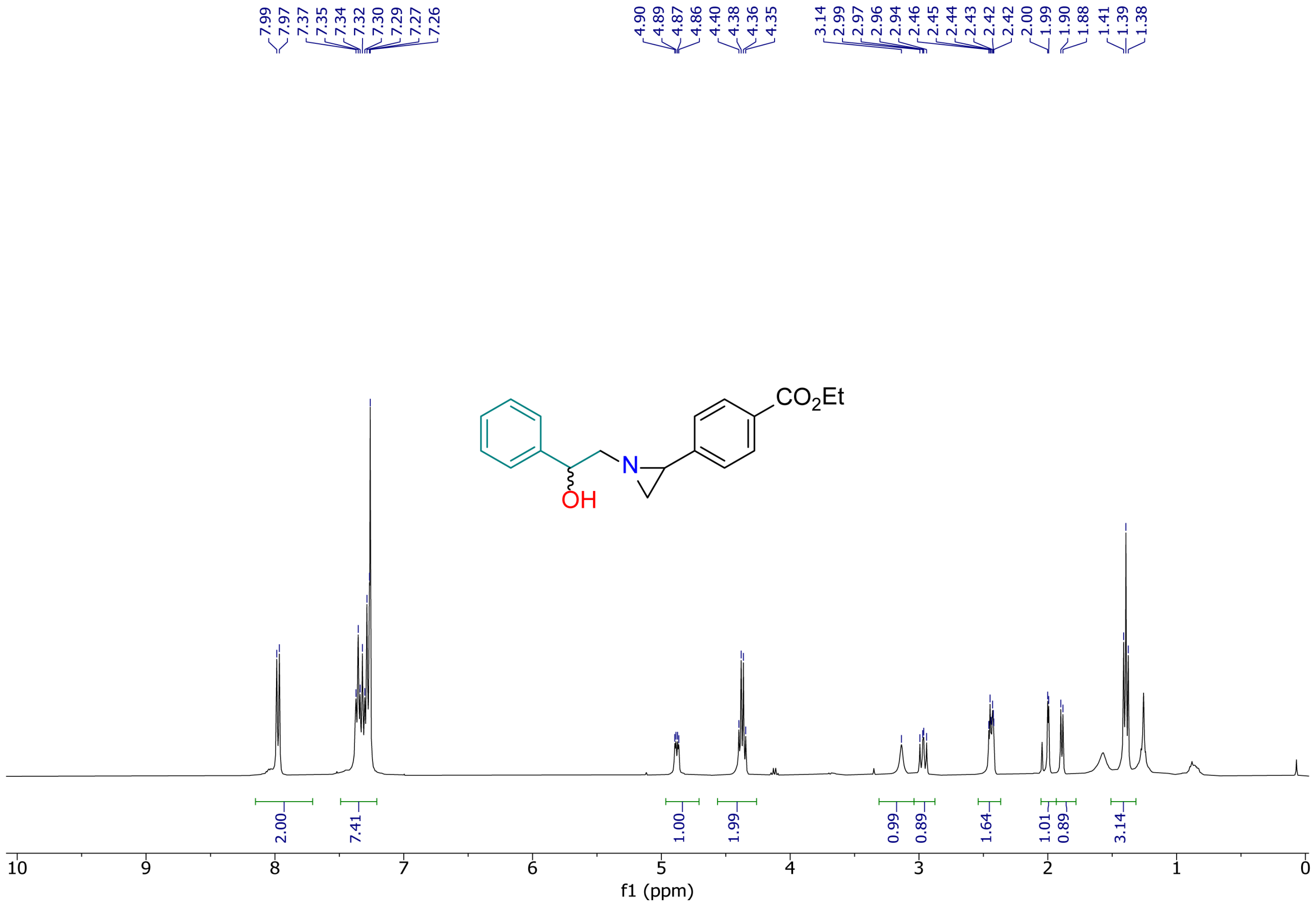

Figure S86. ^{1}H NMR spectrum of ethyl 4-(1-(2-hydroxy-2-phenylethyl)aziridin-2-yl)benzoate (**5u**) in $CDCl_3$ (400 MHz) at 23 °C.

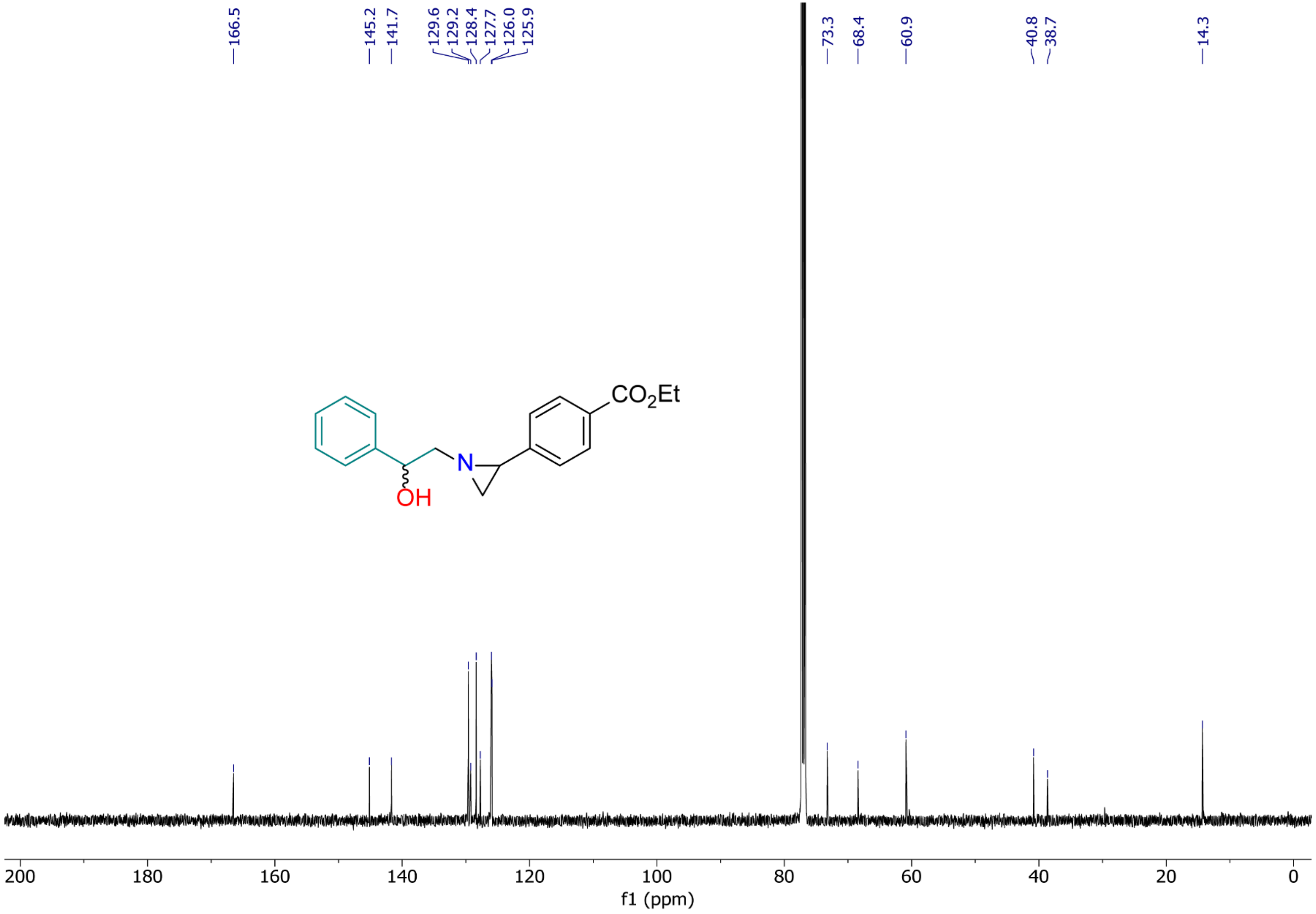

Figure S87. ^{13}C NMR spectrum of ethyl 4-(1-(2-hydroxy-2-phenylethyl)aziridin-2-yl)benzoate (**5u**) in $CDCl_3$ (101 MHz) at 23 °C.

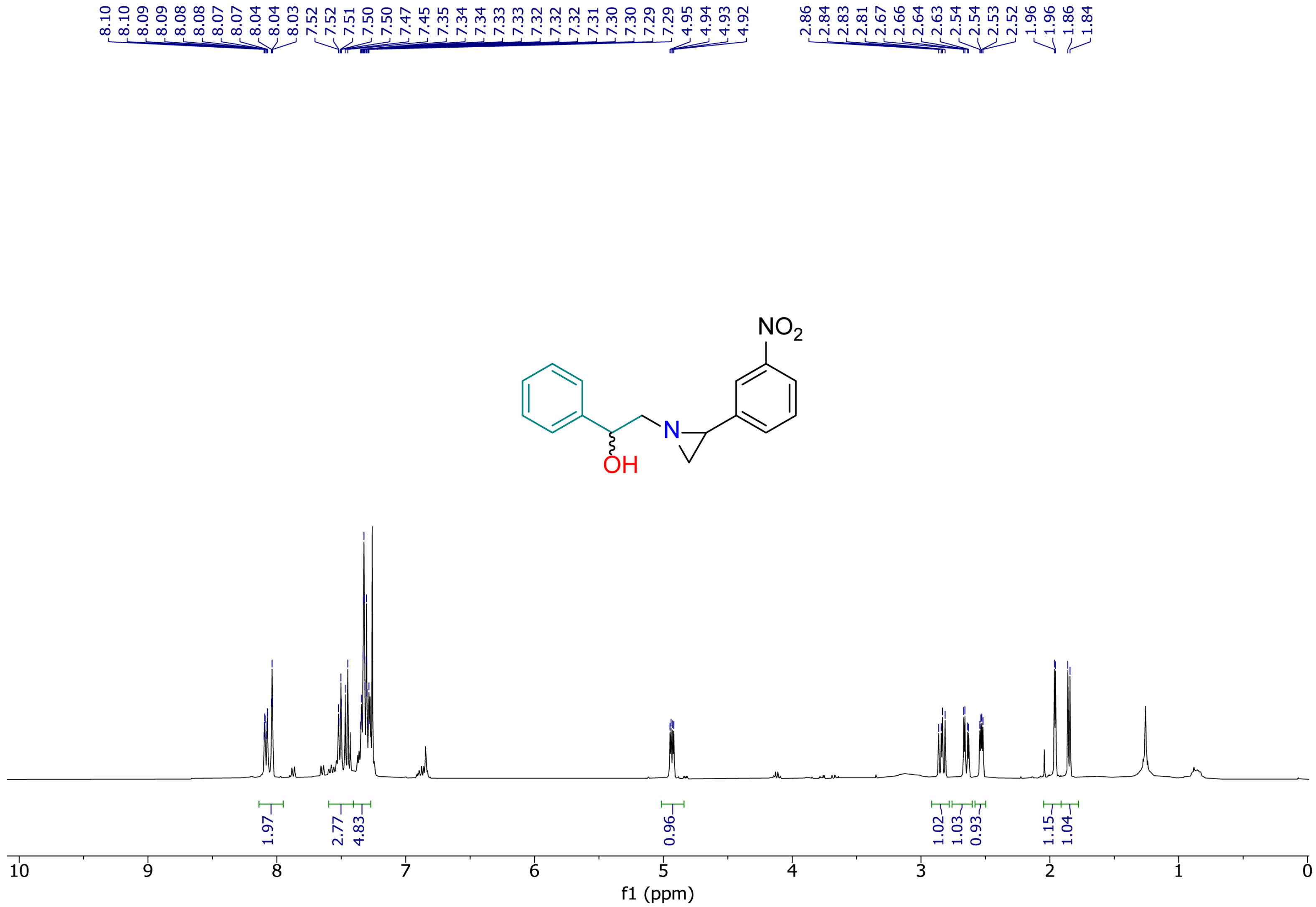

Figure S88. ^{1}H NMR spectrum of 2-(2-(3-nitrophenyl)aziridin-1-yl)-1-phenylethan-1-ol (**5v**) in $CDCl_3$ (400 MHz) at 23 °C.

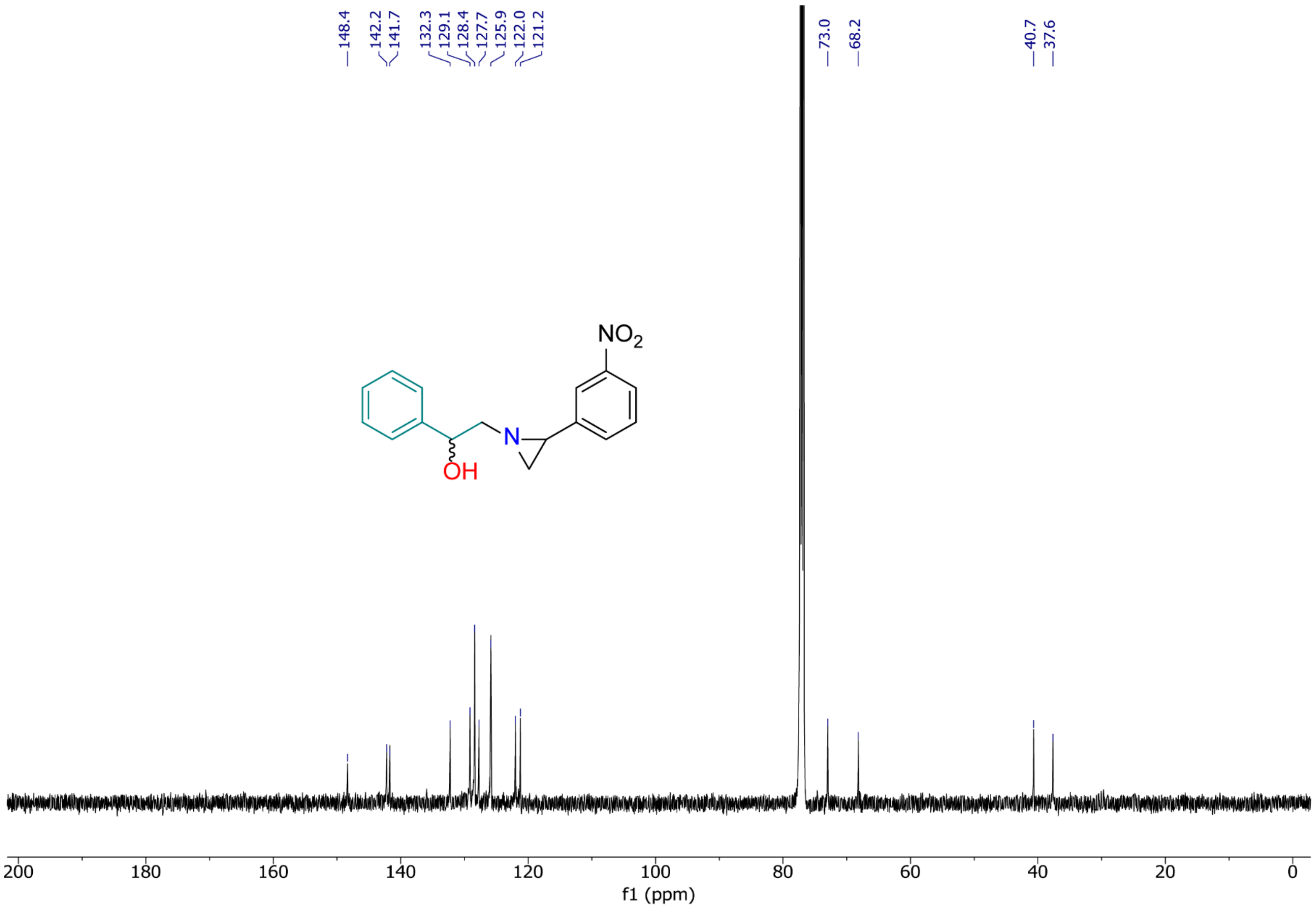

Figure S89. ^{13}C NMR spectrum of 2-(2-(3-nitrophenyl)aziridin-1-yl)-1-phenylethan-1-ol (**5v**) in $CDCl_3$ (126 MHz) at 23 °C

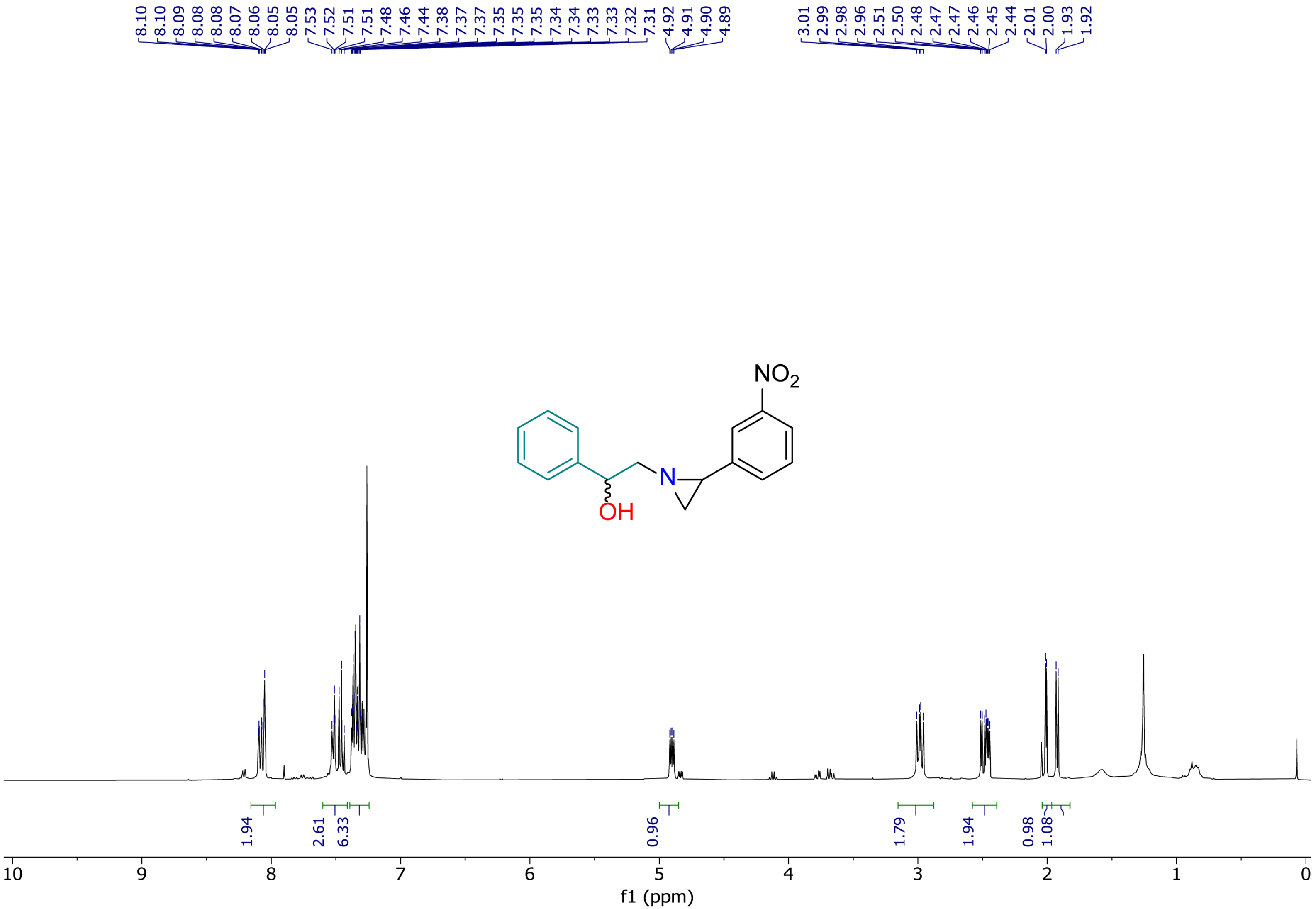

Figure S90. ^{1}H NMR spectrum of 2-(2-(3-nitrophenyl)aziridin-1-yl)-1-phenylethan-1-ol (**5v**) in $CDCl_3$ (400 MHz) at 23 °C.

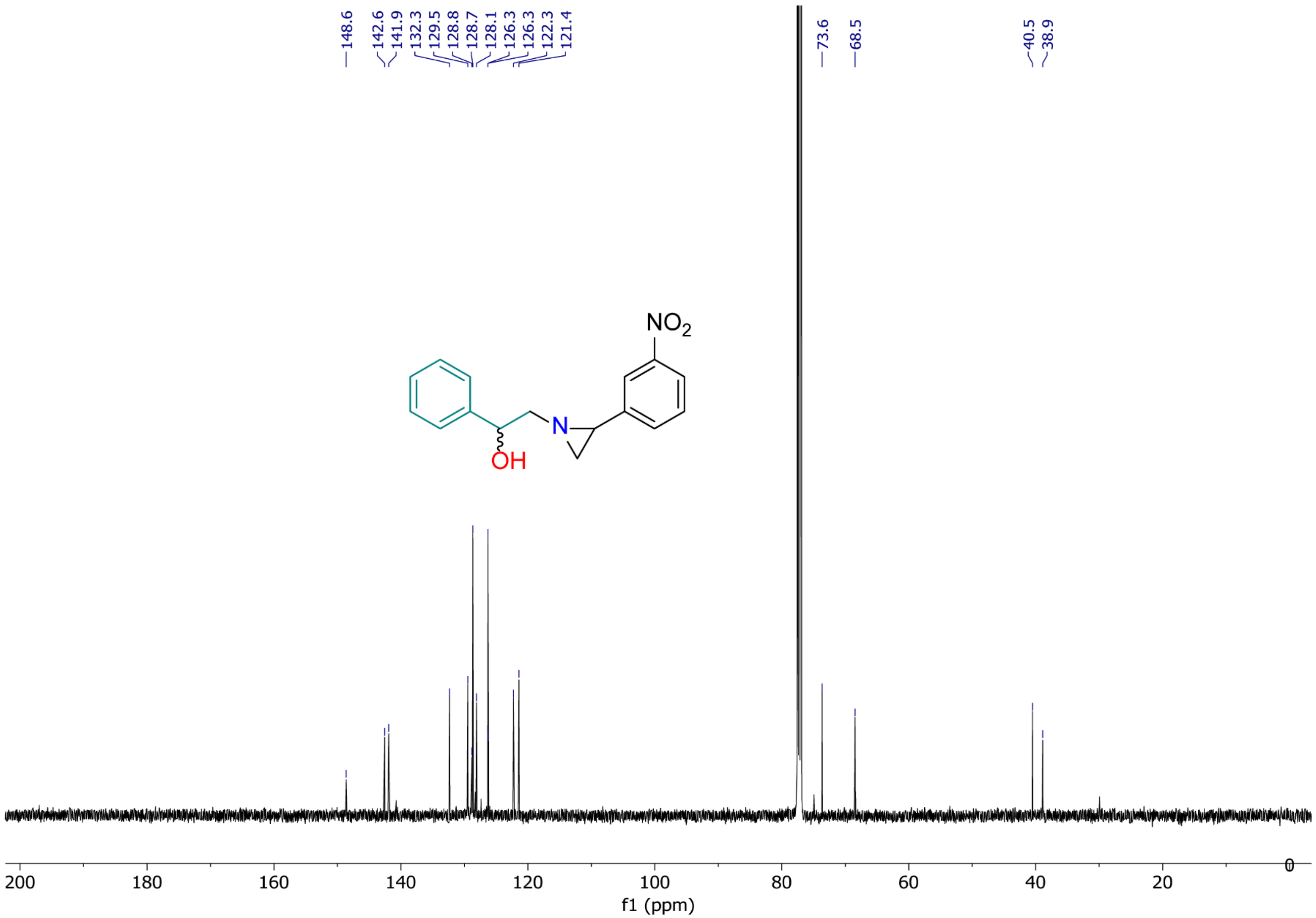

Figure S91. ^{13}C NMR spectrum of 2-(2-(3-nitrophenyl)aziridin-1-yl)-1-phenylethan-1-ol (**5v**) in $CDCl_3$ (126 MHz) at 23 °C

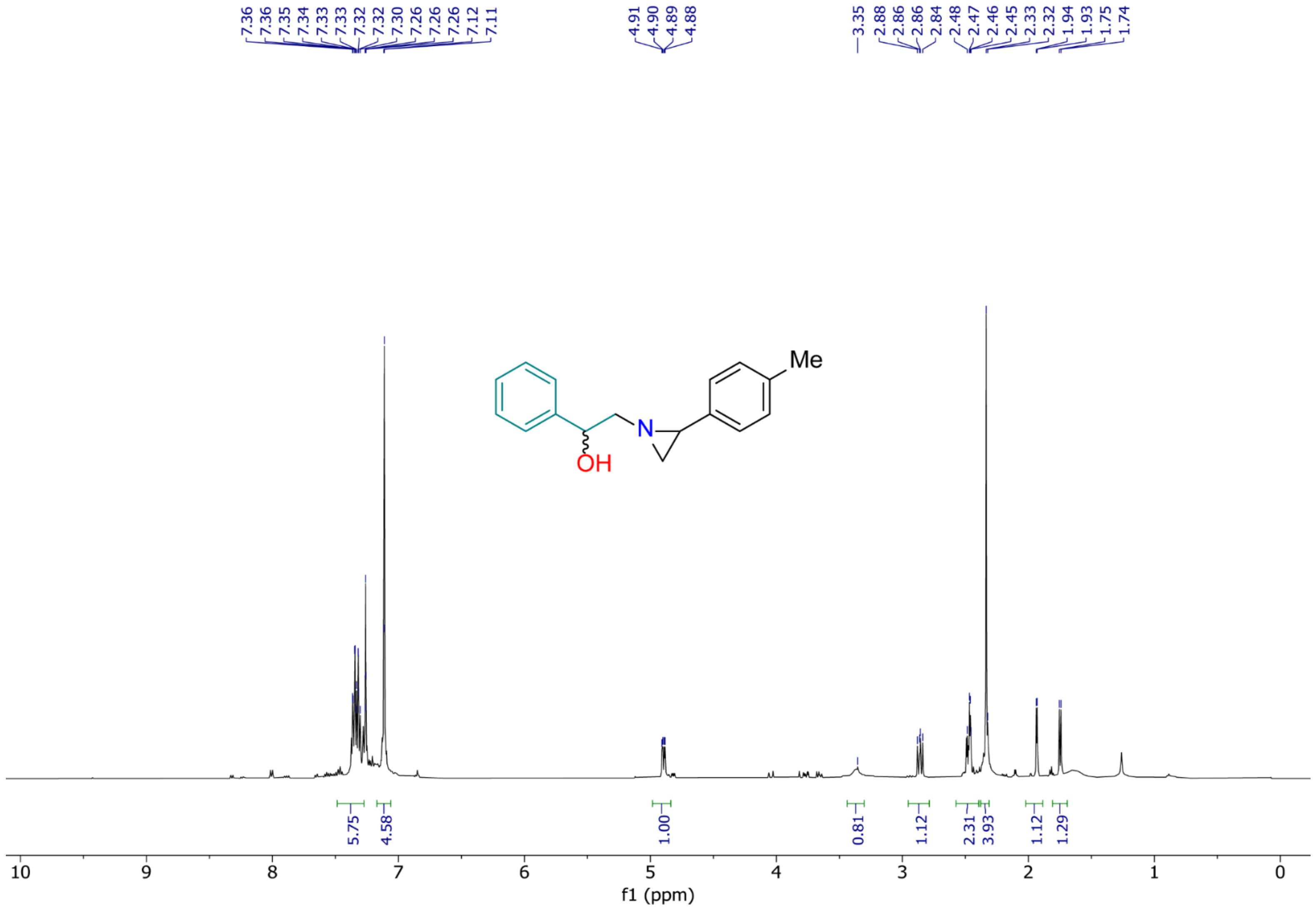

Figure S92. ^{1}H NMR spectrum of (1S)-1-phenyl-2-(2-(p-tolyl)aziridin-1-yl)ethan-1-ol (**5w**) in $CDCl_3$ (400 MHz) at 23 °C.

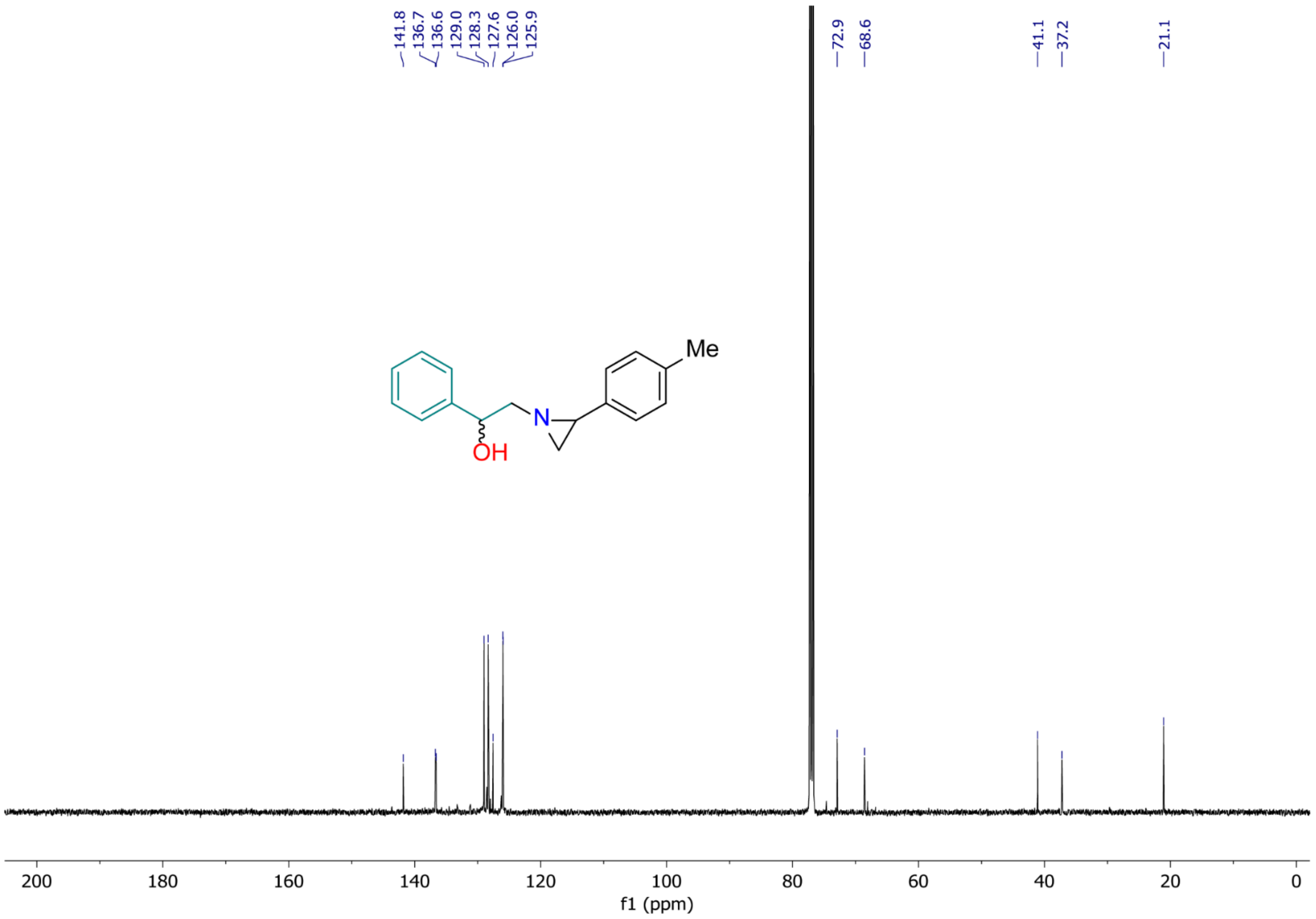

Figure S93. ^{13}C NMR spectrum of (1S)-1-phenyl-2-(2-(p-tolyl)aziridin-1-yl)ethan-1-ol (**5w**) in $CDCl_3$ (101 MHz) at 23 °C.

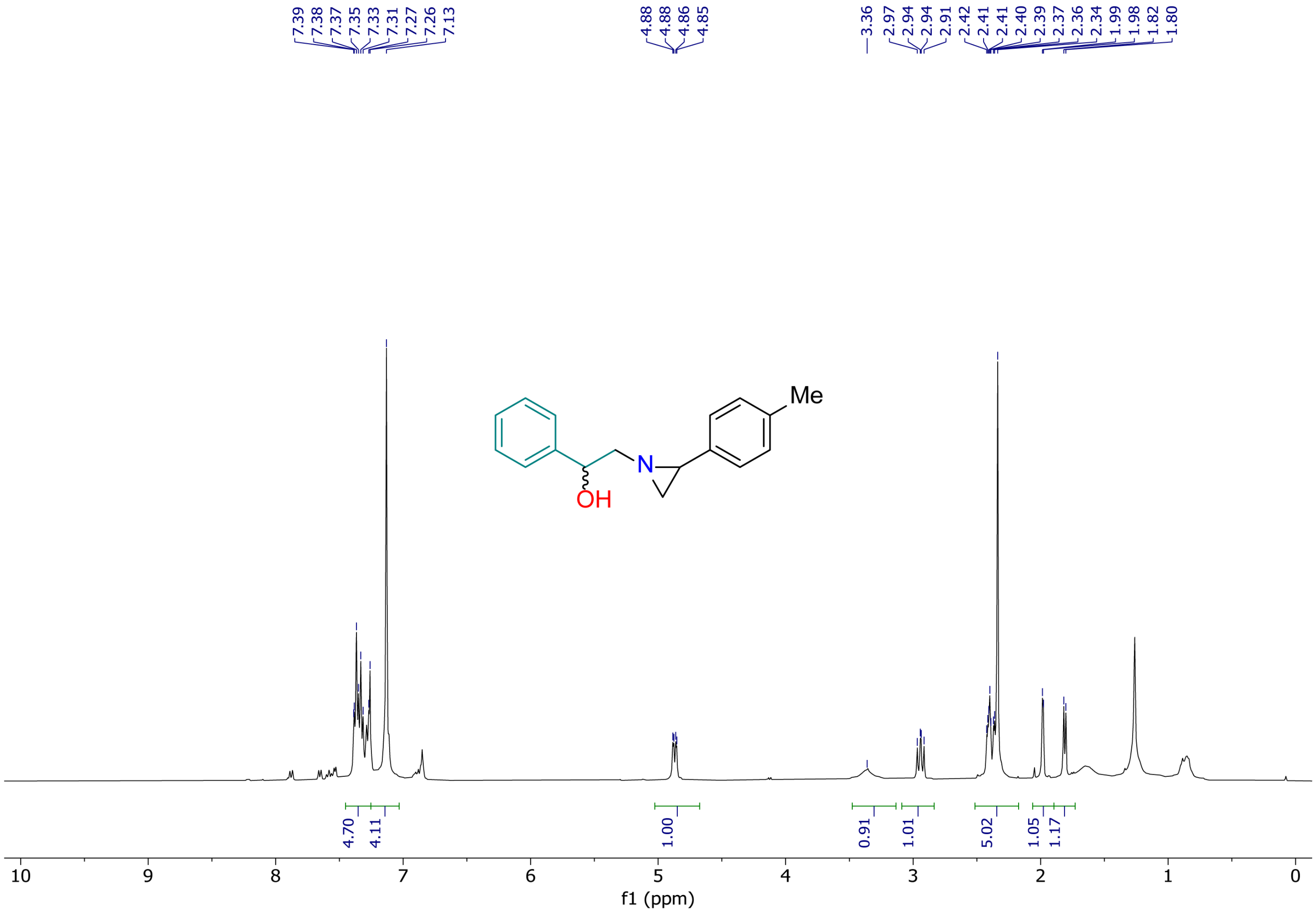

Figure S94. ^{1}H NMR spectrum of (1S)-1-phenyl-2-(2-(p-tolyl)aziridin-1-yl)ethan-1-ol (**5w**) in $CDCl_3$ (400 MHz) at 23 °C.

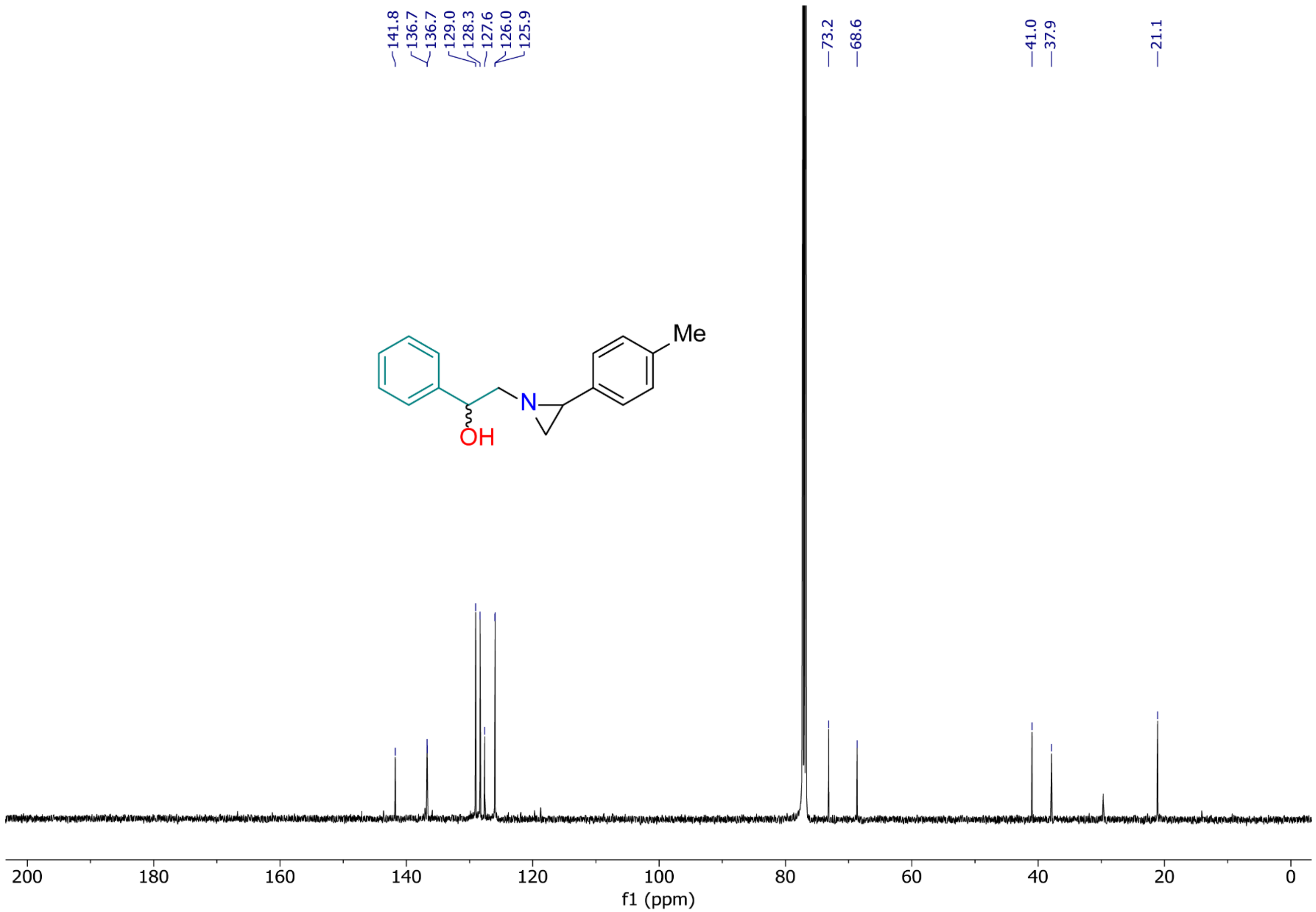

Figure S95. ^{13}C NMR spectrum of (1S)-1-phenyl-2-(2-(p-tolyl)aziridin-1-yl)ethan-1-ol (**5w**) in $CDCl_3$ (101 MHz) at 23 °C.

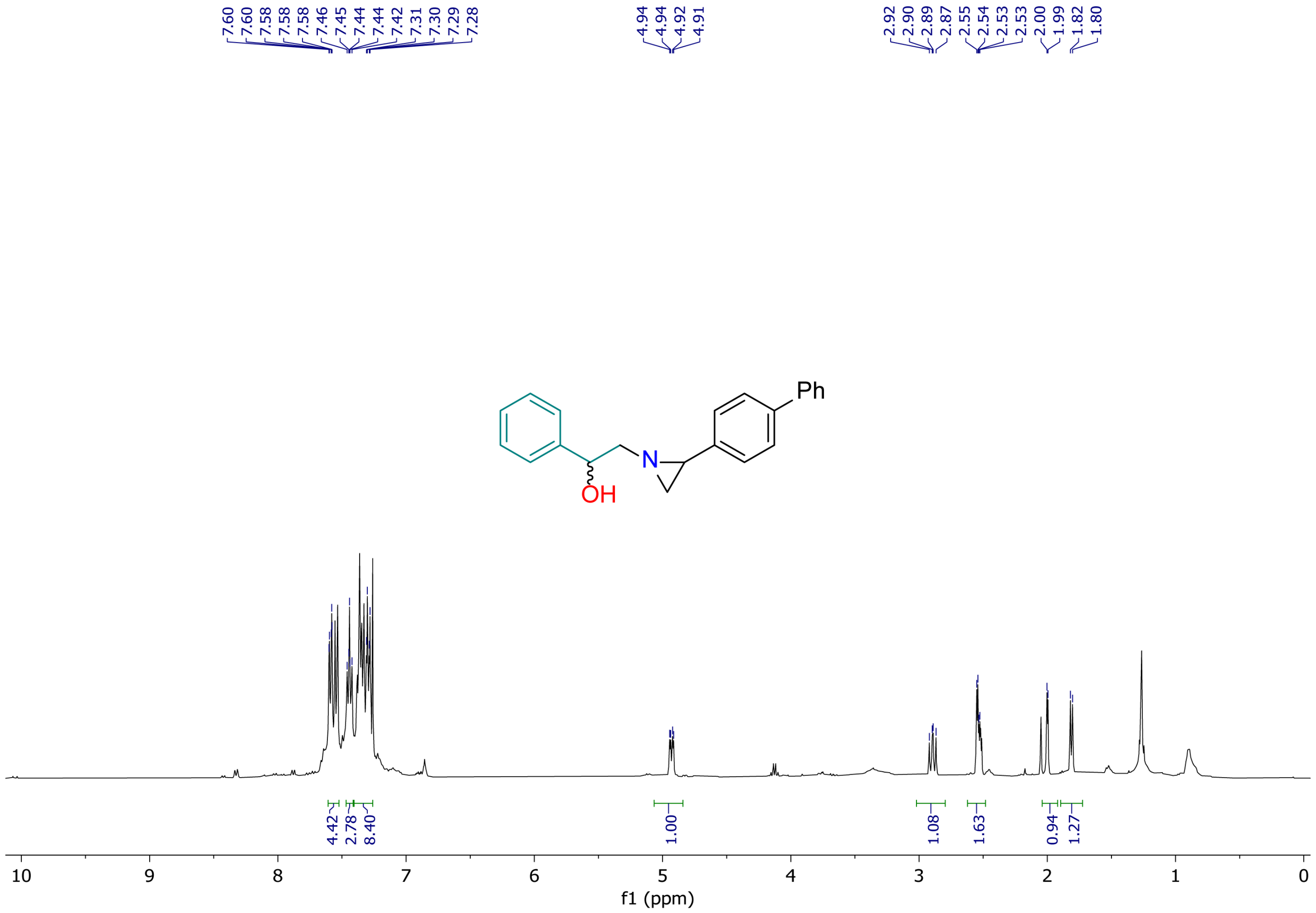

Figure S96. ^{1}H NMR spectrum of 2-(2-([1,1'-biphenyl]-4-yl)aziridin-1-yl)-1-phenylethan-1-ol (**5x**) in $CDCl_3$ (101 MHz) at 23 °C.

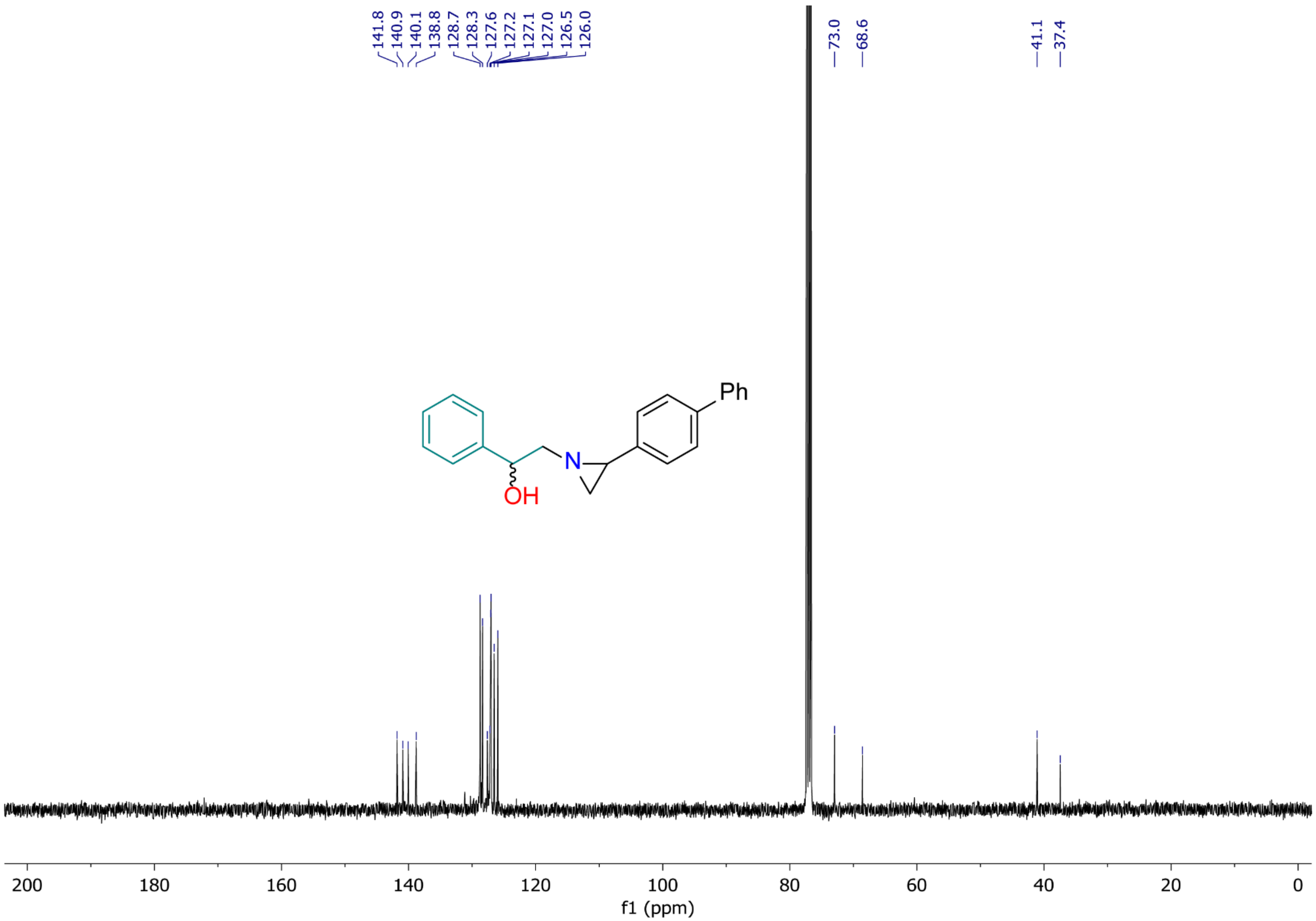

Figure S97. ^{13}C NMR spectrum of 2-(2-([1,1'-biphenyl]-4-yl)aziridin-1-yl)-1-phenylethan-1-ol (**5x**) in $CDCl_3$ (101 MHz) at 23 °C

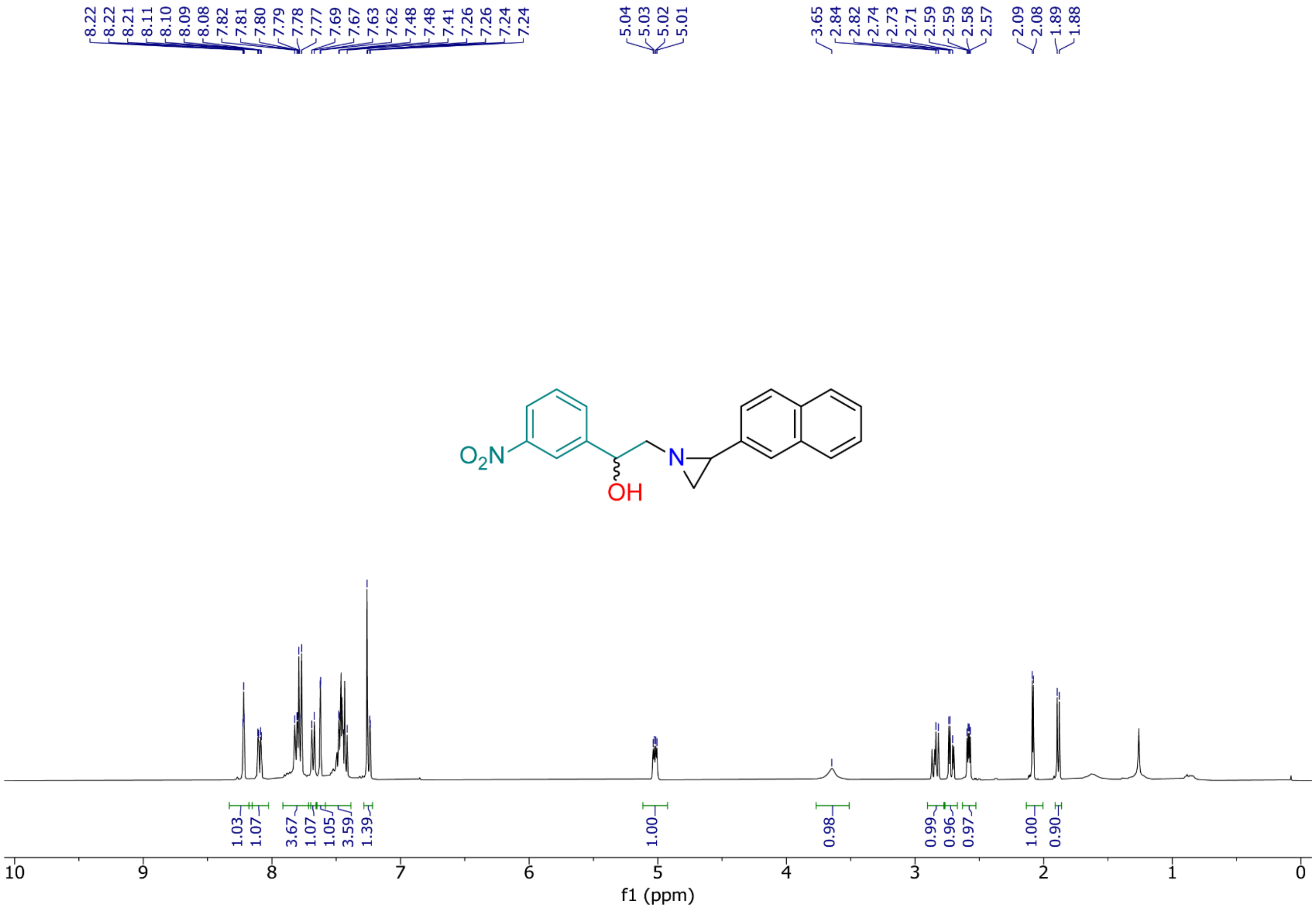

Figure S98. ^{1}H NMR spectrum of 2-(2-(naphthalen-2-yl)aziridin-1-yl)-1-(3-nitrophenyl)ethan-1-ol (**5y**) in $CDCl_3$ (400 MHz) at 23 °C.

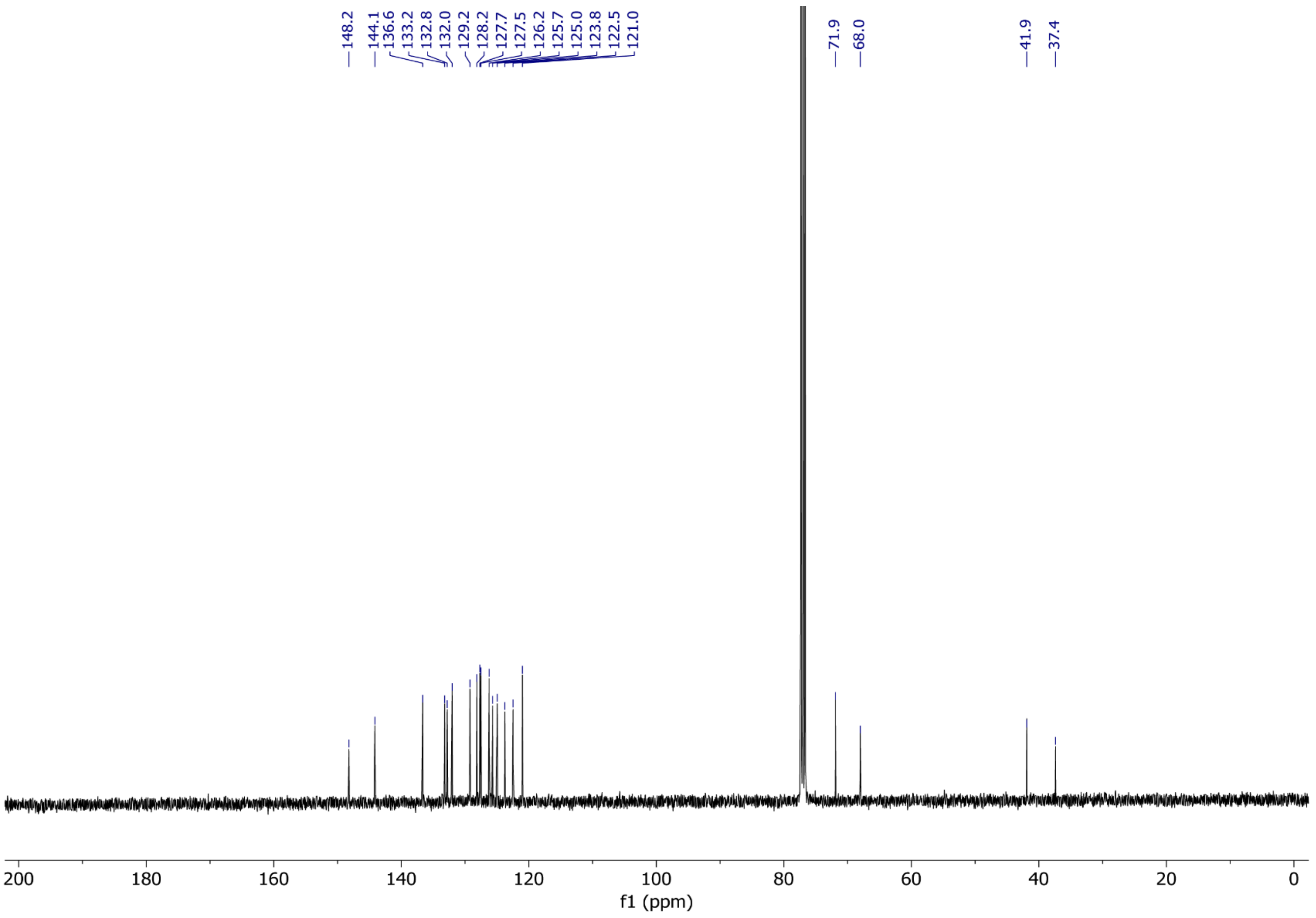

Figure S99. ^{13}C NMR spectrum of 2-(2-(naphthalen-2-yl)aziridin-1-yl)-1-(3-nitrophenyl)ethan-1-ol (**5y**) in $CDCl_3$ (101 MHz) at 23 °C

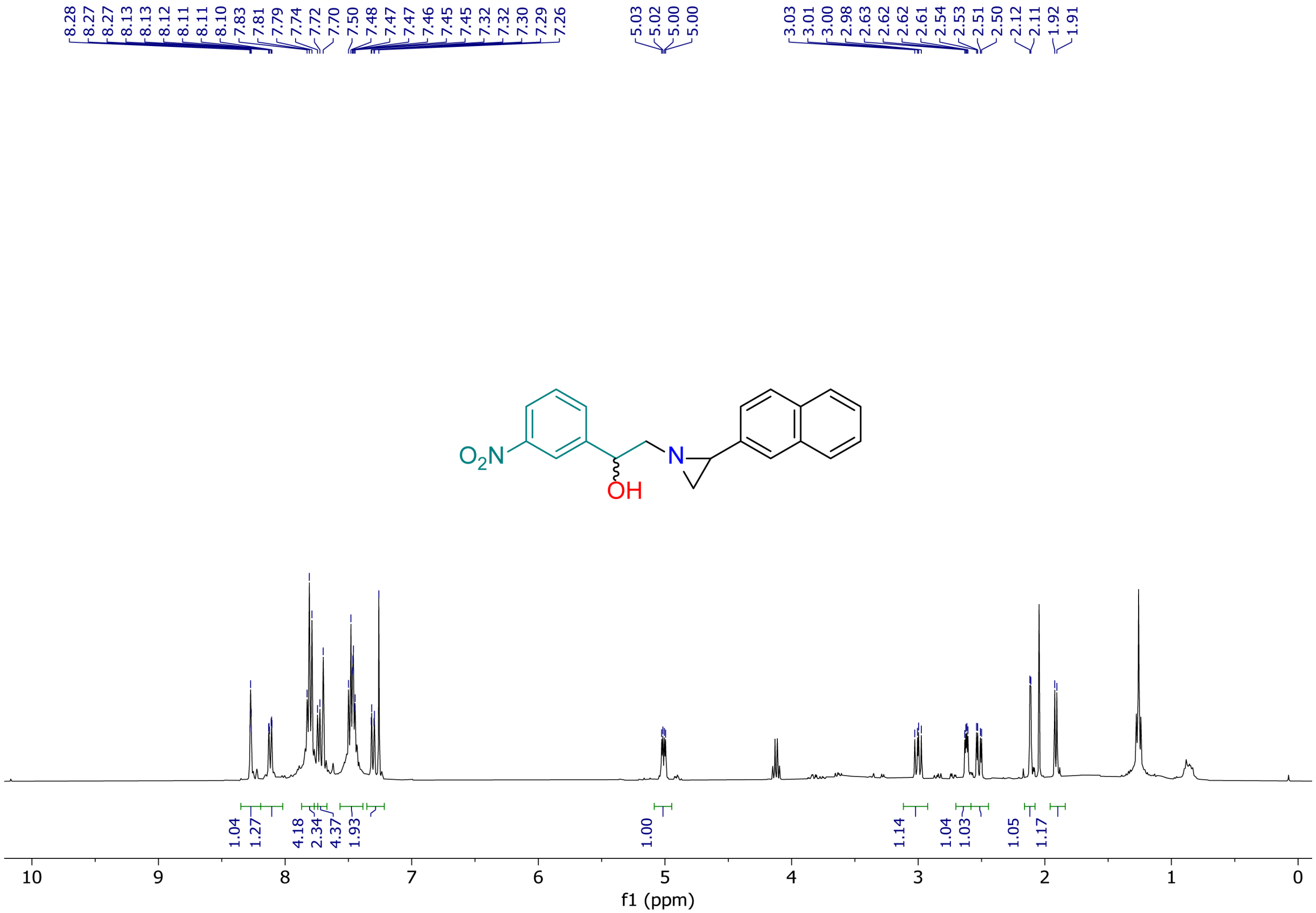

Figure S100. ^{1}H NMR spectrum of 2-(2-(naphthalen-2-yl)aziridin-1-yl)-1-(3-nitrophenyl)ethan-1-ol (**5y**) in $CDCl_3$ (500 MHz) at 23 °C.

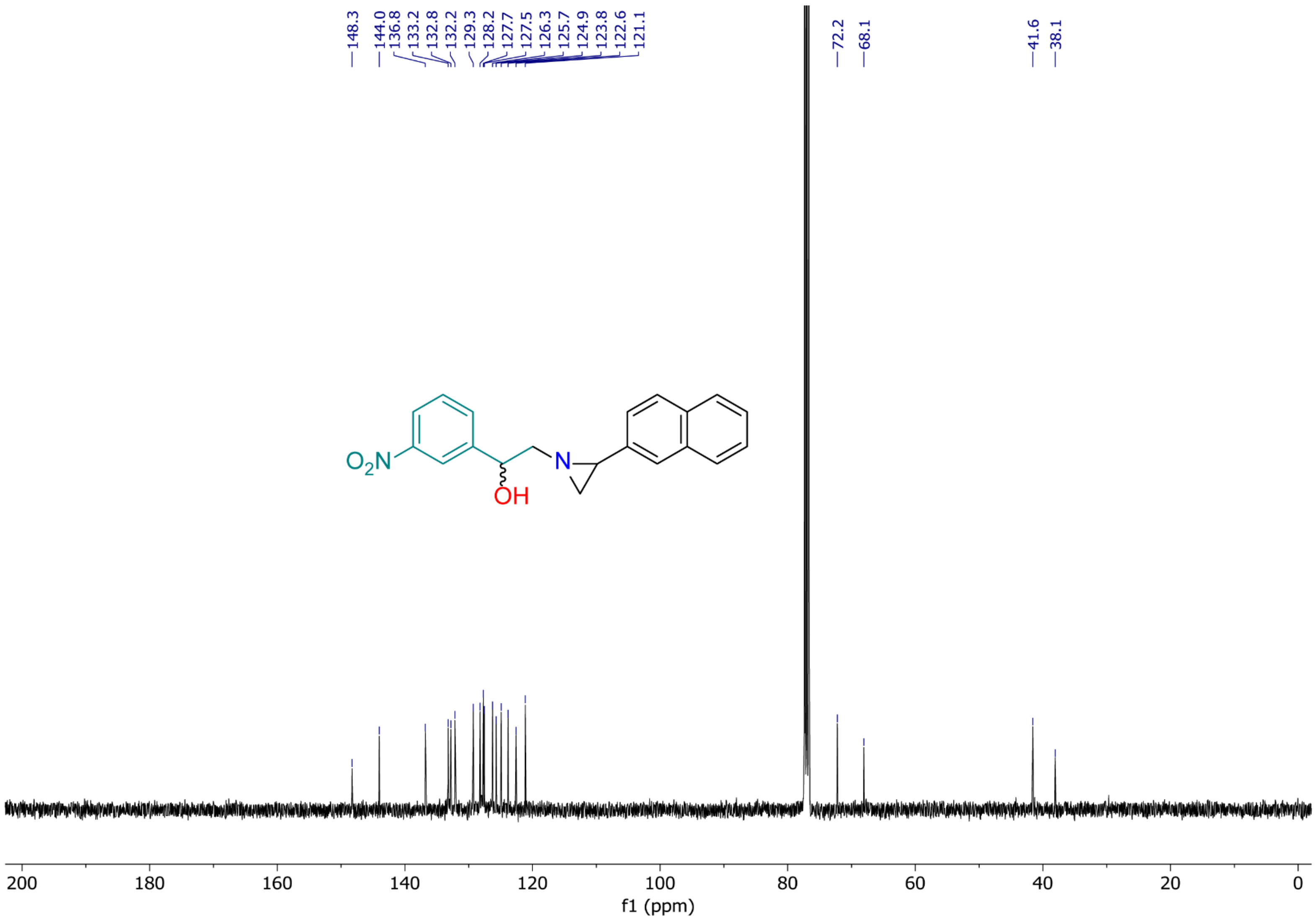

Figure S101. ^{13}C NMR spectrum of 2-(2-(naphthalen-2-yl)aziridin-1-yl)-1-(3-nitrophenyl)ethan-1-ol (**5y**) in $CDCl_3$ (101 MHz) at 23 °C.

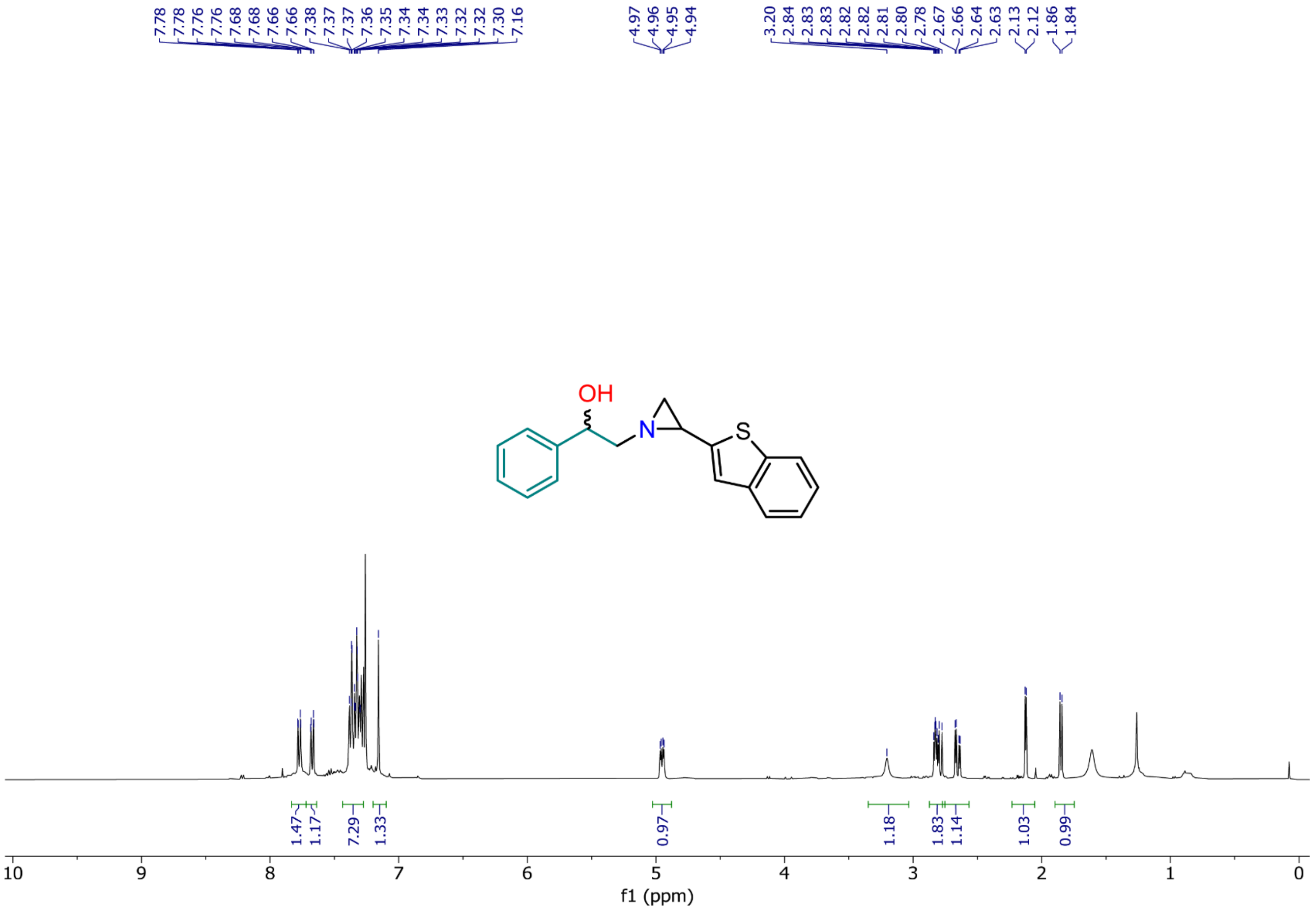

Figure S102. ^{1}H NMR spectrum of 2-(2-(benzo[b]thiophen-2-yl)aziridin-1-yl)-1-phenylethan-1-ol (**5z**) in $CDCl_3$ (400 MHz) at 23 °C.

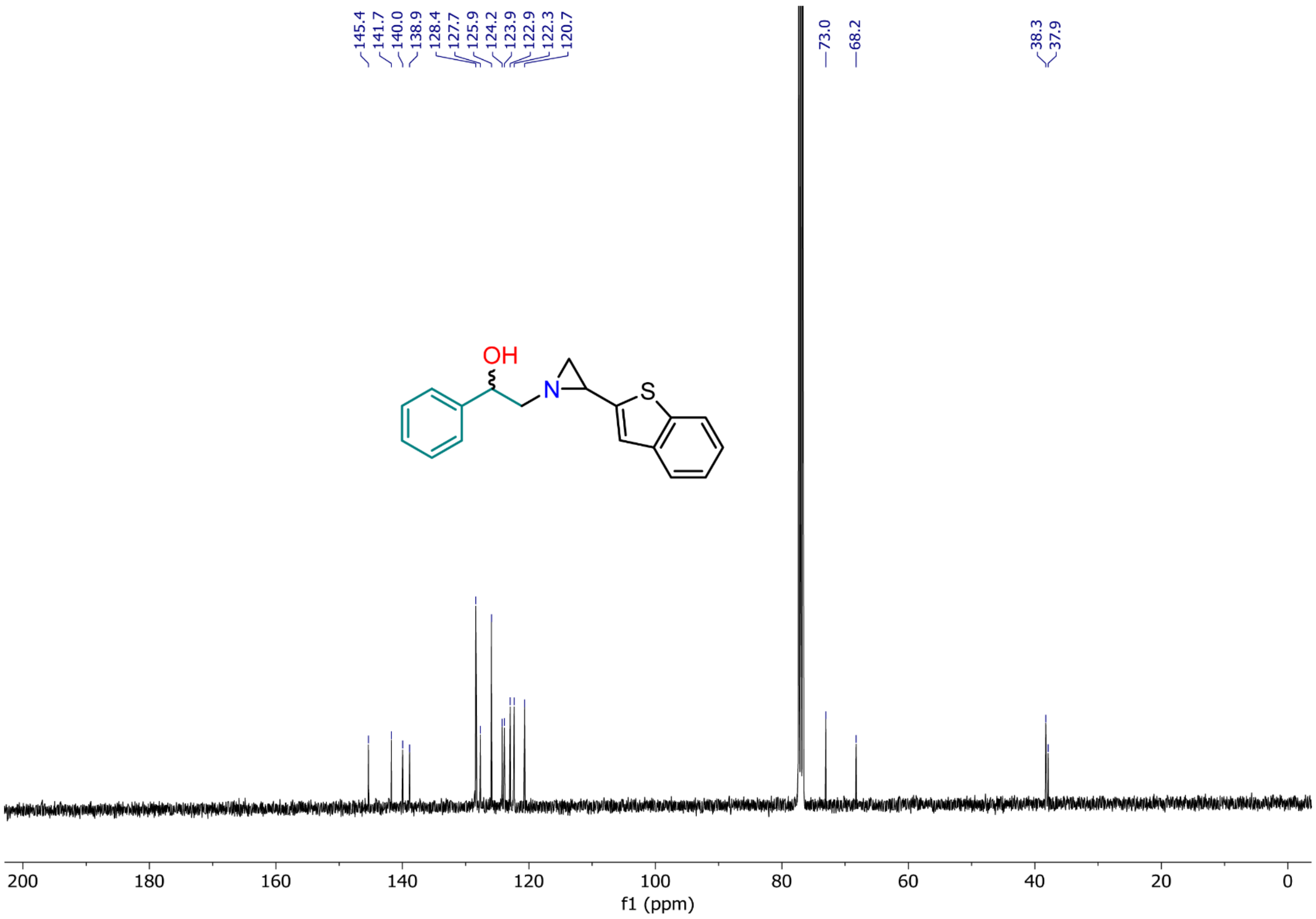

Figure S103. ^{13}C NMR spectrum of 2-(2-(benzo[b]thiophen-2-yl)aziridin-1-yl)-1-phenylethan-1-ol (**5z**) in $CDCl_3$ (126 MHz) at 23 °C.

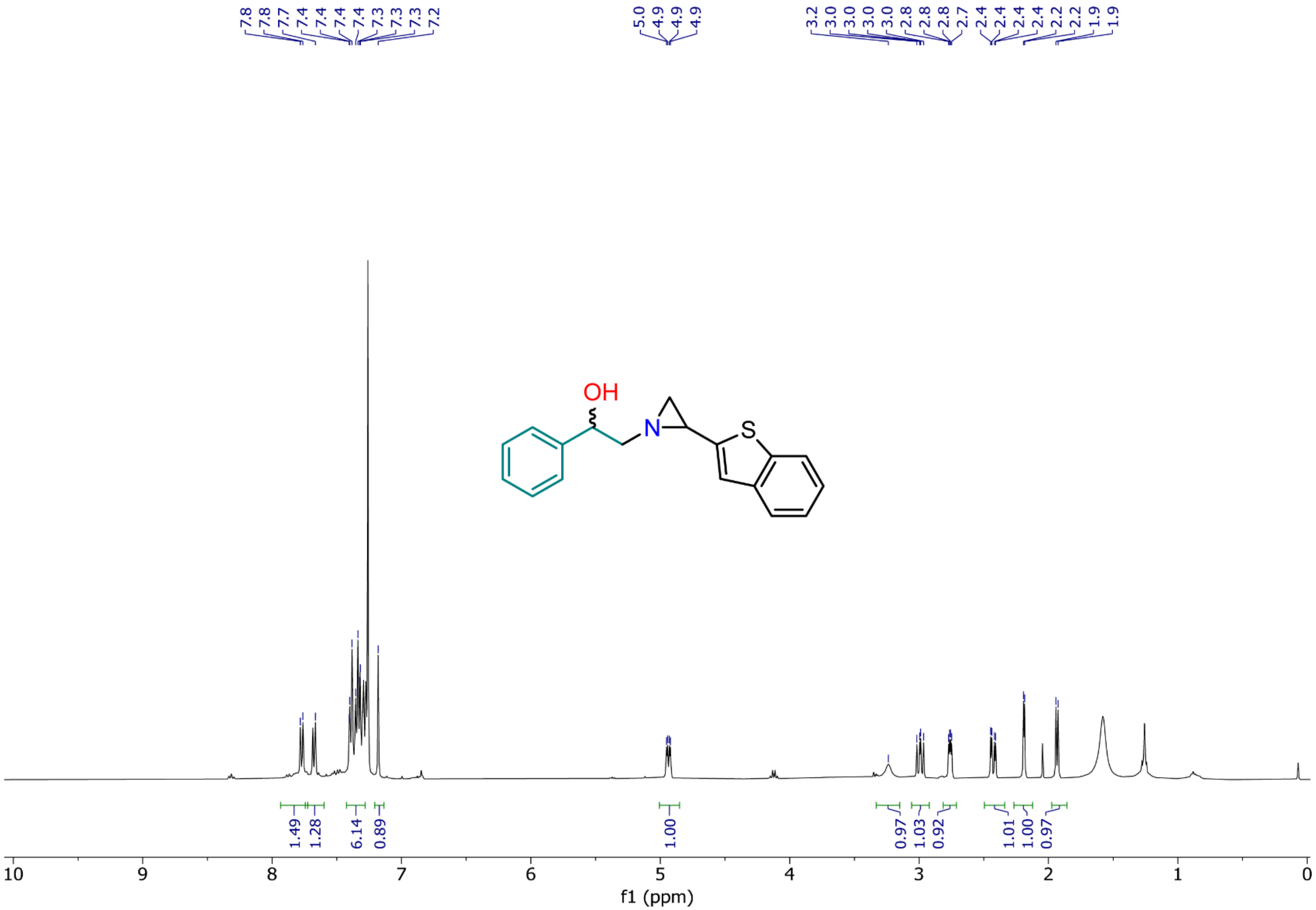

Figure S104. ^{1}H NMR spectrum of 2-(2-(benzo[b]thiophen-2-yl)aziridin-1-yl)-1-phenylethan-1-ol (**5z**) in $CDCl_3$ (400 MHz) at 23 °C.

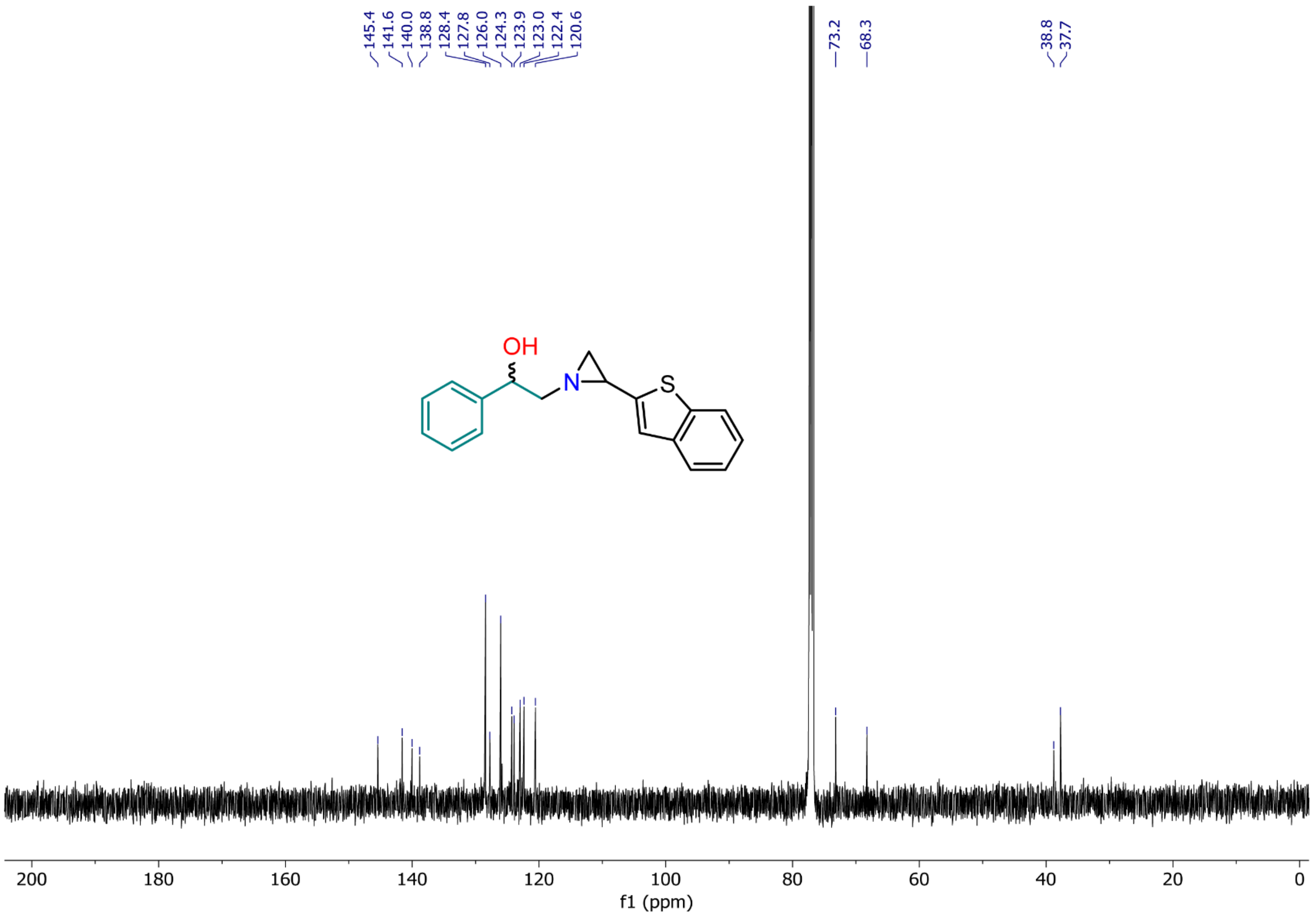

Figure S105. ^{13}C NMR spectrum of 2-(2-(benzo[b]thiophen-2-yl)aziridin-1-yl)-1-phenylethan-1-ol (**5z**) in $CDCl_3$ (126 MHz) at 23 °C.

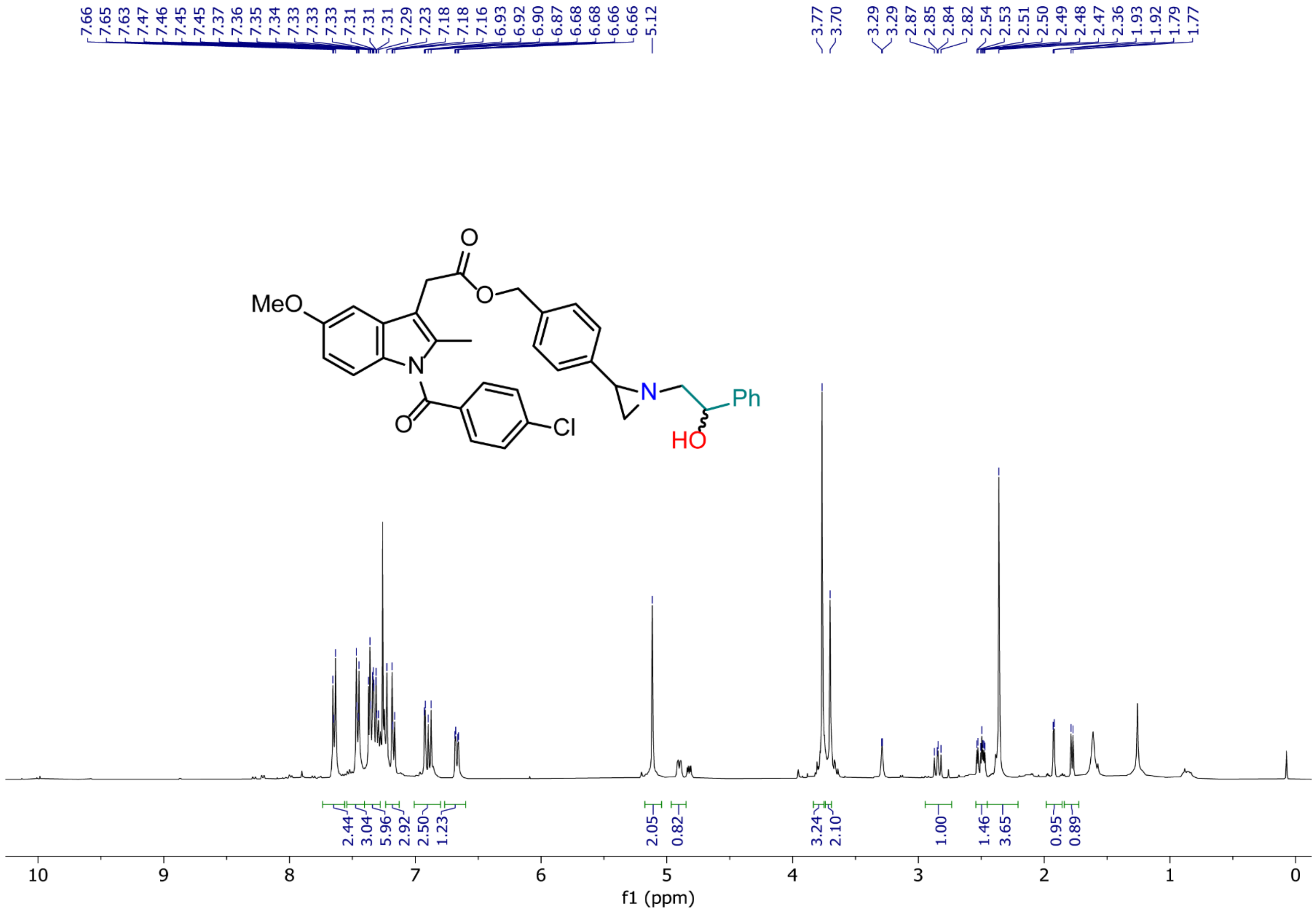

Figure S106. ^{1}H NMR spectrum of 4-(1-(2-hydroxy-2-phenylethyl)aziridin-2-yl)benzyl 2-(1-(4-chlorobenzoyl)-5-methoxy-2-methyl-1H-indol-3-yl)acetate (**5aa**) in $CDCl_3$ (400 MHz) at 23 °C.

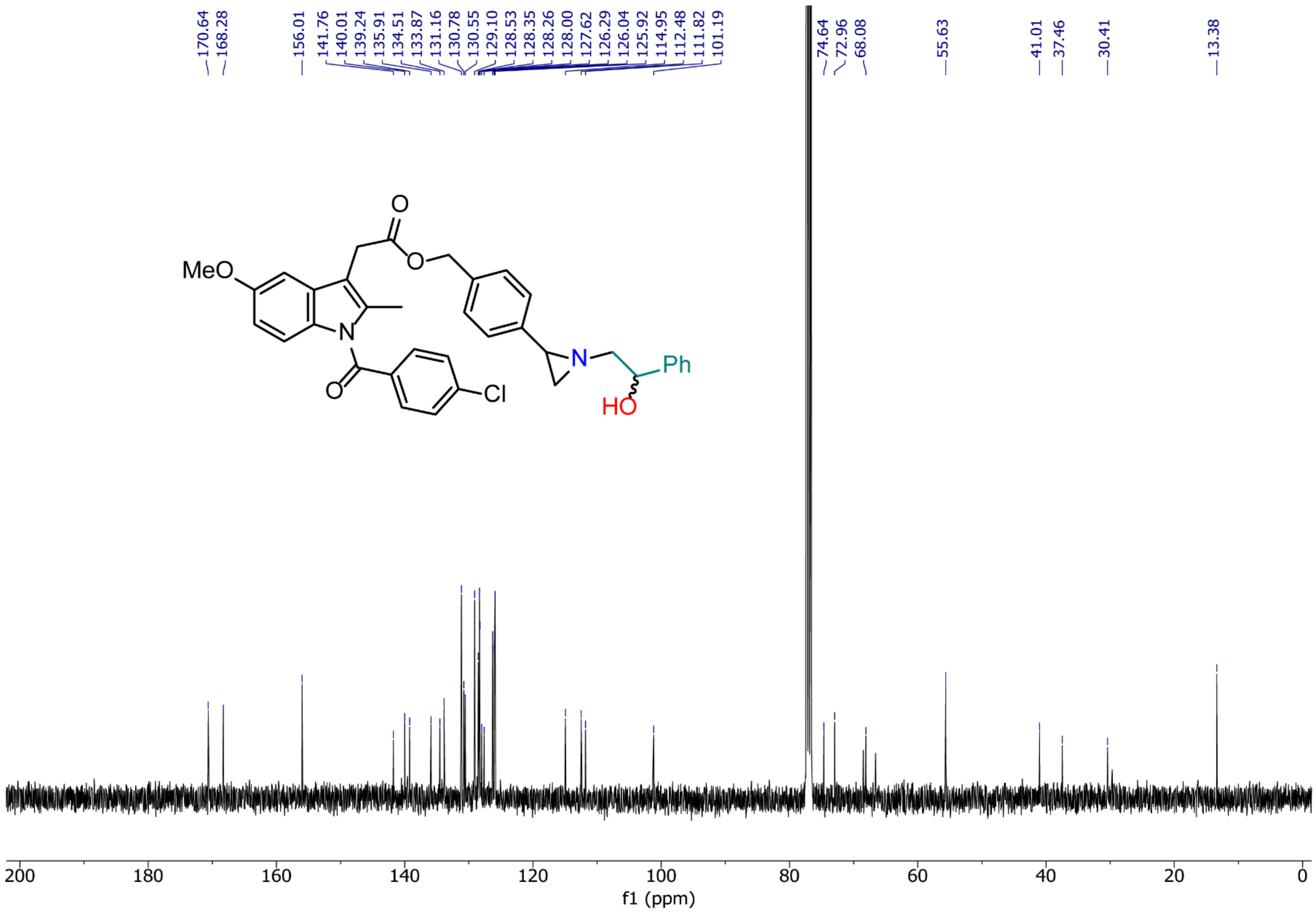

Figure S107. ^{13}C NMR spectrum of 4-(1-(2-hydroxy-2-phenylethyl)aziridin-2-yl)benzyl 2-(1-(4-chlorobenzoyl)-5-methoxy-2-methyl-1H-indol-3-yl)acetate (**5aa**) in $CDCl_3$ (101 MHz) at 23 °C.

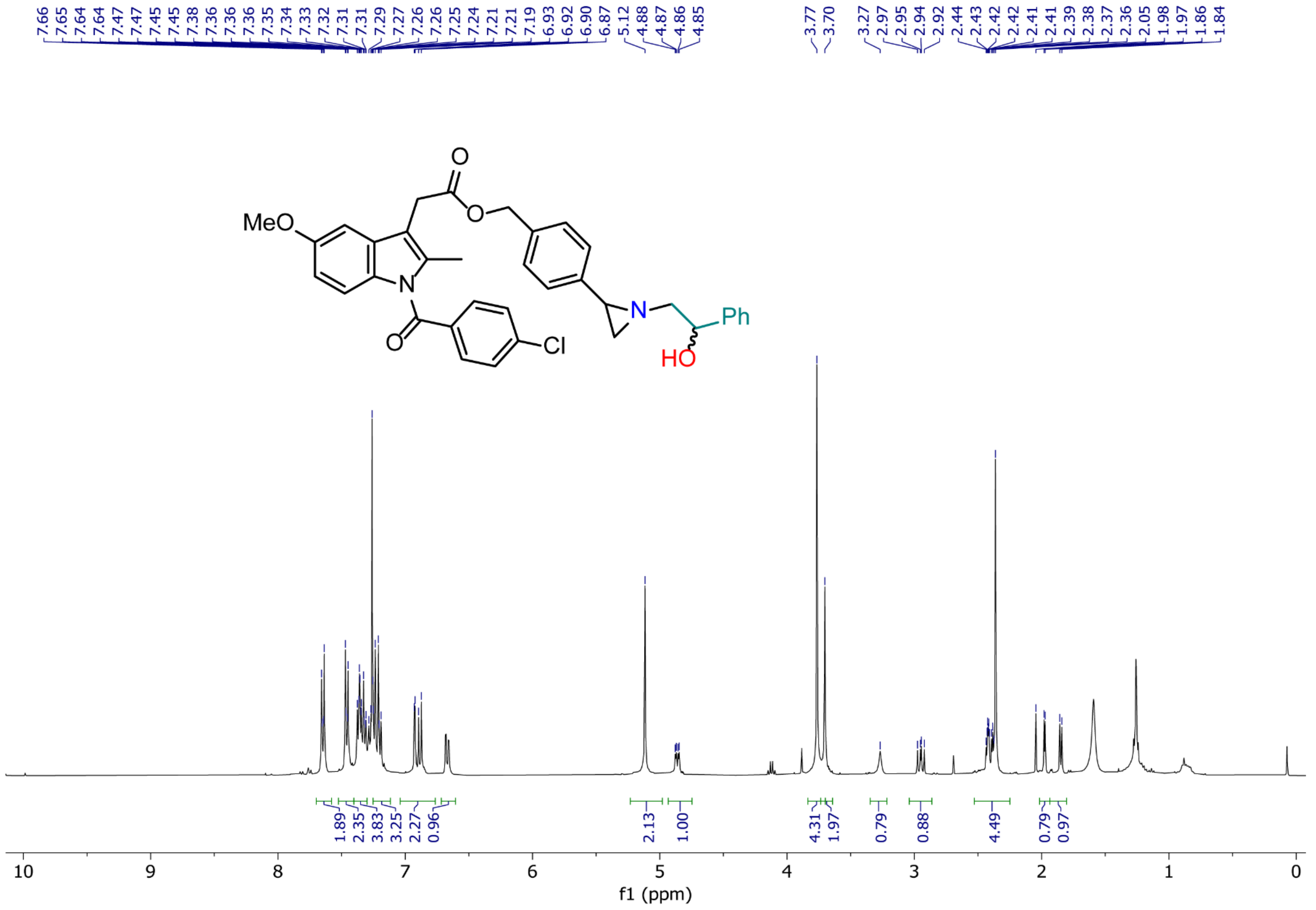

Figure S108. ^{1}H NMR spectrum of 4-(1-(2-hydroxy-2-phenylethyl)aziridin-2-yl)benzyl 2-(1-(4-chlorobenzoyl)-5-methoxy-2-methyl-1H-indol-3-yl)acetate (**5aa**) in $CDCl_3$ (400 MHz) at 23 °C.

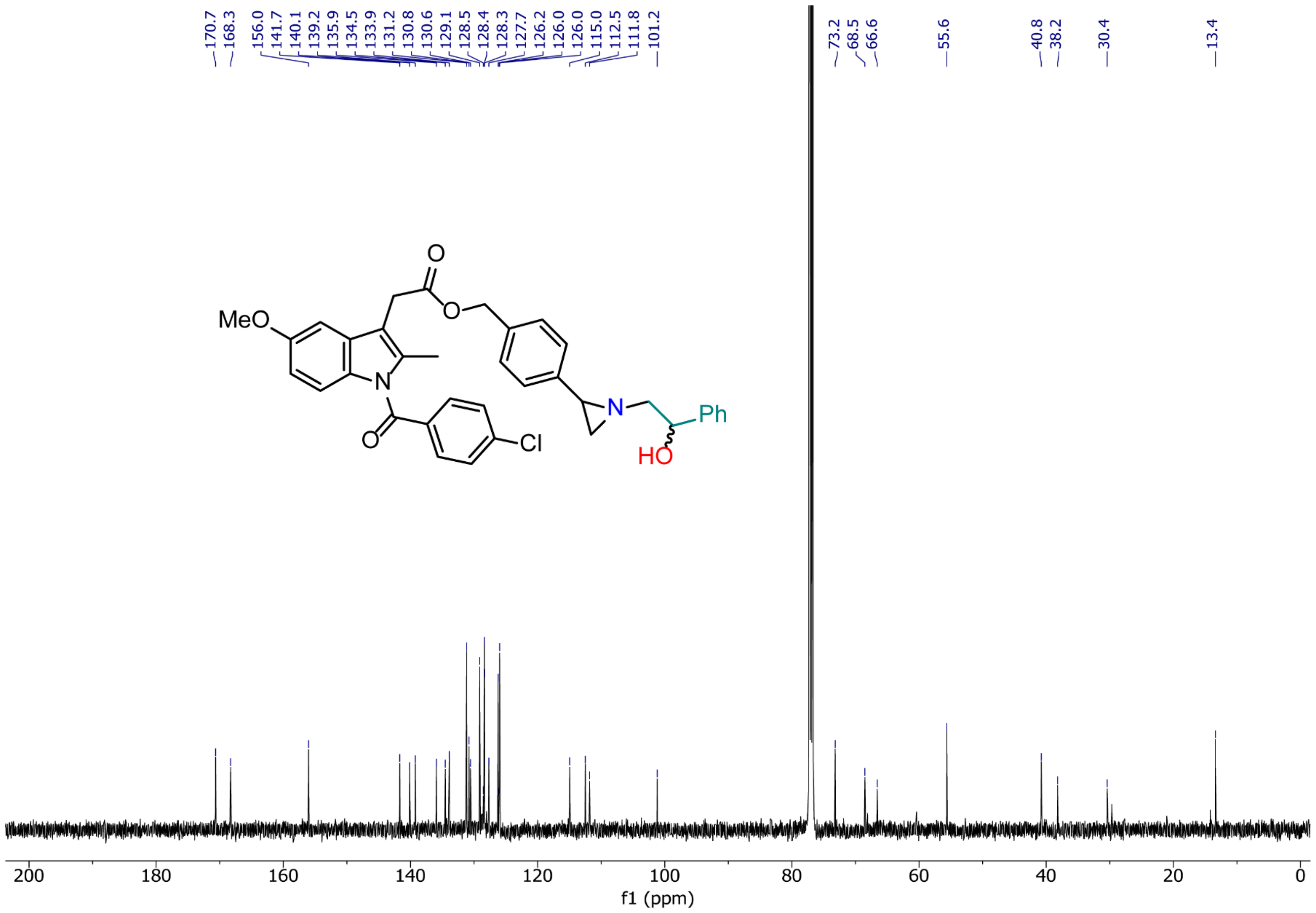

Figure S109. ^{13}C NMR spectrum of 4-(1-(2-hydroxy-2-phenylethyl)aziridin-2-yl)benzyl 2-(1-(4-chlorobenzoyl)-5-methoxy-2-methyl-1H-indol-3-yl)acetate **(5aa)** in $CDCl_3$ (126 MHz) at 23 °C.

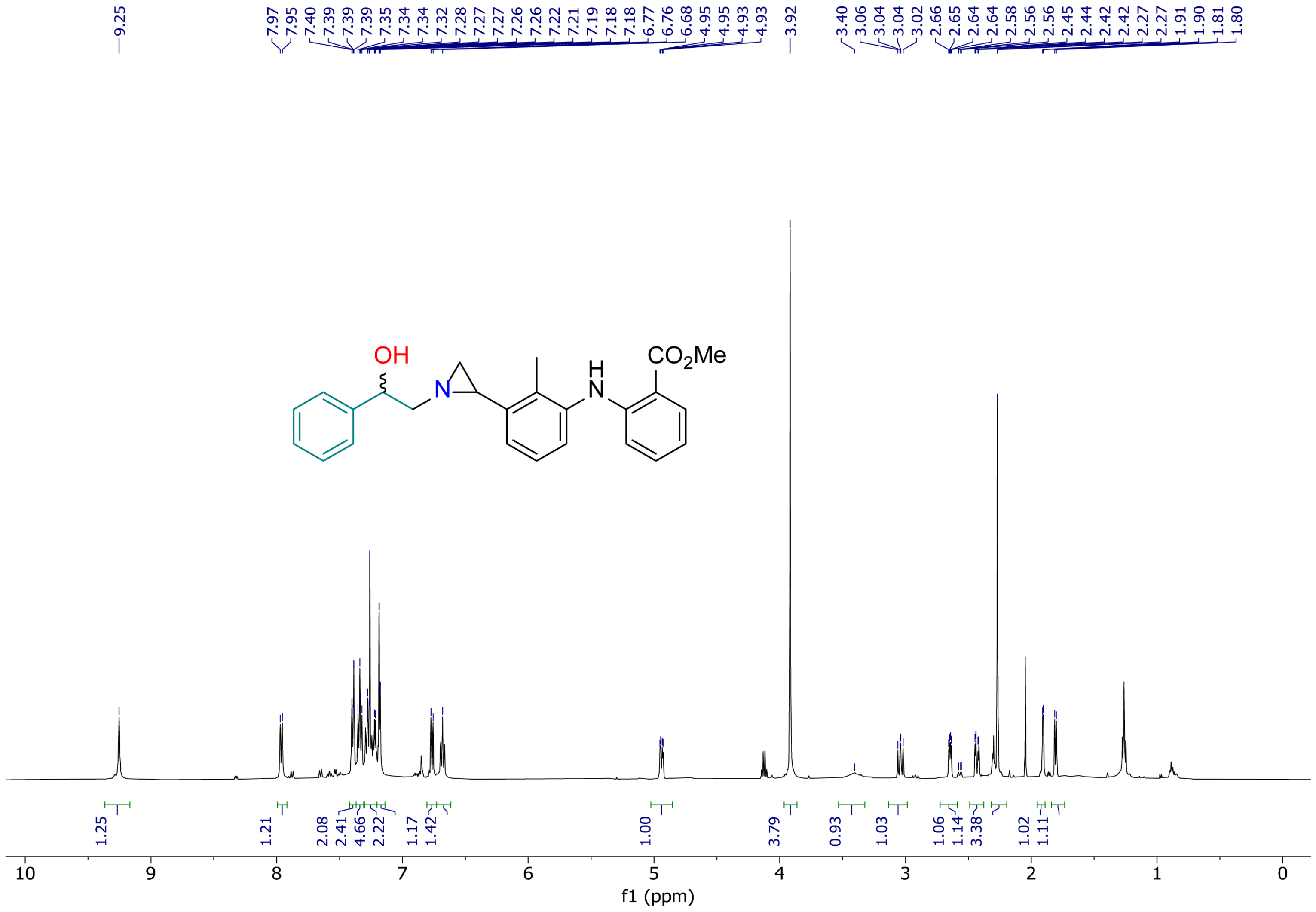

Figure S110. ^{1}H NMR spectrum of methyl 2-((3-(1-(2-hydroxy-2-phenylethyl)aziridin-2-yl)-2-methylphenyl)amino)benzoate (**5ab**)in $CDCl_3$ (400 MHz) at 23 °C.

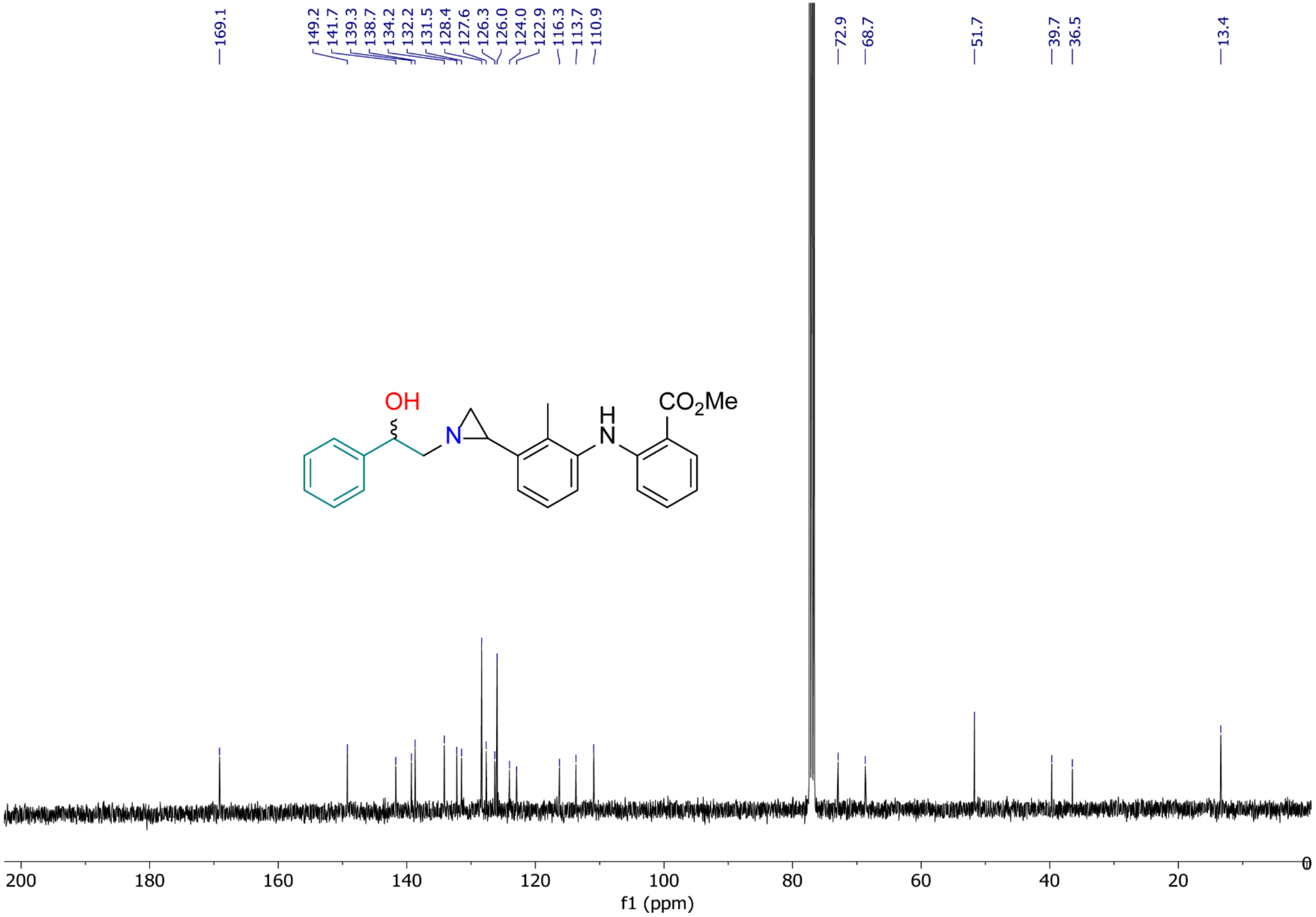

Figure S111. ^{13}C NMR spectrum of methyl 2-((3-(1-(2-hydroxy-2-phenylethyl)aziridin-2-yl)-2-methylphenyl)amino)benzoate (**5ab**) in $CDCl_3$ (126 MHz) at 23 °C

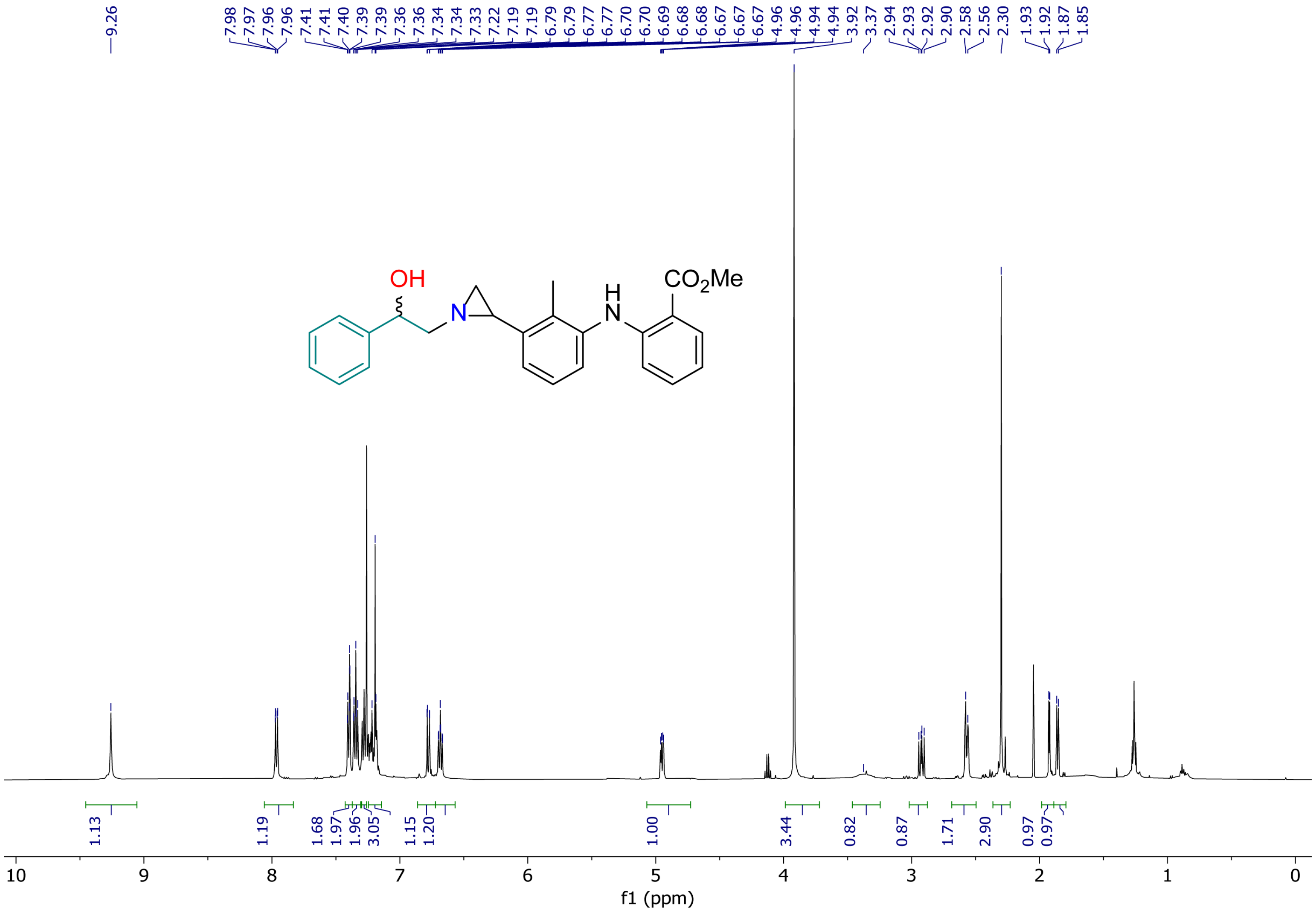

Figure S112. ^{1}H NMR spectrum of methyl 2-((3-(1-(2-hydroxy-2-phenylethyl)aziridin-2-yl)-2-methylphenyl)amino)benzoate (**5ab**)in $CDCl_3$ (400 MHz) at 23 °C.

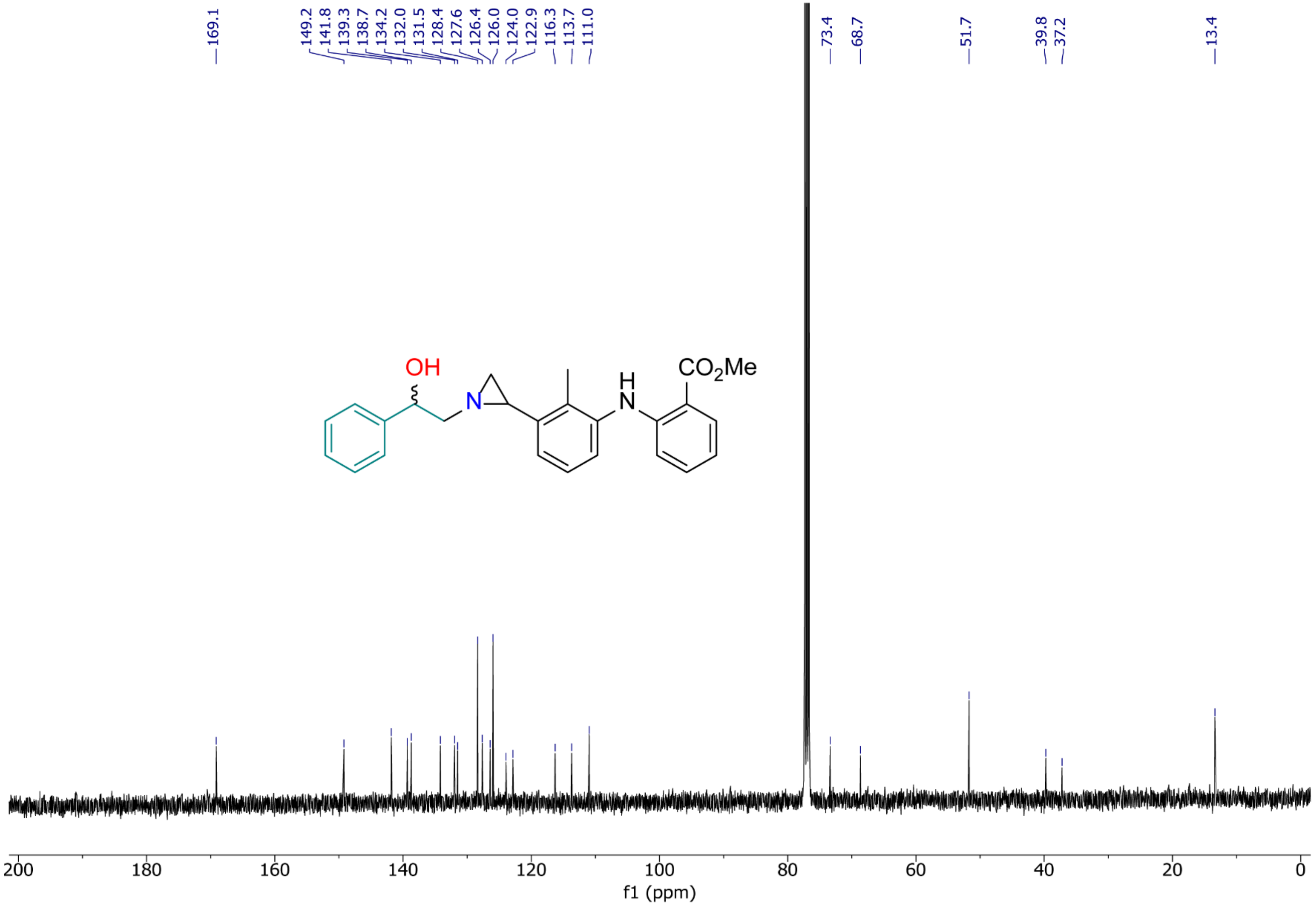

Figure S113. ^{13}C NMR spectrum of methyl 2-((3-(1-(2-hydroxy-2-phenylethyl)aziridin-2-yl)-2-methylphenyl)amino)benzoate (**5ab**) in $CDCl_3$ (126 MHz) at 23 °C

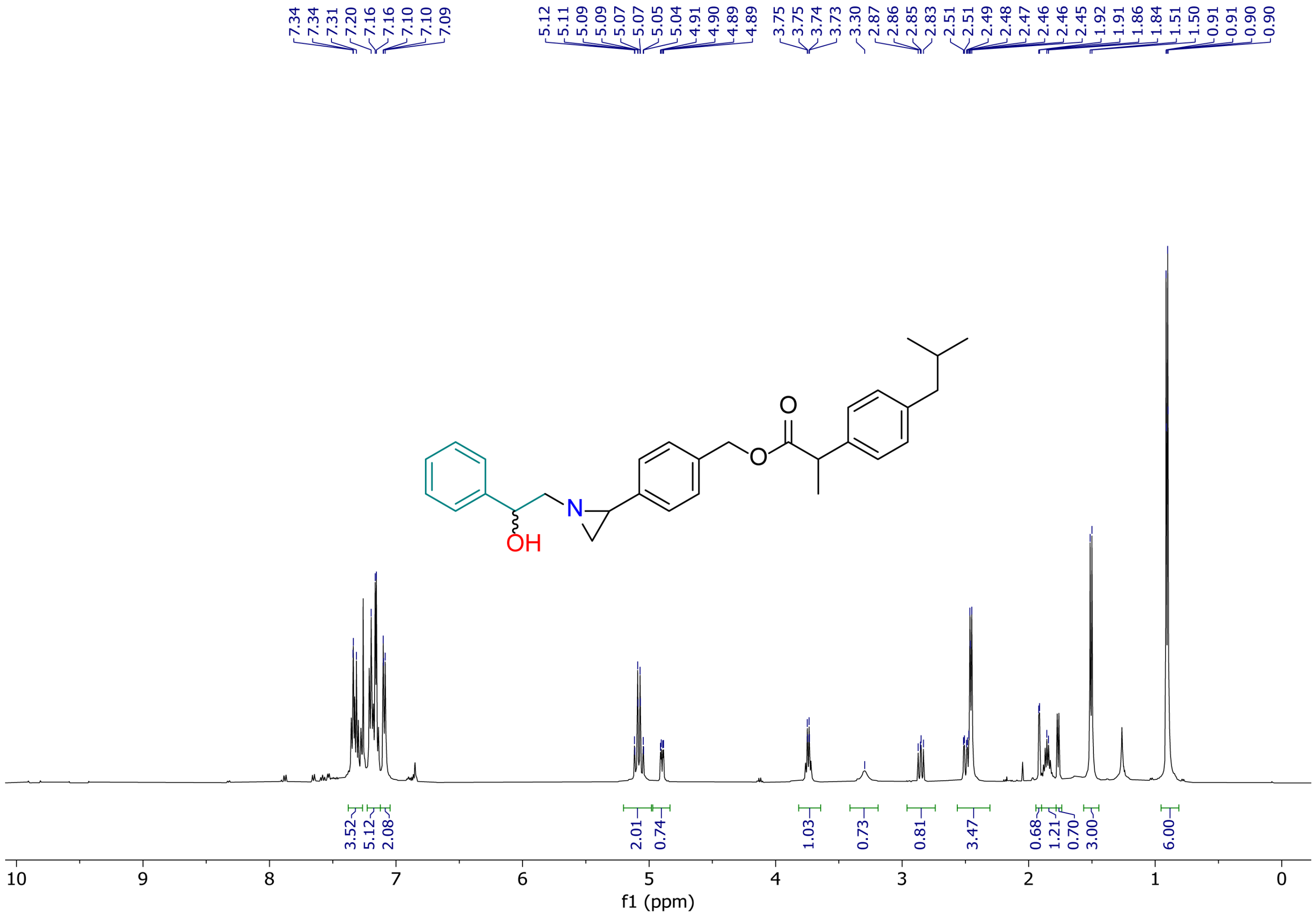

Figure S114. ^{1}H NMR spectrum of 4-(1-(2-hydroxy-2-phenylethyl)aziridin-2-yl)benzyl-2-(4-isobutylphenyl)propanoate (**5ac**) in $CDCl_3$ (400 MHz) at 23 °C.

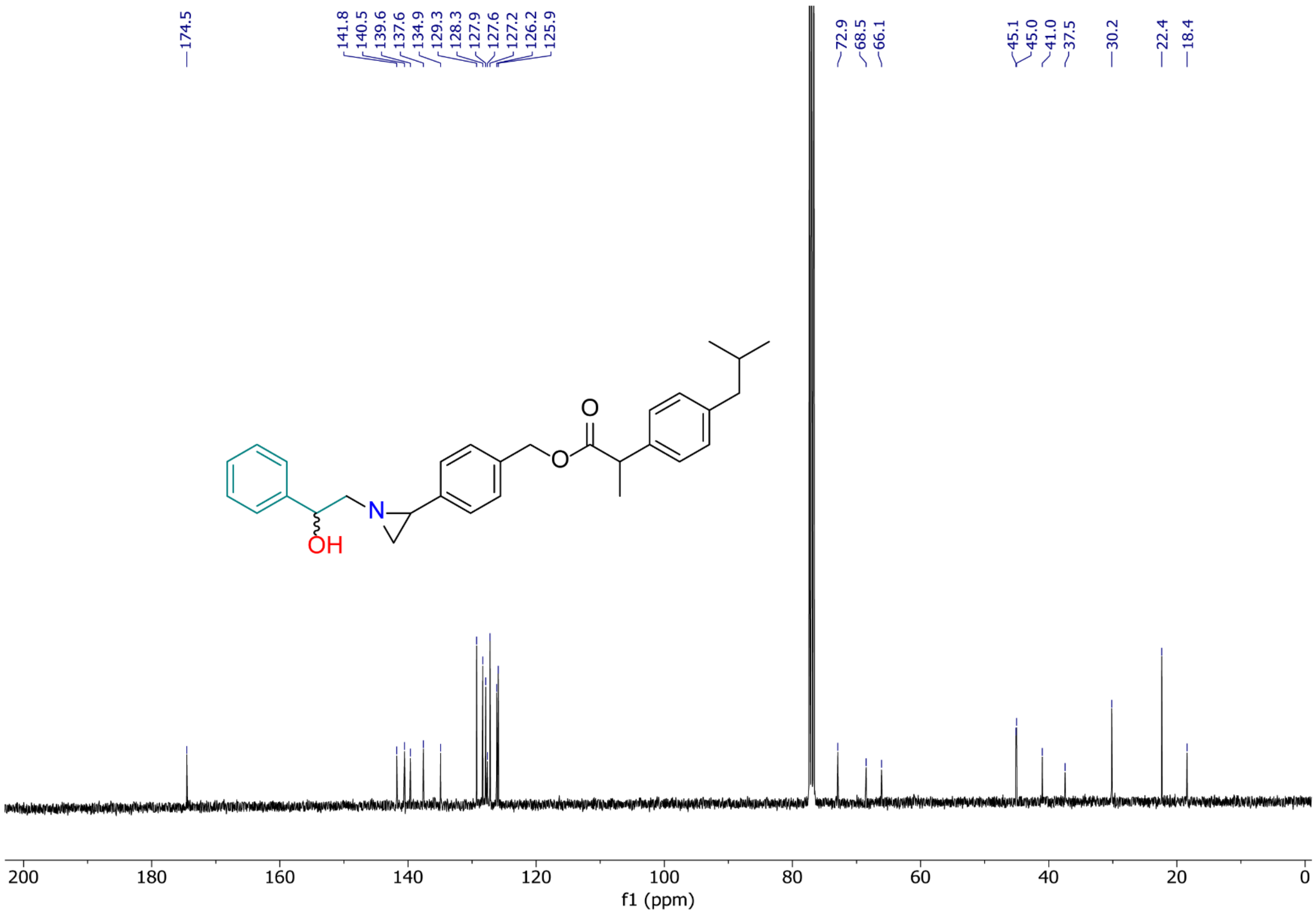

Figure S115. ^{13}C NMR spectrum of 4-(1-(2-hydroxy-2-phenylethyl)aziridin-2-yl)benzyl-2-(4-isobutylphenyl)propanoate (**5ac**) in $CDCl_3$ (101 MHz) at 23 °C.

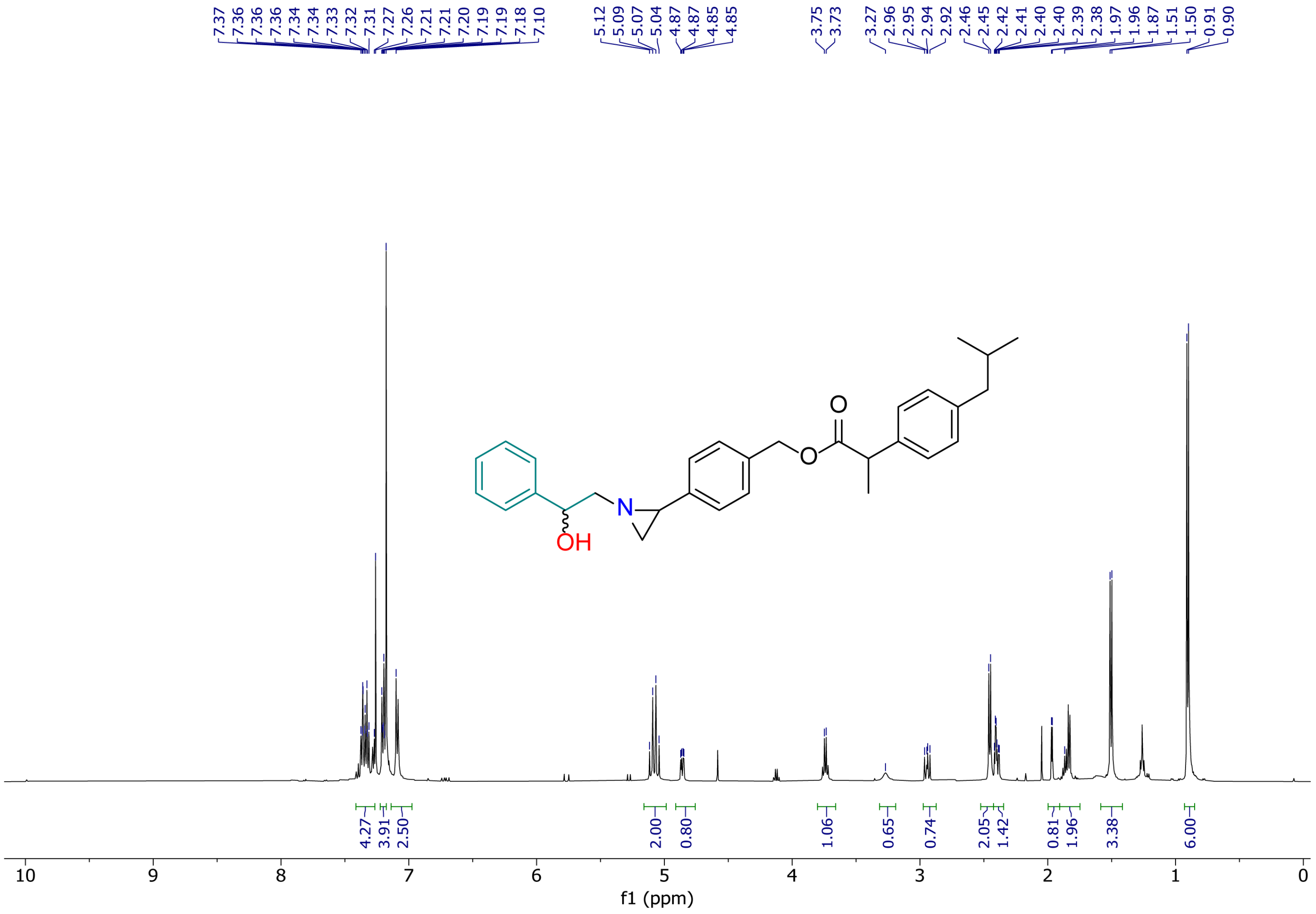

Figure S116. ^{1}H NMR spectrum of 4-(1-(2-hydroxy-2-phenylethyl)aziridin-2-yl)benzyl-2-(4-isobutylphenyl)propanoate (**5ac**) in $CDCl_3$ (400 MHz) at 23 °C.

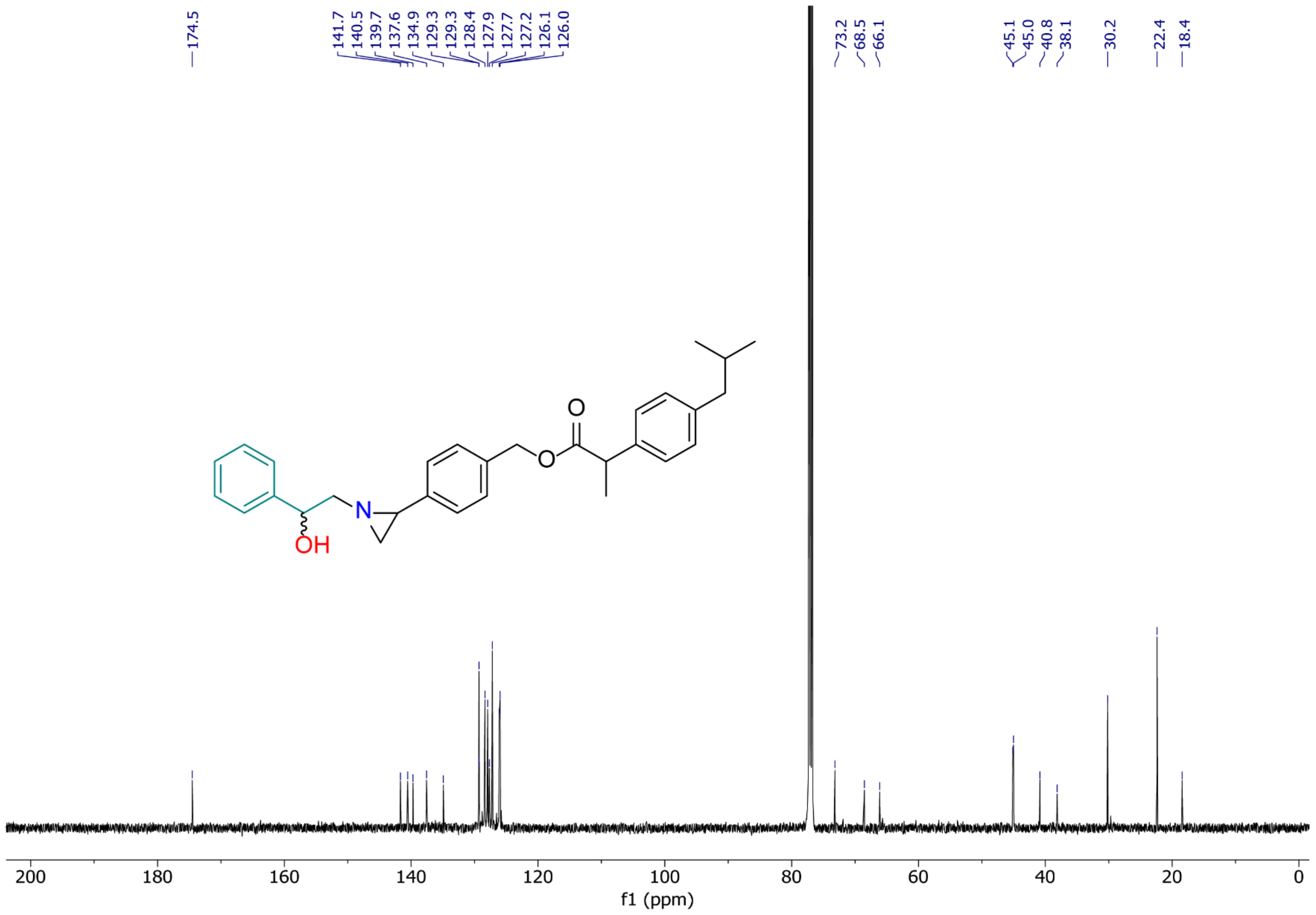

Figure S117. ^{13}C NMR spectrum of 4-(1-(2-hydroxy-2-phenylethyl)aziridin-2-yl)benzyl-2-(4-isobutylphenyl)propanoate (**5ac**) in $CDCl_3$ (101 MHz) at 23 °C

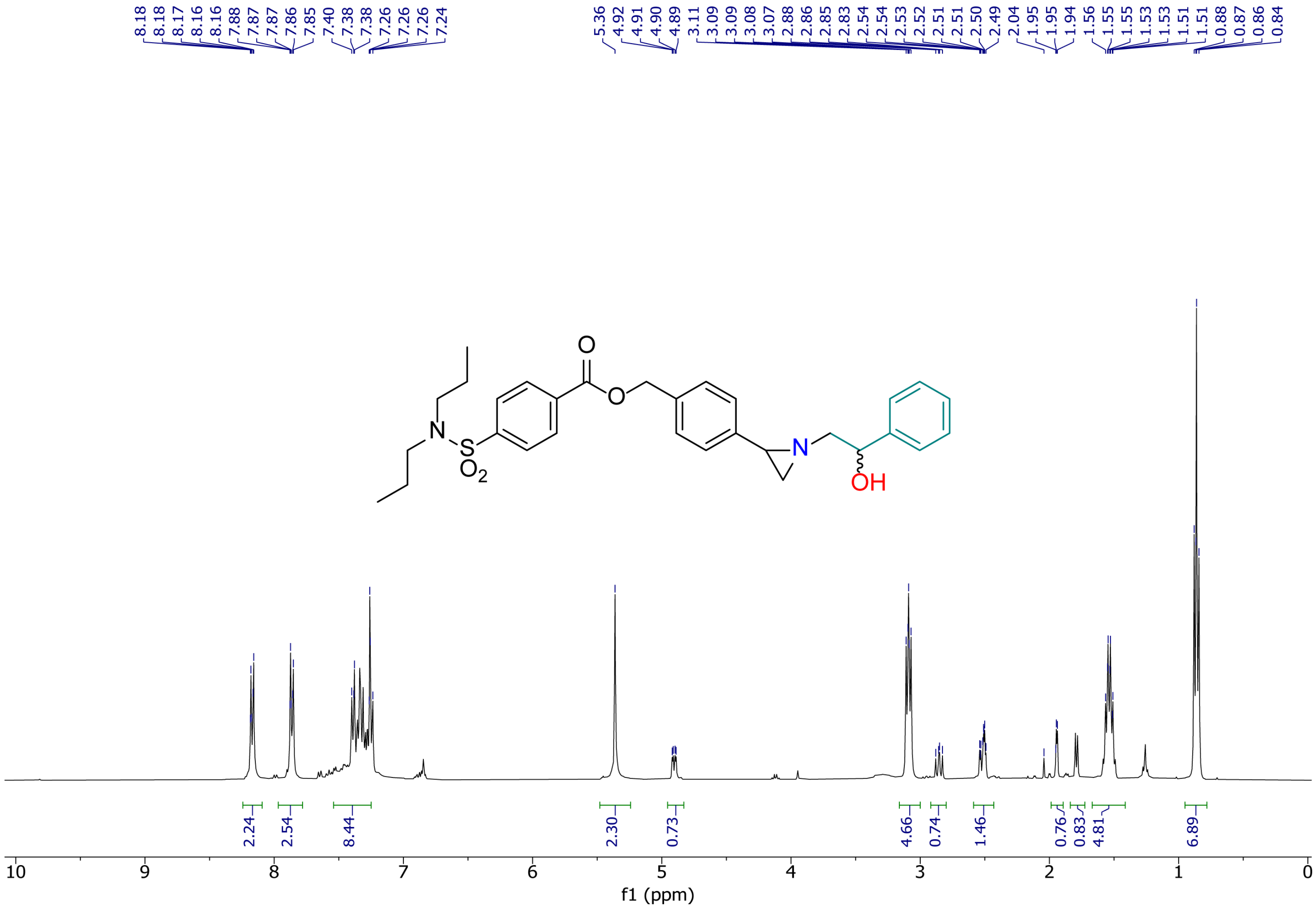

Figure S118. ^{1}H NMR spectrum of 4-(1-(2-hydroxy-2-phenylethyl)aziridin-2-yl)benzyl-2-(4-isobutylphenyl)propanoate (**5ad**) in $CDCl_3$ (400 MHz) at 23 °C.

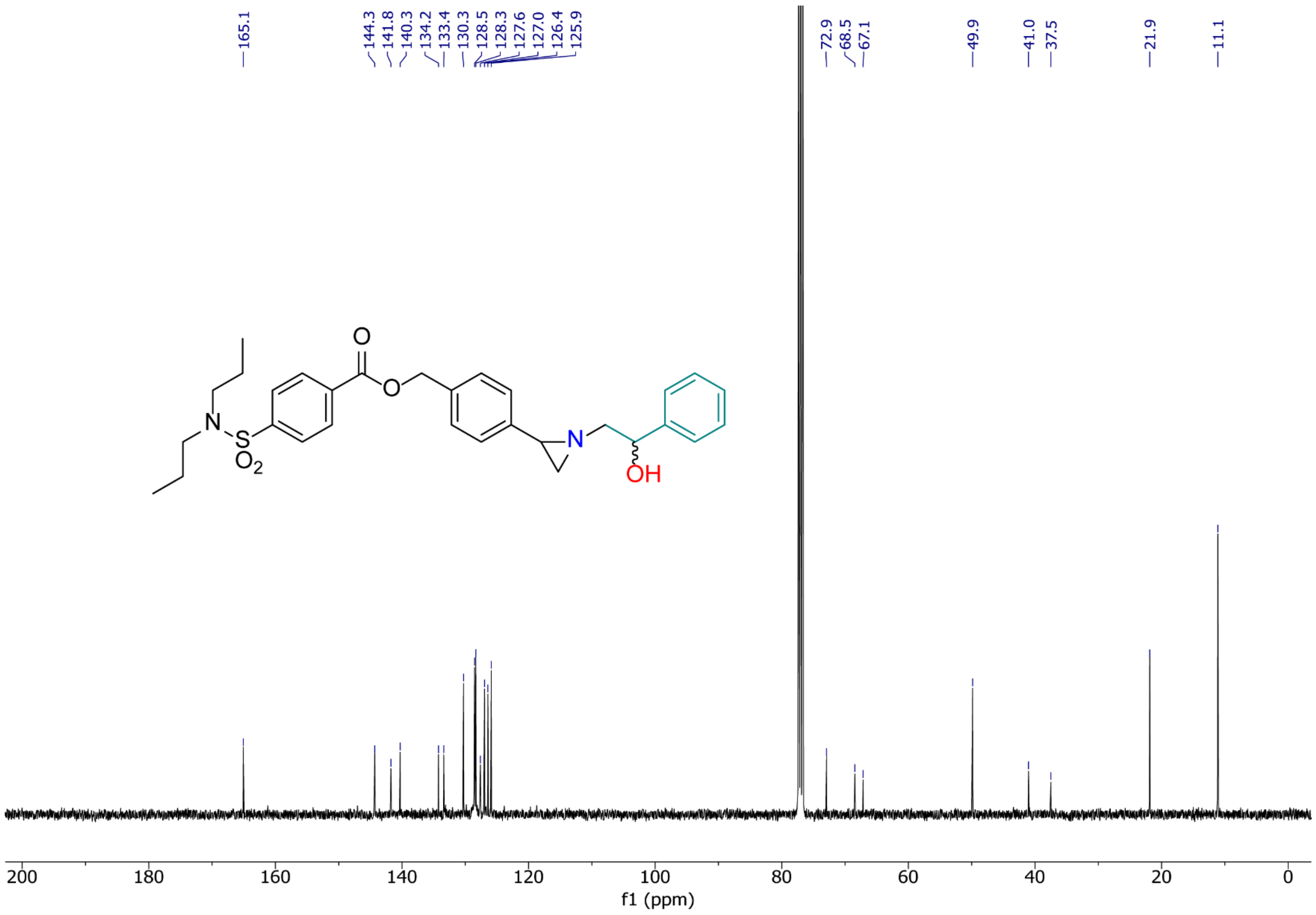

Figure S119. ^{13}C NMR spectrum of 4-(1-(2-hydroxy-2-phenylethyl)aziridin-2-yl)benzyl-2-(4-isobutylphenyl)propanoate (**5ad**) in $CDCl_3$ (101 MHz) at 23 °C.

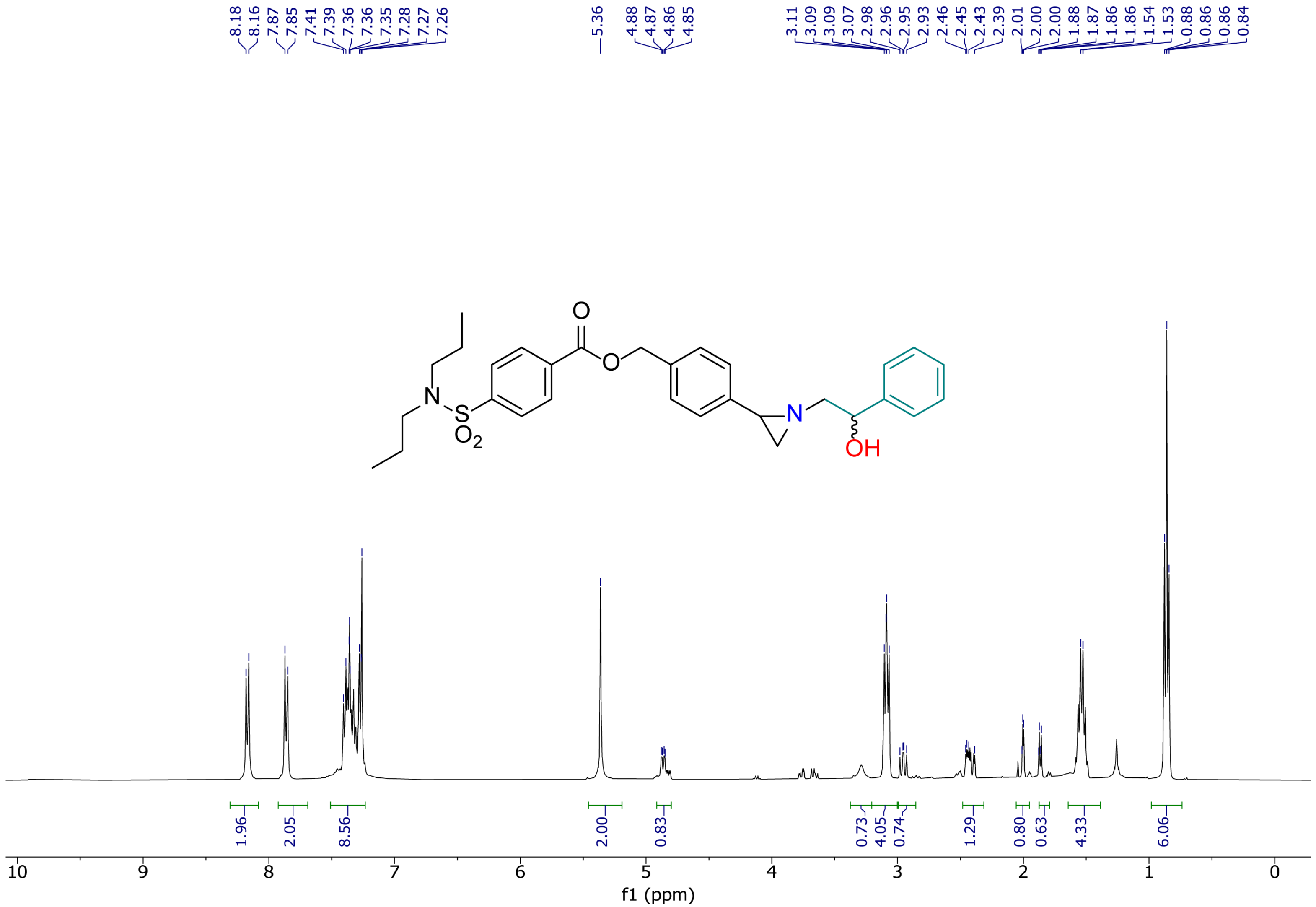

Figure S120. ^{1}H NMR spectrum of 4-(1-(2-hydroxy-2-phenylethyl)aziridin-2-yl)benzyl-2-(4-isobutylphenyl)propanoate (**5ad**) in $CDCl_3$ (400 MHz) at 23 °C.

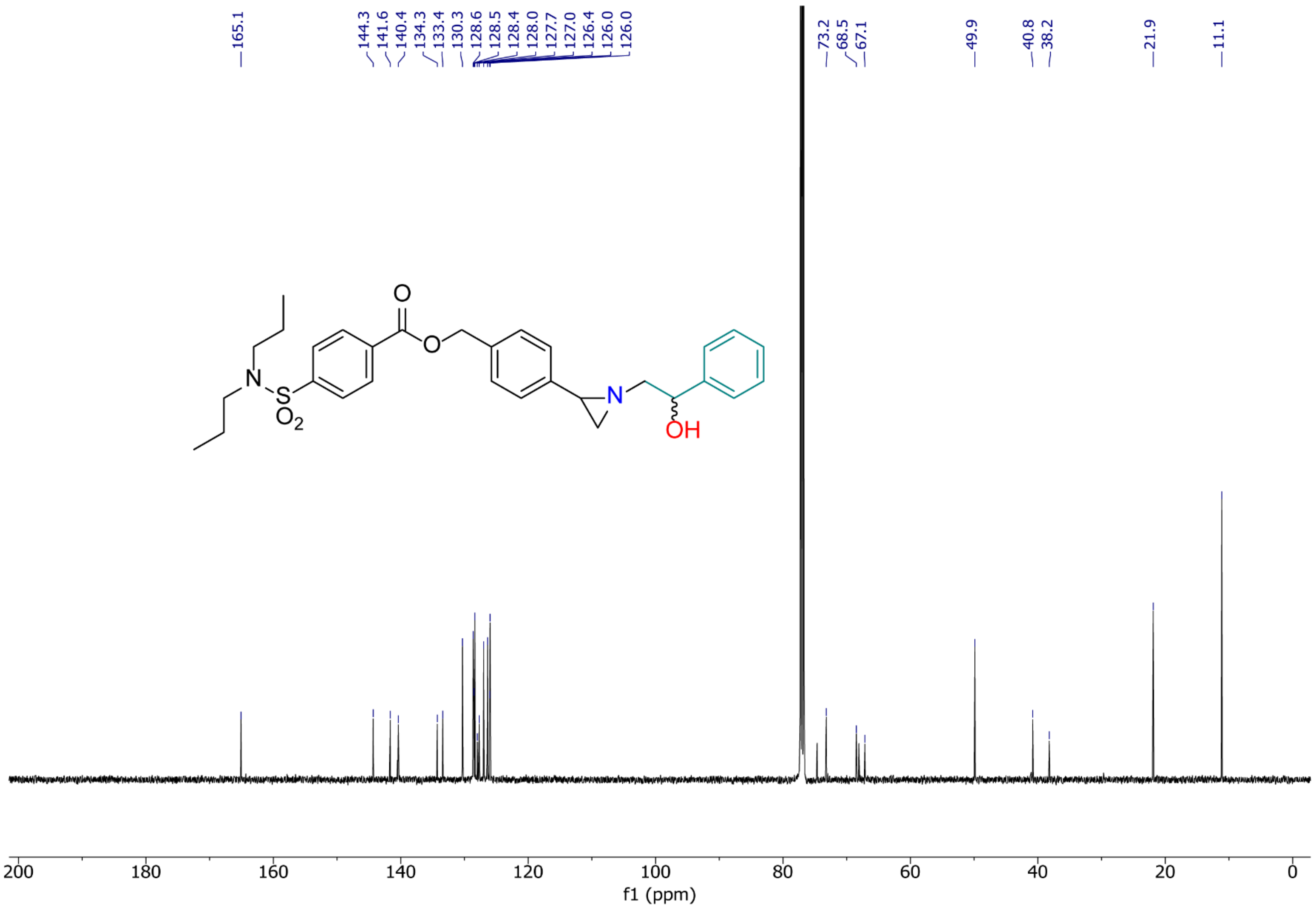

Figure S121. ^{13}C NMR spectrum of 4-(1-(2-hydroxy-2-phenylethyl)aziridin-2-yl)benzyl-2-(4-isobutylphenyl)propanoate (**5ad**) in $CDCl_3$ (101 MHz) at 23 °C.

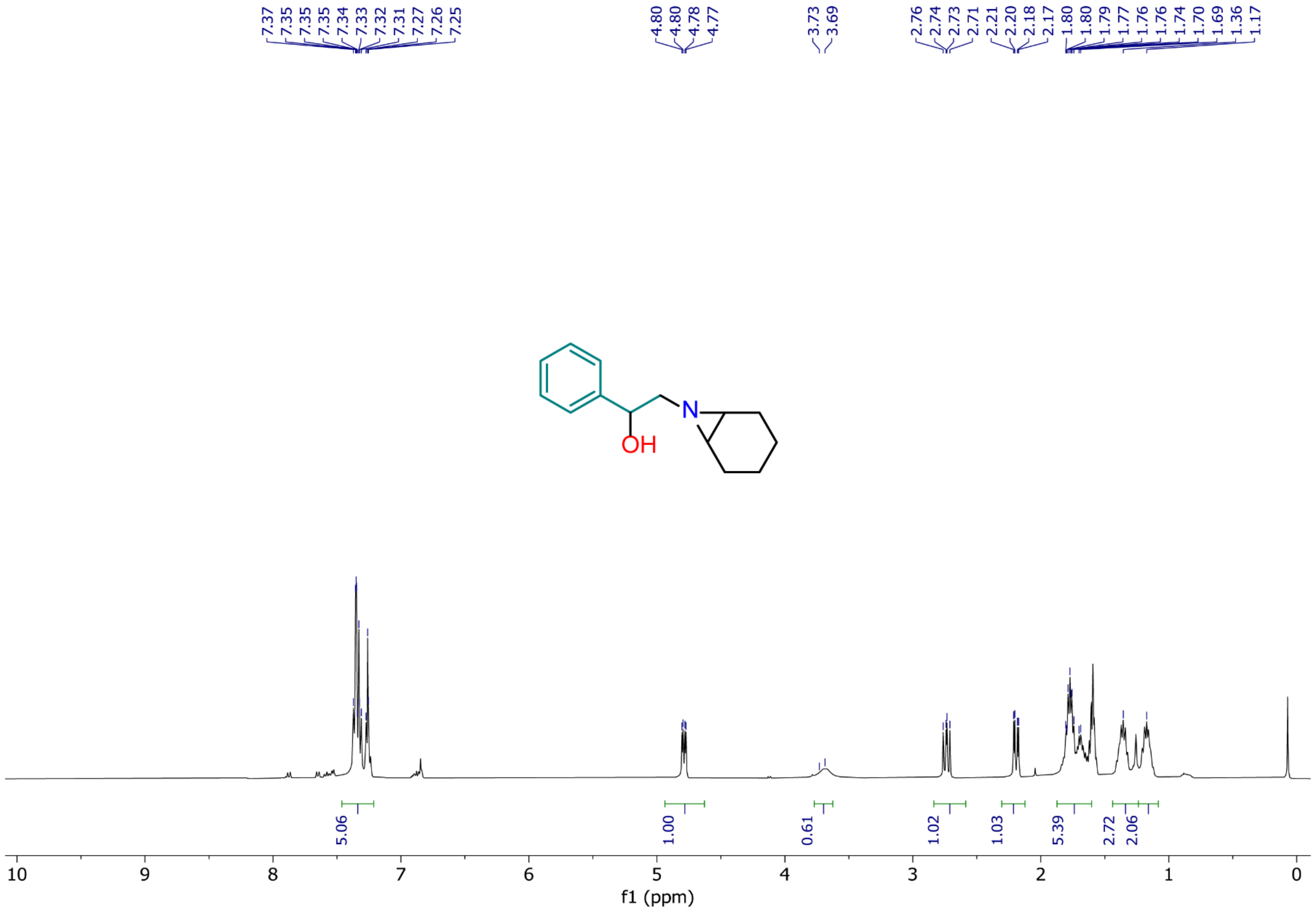

Figure S122. ^{1}H NMR spectrum of 2-(7-azabicyclo[4.1.0]heptan-7-yl)-1-phenylethan-1-ol (**7a**) in $CDCl_3$ (400 MHz) at 23 °C.

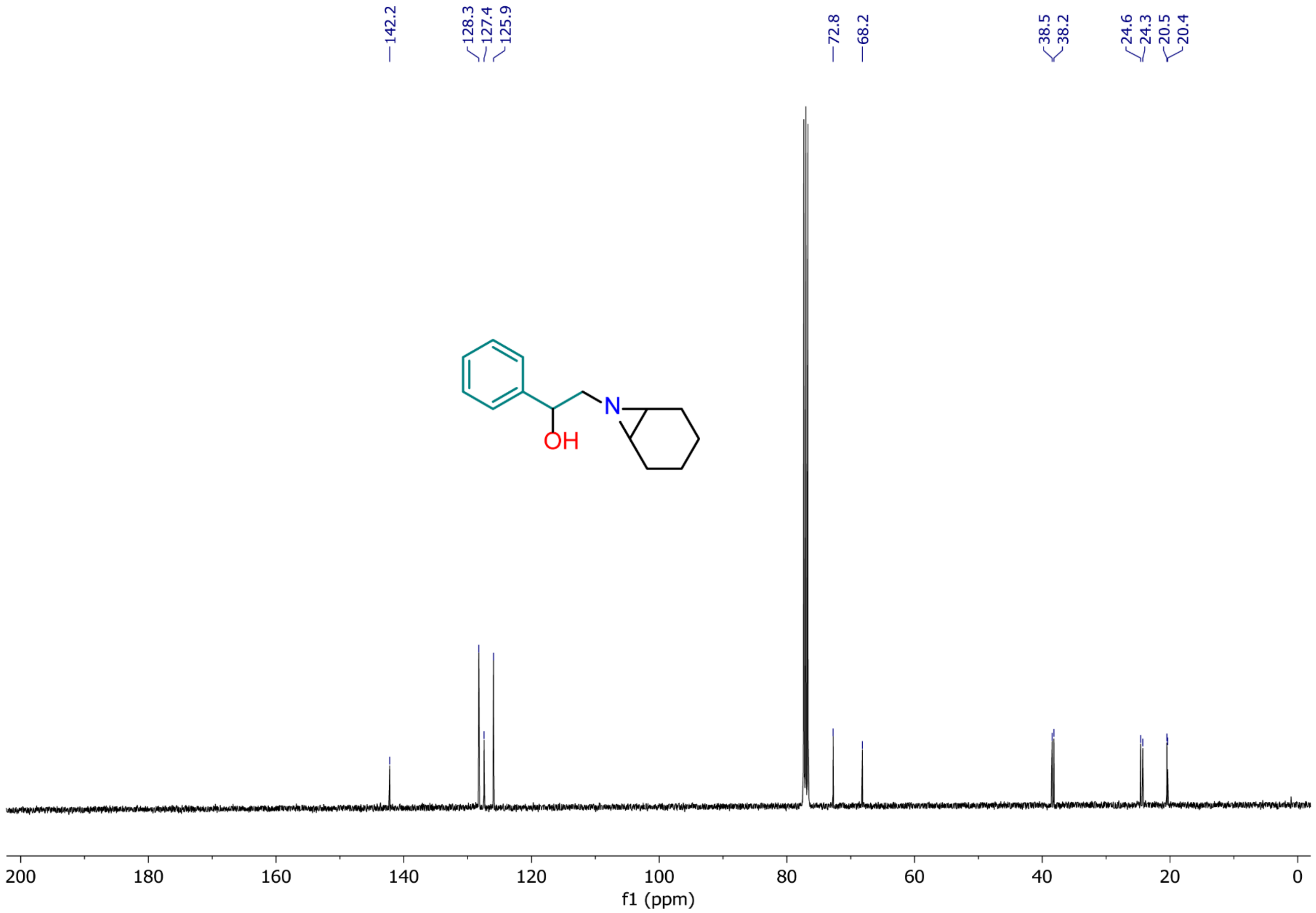

Figure S123. ^{13}C NMR spectrum of 2-(7-azabicyclo[4.1.0]heptan-7-yl)-1-phenylethan-1-ol (**7a**) in $CDCl_3$ (101 MHz) at 23 °C.

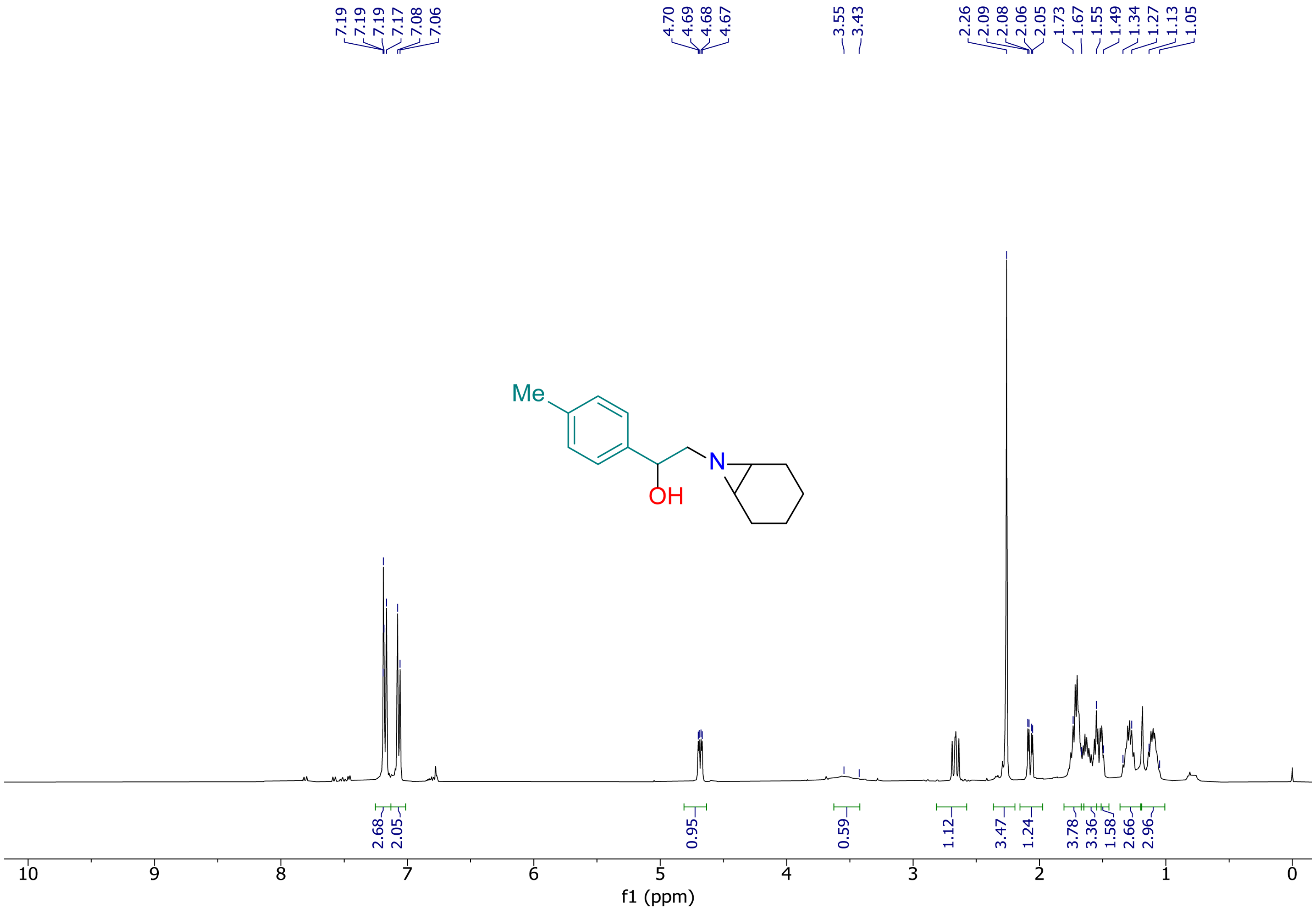

Figure S124. ^{1}H NMR spectrum of 2-(7-azabicyclo[4.1.0]heptan-7-yl)-1-(p-tolyl)ethan-1-ol (**7b**) in $CDCl_3$ (400 MHz) at 23 °C.

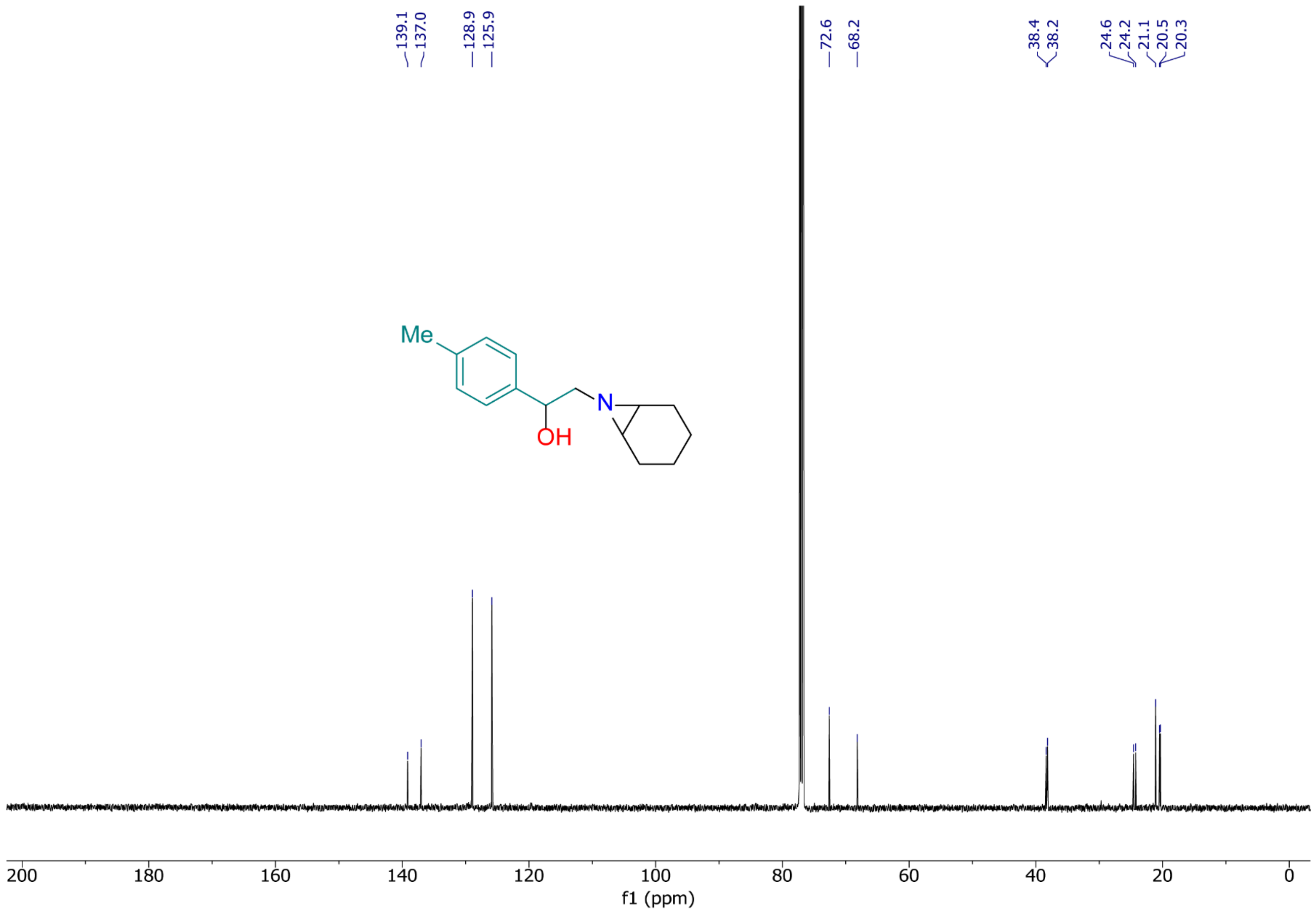

Figure S125. ^{13}C NMR spectrum of 2-(7-azabicyclo[4.1.0]heptan-7-yl)-1-(p-tolyl)ethan-1-ol (**7b**) in $CDCl_3$ (101 MHz) at 23 °C.

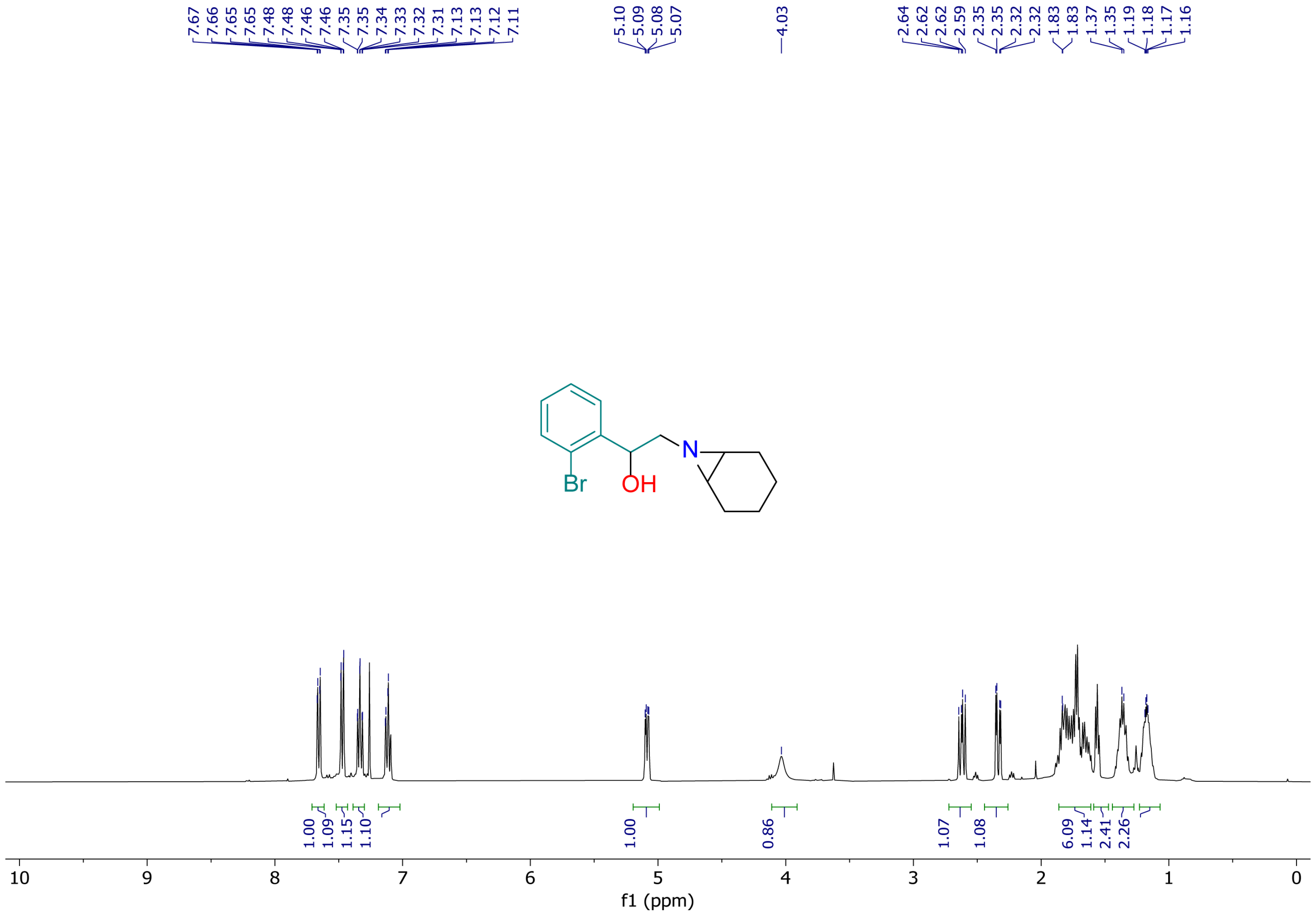

Figure S126. ^{1}H NMR spectrum of 2-(7-azabicyclo[4.1.0]heptan-7-yl)-1-(2-bromophenyl)ethan-1-ol (**7c**) in $CDCl_3$ (400 MHz) at 23 °C.

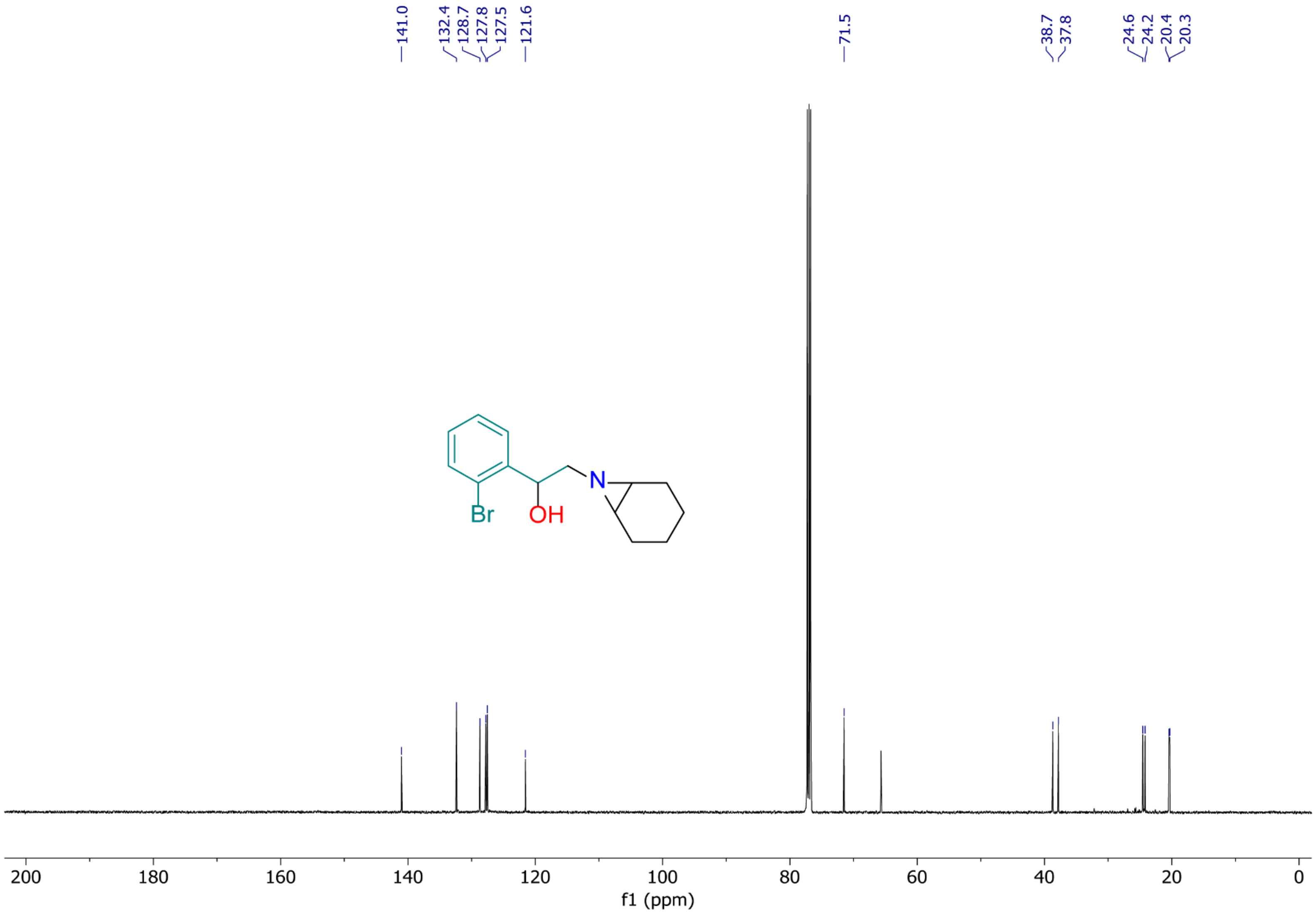

Figure S127. ^{13}C NMR spectrum of 2-(7-azabicyclo[4.1.0]heptan-7-yl)-1-(2-bromophenyl)ethan-1-ol (**7c**) in $CDCl_3$ (101 MHz) at 23 °C.

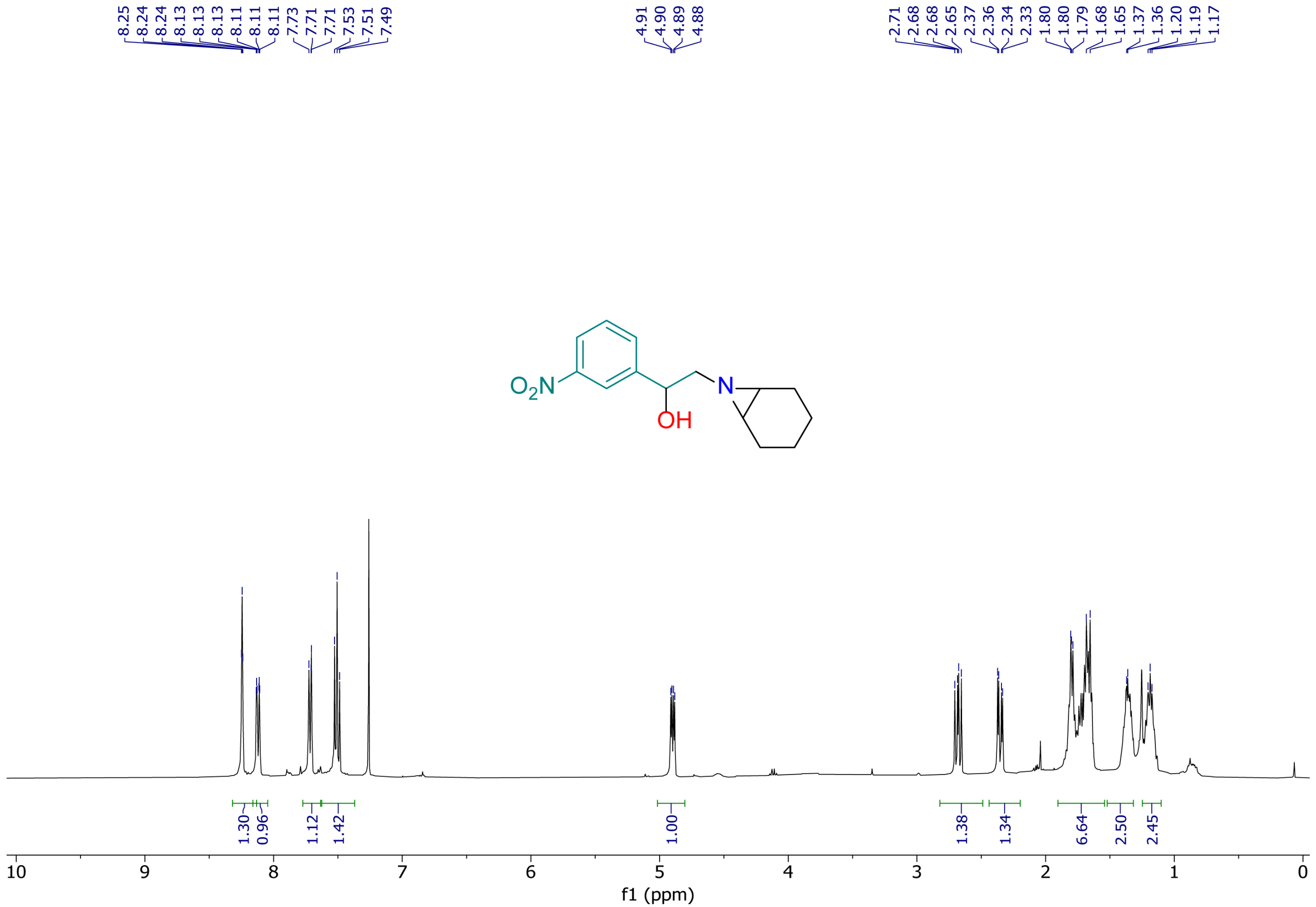

Figure S128. ^{1}H NMR spectrum of 2-(7-azabicyclo[4.1.0]heptan-7-yl)-1-(3-nitrophenyl)ethan-1-ol (**7d**) in $CDCl_3$ (400 MHz) at 23 °C.

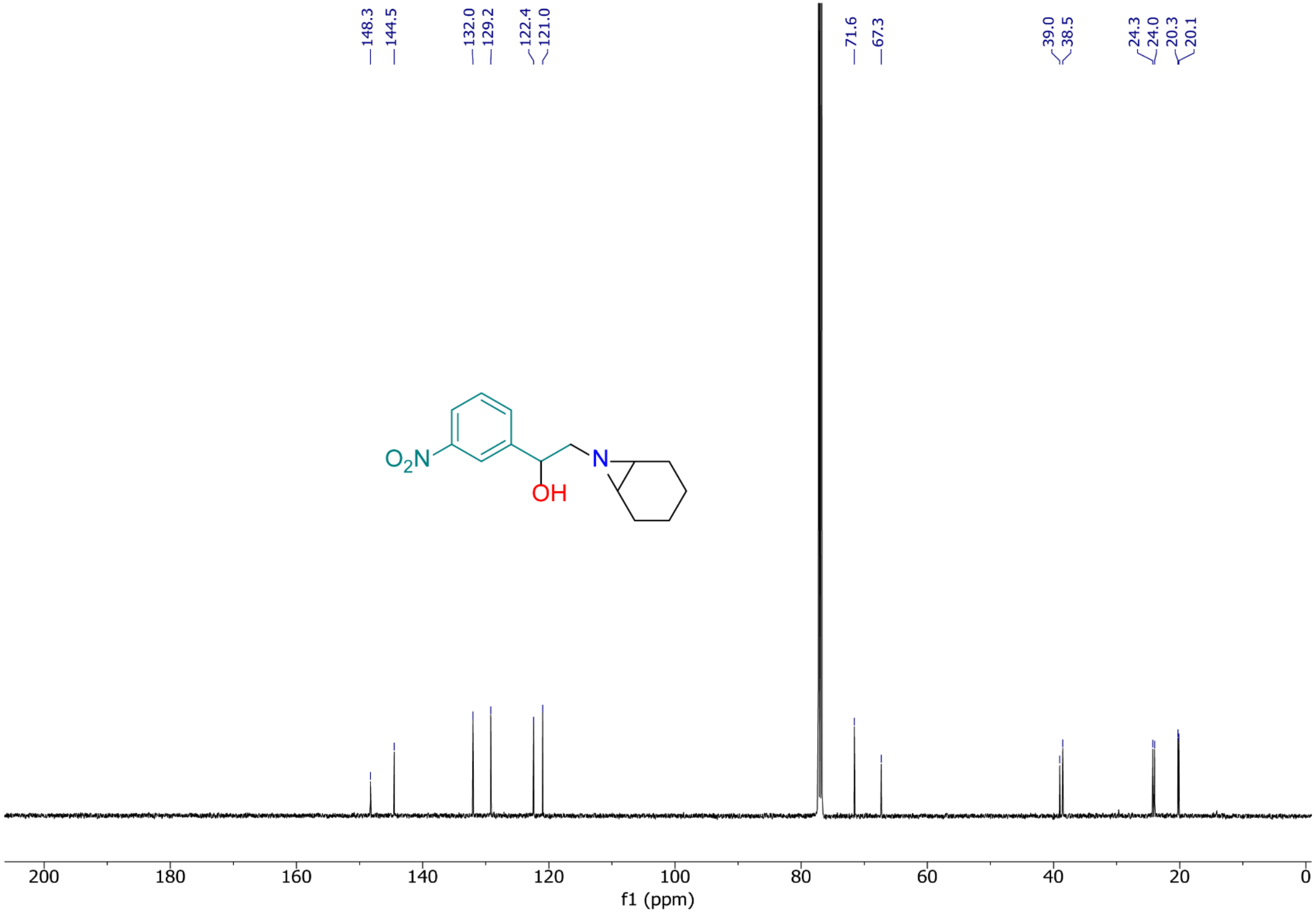

Figure S129. ^{13}C NMR spectrum of 2-(7-azabicyclo[4.1.0]heptan-7-yl)-1-(3-nitrophenyl)ethan-1-ol (**7d**) in $CDCl_3$ (101 MHz) at 23 °C.

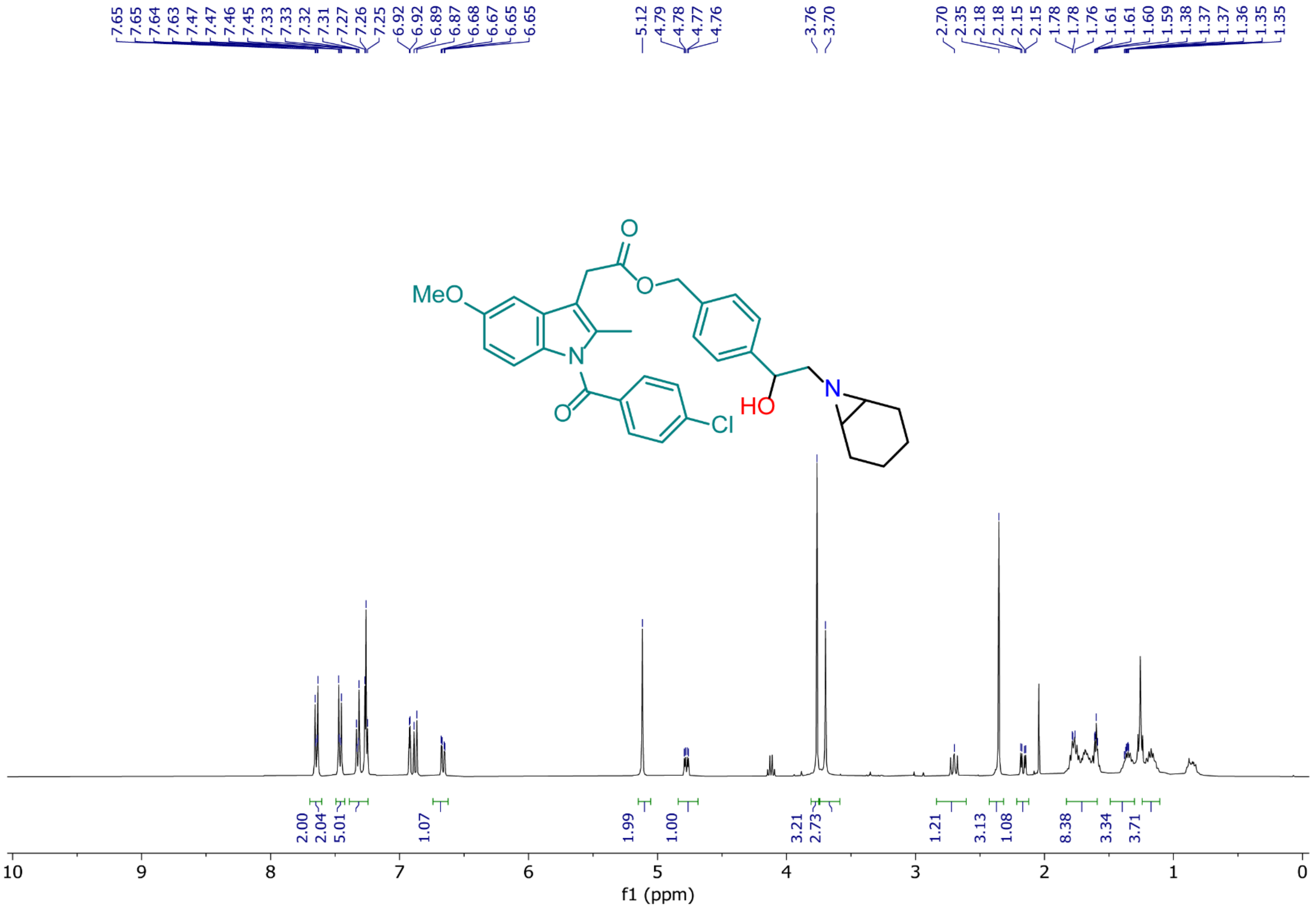

Figure S130. ^{1}H NMR spectrum of 4-((1S)-2-(7-azabicyclo[4.1.0]heptan-7-yl)-1-hydroxyethyl)benzyl 2-(1-(4-chlorobenzoyl)-5-methoxy-2-methyl-1H-indol-3-yl)acetate (**7e**) in $CDCl_3$ (400 MHz) at 23 °C.

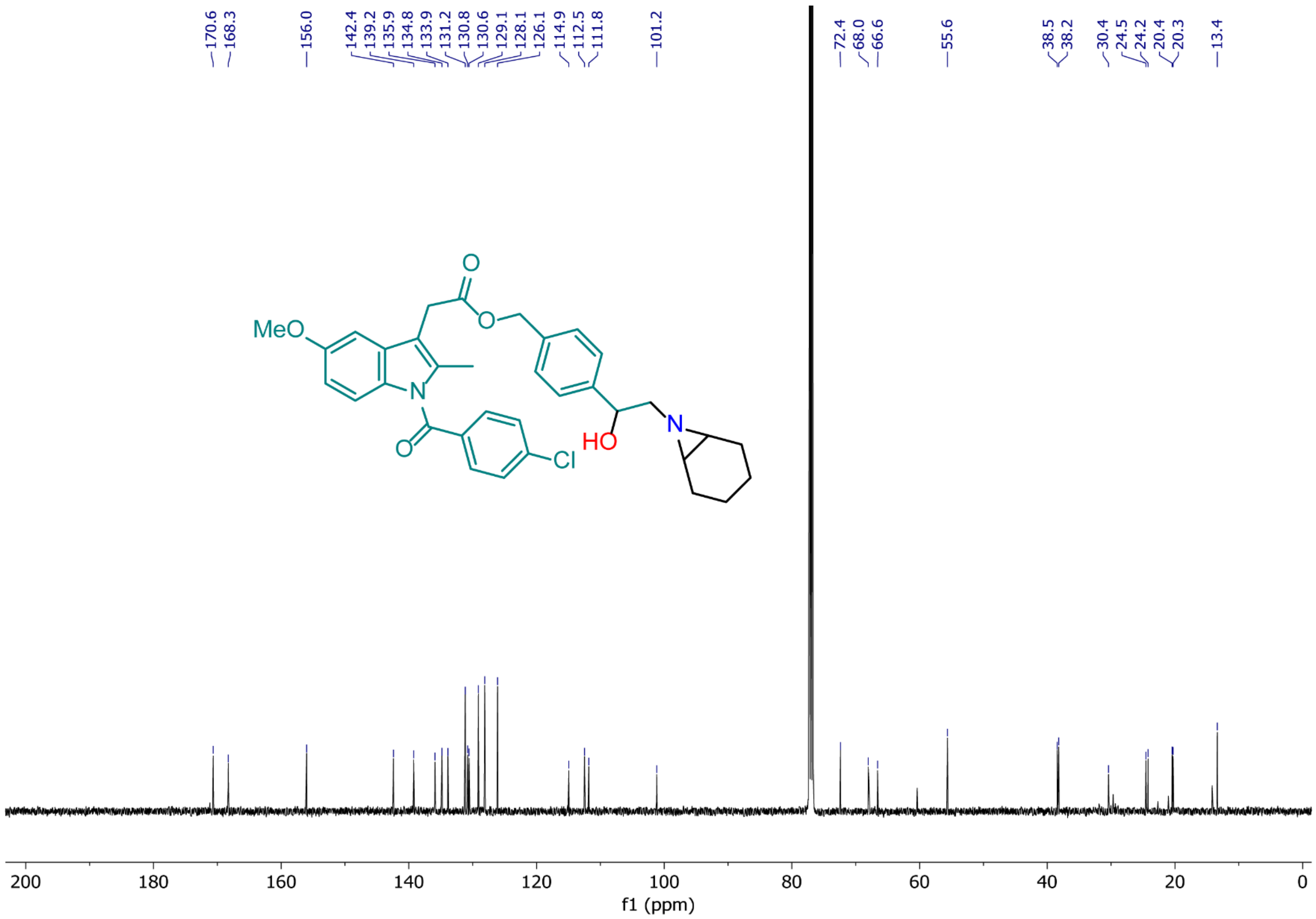

Figure S131. ^{13}C NMR spectrum of 4-((1S)-2-(7-azabicyclo[4.1.0]heptan-7-yl)-1-hydroxyethyl)benzyl 2-(1-(4-chlorobenzoyl)-5-methoxy-2-methyl-1H-indol-3-yl)acetate (**7e**) in $CDCl_3$ (126 MHz) at 23 °C.

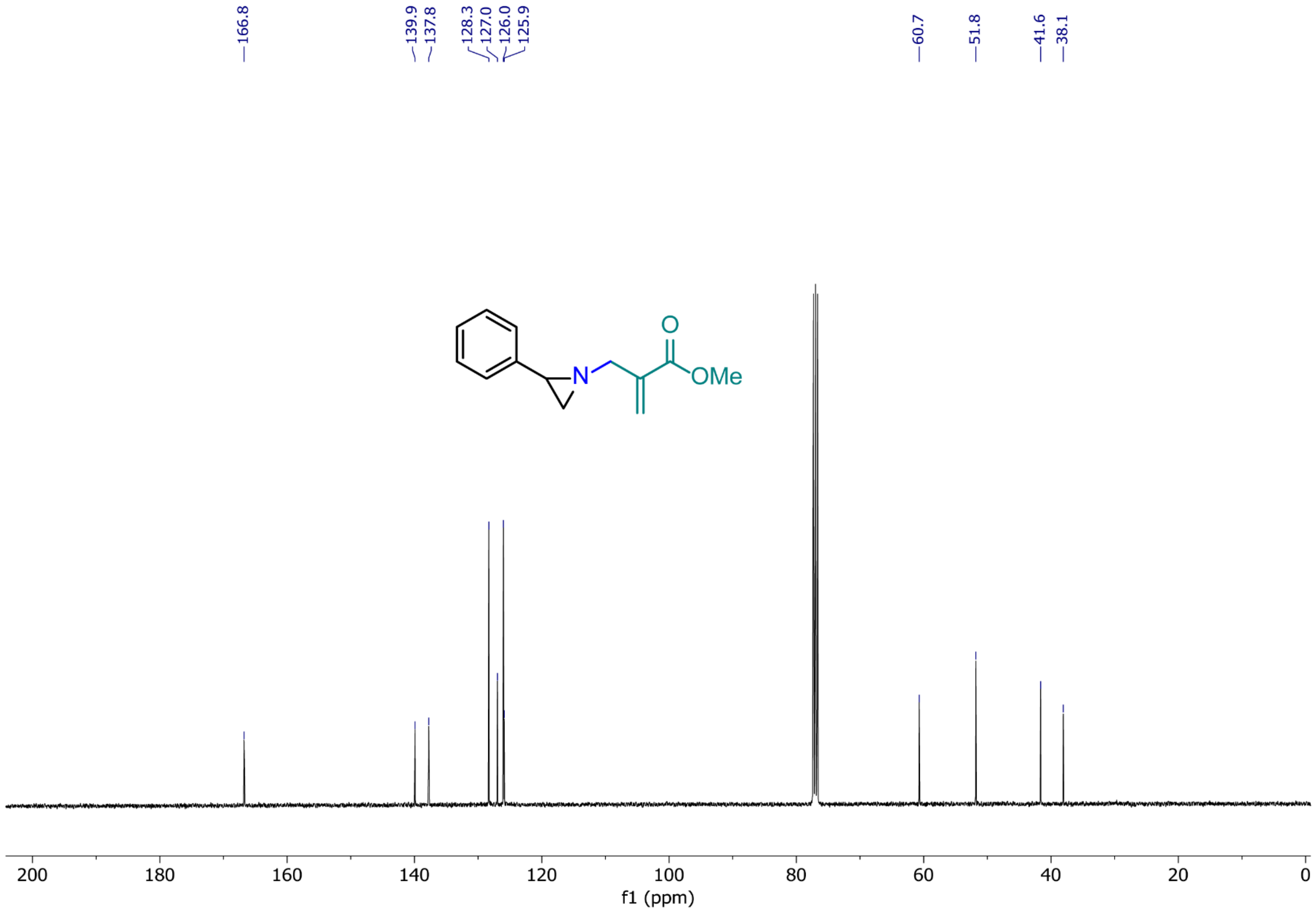

Figure S132. ^{13}C NMR spectrum of methyl 2-((2-phenylaziridin-1-yl)methyl)acrylate (**3**) in $CDCl_3$ (101 MHz) at 23 °C.

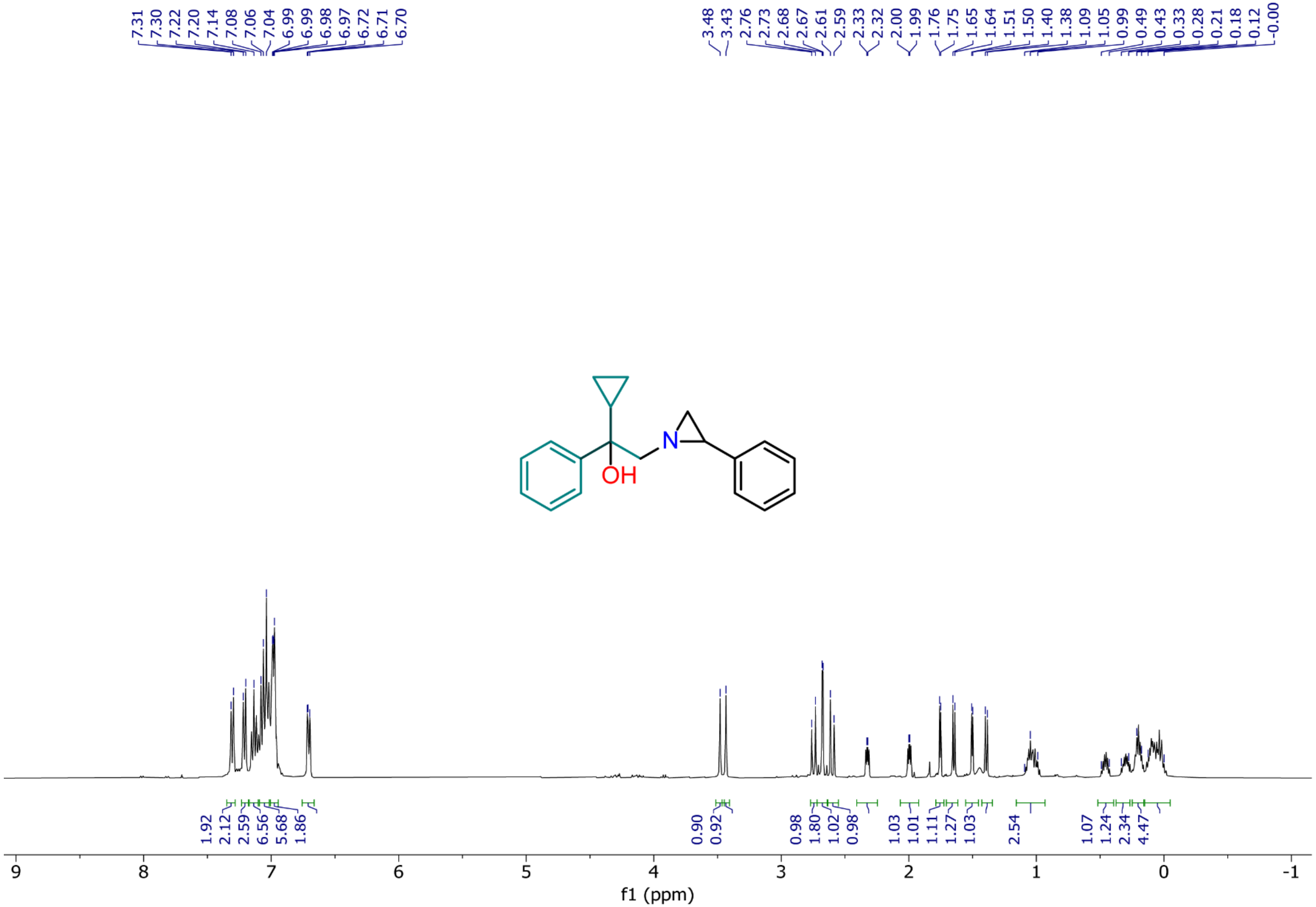

Figure S133. ^{1}H NMR spectrum of 1-cyclopropyl-1-phenyl-2-(2-phenylaziridin-1-yl)ethan-1-ol (**10**) in $CDCl_3$ (101 MHz) at 23 °C.

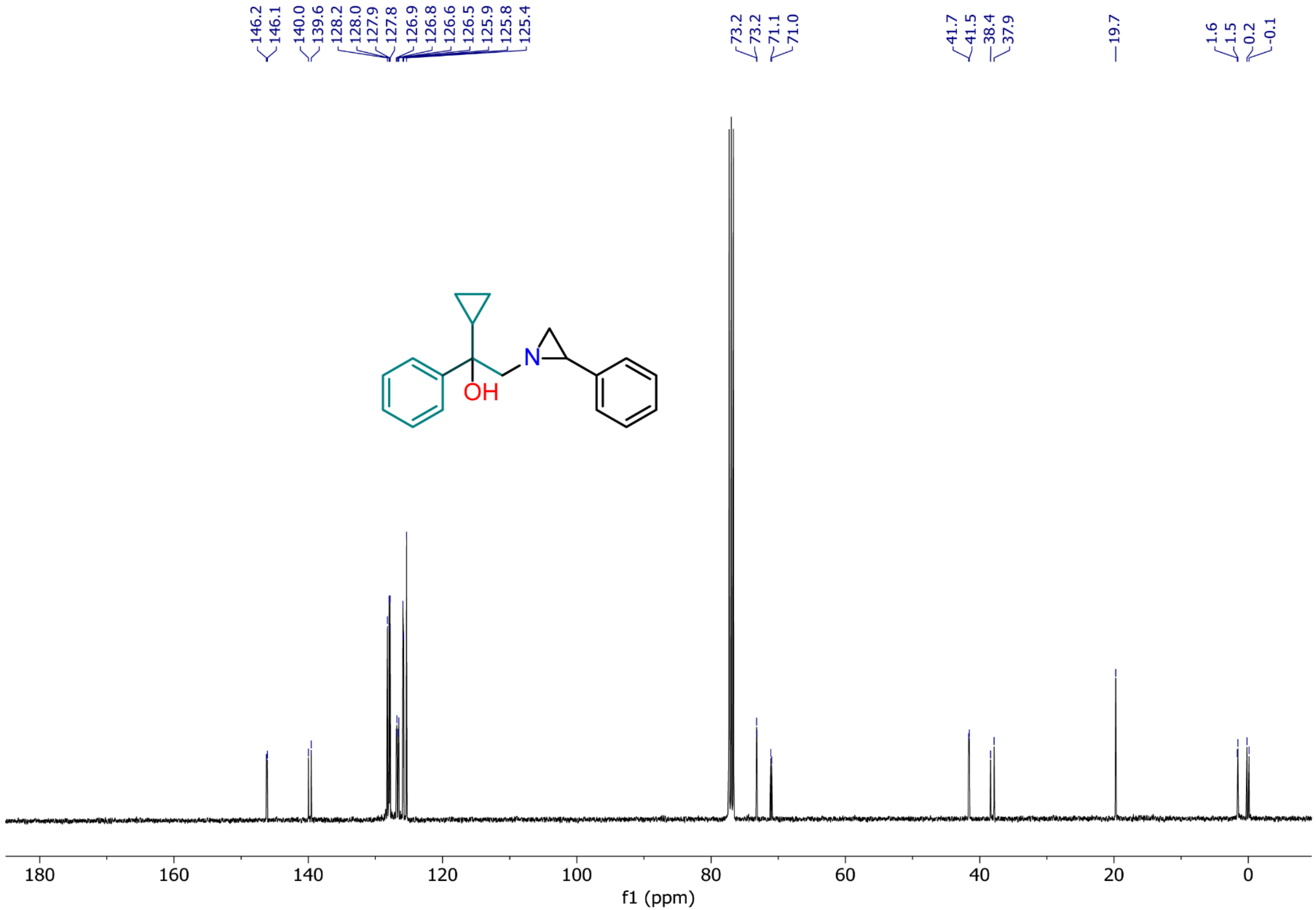

Figure S134. ^{13}C NMR spectrum of 1-cyclopropyl-1-phenyl-2-(2-phenylaziridin-1-yl)ethan-1-ol (**10**) in $CDCl_3$ (101 MHz) at 23 °C.

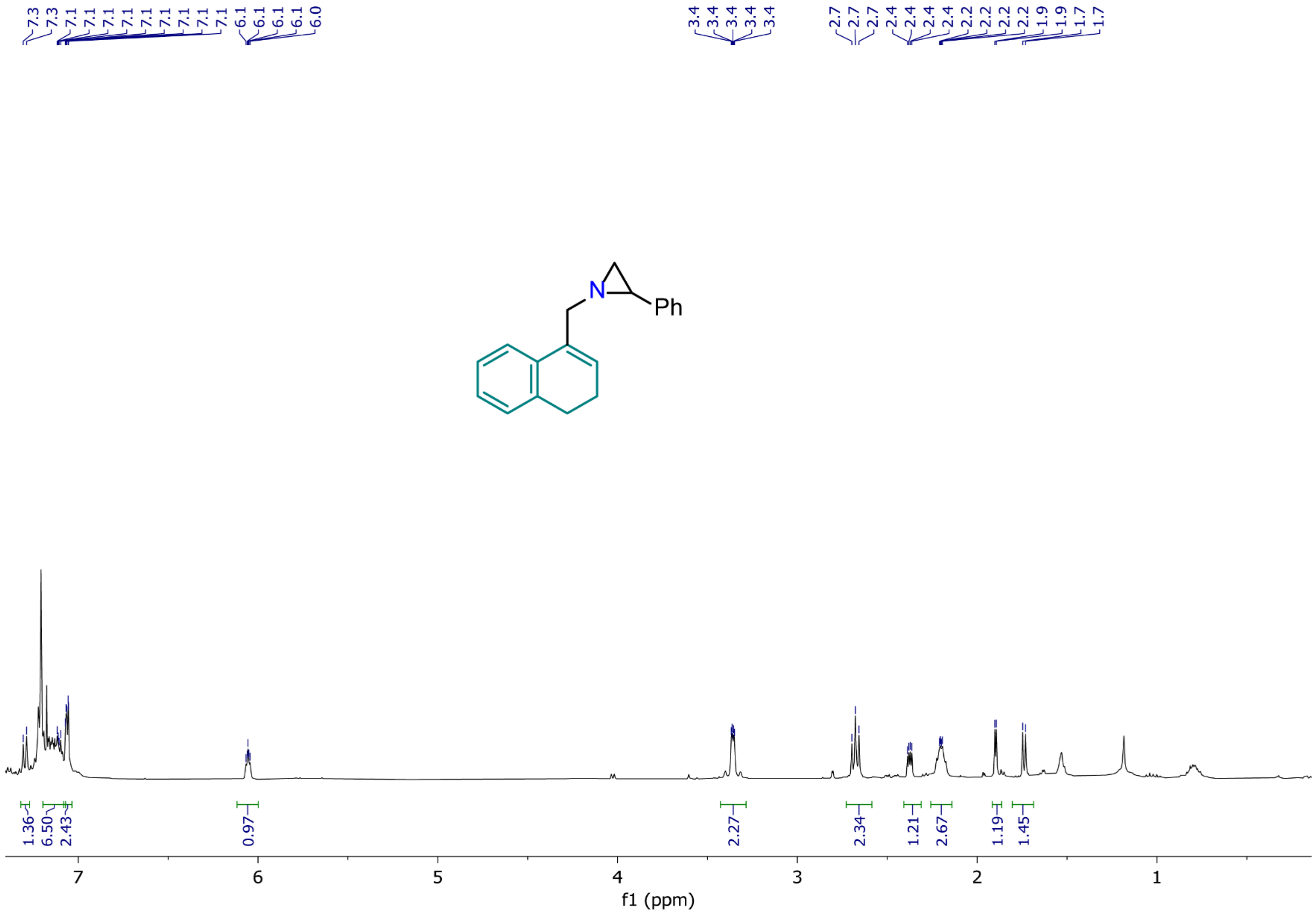

Figure S135. ^{1}H NMR spectrum of 1-((3,4-dihydronaphthalen-1-yl)methyl)-2-phenylaziridine (**11**) in $CDCl_3$ (101 MHz) at 23 °C.

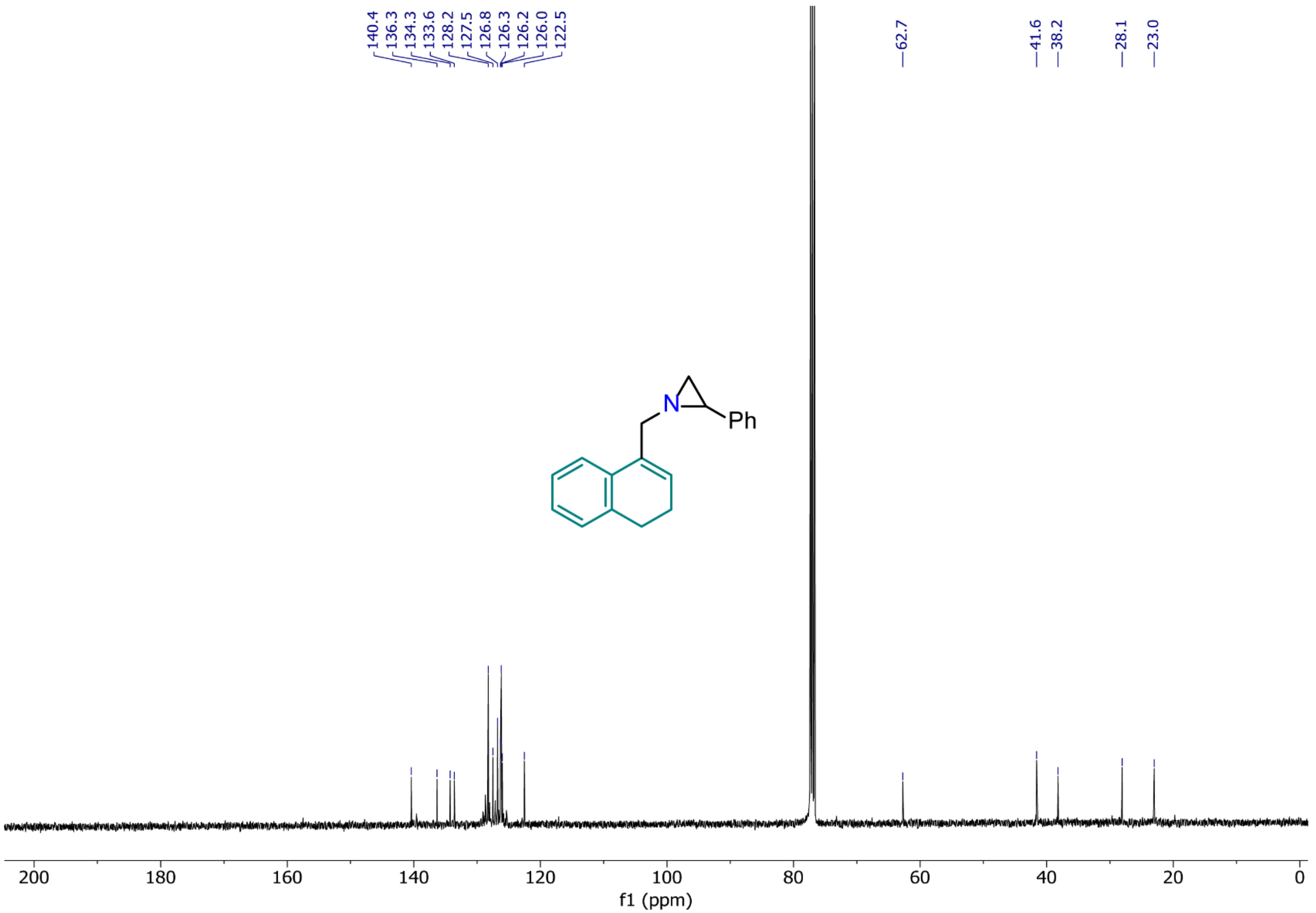

Figure S136. ^{13}C NMR spectrum of 1-((3,4-dihydronaphthalen-1-yl)methyl)-2-phenylaziridine (**11**) in $CDCl_3$ (101 MHz) at 23 °C.

H. References

(1) Pangborn, A. B.; Giardello, M. A.; Grubbs, R. H.; Rosen, R. K.; Timmers, F. J. Safe and Convenient Procedure for Solvent Purification. *Organometallics* **1996**, *15*, 1518–1520.

(2) Tan, H.; Samanta, S.; Maity, A.; Roychowdhury, P.; Powers, D. C. *N*-Aminopyridinium reagents as traceless activating groups in the synthesis of *N*-Aryl aziridines. *Nat. Commun.* **2022**, *13*, 3341.

(3) Fulmer, G. R.; Miller, A. J. M.; Sherden, N. H.; Gottlieb, H. E.; Nudelman, A.; Stoltz, B. M.; Bercaw, J. E.; Goldberg, K. I. NMR Chemical Shifts of Trace Impurities: Common Laboratory Solvents, Organics, and Gases in Deuterated Solvents Relevant to the Organometallic Chemist. *Organometallics* **2010**, *29*, 2176–2179.

(4) (a) Sheldrick, G. A short history of SHELX. *Acta Cryst. A* **2008**, *64*, 112–122; (b) O. V. Dolomanov, L. J. B., R. J. Gildea, J. A. K. Howard, H. Puschmann. OLEX2 : a complete structure solution, refinement and analysis program. *J. Appl. Cryst.* **2009**, *42*, 339–341; (c) Sheldrick, G. Crystal structure refinement with SHELXL. *Acta Cryst. C* **2015**, *71*, 3–8.

(5) Yar, M.; McGarrigle, E. M.; Aggarwal, V. K. An Annulation Reaction for the Synthesis of Morpholines, Thiomorpholines, and Piperazines from β-Heteroatom Amino Compounds and Vinyl Sulfonium Salts. *Angew. Chem. Int. Ed.* **2008**, *47*, 3784–3786.

(6) Ko, T.-M. O., L.; Moncrief, J. W. The crystal and molecular structure of the experimental anticancer agent 2-hydroxy-2-phenyl-1-aziridinoethane. *Acta Cryst. B* **1975**, *31*, 1875–1878.

(7) Wu, J.; Grant, P. S.; Li, X.; Noble, A.; Aggarwal, V. K. Catalyst-Free Deaminative Functionalizations of Primary Amines by Photoinduced Single-Electron Transfer. *Angew. Chem. Int. Ed.* **2019**, *58*, 5697–5701.

(8) Chen, L.; Guo, L.-N.; Liu, S.; Liu, L.; Duan, X.-H. Visible-light-driven palladium-catalyzed Dowd–Beckwith ring expansion/C–C bond formation cascade. *Chem. Sci.* **2021**, *12*, 1791–1795.

(9) Liu, Y.; Wang, Q.-L.; Chen, Z.; Zhou, C.-S.; Xiong, B.-Q.; Zhang, P.-L.; Yang, C.-A.; Zhou, Q. Oxidative radical ring-opening/cyclization of cyclopropane derivatives. *Beilstein J. Org. Chem.* **2019**, *15*, 256–278.

(10) Cismesia, M. A.; Yoon, T. P. Characterizing chain processes in visible light photoredox catalysis. *Chem. Sci.* **2015**, *6*, 5426–5434.

(11) Frisch, M. J.; Trucks, G. W.; Schlegel, H. B.; Scuseria, G. E.; Robb, M. A.; Cheeseman, J. R.; Scalmani, G.; Barone, V.; Petersson, G. A.; Nakatsuji, H.; et al. Gaussian 16 Rev. C.01. **2016**.

(12) Becke, A. D. Densityfunctional thermochemistry. III. The role of exact exchange. *J. Chem. Phys.* **1993**, *98*, 5648–5652.

(13) Lee, C.; Yang, W.; Parr, R. G. Development of the Colle-Salvetti correlation-energy formula into a functional of the electron density. *Phys. Rev. B* **1988**, *37*, 785–789.

(14) Grimme, S. Semiempirical GGA-type density functional constructed with a long-range dispersion correction. *J. Comput. Chem.* **2006**, *27*, 1787–1799.

(15) Grimme, S.; Ehrlich, S.; Goerigk, L. Effect of the damping function in dispersion corrected density functional theory. *J. Comput. Chem.* **2011**, *32*, 1456–1465.

(16) Petersson, G. A.; Al-Laham, M. A. A complete basis set model chemistry. II. Open-shell systems and the total energies of the first-row atoms. *J. Chem. Phys.* **1991**, *94*, 6081–6090.

(17) Krishnan, R.; Binkley, J. S.; Seeger, R.; Pople, J. A. Self-consistent molecular orbital methods. XX. A basis set for correlated wave functions. *J. Chem. Phys.* **1980**, *72*, 650–654.
(18) Clark, T.; Chandrasekhar, J.; Spitznagel, G. W.; Schleyer, P. V. R. *J. Comput. Chem.* **1983**, *4*, 294–301.
(19) Roy Dennington, T. A. K., John M. Millam. GaussView. **2016**.
